# Structure‐Based Design, Synthesis, and Evaluation of Novel Ponatinib Derivatives With a Significantly Altered Selectivity Profile

**DOI:** 10.1002/cmdc.202501007

**Published:** 2026-06-18

**Authors:** Tobias Betzholz, Ting Liu, Andreas Krämer, Verena Dederer, Stefan Knapp, Sebastian Mathea, Christian Ducho, Matthias Engel

**Affiliations:** ^1^ Pharmaceutical and Medicinal Chemistry Saarland University Saarbrücken Germany; ^2^ PharmaScienceHub (PSH) Saarbrücken Germany; ^3^ Institute of Pharmaceutical Chemistry Goethe University Frankfurt am Main Germany; ^4^ Structural Genomics Consortium Buchmann Institute of Molecular Life Sciences (BMLS) Frankfurt am Main Germany; ^5^ Frankfurt Cancer Institute Goethe University Frankfurt am Main Germany

**Keywords:** colony formation, kinase inhibitor, ponatinib derivative, selectivity modulation, structure‐based drug design

## Abstract

The protein kinase inhibitor ponatinib is an approved anti‐cancer drug that remains effective even against kinases with gatekeeper residue mutations. However, serious toxic side effects, particularly cardiotoxicity, prevent its widespread therapeutic use. The undesirable side effects have been attributed to non‐selective inhibition of more than 60 kinases from both the tyrosine and serine/threonine kinase families. With the aim of greatly reducing the spectrum of inhibited kinases, we performed structure‐based design and synthesis of novel ponatinib derivatives. By modifying the methylation pattern of the central benzamide ring and replacing the *N*‐methylpiperazine end group with a propenylamine or propylamine chain, we discovered compound **5** displaying a significantly altered target kinase profile, with B‐Raf and Flt‐1 being its main targets. This specific target combination has not yet been described for a kinase inhibitor. Furthermore, two of the original off‐target kinases that should not be inhibited to avoid cardiotoxicity, SLK and FGFR1, were no longer affected by **5**. Interestingly, **5** almost completely retained the efficacy of ponatinib in inhibiting colony formation by MDA‐MB‐231 breast cancer cells. These results might support the development of novel ponatinib analogs toward therapeutic kinase inhibitors with improved pharmacological properties.

## Graphical abstract

We developed new derivatives of the cancer drug ponatinib, significantly reducing and altering its spectrum of target kinases. Compound **5** retained the efficacy in inhibiting the colony formation of MDA‐MB‐231 cells and acted primarily on B‐Raf and Flt‐1 as its main targets in a kinase panel. A modification option was also identified to add or remove SPRK1 as an additional target.

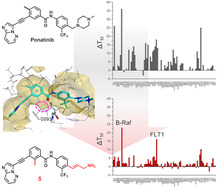

## 1 Introduction

**FIGURE 1:**
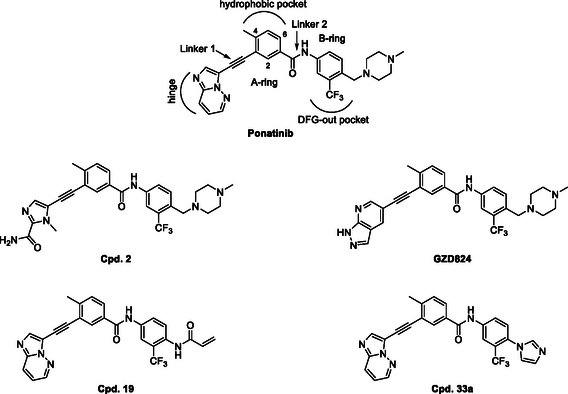
Structures of ponatinib and previously reported derivatives.

Ponatinib (brand name: Iclusig) is a multikinase inhibitor that has proven effective against several types of cancer (Figure 1) [1]. It was approved by the FDA in 2012 and is still in use for the treatment of chronic myeloid leukemia (CML), including cases with the BCR‐Abl T315I mutation conferring resistance to other tyrosine kinase inhibitors [2, 3]. However, a more wide‐spread use of ponatinib in oncology is hampered by its substantial cardiovascular risks, including arterial thrombotic events such as myocardial infarction and stroke, requiring careful monitoring of the treated patients [3, 4]. On a cellular level, cytotoxicity was observed with cardiomyocytes [5], endothelial cells [6], renal cells [7], and less strongly, with hepatocytes (differentiated HepaRG cells) [8].

The broad cytotoxicity of ponatinib is largely caused by its poor kinase selectivity. Although originally developed against BCR‐Abl tyrosine kinase, subsequent selectivity profilings revealed that the compound inhibits more than 60 kinases from both the tyrosine and serine/threonine kinase families [5]. A residual activity below 10% was noted for 32 protein kinases in the presence of 25 nM ponatinib (Figure S1, Supporting Information) [9]. Interestingly, ponatinib was additionally identified in a screening to bind to the ATP‐site of the pseudo‐kinase ROR1 with a submicromolar K_d_ value [10]. ROR1 has been recognized as an attractive anti‐cancer target as it is critically linked with solid and blood cancer progression [11].

Ponatinib possesses a unique scaffold featuring a linear ethynyl linker (Figure 1) which prevents steric collision or even interacts favorably with mutated “gate keeper” residues, such as the drug‐induced T315I mutant of BCR‐Abl [9, 12]. This distinguishes ponatinib from other tyrosine kinase inhibitors, making the structure an attractive target for chemical modification. Optimization attempts should aim at maintaining the activity towards cancer‐relevant kinases while reducing the overall target promiscuity, thus mitigating the nonselective cytotoxicity. So far, only few studies described the synthesis of new ponatinib derivatives; to explore structure–activity relationships, Zhou and colleagues replaced the imidazopyridazine ring of ponatinib by various heterocycles, of which the best compound (“cpd. 2,” Figure 1) displayed a potency toward wild‐type and mutant BCR‐Abl comparable to ponatinib, but was not further evaluated [12]. Similarly, Ke Ding's group identified another BCR‐Abl inhibitor, GZD824, which differs from ponatinib in its heterocyclic hinge‐binding moiety (Figure 1) [13]. While GZD824 strongly suppressed tumor growth in a mouse model, it showed the same broad non‐selectivity as ponatinib in a “kinomescan” screening. Hnatiuk et al. synthesized two chimeric compounds in which the basic piperazine ring of ponatinib was replaced by an imidazole as in the low‐cardiotoxic nilotinib [9]. In parallel screening assays, they found a compound (“cpd. 33a,” Figure 1) to retain anti‐cancer activity, while cytotoxicity was reduced. In a large‐scale kinase profiling study, most of the ponatinib kinase targets were still identified as inhibited, except for seven, four of which may induce cardiotoxicity (i.e., SLK, TAK1, FGFR1, and Flt‐3). Exploiting the promiscuity of ponatinib, the Rauh group synthesized an electrophilic ponatinib derivative (“cpd. 19,” Figure 1) that successfully targeted both wild‐type PDGFR‐A and its T674I mutant as well as c‐KIT in gastrointestinal stromal tumors (GIST), forming covalent adducts [14].

In the present study, we designed and synthesized several new derivatives of ponatinib, which were characterized by cell growth and colony formation assays, selectivity profiling and microscale thermophoresis (MST) measurements. Thus, we wanted to study if a notable alteration of the kinase selectivity profile of ponatinib was feasible.

## 2 Results and Discussion

### 2.1 Design Strategy

**FIGURE 2:**
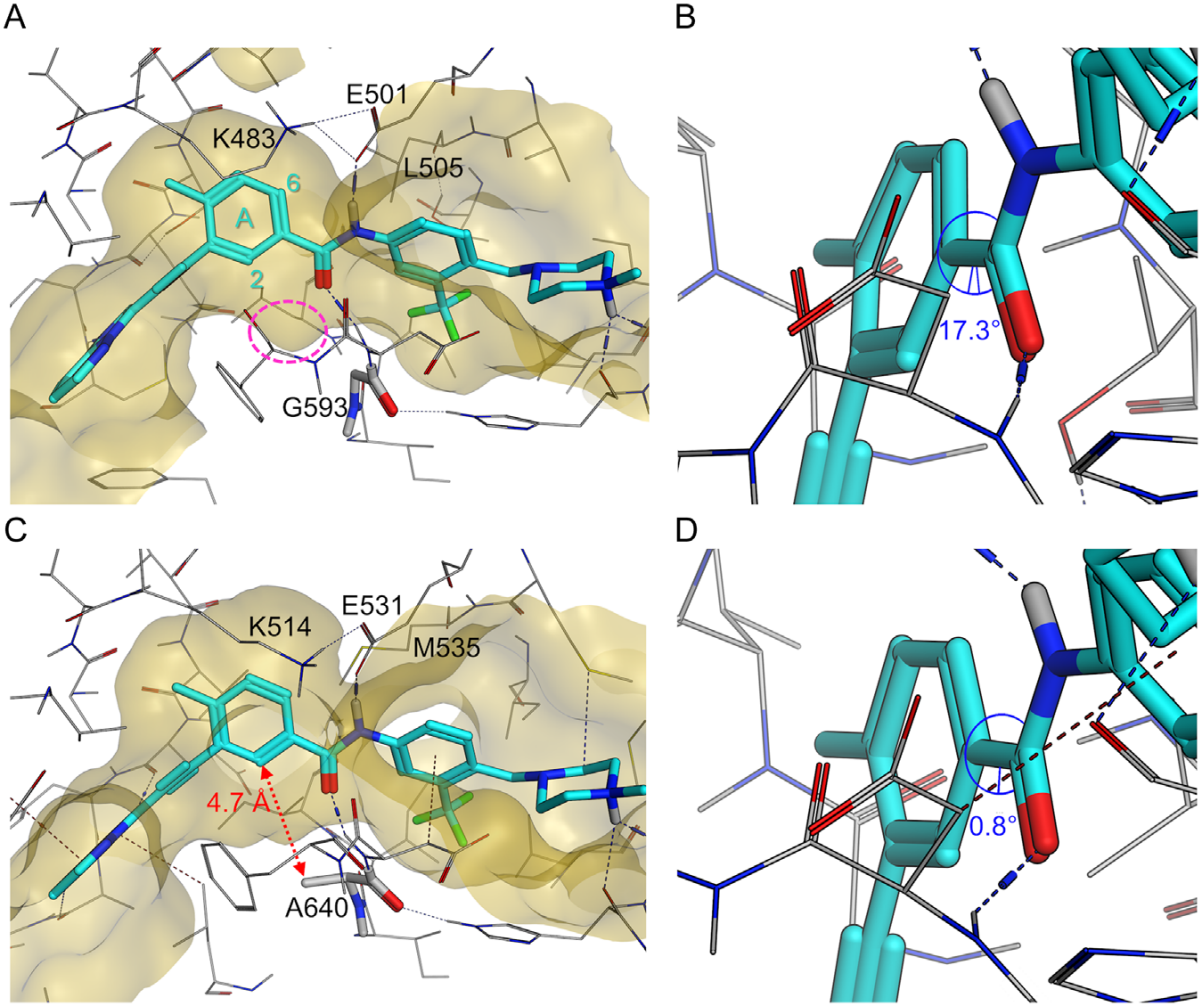
Illustration of the design concept. In B‐Raf kinase (A,B), unfilled pocket space underneath the 2‐position of the ponatinib A‐ring (circled in magenta) can accommodate a methyl group, which would not collide with Gly593 (PDB: 6P3D) [15]. Contrastingly, the corresponding space in FGFR1 kinase (C,D) is smaller due to exchange of the glycine by the homologous Ala640, where the C–C distance between the side chain and the 2‐position of the A‐ring is only 4.7 Å (indicated in red, PDB: 4V01) [16]. In ponatinib bound to B‐Raf, the pendant carboxamide is rotated out of plane by 17.3° (B), suggesting a favorable stabilization by the ortho‐effect, whereas in FGFR1, the carboxamide shows near coplanarity (D).

**FIGURE 3:**
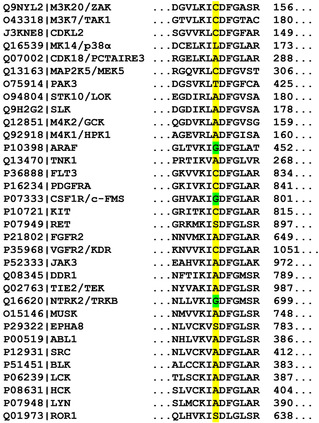
Section of a ClustalW alignment of the major kinase targets of ponatinib encompassing the space‐limiting residue for a ponatinib A‐ring substitution at position 2 (highlighted by color). The highlighted residue (e.g., Ala380 in ABL1) is not strictly conserved, leaving larger sub‐pocket space for a substituent in the case of glycine (green). The entries show (from left to right): Uniprot No., kinase name/alias, sequence segment, number of last residue shown.

No study thus far had focused on the modification of the A‐ring of ponatinib. An analysis of the published cocrystal structures of ponatinib with diverse kinases suggested that methyl substitution of the A‐ring at different positions could effectively modulate the selectivity profile. For instance, the hydrogen at the 2‐position in the ponatinib complex with FGFR1 points toward Ala640 (Figure 2), which is either conserved in most ponatinib target kinases or replaced by more voluminous residues (Figure 3, highlighted in yellow; see Figure 1 for numbering of ponatinib). These residues restrict the available sub‐pocket space for any substituent larger than hydrogen at the 2‐position. However, there are a few exceptions, in which the homologous residue is a glycine, such as in Raf kinase (Gly446 in A‐Raf and Gly593 in B‐Raf, Figure 2A and highlighted in green, Figure 3). As can be seen with B‐Raf, the sub‐pocket near the 2‐position of the A‐ring is larger than that in FGFR1 and capable of accommodating a methyl group, predicting that only this small subset of the ponatinib targets would tolerate the 2‐methyl derivative without loss of binding affinity.

Furthermore, a rotation of the carboxamide group by 17.3° relative to the A‐ring was observed with ponatinib bound to B‐Raf (Figure 2B). Contrastingly, the corresponding dihedral angle in ponatinib was 0.8° when bound to FGFR1 (Figure 2D) and also comparably small in the cocrystals with c‐src (PDB: 7WF5) [17] and Abl (PDB: 3OXZ) [12]: 9.3° and 5.3°, respectively. Therefore, the ortho‐effect imposed by the 2‐methyl toward the carboxamide group would possibly further increase selectivity for kinases that prefer binding of the A‐ring benzamide motif in a non‐coplanar conformation. In unbound ponatinib, the dihedral angle induced by a 2‐methyl group at the benzamide was calculated to be 24° (semi‐empirical model PM3, data not shown), which again predicted a more favorable binding of this derivative to B‐Raf than to, e.g., FGFR1.

In contrast, placing a methyl group at the 6‐position of the ponatinib scaffold (see Figure 1 for numbering of ponatinib) should inevitably lead to a strong decrease in activity towards most targets, as the distance between the position‐6 carbon and the oxygen of the strictly conserved glutamic acid (e.g., Glu286 in Abl) is too short (3.27 Å in Abl, PDB: 3OXZ) [12] to tolerate insertion of a methyl group along this vector. Nevertheless, we decided to try this methyl derivative as well to challenge the flexibility of the binding tunnel around the A‐ring, as it was still possible that some kinases could allow for sliding of the methyl group between the residues homologous to Glu531 and Met535 in FGFR1 (Figure 2C), thus potentially leading to retained inhibition.

In addition, we decided to replace the *N*‐methylpiperazine ring with open‐chain analogs, as previous work had already shown that this part of the molecule can be used to slightly modulate target selectivity [9]. However, we preferred to avoid installing another aromatic ring but rather a linear chain, as more than three aromatic rings in drugs have been associated with unfavorable pharmacokinetic properties [18].

### 2.2 Chemistry

**SCHEME 1:**
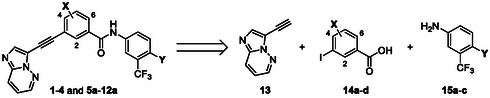
Retrosynthesis of ponatinib **1** toward the three main building blocks.

We aimed for the synthesis of ponatinib **1** and eleven novel ponatinib analogs **2–** **12** using a modular approach (for all target structures **1–** **12**, see Table 2 and Figure S2, Supporting Information). Thus, the ponatinib scaffold was dissected into three main building blocks: (i) the alkynylated aromatic heterocycle **13** representing the Western region of ponatinib; (ii) iodobenzoic acid derivatives **14a**‐**d** for introduction of the A‐ring unit; (iii) substituted anilines **15a**‐**c** for the B‐ring moiety (Scheme 1). We first prepared required building blocks that were not commercially available. Subsequently, it was attempted to identify the most efficient strategy to connect them to the target structures.

**SCHEME 2:**
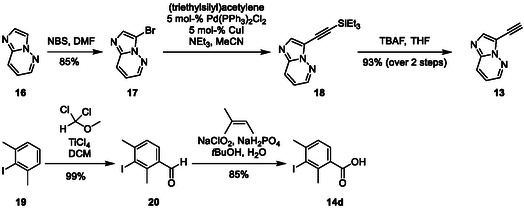
Synthesis of 3‐ethynylimidazo[1,2‐*b*]pyridazine **13** and 2,4‐dimethyl‐3‐iodobenzoic acid **14d** as building blocks.

The alkynylated aromatic heterocycle **13** was synthesized over three steps according to a previously reported method with slight modifications (Scheme 2) [19]. First, imidazo[1,2‐*b*]pyridazine **16** was brominated with *N*‐bromosuccinimide (NBS) in 85% yield. This was followed by a Sonogashira reaction with triethylsilyl‐(TES)‐protected acetylene. In the third step, the TES protecting group was cleaved with TBAF to generate the free alkyne **13** in 93% yield over the last two steps. The TES‐protected alkyne was used instead of the trimethylsilyl‐(TMS)‐congener because it is more stable. With this building block in hand, we aimed for the preparation of compound **14d** as it was not commercially available. Only two syntheses of 2,4‐dimethyl‐3‐iodobenzoic acid **14d** had been reported before [20, 21]. One published method started from 2,4‐dimethylbenzoic acid to obtain the desired compound by iodination in the 3‐position. Unfortunately, this approach turned out to be insufficient in our hands as it was not possible to separate the resultant 3‐iodo‐ and 5‐iodobenzoic acids. Another method employed 2,4‐dimethyl‐5‐nitrobenzoic acid as starting material. Beginning with iodination in the 3‐position, this procedure included reduction of the nitro group and subsequent removal of the amino group to obtain **14d**. This route gave us only small amounts of the desired product and was therefore also not suitable. Hence, we developed an alternative strategy for the synthesis of **14d**. Starting from 2‐iodo‐1,3‐dimethylbenzene **19**, this reaction sequence involved the generation of the aldehyde **20** followed by an oxidation to the corresponding acid **14d**. A typical Vilsmeier‐Haack reaction with POCl_3_ and DMF failed to generate the desired product **20**; thus, we explored other conditions. By mixing titanium(IV)‐chloride, dichloro(methoxy)methane and slowly adding **19** at −78°C, we were able to obtain the desired aldehyde **20** in quantitative yield. This reaction was followed by a Pinnick oxidation to form the desired 2,4‐dimethyl‐3‐iodobenzoic acid **14d** in 85% yield.

**SCHEME 3:**
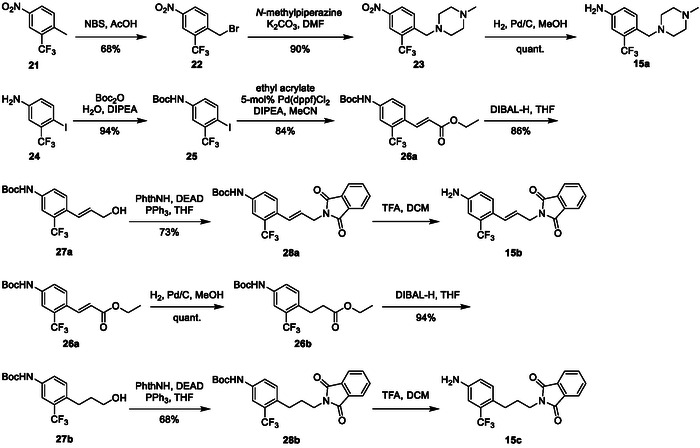
Synthesis of building blocks **15a**‐**c** for variations in the B‐ring.

We then proceeded with the synthesis of building blocks **15a**‐**c** for the substituted B‐ring unit and variations thereof (Scheme 3). For the introduction of the parent ponatinib subunit, building block **15a** was synthesized over three steps by brominating 1‐methyl‐4‐nitro‐2‐(trifluoromethyl)benzene **21** in the benzylic position with NBS (68% yield), followed by nucleophilic substitution with *N*‐methylpiperazine (90% yield) and finally the reduction of the nitro group in quantitative yield to generate the corresponding aniline derivative **15a**. The synthesis of the allyl‐ and propylamines, respectively, required more effort. Boc protection of substituted aniline **24** afforded **25** in 94% yield. Alternative approaches with trifluoroacetyl protection of the amino group gave less satisfactory results in the subsequent steps (reactions not shown). For the following Heck reaction with ethyl acrylate, we used Pd(dppf)Cl_2_ as catalyst, which gave the desired product **26a** in 84% yield. In this transformation, other Pd catalysts such as Pd(PPh_3_)_2_Cl_2_ or Pd(PPh_3_)_4_ gave no conversion toward the desired compound. The obtained 3‐arylacrylate **26a** was also used as precursor for the ethyl 3‐arylpropionate **26b**. The 3‐arylacrylate **26a** was reduced by hydrogenation to obtain **26b** in quantitative yield. The 3‐arylacrylate **26a** and ethyl 3‐arylpropionate derivative **26b** were both reduced using DIBAL‐H at −20°C. This gave the allyl alcohol **27a** in 86% yield and the 3‐arylpropanol derivative **27b** in 94% yield, respectively. Both alcohols were then subjected to Mitsunobu reactions, using phthaloyl as a late‐stage cleavable protection group, to obtain the Boc‐protected products **28a** and **28b** in 73% and 68% yield, respectively. In a final step, both aniline derivatives **28a** and **28b** were Boc‐deprotected under acidic conditions to furnish the allyl‐ and propylamine building blocks **15b** and **15c**. These deprotected anilines were freshly synthesized and directly used in the formation of the amides **30a**‐**l** and **5a**‐**12a** (vide infra) without prior purification. With these reaction sequences, we obtained the free aniline derivatives **15b** and **15c** in 49% yield over 5 and 6 steps, respectively.

**SCHEME 4:**
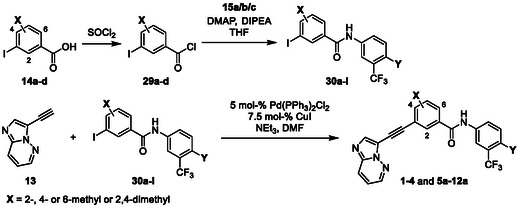
Synthesis of the amide building blocks **30a**‐**l** and subsequent Sonogashira coupling toward target compounds **1**‐**4** and protected intermediates **5a**‐**12a** (for substitution patterns **X** and **Y**, see Table S1, Supporting Information).

The different iodobenzoic acids **14a**‐**d** were converted to the corresponding acid chlorides **29a**‐**d** and then coupled with the synthesized aniline derivatives **15a**‐**c** (Scheme 4). This furnished amides **30a**‐**l** in 31%–75% yield. Finally, the newly synthesized iodinated amides **30a**‐**l** were coupled with 3‐ethynylimidazo[1,2‐*b*]pyridazine **13** to form ponatinib **1** and its derivatives **2**‐**4** as target structures and the phthaloyl‐protected intermediates **5a**‐**12a** in 21%–75% yield.

**SCHEME 5:**
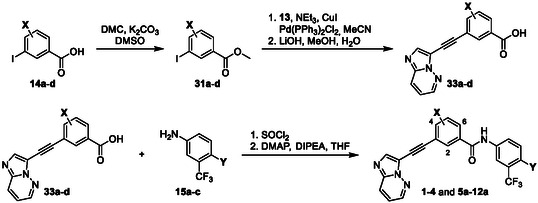
Revised order of reaction steps: Sonogashira coupling towards the alkyne building blocks **33a**‐**d** and subsequent amide coupling towards target compounds **1–** **4** and protected intermediates **5a–** **12a**.

Unfortunately, the aforementioned strategy only gave the 6‐methyl‐substituted derivatives (**3**, **7a**, **11a**) in rather good yields (46‐75%) in the Sonogashira reaction step (see Table S1, Supporting Information). The reaction toward the desired products showed limited to no conversion for the 2‐, 4‐, and 2,4‐substituted derivatives. This problem was overcome by an inversion of reaction steps. In a first step, we protected the benzoic acids **14a**‐**d** with dimethyl carbonate (DMC) to obtain the corresponding methyl esters **31a**‐**d** in quantitative yields for the subsequent Sonogashira reaction. The Sonogashira coupling then gave the corresponding esters **32a**‐**d** in 39‐97% yield. After the Sonogashira step, esters **32a**‐**d** were saponified with lithium hydroxide in a mixture of methanol and water. The quantitatively obtained benzoic acids **33a**‐**d** were once again converted to the acid chlorides using thionyl chloride and then coupled with the different aniline derivatives **15a**‐**c** to obtain the desired target compounds **1–** **4** and protected intermediates **5a**‐**12a** in substantially better yields of 44%–86% (Scheme 5) (see Table S2, Supporting Information).

**SCHEME 6:**
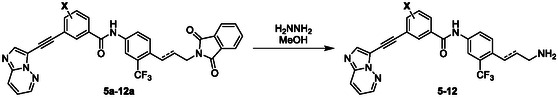
Deprotection (hydrazinolysis) of intermediates **5a–** **12a** toward the target compounds **5–** **12**.

In a last step, the free allyl‐ and propylamines **5–** **12** were generated by hydrazinolysis of the phthalimides **5a–** **12a** (Scheme 6). These transformations gave the desired target compounds **5–** **12** in good yields of 76%–93% (see Table S3, Supporting Information). The structures of all final compounds are summarized in Figure S2, Supporting Information. In addition, truncated versions of the target compounds **1–** **4** without a basic residue were also synthesized, compounds **1S–** **4S** (Figure S2). The synthetic procedures and yields for these compounds are reported in the Supporting Information (cf. Scheme S1 and Table S4).

### 2.3 Antiproliferative Activity

Ponatinib (**1**) and its newly synthesized derivatives **2–** **12** were first screened against the triple negative breast cancer cell line MDA‐MB‐231 to evaluate their potential anti‐tumor activities. MDA‐MB‐231 is a malignant cell line with high capacity to metastasize in vivo [22], expressing many oncogenic protein kinases that are targets of ponatinib, e.g., c‐src, FGFR1, PDGFR, MEKK2, B‐Raf, and ephrin receptor kinase [23, 24, 25]. Therefore, this cell line was a suitable model to find out whether ponatinib derivatives with a more restricted target profile would continue to inhibit tumor growth, ideally without further inhibition of FGFR1.

Assuming that there are no major differences in the compounds’ cell permeabilities, the measured cytotoxic effects can be expected to correlate with changes in the spectrum of inhibited kinases. As can be seen from Table 1, any positional shift of the methyl group resulted in a decrease in anti‐proliferative activity relative to ponatinib **1**. In particular, the *ortho*‐methyl derivative **3** (methyl at position 6 in the ponatinib scaffold) was considerably weaker. Measurements in the presence versus absence of serum indicated that serum caused a moderate loss of activity, probably due to binding to serum proteins.

**TABLE 1:** Antiproliferative activity of the pilot compound series 1–4 against the MDA‐MB‐231 cell line.

Cpd.	Structure	GI_50_ ± SD, µM	
		5% serum	0.1% serum
**1**	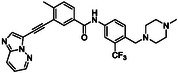	0.30 ± 0.07	0.28 ± 0.02
**2**	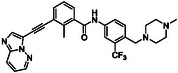	4.1 ± 0.4	4.3 ± 0.5
**3**	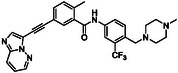	15 ± 1	n.d.
**4**	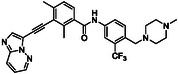	3.9 ± 0.4	1.8 ± 0.2

**TABLE 2:** Cytotoxicity of the most potent compounds **4–12** and their physicochemical parameters.

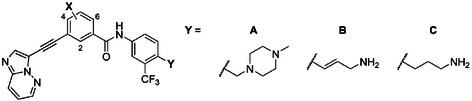								
			MDA‐MB‐231			MRC‐5		
	X	Y	aGI_50_ ± SD, µM 5% serum, low cell density	bGI_50_ ± SD, µM no serum, low cell density	cGI_50_ ± SD, µM no serum, high cell density	dGI_50_ ± SD, µM 5% serum, low cell density	epKa	eclogD
**1** (ponatinib)	4‐Methyl	A	0.30 ± 0.07	0.28 ± 0.02	0.54 ± 0.02	1.6 ± 0.4	7.5	3.4
**4**	2,4‐Dimethyl	A	3.9 ± 0.4	1.8 ± 0.2	5.1 ± 0.6	5.1 ± 0.3	7.5	3.9
**5**	2‐Methyl	B	2.8 ± 0.1	0.82 ± 0.04	5.6 ± 0.5	6.6 ± 0.3	9.0	2.2
**6**	4‐Methyl	B	1.7 ± 0.2	0.77 ± 0.05	1.7 ± 0.3	11 ± 3	9.0	2.2
**7**	6‐Methyl	B	>11	n.d.	n.d.	n.d.	9.0	2.2
**8**	2,4‐Dimethyl	B	2.4 ± 0.1	1.1 ± 0.1	3.2 ± 0.4	<5^f^	9.0	2.4
**9**	2‐Methyl	C	>5	n.d.	n.d.	n.d.	10.2	1.2
**10**	4‐Methyl	C	0.92 ± 0.08	0.53 ± 0.1	1.5 ± 0.1	6.4 ± 0.5	10.2	1.2
**11**	6‐Methyl	C	>5	n.d.	n.d.	n.d.	10.2	1.2
**12**	2,4‐Dimethyl	C	4.3 ± 0.8	1.1 ± 0.2	n.d.	n.d.	10.2	1.5

*Note:* All data shown are mean values of at least two independent measurements.

a GI_50_ was determined starting with 6000 cells.

b GI_50_ was determined for compounds showing a GI_50_ < 4 µM with serum, seeding 6000 cells.

c GI_50_ determined with 15 000 cells seeded.

d GI_50_ was determined with 6000 cells seeded.

e Values were calculated using ACD/Labs software.

f 82% inhibition at 5 µM.

We reasoned that the altered steric demands of the A‐ring modifications might induce a slight adaptation of the overall binding position, entailing weaker interactions of the imidazopyridazine and/or the *N*‐methylpiperazine moiety in the binding pocket. We speculated that this drawback could be overcome by combining the methyl‐permutated and dimethylated A‐ring with open‐chain analogs of the *N*‐methylpiperazine. Indeed, the latter “de‐rigidification” strategy compensated for a part of the activity losses induced by the modified A‐ring (Table 2), albeit not in all cases. An initial screening was again performed in the presence of 5% serum, revealing that the propenylamine chain (**5–** **8**) significantly improved the cellular activity of the 2‐methylated and the 2,4‐dimethylated A‐ring analogs (compare **2** with **5** and **4** with **8**), which was also found under serum‐free assay conditions. This activity enhancement was not observed with the saturated propylamine chain (cpds. **9** and **12**); it might be speculated that this was attributable to the rather high basicity of the propylamine (cf. pKa values in Table 2), potentially impairing cell penetration. However, the propylamine was superior in case of the 4‐methyl derivative **10** when compared to the propenylamine congener **6**.

Overall, **10** was identified to be the most potent compound of our series, being slightly less potent than ponatinib (**1**). In addition, **10** showed only a little loss of activity in the presence of serum compared to the serum‐free conditions. The 2‐methyl‐ and the 2,4‐dimethyl derivatives, i.e., **5** and **8**, respectively, turned out to be the second most potent compounds. In contrast, the congeners with 6‐methylation (ponatinib numbering) at the A‐ring, i.e., **3**, **7** and **11** (Tables 1 and 2), were essentially inactive in cells, suggesting that the inhibitory potency against kinases was abolished, as predicted by the modeling (vide supra).

We were also interested in investigating the influence of cell–cell contacts on the sensitivity toward the most potent inhibitors. Ponatinib (**1**) and derivatives **6** and **10** displayed only a moderate drop of potency to suppress cell growth, while the 2‐methyl‐substituted derivatives **5** and **8** had a significantly weaker effect on cell viability (Table 2, “no serum, high cell density”). **5** in particular inhibited the growth of MDA‐MB‐231 cells 7‐fold less effectively when cell–cell contacts could be established. Overall, changes in the methylation pattern of the A‐ring led to strong structure–activity relationships for the ponatinib derivatives in terms of cellular activity. It is evident that the number and position of the methyl groups effected both quantitative and qualitative changes in the inhibition of cell growth.

Next, we wanted to analyze the effects of the most effective compounds on non‐cancerous cells and chose human MRC‐5 lung fibroblasts for this purpose. Compared with ponatinib, compounds **6** and **10** showed a slight improvement in tumor cell selectivity, as they inhibited the growth of MRC‐5 cells with 6.5‐ and 7‐fold lower potency, respectively, compared with MDA‐MB‐231 cells under the same experimental conditions (Table 2). However, compound **5** did not show a higher selectivity ratio for inhibiting MRC‐5 versus MDA‐MB‐231 cell growth than ponatinib. Previous studies suggested that growth factor‐induced proliferation of fibroblasts in cell culture depends on the activation of the MEK signaling pathway by B‐Raf [26, 27]. Since our compounds retained activity against B‐Raf as the main target (see below), this could explain why the growth of MRC‐5 fibroblast cells was still significantly inhibited.

### 2.4 Potency to Suppress Colony Formation

A primary goal of this study was to develop compounds with a larger safety window between tumor cell‐directed and general cytotoxicity. We therefore decided to further characterize **5**, which was significantly more active against single cells than against cells establishing cell–cell contacts. Such a selective cytotoxicity could make it possible to combat predominantly disseminating tumor cells without affecting cells embedded in tissue. Thus, we wanted to investigate the efficacy of **5** in suppressing the capacity of seeded cells to produce colonies. For comparison, **10** as the most potent congener of our series was included, as well as ponatinib (**1**) as a reference. The colony formation assay was performed according to Franken et al. [28] using the MDA‐MB‐231 cell line.

**FIGURE 4:**
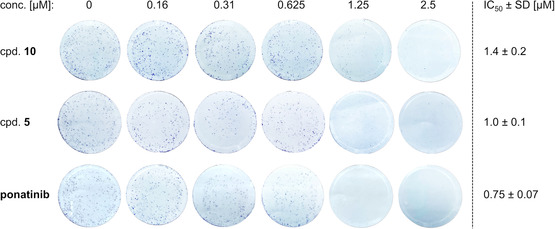
Ponatinib derivatives **5** and **10** effectively inhibit colony formation from single cells. MDA‐MB‐231 cells were plated in six‐well plates at low density and treated with the compounds at the indicated concentrations. After 11 days, cells were stained with crystal violet. The image shows the result of one representative experiment out of three (a high resolution version of this image is available in the Supporting Information).

It was not surprising that the multikinase inhibitor ponatinib most strongly suppressed colony formation from single cells (IC_50_ = 0.75 µM, Figure 4). This finding was in the range of previously reported values for ponatinib: In previous studies, the concentration needed for 50% inhibition of colony formation compared with DMSO was below or around 1 µM in several nonsmall cell lung cancer cell lines [29] and neuroblastoma cell lines [30]. Remarkably, compound **5**, with an IC_50_ value of 1.0 µM, was only slightly less potent in inhibiting colony formation and even more effective than **10** (IC_50_ = 1.4 µM).

### 2.5 Selectivity Profiling

**FIGURE 5:**
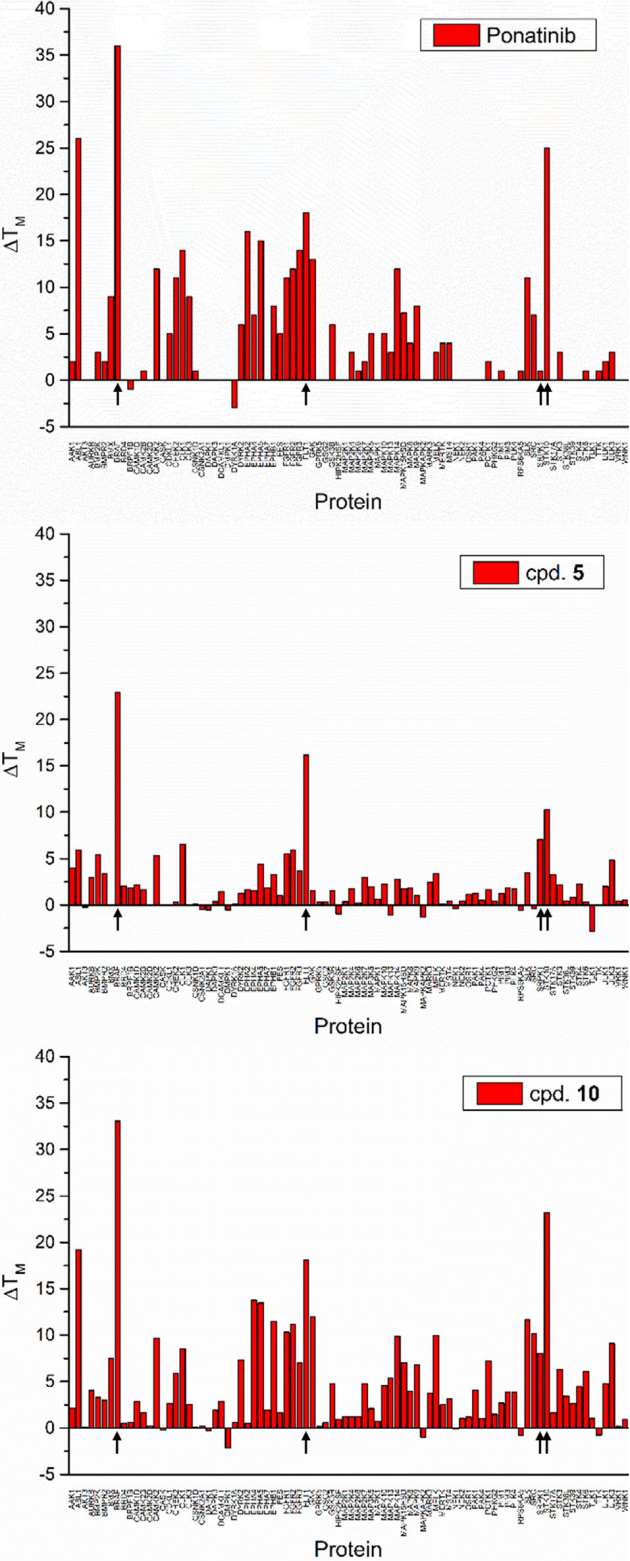
Investigation of the kinase target spectrum of ponatinib (**1**) vs. new ponatinib analogs **5** and **10**. The thermal stabilizations due to ligand binding (expressed as ΔTm values), that are indicative of relative binding affinities, were determined in a representative panel of kinases using DSF. Arrows denote the main target kinases of **10** in each diagram, from left to right: B‐Raf, Flt‐1, SRPK1, STK10.

In light of these notable findings, we aimed to analyze the changes in the kinase target spectrum of the new inhibitors **5** and **10**. To this end, we measured the thermal stabilization of 90 representative kinases by these compounds using differential scanning fluorimetry (DSF) and compared them with the ponatinib data. As for **10**, replacement of the basic *N*‐methylpiperazine moiety with the more flexible aminopropyl group mostly retained the target spectrum of ponatinib (**1**) (Figure 5 as well as Figure S3 and Table S5, Supporting Information). However, some differences were noted: some additional affinities, e.g., toward SRPK1 and MELK occurred, while the thermal stabilization of Clk3 and FGFR3 kinase by **10** was significantly reduced. Interestingly, the isoform selectivity toward the Eph kinase family changed from EphA2 to EphA4. Overall, the effect of the basic side chain modification was rather moderate, although the number of strongly affected kinases (ΔTm ≥ 15°C) in our panel decreased from six with ponatinib to four with **10** (Figure S3, Supporting Information).

This was in marked contrast to the methyl shift at the benzamide moiety; when the methyl group was moved to the 2‐position in **5**, only four kinases in our panel were thermostabilized by more than 7°C: B‐Raf, Flt‐1, SRPK1 and STK10. Intriguingly, the original target Abl‐1 was much less affected by **5** than by **10** or ponatinib (**1**). A closer examination revealed that the activity against B‐Raf, Flt‐1, and STK10 was retained from the original ponatinib target ensemble, while the splicing factor kinase SRPK1 was newly added due to the replacement of *N*‐methylpiperazine by the open‐chain analogs. In summary, it can be concluded that with **5**, the target spectrum was greatly reduced or, in the case of SRPK1, also qualitatively altered.

### 2.6 Additional Investigations Using MST

Next, we performed MST measurements to compare the binding affinities of the new compounds **2**‐**12** with those of ponatinib using an exemplary target protein, i.e., the pseudo‐kinase ROR1. The K_d_ value for the reference compound ponatinib (**1**) was determined to be ca. 230 nM (Table 3), which had translated to a ΔTm value of 4.1°C in an earlier DSF assay [10]. In line with our DSF‐based selectivity profiling results, we found that shifting the methyl group on the benzamide ring or dimethylating it led to a significant loss of binding affinity also for ROR1. The only exceptions were compounds **6** and **10**, in which only the basic moiety had changed compared to ponatinib (**1**), but not the position of the methyl group (K_d_ values: 1.2 and 0.79 µM, respectively, Table 3). According to our modeling predictions, a 2‐methyl group on the A‐ring is expected to collide with serine‐632 in ROR1. Indeed, all congeners with the latter modification, i.e., **2**, **4**, **5**, **8**, **9**, and **12**, exhibited a considerable loss of binding affinity to ROR1, although the more adaptable propylamine end chain in **9** – but not the more rigid propenylamine chain in **5** – recovered some of the binding strength.

**TABLE 3:** Binding affinities of ponatinib (**1**) versus the new derivatives **2–12** toward the pseudokinase ROR1.

Cpd.	K_d_ ± SD, μM	Cpd.	K_d_ ± SD, μM
**1**	0.23 ± 0.12	**7**	26 ± 26
**2**	26 ± 13	**8**	30 ± 10
**3**	22 ± 21	**9**	3.9 ± 2.9
**4**	17 ± 21	**10**	0.79 ± 0.82
**5**	26 ± 11	**11**	23 ± 10
**6**	1.2 ± 0.9	**12**	32 ± 8

These data confirmed that the methylation pattern at the A‐ring strongly modulates the target spectrum of the ponatinib derivatives, leading to selective inhibition of fewer kinases. The importance of the basic residue at the B‐ring for selectivity was also indicated here. In order to determine its contribution to binding to ROR1, the MST measurement was also performed with the truncated compounds **1S**‐**4S** without the basic residue (Figure S2 and Table S6, Supporting Information). This resulted in a K_d_ value of 2.7 µM for the truncated ponatinib analog **1S**, indicating that the ionic interaction of the tertiary amine increases the binding affinity of ponatinib by approximately 12‐fold. Obviously, the more flexible propylamine chain did not achieve the same degree of binding enhancement.

## 3 Conclusion

In an attempt to add new value to the interesting ponatinib scaffold, we have established targeted, structure‐guided modifications to the benzamide ring and the basic unit of ponatinib. The replacement of the *N*‐methylpiperazine by propylamine or propenylamine did not significantly decrease the number of inhibited kinases in a DSF profiling screen (cf. **10**, Figure 5). However, it induced some small qualitative changes in the kinase target spectrum. Obviously, the binding of **10** to SRPK1 was strongly enhanced by the more flexible and therefore adaptable side chain. In contrast, changing the methylation pattern at the benzamide ring (A‐ring in Figure 1) resulted in a steep SAR. A 2‐methyl substituent tremendously reduced the number of affected kinases, as seen with **5**. The 6‐methyl derivatives (ponatinib numbering) **3**, **7,** and **11** were not analyzed in our kinase test panel. However, their lack of activity in our cellular assays strongly suggested that a methyl group at position 6 totally abolished the activity toward all kinases regulating cell growth, in line with our modeling prediction (cf. Figure 2). In **5**, the slight target shift caused by the propenylamine was combined with the strong selectivity‐modulating effect of the 2‐methyl group, resulting in a significantly reduced number of targets and a novel selectivity profile. It is important to note that two of the target kinases suspected of inducing cardiotoxicity when inhibited by ponatinib, SLK and FGFR1, were also included in that kinase panel and were no longer affected by **5**.

The strong effect of the 2‐methyl group on the selectivity of the scaffold towards B‐Raf was consistent with our modeling results, predicting tolerance against this modification by a small number of kinases possessing a small glycine in the critical position highlighted in Figure 3. It was found indeed that most kinases with a larger residue at this position could no longer bind **5** (e.g., FGFR1), but this did not necessarily apply to all previous target molecules, as exemplified by Flt‐1. It is likely that a small number of kinases, including Flt‐1, exhibit greater flexibility in the ATP binding site around the A‐ring, allowing them to compensate for the stronger steric requirements of the 2‐methyl group.

According to our DSF assay results, **5** bound most strongly to B‐Raf and Flt‐1 with ΔTm values of 22.9 and 16.2°C, respectively, suggesting that these were the main targets. Do the measured ΔTm values indicate relevant target inhibition to mediate the observed cellular effects? In order to estimate inhibitory potencies based on the ΔTm values, we compared the percentage inhibition values at 23.5 nM ponatinib as published in [9] with the corresponding ΔTm values measured by us with ponatinib (Table S7, Supporting Information). The target kinases B‐Raf, Flt‐1 and STK10 were at least 67% inhibited by ponatinib at 23.5 nM, pointing to IC_50_ values below that concentration. Although there is no simple linear relationship between ΔTm and enzymatic inhibition, the moderate reductions of the ΔTm values against B‐Raf and Flt‐1 with **5**, 36% and 10%, respectively, suggested that the IC_50_ values of **5** may still be in the double‐digit nM range. Against STK10, the ΔTm value of **5** had decreased 2.5‐fold; however, as the ΔTm of 25°C for ponatinib corresponded to a nearly 100% inhibition, the ΔTm value of 10.3°C might still indicate a nM inhibition of STK10 by cpd. **5**. This is also supported by an earlier study conducted by one of us using the same DSF conditions with a panel of >100 kinases, where ΔTm values >5°C were associated with double‐digit nM potencies [31]. No comparative assessment could be made for SRPK1 in a similar way, as this kinase was not a target of ponatinib (Table S7, Supporting Information).

In summary, based on the measured Tm shifts, the inhibitory potency of **5** against B‐Raf and Flt‐1 can be estimated sufficiently high that inhibition of these kinases in cells could be possible. Although further target kinases of **5** may be identified in kinome‐wide screenings, to our knowledge no other compound has been described so far that is a dual inhibitor of these two kinases with otherwise acceptable selectivity.

FLT1 (VEGFR1) has been identified, along with BRAF, BRIP1, and FGF10, as part of a gene signature predictive of response to checkpoint inhibitor therapies in non‐small cell lung cancer (NSCLC) [32]. BRAF mutations are known to occur in various types of cancer, including NSCLC, and B‐Raf inhibitors are already used to treat NSCLC [33]. Therefore, a dual inhibitor that inhibits both B‐Raf and Flt‐1 could be a promising strategy for improving therapeutic outcomes in NSCLC, especially in combination with immunotherapies [32]. Due to its effect in particular on single cells, **5** or further optimized derivatives thereof might be tested in suitable models for their potential to diminish formation of metastases.

In summary, this study demonstrates how minor structural changes in an established protein kinase inhibitor can significantly affect its selectivity profile. Future work in our laboratory will include the investigation of other substituents on the A‐ring, e.g., halogens, which are likely to have a strong impact on potency and selectivity, given the steep SAR we observed with the methyl groups. Different combinations with terminal basic chains are also promising for further improving the desired selectivity profile. For example, this should allow to add or remove SRPK1 as an additional target. Eventually, our approach could also be used to significantly limit the number of targets of other kinase inhibitors with rather low selectivity, such as imatinib, sorafenib, or regorafenib.

## 4 Experimental Section

### 4.1 General methods

^1^H and ^13^C NMR spectra were recorded on a Bruker Avance‐500 UltraShield spectrometer. ^19^F NMR spectra were recorded on a Bruker Avance‐II‐400 Ultrashield Plus spectrometer. Chemical shifts (δ) are given in ppm. Coupling constants (*J*) are reported in Hz. The signals were assigned using ^1^H,^1^H‐COSY, ^1^H,^13^C‐HSQC, and ^1^H,^13^C‐HMBC spectra. All ^13^C NMR spectra are ^1^H‐decoupled. All spectra were recorded at room temperature and referenced internally to solvent reference frequencies. Reactions were monitored via thin‐layer chromatography (TLC) on precoated Alugram plates from VWR or Macherey–Nagel. Spots were visualized with UV light (254 nm or 365 nm) or by staining with a solution of vanillin (5 g) in a mixture of conc. H_2_SO_4_ (25 mL), glacial acetic acid (80 mL) and methanol (680 mL). Flash column chromatography was performed using VWR silica gel (particle size 40–63 μm). Acetonitrile (4 Å), dichloromethane (4 Å), *N*,*N*‐dimethylformamide (DMF, 3 Å), and THF (4 Å) were dried over molecular sieves and stored under argon. All other starting materials and solvents used in this study were sourced from Merck (Darmstadt, Germany) or Thermo Fisher (Waltham, USA). Mass spectra were recorded with a Finnigan Surveyor MSQ Plus spectrometer. High resolution mass spectra (HRMS) were recorded with a Thermo scientific Orbitrap Q Exactive spectrometer.

### 4.2 Synthesis

#### 4.2.1 General Procedure (A) for the Synthesis of Intermediates 31a‐d

The benzoic acids **14a**‐**d** (1.91 mmol, 1.0 eq.) were dissolved with two equivalents of dimethyl carbonate in DMSO (10 mL). To this mixture, K_2_CO_3_ (0.76 mmol, 0.4 eq.) was added, and the solution was heated at 90°C for 20 h. The reaction mixture was diluted with EtOAc (10 mL), washed with water (2 × 20 mL) and brine (20 mL). The organic layer was dried over Na_2_SO_4_, filtered and evaporated under reduced pressure to give the pure products **31a**‐**d**.

#### 4.2.2 General Procedure (B) for the Synthesis of Intermediates 32a‐d

One equivalent of the corresponding methyl benzoates **31a**‐**d** (1.81 mmol, 1.0 eq.), 3‐ethynylimidazo[1,2‐*b*]pyridazine **13** (2.17 mmol, 1.2 eq.), Pd(PPh_3_)_2_Cl_2_ (91 μmol, 5 mol‐%), and CuI (91 μmol, 5 mol‐%) were dissolved in MeCN (5 mL). To this mixture, DIPEA (3.62 mmol, 2.0 eq.) was added. The reaction mixture was heated at reflux for 16 h. After cooling to rt, the mixture was filtered, washed with brine (3 x 25 mL) and evaporated under reduced pressure. Flash chromatography (CH_2_Cl_2_/MeOH 99:1) gave the desired products **32a**‐**d**.

#### 4.2.3 General Procedure (C) for the Synthesis of Intermediates 33a‐d

Methyl benzoates **32a**‐**d** (0.687 mmol, 1.0 eq.) and LiOH (2.06 mmol, 3.0 eq.) were dissolved in a 1:1 mixture of MeOH and water (2 mL) and heated at 70°C for 12 h. After cooling to rt, the mixture was acidified with HCl (2 M) until the acid precipitated. The mixture was extracted with EtOAc (5 × 25 mL), the combined organics were dried over Na_2_SO_4_ and evaporated under reduced pressure. The products **33a**‐**d** were used for the next step without further purification.

#### 4.2.4 General Procedure (D) for the Synthesis of Target Compounds 1–4

The respective methyl benzoate **32a**‐**d** (1.0 eq.) and LiOH (3.0 eq.) were stirred at 70°C in a 1:1 mixture of MeOH and water. The resultant benzoic acids **33a**‐**d** (0.687 mmol, 1.0 eq.) were heated under reflux in SOCl_2_ (2.0 mL) for 1 h. The mixture was evaporated in vacuo; the residue was dissolved in THF (1.0 mL) and added dropwise to a solution of **15a** (0.701 mmol, 1.02 eq.), DMAP (0.034 mmol, 5 mol‐%) and DIPEA (0.824 mmol, 1.2 eq.) in THF (3.0 mL). The mixture was stirred at rt for 16 h, quenched with water and extracted with EtOAc. The combined organics were washed with brine and dried over Na_2_SO_4_. The mixture was filtered and evaporated in vacuo. Flash chromatography (CH_2_Cl_2_/MeOH 95:5) gave the desired products **1**‐**4**.

#### 4.2.5 General Procedure (E) for the Synthesis of Protected Intermediates 5a‐8a

One equivalent of **28a** (0.221 mmol, 1.0 eq.) was stirred in a mixture of 1 mL TFA and 1 mL CH_2_Cl_2_ for 30 min. The mixture was evaporated in vacuo, the residue was dissolved in THF (1.0 mL) and added to a solution of DMAP (0.01 mmol, 5 mol‐%) and DIPEA (0.265 mmol, 1.2 eq.) in THF (3.0 mL). One equivalent of the corresponding benzoic acids **33a**‐**d** (0.221 mmol, 1.0 eq.) were heated under reflux in SOCl_2_ (2.0 ml) for 1 h. The mixture was evaporated in vacuo, the residue was dissolved in THF (1.0 mL) and added dropwise to the reaction mixture. The mixture was stirred at rt for 16 h, quenched with water and extracted with EtOAc. The combined organic layers were washed with brine and dried over Na_2_SO_4_. The mixture was filtered and evaporated in vacuo. Flash chromatography (CH_2_Cl_2_/MeOH 95:5) gave the desired products **5a**‐**8a**.

#### 4.2.6 General Procedure (F) for the Synthesis of Protected Intermediates 9a‐12a

One equivalent of **28b** (0.221 mmol, 1.0 eq.) was stirred in a mixture of 1 mL TFA and 1 mL CH_2_Cl_2_ for 30 min. The mixture was evaporated in vacuo, the residue was dissolved in THF (1.0 mL) and added to a solution of DMAP (0.01 mmol, 5 mol‐%) and DIPEA (0.265 mmol, 1.2 eq.) in THF (3.0 mL). One equivalent of the corresponding benzoic acids **33a**‐**d** (0.221 mmol, 1.0 eq.) was heated under reflux in SOCl_2_ (2.0 ml) for 1 h. The mixture was evaporated in vacuo, the residue was dissolved in THF (1.0 mL) and added dropwise to the reaction mixture. The mixture was stirred at rt for 16 h, quenched with water, and extracted with EtOAc. The combined organic layers were washed with brine and dried over Na_2_SO_4_. The mixture was filtered and evaporated in vacuo. Flash chromatography (CH_2_Cl_2_/MeOH 95:5) gave the desired products **9a**‐**12a**.

#### 4.2.7 General Procedure (G) for the Synthesis of Target Compounds 5–12

One equivalent of the corresponding phthalimides **5a** **–12a** (0.020 mmol, 1.0 eq.) was dissolved in MeOH (1.0 mL). Hydrazine monohydrate (0.100 mmol, 5.0 eq.) was added, and the reaction mixture was stirred at rt for 45 min. The mixture was evaporated in vacuo. Preparative chromatography (acetonitrile/water) gave the desired products **5‐12**.

#### 4.2.8 3‐(Imidazo[1,2‐b]pyridazin‐3‐ylethynyl)‐4‐methyl‐N‐(4‐((4‐methylpiperazin‐1‐yl)methyl)‐3‐(trifluoromethyl)phenyl)benzamide (1)

Synthesized according to General Procedure D, Colorless powder; 274 mg, 0.515 mmol, 75% yield. *R*_f_ = 0.17 (CH_2_Cl_2_/MeOH 98:2); ^1^H NMR (500 MHz, MeOD‐d_4_): δ [ppm] = 8.71–8.68 (m, 1H, H‐6′′), 8.18–8.15 (m, 3H, H‐2/H‐2′/H‐4′′), 8.15–8.13 (m, 1H, H‐2′′), 8.02–7.97 (m, 1H, H‐6′), 7.93–7.88 (m, 1H, H‐6), 7.78–7.74 (m, 1H, H‐5′), 7.50–7.47 (m, 1H, H‐5), 7.47–7.43 (m, 1H, H‐5′′), 3.78 (s, 2H, H‐7′), 3.72–2.20 (m, 8H, H‐1′′′′/H‐2′′′′), 2.91 (s, 3H, H‐3′′′′), 2.66 (s, 3H, CH_3_); ^13^C NMR (126 MHz, MeOD‐d_4_): δ [ppm] = 167.73 (C = O), 146.63 (C‐6′′), 145.71 (C‐4), 140.77 (C‐3′′), 139.80 (C‐1′), 136.57 (C‐2′′), 132.79 (C‐5′), 131.89 (C‐2), 131.16 (C‐5), 130.28 (q, ^2^ *J*_CF_ = 30.3 Hz, C‐3′), 129.31 (C‐6), 128.95 (C‐1), 126.78 (C‐4′), 125.99 (C‐4′′), 125.70 (q, ^1^ *J*_CF_ = 290.5 Hz, CF_3_), 125.14 (C‐6′), 123.78 (C‐3), 121.52 (C‐5′′), 119.34 (q, ^3^ *J*_CF_ = 6.5 Hz, C‐2′), 114.68 (C‐1′′), 97.97 (C‐1′′′), 80.93 (C‐2′′′), 58.18 (C‐7′), 54.97 (C‐2′′′′), 51.02 (C‐1′′′′), 43.48 (C‐3′′′′), 20.95 (CH_3_); ^19^F NMR (376 MHz, CDCl_3_): δ [ppm] = 60.36; MS (ESI^+^): *m/z* = 533.46 [M + H]^+^; HRMS (ESI): *m/z* calcd for C_29_H_28_F_3_N_6_O^+^: 533.2271 [M + H]^+^; found: 533.2244.

#### 4.2.9 3‐(Imidazo[1,2‐b]pyridazin‐3‐ylethynyl)‐2‐methyl‐N‐(4‐((4‐methylpiperazin‐1‐yl)methyl)‐3‐(trifluoromethyl)phenyl)benzamide (2)

Synthesized according to General Procedure D, Colorless powder; 212 mg, 0.398 mmol, 58% yield. *R*_f_ = 0.17 (CH_2_Cl_2_/MeOH 98:2); ^1^H NMR (500 MHz, MeOD‐d_4_): δ [ppm] = 8.70–8.66 (m, 1H, H‐6′′), 8.17–8.14 (m, 1H, H‐4′′), 8.14–8.13 (m, 1H, H‐2′′), 8.13–8.11 (m, 1H, H‐2′), 7.98–7.94 (m, 1H, H‐6′), 7.80–7.75 (m, 1H, H‐6), 7.74–7.70 (m, 1H, H‐5′), 7.55–7.51 (m, 1H, H‐4), 7.46–7.42 (m, 1H, H‐5′′), 7.41–7.36 (m, 1H, H‐5), 3.79 (s, 3H, H‐7′), 3.71–2.24 (m, 8H, H‐1′′′′/H‐2′′′′), 2.91 (s, 3H, H‐3′′′′), 2.69 (s, 3H, CH_3_); ^13^C NMR (126 MHz, MeOD‐d_4_): δ [ppm] = 170.73 (C = O), 146.56 (C‐6′′), 140.83 (C‐3′′), 139.67 (C‐2), 138.95 (C‐1), 138.71 (C‐1′), 136.73 (C‐2′′), 134.44 (C‐6), 132.94 (C‐5′), 132.84 (C‐3′′), 130.53 (q, ^2^ *J*_CF_ = 30.3 Hz, C‐3′), 128.74 (C‐4), 127.21 (C‐5), 126.74 (C‐4′), 126.05 (C‐4′′), 125.70 (q, ^1^ *J*_CF_ = 290.5 Hz, CF_3_), 124.89 (C‐3), 124.55 (C‐6′), 121.37 (C‐5′′), 118.70 (q, ^3^ *J*_CF_ = 6.5 Hz, C‐2′), 114.70 (C‐1′′), 98.20 (C‐1′′′), 81.00 (C‐2′′′), 58.18 (C‐7′), 55.01 (C‐2′′′′), 51.03 (C‐1′′′′), 43.49 (C‐3′′′′), 18.20 (CH_3_); ^19^F NMR (376 MHz, MeOD‐d_4_): δ [ppm] = −60.42; MS (ESI^+^): *m/z* = 533.46 [M + H]^+^; HRMS (ESI): *m/z* calcd for C_29_H_28_F_3_N_6_O^+^: 533.2271 [M + H]^+^; found: 533.2242.

#### 4.2.10 5‐(Imidazo[1,2‐b]pyridazin‐3‐ylethynyl)‐2‐methyl‐N‐(4‐((4‐methylpiperazin‐1‐yl)methyl)‐3‐(trifluoromethyl)phenyl)benzamide (3)

Synthesized according to General Procedure D, Colorless powder; 161 mg, 0.302 mmol, 44% yield. *R*_f_ = 0.17 (CH_2_Cl_2_/MeOH 98:2); ^1^H NMR (500 MHz, MeOD‐d_4_): δ [ppm] = 8.71–8.67 (m, 1H, H‐6′′), 8.18–8.15 (m, 1H, H‐4′′), 8.15–8.13 (m, 1H, H‐2′), 8.13–8.12 (m, 1H, H‐2′′), 7.98–7.93 (m, 1H, H‐6′), 7.79–7.76 (m, 1H, H‐5′), 7.76–7.74 (m, 1H, H‐2), 7.66–7.62 (m, 1H, H‐4), 7.48–7.44 (m, 1H, H‐5′′), 7.42–7.39 (m, 1H, H‐5), 3.79 (s, 2H, H‐7′), 3.66–2.25 (m, 8H, H‐1′′′′/H‐2′′′′), 2.91 (s, 3H, H‐3′′′′), 2.50 (s, 3H, CH_3_); ^13^C NMR (126 MHz, MeOD‐d_4_): δ [ppm] = 170.13 (C = O), 146.69 (C‐6′′), 140.53 (C‐3′′), 139.71 (C‐1′), 138.66 (C‐6), 138.31 (C‐1), 136.25 (C‐2′′), 133.99 (C‐4), 132.93 (C‐5′), 132.72 (C‐4′), 132.50 (C‐5), 131.11 (C‐2), 130.42 (q, ^2^ *J*_CF_ = 30.4 Hz, C‐3′), 125.91 (C‐4′′), 125.64 (q, ^1^ *J*_CF_ = 273.4 Hz, CF_3_), 124.59 (C‐6′), 121.69 (C‐5′′), 121.13 (C‐3), 118.75 (q, ^3^ *J*_CF_ = 6.0 Hz, C‐2′), 114.78 (C‐1′′), 99.12 (C‐1′′′), 76.28 (C‐2′′′), 58.17 (C‐7′), 54.97 (C‐2′′′′), 51.01 (C‐1′′′′), 43.48 (C‐3′′′′), 19.77 (CH_3_); ^19^F NMR (376 MHz, CDCl_3_): δ [ppm] = −60.40; MS (ESI^+^): *m/z* = 533.46 [M + H]^+^; HRMS (ESI): *m/z* calcd for C_29_H_28_F_3_N_6_O^+^: 533.2271 [M + H]^+^; found: 533.2244.

#### 4.2.11 3‐(Imidazo[1,2‐b]pyridazin‐3‐ylethynyl)‐2,4‐dimethyl‐N‐(4‐((4‐methylpiperazin‐1‐yl)methyl)‐3‐(trifluoromethyl)phenyl)benzamide (4)

Synthesized according to General Procedure D, Colorless powder; 262 mg, 0.481 mmol, 70% yield. *R*_f_ = 0.17 (CH_2_Cl_2_/MeOH 98:2); ^1^H NMR (500 MHz, MeOD‐d_4_): δ [ppm] = 8.73–8.70 (m, 1H, H‐6′′), 8.21–8.16 (m, 2H, H‐2′′/H‐4′′), 8.14–8.10 (m, 1H, H‐2′), 7.98–7.92 (m, 1H, H‐6′), 7.79–7.74 (m, 1H, H‐5′), 7.51–7.46 (m, 1H, H‐5′′), 7.45–7.41 (m, 1H, H‐6), 7.32–7.27 (m, 1H, H‐5), 3.79 (s, 2H, H‐7′), 3.67–2.30 (m, 8H, H‐1′′′′/H‐2′′′′), 2.91 (s, 3H, H‐3′′′′), 2.69 (s, 3H, CH_3_), 2.63 (s, 3H, CH_3_); ^13^C NMR (126 MHz, MeOD‐d_4_): δ [ppm] = 170.96 (C = O), 146.87 (C‐6′′), 143.86 (C‐4), 140.57 (C‐3′′), 139.78 (C‐1′), 138.99 (C‐2), 136.19 (C‐1), 135.60 (C‐2′′), 132.95 (C‐5′), 132.67 (C‐4′), 130.41 (q, ^2^ *J*_CF_ = 30.3 Hz, C‐3′), 128.44 (C‐6), 128.14 (C‐5), 125.74 (C‐4′′), 125.66 (q, ^1^ *J*_CF_ = 273.4 Hz, CF_3_), 124.60 (C‐3), 124.55 (C‐6′), 121.93 (C‐5′′), 118.70 (q, ^3^ *J*_CF_ = 5.9 Hz, C‐2′), 114.99 (C‐1′′), 97.22 (C‐1′′′), 85.27 (C‐2′′′), 58.18 (C‐7′), 54.98 (C‐2′′′′), 51.02 (C‐1′′′′), 43.48 (C‐3′′′′), 21.52 (CH_3_), 18.60 (CH_3_); ^19^F NMR (376 MHz, CDCl_3_): δ [ppm] = −60.40; MS (ESI^+^): *m/z* = 547.49 [M + H]^+^; HRMS (ESI): *m/z* calcd for C_30_H_30_F_3_N_6_O^+^: 547.2428 [M + H]^+^; found: 547.2404.

#### 4.2.12 (E)‐N‐(4‐(3‐Aminoprop‐1‐en‐1‐yl)‐3‐(trifluoromethyl)phenyl)‐3‐(imidazo[1,2‐b]pyridazin‐3‐ylethynyl)‐2‐methylbenzamide (5)

Synthesized according to General Procedure G, Colorless powder; 8.2 mg, 0.017 mmol, 87% yield. *R*_f_ = 0.18 (CH_2_Cl_2_/MeOH 95:5); ^1^H NMR (500 MHz, DMSO‐d_6_): δ [ppm] = 10.84 (s, 1H, NH(C = O)), 8.73–8.70 (m, 1H, H‐6′′), 8.28–8.24 (m, 2H, H‐2′/H‐4′′), 8.24–8.22 (m, 1H, H‐2′′), 8.11–7.97 (m, 3H, H‐6′/NH_2_), 7.83–7.79 (m, 1H, H‐5′), 7.75–7.70 (m, 1H, H‐6), 7.59–7.55 (m, 1H, H‐4), 7.46–7.41 (m, 1H, H‐5), 7.41–7.37 (m, 1H, H‐5′), 6.96 (d, ^3^ *J*_HH_ = 16.0 Hz, 1H, H‐1′′′′), 6.31 (dt, ^3^ *J*_HH_ = 15.8 Hz, ^3^ *J*_HH_ = 6.8 Hz, 1H, H‐2′′′′), 3.71 (d, ^3^ *J*_HH_ = 5.8 Hz, 2H, H‐3′′′′), 2.61 (s, 3H, CH_3_); ^13^C NMR (126 MHz, DMSO‐d_6_): δ [ppm] = 167.64 (C = O), 145.11 (C‐6′′), 139.64 (C‐3′′), 139.19 (C‐1′), 138.16 (C‐2′′), 137.64 (C‐2), 136.80 (C‐1), 132.83 (C‐6), 129.13 (C‐1′′′′), 129.08 (C‐4′), 128.37 (C‐5′), 127.93 (C‐4), 126.33 (q, ^2^ *J*_CF_ = 30.0 Hz, C‐3′), 126.25 (C‐5), 126.10 (C‐4′′), 125.73 (C‐2′′′′), 124.05 (q, ^1^ *J*_CF_ = 273.7 Hz, CF_3_), 123.21 (C‐6′), 122.95 (C‐3), 119.13 (C‐5′′), 116.40 (q, ^3^ *J*_CF_ = 6.1 Hz, C‐2′), 111.73 (C‐1′′), 96.62 (C‐1′′′), 81.16 (C‐2′′′), 40.58 (C‐3′′′′), 17.76 (CH_3_); ^19^F NMR (376 MHz, DMSO‐d_6_): δ [ppm] = −58.10; MS (ESI^+^): *m/z* = 476.42 [M + H]^+^; HRMS (ESI): *m/z* calcd for C_26_H_21_F_3_N_5_O^+^: 476.1693 [M + H]^+^; found: 476.1678.

#### 4.2.13 (E)‐N‐(4‐(3‐(1,3‐Dioxoisoindolin‐2‐yl)prop‐1‐en‐1‐yl)‐3‐(trifluoromethyl)phenyl)‐3‐(imidazo[1,2‐b]pyridazin‐3‐ylethynyl)‐2‐methylbenzamide (5a)

Synthesized according to General Procedure E, Colorless powder; 111 mg, 0.183 mmol, 83% yield. *R*_f_ = 0.27 (CH_2_Cl_2_/MeOH 99:1); ^1^H NMR (400 MHz, CDCl_3_): δ [ppm] = 8.48–8.44 (m, 1H, H‐6′′), 8.10–8.05 (m, 1H, H‐2′), 8.01–7.98 (m, 1H, H‐6′), 7.96–7.92 (m, 1H, H‐4′′), 7.91–7.88 (m, 1H, H‐2′′), 7.88–7.84 (m, 2H, H‐2′′′′′), 7.76–7.69 (m, 2H, H‐3′′′′′), 7.67–7.62 (m, 1H, H‐5′), 7.61–7.56 (m, 1H, H‐6), 7.44–7.39 (m, 1H, H‐4), 7.25–7.20 (m, 1H, H‐5), 7.14–7.09 (m, 1H, H‐5′′), 6.98 (d, ^3^ *J*_HH_ = 15.6 Hz, 1H, H‐1′′′′), 6.20 (dt, ^3^ *J*_HH_ = 15.6 Hz, ^3^ *J*_HH_ = 6.5 Hz, 1H, H‐2′′′′), 4.48 (dd, ^3^ *J*_HH_ = 6.5 Hz, ^4^ *J*_HH_ = 1.4 Hz, 2H, H‐3′′′′), 2.68 (s, 3H, CH_3_); ^13^C NMR (126 MHz, CDCl_3_): δ [ppm] = 168.04 (N(C = O)_2_), 167.77 (C = O), 144.05 (C6′′), 140.60 (C‐2), 138.49 (C‐3′′), 138.47 (C‐1′), 135.95 (C‐4), 134.21 (C‐3′′′′′), 133.00 (C‐1′′′′′), 132.26 (C‐2′′′′), 132.19 (C‐1′′′′), 132.11 (C‐4′), 132.09 (C‐5′), 131.50 (C‐3′), 128.70 (C‐6), 128.60 (C‐5), 126.68 (C‐4′′), 126.11 (C‐6′), 124.40 (q, ^1^ *J*_CF_ = 273.5 Hz, CF_3_), 123.54 (C‐2′′′′′),119.81 (C‐2′′), 117.84 (C‐5′′), 96.66 (C‐1′′′), 84.49 (C‐2′′′), 40.00 (C‐3′′′′), 18.35 (CH_3_); ^19^F NMR (376 MHz, CDCl_3_): δ [ppm] = −59.57; MS (ESI^+^): *m/z* = 606.39 [M + H]^+^; HRMS (ESI): *m/z* calcd for C_34_H_23_F_3_N_5_O_3_^+^: 606.1748 [M + H]^+^; found: 606.1724.

#### 4.2.14 (E)‐N‐(4‐(3‐Aminoprop‐1‐en‐1‐yl)‐3‐(trifluoromethyl)phenyl)‐3‐(imidazo[1,2‐b]pyridazin‐3‐ylethynyl)‐4‐methylbenzamide (6)

Synthesized according to General Procedure G, Colorless powder; 8.6 mg, 0.018 mmol, 92% yield. *R*_f_ = 0.18 (CH_2_Cl_2_/MeOH 95:5); ^1^H NMR (500 MHz, DMSO‐d_6_): δ [ppm] = 10.68 (s, 1H, NH(C = O)), 8.75–8.71 (m, 1H, H‐6′′), 8.31–8.25 (m, 2H, H‐2′/H‐4′′), 8.25–8.23 (m, 1H, H‐2′′), 8.23–8.21 (m, 1H, H‐2), 8.19–8.14 (m, 1H, H‐6′), 8.10–7.99 (m, 2H, NH_2_), 7.98–7.94 (m, 1H, H‐6), 7.84–7.78 (m, 1H, H‐5′), 7.59–7.54 (m, 1H, H‐5), 7.43–7.37 (m, 1H, H‐5′′), 6.96 (d, ^3^ *J*_HH_ = 15.7 Hz, 1H, H‐1′′′′), 6.32 (dt, ^3^ *J*_HH_ = 15.7 Hz, ^3^ *J*_HH_ = 6.8 Hz, 1H, H‐2′′′′), 3.70 (d, ^3^ *J*_HH_ = 5.7 Hz, 2H, H‐3′′′′), 2.62 (s, 3H, CH_3_); ^13^C NMR (126 MHz, DMSO‐d_6_): δ [ppm] = 164.73 (C = O), 145.11 (C‐6′′), 143.68 (C‐4), 139.68 (C‐3′′), 139.29 (C‐1′), 138.25 (C‐2′′), 132.10 (C‐1), 130.20 (C‐2), 130.15 (C‐5), 129.10 (C‐4′), 129.02 (C‐1′′′′), 128.55 (C‐6), 128.22 (C‐5′), 126.19 (q, ^2^ *J*CF = 29.4 Hz, C‐3′), 126.13 (C‐4′′), 125.68 (C‐2′′′′), 124.07 (q, ^1^ *J*_CF_ = 277.0 Hz, CF_3_), 123.76 (C‐6′), 121.82 (C‐3), 119.16 (C‐5′′), 117.11 (q, ^3^ *J*_CF_ = 6.1 Hz, C‐2′), 111.69 (C‐1′′), 96.38 (C‐1′′′), 81.20 (C‐2′′′), 40.58 (C‐3′′′′), 20.42 (CH_3_); ^19^F NMR (376 MHz, DMSO‐d_6_): δ [ppm] = −58.03; MS (ESI^+^): *m/z* = 476.36 [M + H]^+^; HRMS (ESI): *m/z* calcd for C_26_H_21_F_3_N_5_O^+^: 476.1693 [M + H]^+^; found: 476.1681.

#### 4.2.15 (E)‐N‐(4‐(3‐(1,3‐Dioxoisoindolin‐2‐yl)prop‐1‐en‐1‐yl)‐3‐(trifluoromethyl)phenyl)‐3‐(imidazo[1,2‐b]pyridazin‐3‐ylethynyl)‐4‐methylbenzamide (6a)

Synthesized according to General Procedure E, Colorless powder; 102 mg, 0.168 mmol, 76% yield. *R*_f_ = 0.27 (CH_2_Cl_2_/MeOH 99:1); ^1^H NMR (500 MHz, DMSO‐d_6_): δ [ppm] = 10.60 (s, 1H, NH), 8.77–8.70 (m, 1H, H‐6′′), 8.56–8.33 (m, 1H, H‐2′′), 8.32–8.25 (m, 1H, H‐4′′), 8.24–8.22 (m, 1H, H‐2′), 8.22–8.18 (m, 1H, H‐2), 8.10–8.03 (m, 1H, H‐6′), 7.96–7.92 (m, 1H, H‐6), 7.94–7.90 (m, 2H, H‐2′′′′′), 7.89–7.84 (m, 2H, H‐3′′′′′), 7.84–7.80 (m, 1H, H‐5′), 7.57–7.52 (m, 1H, H‐5), 7.44–7.36 (m, 1H, H‐5′′), 6.73 (d, ^3^ *J*_HH_ = 15.6 Hz, 1H, H‐1′′′′), 6.39 (dt, ^3^ *J*_HH_ = 15.6 Hz, ^3^ *J*_HH_ = 5.6 Hz, 1H, H‐2′′′′), 4.41 (d, ^3^ *J*_HH_ = 5.5 Hz, 2H, H‐3′′′′), 2.60 (s, 3H, CH_3_); ^13^C NMR (126 MHz, DMSO‐d_6_): δ [ppm] = 167.47 (N(C = O)_2_), 164.60 (C = O), 145.08 (C‐6′′), 143.56 (C‐4), 138.80 (C‐1′), 134.54 (C‐3′′′′′), 132.11 (C‐2′′), 131.60 (C‐1′′′′′), 130.16 (C‐2), 130.09 (C‐5), 129.61 (C‐1), 129.45 (C‐3′′), 128.50 (C‐6), 128.16 (C‐5′), 127.75 (C‐2′′′′), 126.27 (C‐4′′), 126.09 (C‐1′′′′), 125.83 (q, ^2^ *J*_CF_ = 29.1 Hz, C‐3′), 125.19 (C‐4′), 124.34 (q, ^1^ *J*_CF_ = 273.5 Hz, CF_3_), 123.56 (C‐6′), 123.15 (C‐2′′′′′), 123.04 (C‐2′), 123.01 (C‐1), 119.02 (C‐5′′), 116.85 (q, ^3^ *J*_CF_ = 5.7 Hz, C‐2′), 96.44 (C‐1′′′), 81.42 (C‐2′′′), 45.70 (C‐3′′′′), 20.38 (CH_3_); ^19^F NMR (376 MHz, DMSO‐d_6_): δ [ppm] = −58.27; MS (ESI): *m/z* = 604.38 [M‐H]^‐^; HRMS (ESI): *m/z* calcd for C_34_H_23_F_3_N_5_O_3_^+^: 606.1748 [M + H]^+^; found: 606.1722.

#### 4.2.16 (E)‐N‐(4‐(3‐Aminoprop‐1‐en‐1‐yl)‐3‐(trifluoromethyl)phenyl)‐3‐(imidazo[1,2‐b]pyridazin‐3‐ylethynyl)‐6‐methylbenzamide (7)

Synthesized according to General Procedure G, Colorless powder; 7.2 mg, 0.015 mmol, 76% yield. *R*_f_ = 0.18 (CH_2_Cl_2_/MeOH 95:5); ^1^H NMR (500 MHz, DMSO‐d_6_): δ [ppm] = 10.79 (s, 1H, NH(C = O)), 8.71–8.68 (m, 1H, H‐6′′), 8.30–8.22 (m, 2H, H‐2′/4′′), 8.21–8.19 (m, 1H, H‐2′′), 8.08–7.91 (m, 3H, H‐6′/NH_2_), 7.83–7.78 (m, 1H, H‐5′), 7.77–7.75 (m, 1H, H‐2), 7.69–7.63 (m, 1H, H‐5), 7.46–7.42 (m, 1H, H‐4), 7.41–7.36 (m, 1H, H‐5′′), 6.96 (d, ^3^ *J*_HH_ = 15.5 Hz, 1H, H‐1′′′′), 6.31 (dt, ^3^ *J*_HH_ = 15.6 Hz, ^3^ *J*_HH_ = 6.7 Hz, 1H, H‐2′′′′), 3.71 (d, ^3^ *J*_HH_ = 5.7 Hz, 2H, H‐3′′′′), 2.45 (s, 3H, CH_3_); ^13^C NMR (126 MHz, DMSO‐d_6_): δ [ppm] = 166.99 (C = O), 144.96 (C‐6′′), 139.54 (C‐3′′), 139.24 (C‐1′), 138.26 (C‐2′′), 137.28 (C‐6), 136.70 (C‐1), 132.51 (C‐5), 131.53 (C‐4), 130.03 (C‐2), 129.09 (C‐1′′′′), 129.04 (C‐4′), 128.30 (C‐5′), 126.26 (q, ^2^ *J*_CF_ = 29.4 Hz, C‐3′), 126.10 (C‐4′′), 125.68 (C‐2′′′′), 124.08 (q, ^1^ *J*_CF_ = 275.8 Hz, CF_3_), 123.32 (C‐6′), 122.64 (C‐3), 119.07 (C‐5′′), 116.58 (q, ^3^ *J*_CF_ = 6.4 Hz, C‐2′), 111.69 (C‐1′′), 97.42 (C‐1′′′), 76.97 (C‐2′′′), 40.58 (C‐3′′′′), 19.46 (CH_3_); ^19^F NMR (376 MHz, DMSO‐d_6_): δ [ppm] = −58.08; MS (ESI^+^): *m/z* = 476.40 [M + H]^+^; HRMS (ESI): *m/z* calcd for C_26_H_21_F_3_N_5_O^+^: 476.1693 [M + H]^+^; found: 476.1680.

#### 4.2.17 (E)‐N‐(4‐(3‐(1,3‐Dioxoisoindolin‐2‐yl)prop‐1‐en‐1‐yl)‐3‐(trifluoromethyl)phenyl)‐5‐(imidazo[1,2‐b]pyridazin‐3‐ylethynyl)‐2‐methylbenzamide (7a)

Synthesized according to General Procedure E, Colorless powder; 115 mg, 0.190 mmol, 86% yield. *R*_f_ = 0.27 (CH_2_Cl_2_/MeOH 99:1); ^1^H NMR (400 MHz, CDCl_3_): δ [ppm] = 8.48–8.45 (m, 1H, H‐6′′), 8.06–8.03 (m, 1H, H‐2′), 7.96–7.94 (m, 2H, H‐6′/H‐4′′), 7.91–7.89 (m, 2H, H‐2/H‐2′′), 7.88–7.86 (m, 2H, H‐2′′′′′), 7.75–7.70 (m, 3H, H‐6/H‐3′′′′′), 7.62–7.57 (m, 1H, H‐5′), 7.32–7.27 (m, 1H, H‐5), 7.16–7.09 (m, 1H, H‐5′′), 6.99 (d, ^3^ *J*_HH_ = 15.7 Hz, 1H, H‐1′′′′), 6.22 (dt, ^3^ *J*_HH_ = 15.7 Hz, ^3^ *J*_HH_ = 6.4 Hz, 1H, H‐2′′′′), 4.49 (d, ^3^ *J*_HH_ = 6.4 Hz, 2H, H‐3′′′′), 2.54 (s, 3H, CH_3_); ^13^C NMR (100 MHz, CDCl_3_): δ [ppm] = 168.54 (C = O), 168.03 (N(C=O)_2_), 146.03 (C‐4), 142.97 (C‐6′′), 139.93 (C‐1′), 138.34 (C‐3′′), 137.69 (C‐2), 135.96 (C‐1), 134.20 (C‐3′′′′′), 132.23 (C‐1′′′′′), 132.11 (C‐2′′′′), 131.70 (C‐1′′′′), 131.38 (C‐4′), 129.13 (C‐5), 128.89 (C‐5′), 128.70 (C‐6), 128.53 (C‐3′), 126.63 (C‐4′′), 124.41 (q, ^1^ *J*_CF_ = 274.0 Hz, CF_3_), 123.52 (C‐2′′′′′), 123.39 (C‐6′), 121.77 (C‐4′′), 117.18 (C‐2′), 117.11 (C‐5′′), 107.11 (C‐1′′), 95.14 (C‐1′′′), 84.25 (C‐2′′′), 39.81 (C‐3′′′′), 20.09 (CH_3_); ^19^F NMR (376 MHz, CDCl_3_): δ [ppm] = −59.60; MS (ESI^+^): *m/z* = 606.43 [M + H]^+^; HRMS (ESI): *m/z* calcd for C_34_H_23_F_3_N_5_O_3_^+^: 606.1748 [M + H]^+^; found: 606.1721.

#### 4.2.18 (E)‐N‐(4‐(3‐Aminoprop‐1‐en‐1‐yl)‐3‐(trifluoromethyl)phenyl)‐3‐(imidazo[1,2‐b]pyridazin‐3‐ylethynyl)−2,4‐dimethylbenzamide (8)

Synthesized according to General Procedure G, Colorless powder; 8.7 mg, 0.018 mmol, 89% yield. *R*_f_ = 0.18 (CH_2_Cl_2_/MeOH 95:5); ^1^H NMR (500 MHz, DMSO‐d_6_): δ [ppm] = 10.76 (s, 1H, NH(C = O)), 8.73–8.70 (m, 1H, H‐6′′), 8.26–8.24 (m, 2H, H‐2′/H‐4′′), 8.24–8.23 (m, 1H, H‐2′′), 8.12–7.91 (m, 3H, H‐6′/NH_2_), 7.82–7.78 (m, 1H, H‐5′), 7.48–7.45 (m, 1H, H‐6), 7.41–7.37 (m, 1H, H‐5′′), 7.36–7.32 (m, 1H, H‐5), 6.96 (d, ^3^ *J*_HH_ = 15.5 Hz, 1H, H‐1′′′′), 6.31 (dt, ^3^ *J*_HH_ = 15.6 Hz, ^3^ *J*_HH_ = 6.7 Hz, 1H, H‐2′′′′), 3.71 (d, ^3^ *J*_HH_ = 5.7 Hz, 2H, H‐3′′′′), 2.61 (s, 3H, CH_3_), 2.59 (s, 3H, CH_3_); ^13^C NMR (126 MHz, DMSO‐d_6_): δ [ppm] = 167.79 (C = O), 145.14 (C‐6′′), 141.57 (C‐4), 139.65 (C‐3′′′), 139.29 (C‐1′), 138.01 (C‐2′′), 136.97 (C‐2), 134.89 (C‐1), 129.11 (C‐1′′′′), 129.00 (C‐4′), 128.33 (C‐5′), 127.44 (C‐6), 126.91 (C‐5), 126.31 (q, ^2^ *J*_CF_ = 29.4 Hz, C‐3′), 126.09 (C‐4′′), 125.65 (C‐2′′′′), 124.06 (q, ^1^ *J*_CF_ = 273.9 Hz, CF_3_), 123.17 (C‐6′), 122.98 (C‐3), 119.05 (C‐5′′), 116.37 (q, ^3^ *J*_CF_ = 6.4 Hz, C‐2′), 111.90 (C‐1′′), 95.43 (C‐1′′′), 85.46 (C‐2′′′), 40.58 (C‐3′′′′), 20.99 (CH_3_), 18.17 (CH_3_); ^19^F NMR (376 MHz, DMSO‐d_6_): δ [ppm] = −58.08; MS (ESI^+^): *m/z* = 490.40 [M + H]^+^; HRMS (ESI): *m/z* calcd for C_27_H_23_F_3_N_5_O^+^: 490.1849 [M + H]^+^; found: 490.1834.

#### 4.2.19 (E)‐N‐(4‐(3‐(1,3‐Dioxoisoindolin‐2‐yl)prop‐1‐en‐1‐yl)‐3‐(trifluoromethyl)phenyl)‐3‐(imidazo[1,2‐b]pyridazin‐3‐ylethynyl)−2,4‐dimethylbenzamide (8a)

Synthesized according to General Procedure E, Colorless powder; 102 mg, 0.166 mmol, 75% yield. *R*_f_ = 0.27 (CH_2_Cl_2_/MeOH 99:1); ^1^H NMR (500 MHz, CDCl_3_): δ [ppm] = 8.51–8.47 (m, 1H, H‐6′′), 8.05–7.99 (m, 3H, H‐2′/H‐2′′/H‐4′′), 7.88–7.86 (m, 3H, H‐6′/H‐2′′′′′), 7.74–7.72 (m, 2H, H‐3′′′′′), 7.61–7.57 (m, 1H, H‐5′), 7.36–7.33 (m, 1H, H‐6), 7.18–7.12 (m, 2H, H‐5/H‐5′′), 6.98 (d, ^3^ *J*_HH_ = 15.5 Hz, 1H, H‐1′′′′), 6.21 (dt, ^3^ *J*_HH_ = 15.5 Hz, ^3^ *J*_HH_ = 6.4 Hz, 1H, H‐2′′′′), 4.48 (d, ^3^ *J*_HH_ = 6.5 Hz, 1H, H‐3′′′′), 2.70 (s, 3H, CH_3_), 2.59 (s, 3H, CH_3_); ^13^C NMR (126 MHz, CDCl_3_): δ [ppm] = 168.55 (C = O), 168.06 (N(C = O)_2_), 144.95 (C‐4), 144.20 (C‐6′′), 143.14 (C‐2), 142.21 (C‐1′), 138.64 (C‐3′′), 137.75 (C‐1), 134.22 (C‐3′′′′′), 134.18 (C‐2′′), 132.26 (C‐1′′′′′), 132.10 (C‐2′′′′), 131.89 (C‐1′′′′), 131.37 (C‐4′), 128.60 (C‐6), 128.48 (C‐5′), 128.13 (C‐3′), 127.07 (C‐5), 126.88 (C‐6′), 125.85 (C‐4′′), 124.41 (C‐3), 124.33 (q, ^1^ *J*_CF_ = 274.0 Hz, CF_3_), 123.55 (C‐2′′′′′), 122.87 (C‐5′′), 117.08 (C‐2′), 106.62 (C‐1′′), 96.07 (C‐1′′′), 85.27 (C‐2′′′), 39.82 (C‐3′′′′), 21.65 (CH_3_), 18.67 (CH_3_); ^19^F NMR (376 MHz, CDCl_3_): δ [ppm] = −59.57; MS (ESI^+^): *m/z* = 620.41 [M + H]^+^; HRMS (ESI): *m/z* calcd for C_35_H_25_F_3_N_5_O_3_^+^: 620.1904 [M + H]^+^; found: 620.1884.

#### 4.2.20 N‐(4‐(3‐Aminopropyl)‐3‐(trifluoromethyl)phenyl)‐3‐(imidazo[1,2‐b]pyridazin‐3‐ylethynyl)‐2‐methylbenzamide (9)

Synthesized according to General Procedure G, Colorless powder; 8.7 mg, 0.018 mmol, 91% yield. *R*_f_ = 0.19 (CH_2_Cl_2_/MeOH 95:5); ^1^H NMR (500 MHz, DMSO‐d_6_): δ [ppm] = 10.70 (s, 1H, NH(C = O)), 8.73–8.70 (m, 1H, H‐6′′), 8.28–8.24 (m, 1H, H‐4′′), 8.24–8.22 (m, 1H, H‐2′′), 8.20–8.17 (m, 1H, H‐2′), 7.98–7.93 (m, 1H, H‐6′), 7.77–7.64 (m, 3H, H‐6/NH_2_), 7.57–7.53 (m, 1H, H‐4), 7.51–7.46 (m, 1H, H‐5′), 7.45–7.41 (m, 1H, H‐5), 7.41–7.36 (m, 1H, H‐5′′), 2.92–2.82 (m, 2H, H‐3′′′′), 2.77 (t, ^3^ *J*_HH_ = 7.8 Hz, 2H, H‐1′′′′), 2.60 (s, 3H, CH_3_), 1.84 (tt, ^3^ *J*_HH_ = 7.8 Hz, ^3^ *J*_HH_ = 7.7 Hz, 2H, H‐2′′′′); ^13^C NMR (126 MHz, DMSO‐d_6_): δ [ppm] = 167.50 (C = O), 145.08 (C‐6′′), 139.64 (C‐3′′), 138.20 (C‐2′′), 137.78 (C‐2), 137.65 (C‐1′), 136.70 (C‐1), 134.22 (C‐4′), 132.73 (C‐6), 131.91 (C‐5′), 127.86 (C‐4), 127.18 (q, ^2^ *J*_CF_ = 28.7 Hz, C‐3′), 126.22 (C‐4′′), 126.11 (C‐5), 124.37 (q, ^1^ *J*_CF_ = 273.0 Hz, CF_3_), 123.29 (C‐6′), 122.91 (C‐3), 119.09 (C‐5′′), 116.67 (q, ^3^ *J*_CF_ = 5.5 Hz, C‐2′), 111.71 (C‐1′′), 96.62 (C‐1′′′), 81.13 (C‐2′′′), 38.60 (C‐3′′′′), 29.06 (C‐2′′′′), 28.37 (C‐1′′′′), 17.74 (CH_3_); ^19^F NMR (376 MHz, DMSO‐d_6_): δ [ppm] = −58.52; MS (ESI^+^): *m/z* = 478.40 [M + H]^+^; HRMS (ESI): *m/z* calcd for C_26_H_23_F_3_N_5_O^+^: 478.1849 [M + H]^+^; found: 478.1836.

#### 4.2.21 N‐(4‐(3‐(1,3‐Dioxoisoindolin‐2‐yl)propyl)‐3‐(trifluoromethyl)phenyl)‐3‐(imidazo[1,2‐b]pyridazin‐3‐ylethynyl)‐2‐methylbenzamide (9a)

Synthesized according to General Procedure F, Colorless powder; 102 mg, 0.168 mmol, 76% yield. *R*_f_ = 0.26 (CH_2_Cl_2_/MeOH 99:1); ^1^H NMR (500 MHz, CDCl_3_): δ [ppm] = 8.40–8.27 (m, 1H, H‐6′′), 7.92–7.88 (m, 1H, H‐2′), 7.87–7.82 (m, 2H, H‐2′′′′′), 7.74–7.69 (m, 2H, H‐3′′′′′), 7.65–7.63 (m, 1H, H‐2′), 7.61–7.59 (m, 2H, H‐6′/H‐4′′), 7.53–7.50 (m, 1H, H‐2′′), 7.48–7.45 (m, 2H, H‐6/H‐5′), 7.38–7.33 (m, 1H, H‐5), 7.22–7.15 (m, 1H, H‐5′′), 3.77 (t, ^3^ *J*_HH_ = 7.1 Hz, 2H, H‐3′′′′), 2.83 (t, ^3^ *J*_HH_ = 7.7 Hz, 2H, H‐1′′′′), 2.68 (s, 3H, CH_3_), 2.01 (tt, ^3^ *J*_HH_ = 7.7 Hz, ^3^ *J*_HH_ = 7.1 Hz, 2H, H‐2′′′′); ^13^C NMR (126 MHz, CDCl_3_): δ [ppm] = 168.52 (N(C = O)_2_), 167.94 (C = O), 144.30 (C‐6′′), 138.53 (C‐2), 137.21 (C‐1), 136.54 (C‐1′), 135.85 (C‐2′′), 134.11 (C‐3′′′′′), 132.91 (C‐5′), 132.23 (C‐4), 132.21 (C‐1′′′′′), 132.16 (C‐3′′), 132.12 (C‐3′), 132.10 (C‐6), 131.81 (C‐5), 129.27 (C‐4′), 129.03 (C‐4′′), 128.70 (C‐1′), 128.60 (C‐6′), 127.31 (C‐3), 125.88 (CF_3_), 123.39 (C‐2′′′′′), 123.34 (C‐5′′), 117.81 (C‐2′), 94.17 (C‐1′′′), 86.01 (C‐2′′′), 37.84 (C‐3′′′′), 30.39 (C‐1′′′′), 29.60 (C‐2′′′′), 18.28 (CH_3_); ^19^F NMR (376 MHz, CDCl_3_): δ [ppm] = −59.90; MS (ESI^‐^): *m/z* = 606.39 [M‐H]; HRMS (ESI): *m/z* calcd for C_34_H_23_F_3_N_5_O_3_^‐^: 606.1758 [M‐H]^‐^; found: 606.1761.

#### 4.2.22 N‐(4‐(3‐Aminopropyl)‐3‐(trifluoromethyl)phenyl)‐3‐(imidazo[1,2‐b]pyridazin‐3‐ylethynyl)‐4‐methylbenzamide (10)

Synthesized according to General Procedure G, Colorless powder; 8.9 mg, 0.019 mmol, 93% yield. *R*_f_ = 0.19 (CH_2_Cl_2_/MeOH 95:5); ^1^H NMR (500 MHz, DMSO‐d_6_): δ [ppm] = 10.57 (s, 1H, NH(C = O)), 8.74–8.70 (m, 1H, H‐6′′), 8.29–8.25 (m, 1H, H‐4′′), 8.25–8.23 (m, 1H, H‐2′′), 8.22–8.20 (m, 1H, H‐2), 8.20–8.17 (m, 1H, H‐2′), 8.10–8.06 (m, 1H, H‐6′), 7.97–7.93 (m, 1H, H‐6), 7.75 (bs, 2H, NH_2_), 7.58–7.54 (m, 1H, H‐5), 7.51–7.46 (m, 1H, H‐5′), 7.43–7.38 (m, 1H, H‐5′′), 2.92–2.82 (m, 2H, H‐3′′′′), 2.78 (t, ^3^ *J*_HH_ = 7.8 Hz, 2H, H‐1′′′′), 2.61 (s, 3H, CH_3_), 1.84 (tt, ^3^ *J*_HH_ = 8.0 Hz, ^3^ *J*_HH_ = 7.7 Hz, 2H, H‐2′′′′); ^13^C NMR (126 MHz, DMSO‐d_6_): δ [ppm] = 164.59 (C = O), 145.10 (C‐6′′), 143.56 (C‐4), 139.67 (C‐3′′), 138.23 (C‐2′′), 137.70 (C‐1′), 134.15 (C‐4′), 132.18 (C‐1), 131.74 (C‐5′), 130.13 (C‐2/C‐5), 128.49 (C‐6), 127.07 (q, ^2^ *J*_CF_ = 29.1 Hz, C‐3′), 126.13 (C‐4′′), 124.40 (q, ^1^ *J*_CF_ = 270.9 Hz, CF_3_), 123.91 (C‐6′), 121.78 (C‐3), 119.14 (C‐5′′), 117.44 (q, ^3^ *J*_CF_ = 6.1 Hz, C‐2′), 111.69 (C‐1′′), 96.40 (C‐1′′′), 81.16 (C‐2′′′), 38.61 (C‐3′′′′), 29.03 (C‐2′′′′), 28.35 (C‐1′′′′), 20.40 (CH_3_); ^19^F NMR (376 MHz, DMSO‐d_6_): δ [ppm] = −58.48; MS (ESI^+^): *m/z* = 478.36 [M + H]^+^; HRMS (ESI): *m/z* calcd for C_26_H_23_F_3_N_5_O^+^: 478.1849 [M + H]^+^; found: 478.1830.

#### 4.2.23 N‐(4‐(3‐(1,3‐Dioxoisoindolin‐2‐yl)propyl)‐3‐(trifluoromethyl)phenyl)‐3‐(imidazo[1,2‐b]pyridazin‐3‐ylethynyl)‐4‐methylbenzamide (10a)

Synthesized according to General Procedure F, Colorless powder; 91.2 mg, 0.150 mmol, 68% yield. *R*_f_ = 0.26 (CH_2_Cl_2_/MeOH 99:1); ^1^H NMR (300 MHz, CDCl_3_): δ [ppm] = 8.65–8.55 (m, 1H, H‐6′′), 8.13–8.04 (m, 1H, H‐2), 8.03–7.95 (m, 1H, H‐2′), 7.95–7.89 (m, 1H, H‐6), 7.88–7.79 (m, 2H, H‐2′′′′′), 7.75–7.68 (m, 2H, H‐3′′′′′), 7.67–7.57 (m, 2H, H‐6′), 7.56–7.49 (m, 1H, H‐5′), 7.48–7.39 (m, 2H, H‐2′′/4′′), 7.36–7.27 (m, 2H, H‐5/5′′), 3.76 (t, ^3^ *J*_HH_ = 7.2 Hz, 2H, H‐3′′′′), 2.80 (t, ^3^ *J*_HH_ = 7.7 Hz, 2H, H‐1′′′′), 2.58 (s, 3H, CH_3_), 1.99 (tt, ^3^ *J*_HH_ = 7.7 Hz, ^3^ *J*_HH_ = 7.6 Hz, 2H, H‐2′′′′); MS (ESI^‐^): *m/z* = 606.37 [M‐H]; HRMS (ESI): *m/z* calcd for C_34_H_23_F_3_N_5_O_3_^‐^: 606.1758 [M‐H]^‐^; found: 606.1761.

#### 4.2.24 N‐(4‐(3‐Aminopropyl)‐3‐(trifluoromethyl)phenyl)‐3‐(imidazo[1,2‐b]pyridazin‐3‐ylethynyl)‐6‐methylbenzamide (11)

Synthesized according to General Procedure G, Colorless powder; 8.1 mg, 0.017 mmol, 85% yield. *R*_f_ = 0.19 (CH_2_Cl_2_/MeOH 95:5); ^1^H NMR (500 MHz, DMSO‐d_6_): δ [ppm] = 10.68 (s, 1H, NH(C = O)), 8.72–8.67 (m, 1H, H‐6′′), 8.28–8.23 (m, 1H, H‐4′′), 8.21–8.19 (m, 1H, H‐2′′), 8.19–8.16 (m, 1H, H‐2′), 8.00–7.94 (m, 1H, H‐6′), 7.76 (bs, 2H, NH_2_), 7.74–7.71 (m, 1H, H‐2), 7.67–7.63 (m, 1H, H‐5), 7.50–7.46 (m, 1H, H‐5′), 7.45–7.41 (m, 1H, H‐6), 7.41–7.36 (m, 1H, H‐5′′), 2.92–2.82 (m, 2H, H‐3′′′′), 2.77 (t, ^3^ *J*_HH_ = 7.8 Hz, 2H, H‐1′′′′), 2.44 (s, 3H, CH_3_), 1.84 (tt, ^3^ *J*_HH_ = 8.0 Hz, ^3^ *J*_HH_ = 7.8 Hz, 2H, H‐2′′′′); ^13^C NMR (126 MHz, DMSO‐d_6_): δ [ppm] = 166.87 (C = O), 144.95 (C‐6′′), 139.53 (C‐3′′), 138.24 (C‐2′′), 137.69 (C‐1′), 137.20 (C‐6), 136.87 (C‐1), 134.21 (C‐4′), 132.40 (C‐5), 131.84 (C‐5′), 131.49 (C‐6), 129.96 (C‐2), 127.14 (q, ^2^ *J*_CF_ = 29.1 Hz, C‐3′), 126.09 (C‐4′′), 124.40 (q, ^1^ *J*_CF_ = 273.9 Hz, CF_3_), 123.43 (C‐6′), 119.09 (C‐3), 119.06 (C‐5′′), 116.86 (q, ^3^ *J*_CF_ = 6.1 Hz, C‐2′), 111.69 (C‐1′′), 97.44 (C‐1′′′), 76.93 (C‐2′′′), 38.60 (C‐3′′′′), 29.07 (C‐2′′′′), 28.38 (C‐1′′′′), 19.44 (CH_3_); ^19^F NMR (376 MHz, DMSO‐d_6_): δ [ppm] = −58.51; MS (ESI^+^): *m/z* = 478.39 [M + H]^+^; HRMS (ESI): *m/z* calcd for C_26_H_23_F_3_N_5_O^+^: 478.1849 [M + H]^+^; found: 478.1836.

#### 4.2.25 N‐(4‐(3‐(1,3‐Dioxoisoindolin‐2‐yl)propyl)‐3‐(trifluoromethyl)phenyl)‐3‐(imidazo[1,2‐b]pyridazin‐3‐ylethynyl)‐6‐methylbenzamide (11a)

Synthesized according to General Procedure F, Colorless powder; 85.8 mg, 0.141 mmol, 64% yield. *R*_f_ = 0.26 (CH_2_Cl_2_/MeOH 99:1); ^1^H NMR (500 MHz, CDCl_3_): δ [ppm] = 8.69–8.45 (m, 1H, H‐6′′), 8.08–7.92 (m, 3H, H‐2′/H‐6′/H‐4′′), 7.88–7.82 (m, 2H, H‐2′′′′′), 7.77–7.68 (m, 2H, H‐3′′′′′), 7.66–7.60 (m, 2H, H‐2/H‐2′′), 7.57–7.50 (m, 1H, H‐5′), 7.49–7.40 (m, 2H, H‐4/5), 7.38–7.30 (m, 1H, H‐5′′), 3.77 (t, ^3^ *J*_HH_ = 7.0 Hz, 2H, H‐3′′′′), 2.82 (t, ^3^ *J*_HH_ = 7.4 Hz, 2H, H‐1′′′′), 2.55 (s, 3H, CH_3_), 2.00 (tt, ^3^ *J*_HH_ = 7.4 Hz, ^3^ *J*_HH_ = 7.1 Hz, 2H, H‐2′′′′); ^13^C NMR (126 MHz, CDCl_3_): δ [ppm] = 168.51 (N(C=O)_2_), 162.64 (C=O), 146.49 (C‐6′′), 139.08 (C‐1′), 136.70 (C‐1), 136.47 (C‐2′′), 135.91 (C‐6), 134.91 (C‐4), 134.11 (C‐3′′′′′), 132.19 (C‐1′′′′′), 132.15 (C‐3′′), 131.76 (C‐4′′), 131.67 (C‐4′), 130.89 (C‐2), 129.22 (C‐5), 128.98 (C‐3′), 128.68 (C‐5′′), 128.59 (C‐5′), 125.38 (C‐1′′), 123.69 (CF_3_), 123.50 (C‐6′), 123.38 (C‐2′′′′′), 118.34 (C‐3), 117.93 (C‐2′), 93.95 (C‐1′′′), 85.16 (C‐2′′′), 37.83 (C‐3′′′′), 30.38 (C‐1′′′′), 29.59 (C‐2′′′′), 20.25 (CH_3_); ^19^F NMR (376 MHz, CDCl_3_): δ [ppm] = −59.87; MS (ESI^‐^): *m/z* = 606.34 [M‐H]^‐^; HRMS (ESI): *m/z* calcd for C_34_H_23_F_3_N_5_O_3_^‐^: 606.1758 [M‐H]^‐^; found: 606.1759.

#### 4.2.26 N‐(4‐(3‐Aminopropyl)‐3‐(trifluoromethyl)phenyl)‐3‐(imidazo[1,2‐b]pyridazin‐3‐ylethynyl)−2,4‐dimethylbenzamide (12)

Synthesized according to General Procedure G, Colorless powder; 9.0 mg, 0.018 mmol, 92% yield. *R*_f_ = 0.19 (CH_2_Cl_2_/MeOH 95:5); ^1^H NMR (500 MHz, DMSO‐d_6_): δ [ppm] = 10.63 (s, 1H, NH(C = O)), 8.73–8.70 (m, 1H, H‐6′′), 8.28–8.24 (m, 1H, H‐4′′), 8.24–8.22 (m, 1H, H‐2′′), 8.20–8.16 (m, 1H, H‐2′), 7.97–7.92 (m, 1H, H‐6′), 7.77 (bs, 2H, NH_2_), 7.50–7.46 (m, 1H, H‐5′), 7.46–7.43 (m, 1H, H‐6), 7.41–7.36 (m, 1H, H‐5′′), 7.35–7.32 (m, 1H, H‐5), 2.92–2.82 (m, 2H, H‐3′′′′), 2.77 (t, ^3^ *J*_HH_ = 7.8 Hz, 2H, H‐1′′′′), 2.61 (s, 3H, CH_3_), 2.59 (s, 3H, CH_3_), 1.84 (tt, ^3^ *J*_HH_ = 8.1 Hz, ^3^ *J*_HH_ = 7.7 Hz, 2H, H‐2′′′′); ^13^C NMR (126 MHz, DMSO‐d_6_): δ [ppm] = 167.66 (C = O), 145.14 (C‐6′′), 141.43 (C‐4), 139.64 (C‐3′′), 137.98 (C‐2′′), 137.75 (C‐1′), 136.89 (C‐2), 135.05 (C‐1), 134.12 (C‐4′), 131.87 (C‐5′), 127.37 (C‐6), 127.17 (q, ^2^ *J*_CF_ = 29.4 Hz, C‐3′), 126.88 (C‐5), 126.08 (C‐4′′), 124.40 (q, ^1^ *J*_CF_ = 273.5 Hz, CF_3_), 123.25 (C‐6′), 122.93 (C‐3), 119.04 (C‐5′′), 116.65 (q, ^3^ *J*_CF_ = 6.8 Hz, C‐2′), 111.91 (C‐1′′), 95.45 (C‐1′′′), 85.41 (C‐2′′′), 38.59 (C‐3′′′′), 29.07 (C‐2′′′′), 28.37 (C‐1′′′′), 20.98 (CH_3_), 18.16 (CH_3_); ^19^F NMR (376 MHz, DMSO‐d_6_): δ [ppm] = −58.52; MS (ESI^+^): *m/z* = 492.41 [M + H]^+^; HRMS (ESI): *m/z* calcd for C_27_H_25_F_3_N_5_O^+^: 492.2006 [M + H]^+^; found: 492.1997.

#### 4.2.27 N‐(4‐(3‐(1,3‐Dioxoisoindolin‐2‐yl)propyl)‐3‐(trifluoromethyl)phenyl)‐3‐(imidazo[1,2‐b]pyridazin‐3‐ylethynyl)−2,4‐dimethylbenzamide (12a)

Synthesized according to General Procedure F, Colorless powder; 115 mg, 0.186 mmol, 84% yield. *R*_f_ = 0.26 (CH_2_Cl_2_/MeOH 99:1); ^1^H NMR (500 MHz, CDCl_3_): δ [ppm] = 8.56–8.51 (m, 1H, H‐6′′), 8.14–8.07 (m, 1H, H‐4′′), 8.03–7.99 (m, 1H, H‐2′′), 7.91–7.81 (m, 3H, H‐2′/H‐2′′′′′), 7.75–7.68 (m, 2H, H‐3′′′′′), 7.66–7.60 (m, 1H, H‐6′), 7.48–7.43 (m, 1H, H‐6), 7.40–7.34 (m, 2H, H‐5/H‐5′), 7.16–7.11 (m, 1H, H‐5′′), 3.77 (t, ^3^ *J*_HH_ = 7.6 Hz, 2H, H‐3′′′′), 2.83 (t, ^3^ *J*_HH_ = 7.8 Hz, 2H, H‐1′′′′), 2.69 (s, 3H, CH_3_), 2.58 (s, 3H, CH_3_), 2.01 (tt, ^3^ *J*_HH_ = 7.8 Hz, ^3^ *J*_HH_ = 7.5 Hz, 2H, H‐2′′′′); ^13^C NMR (126 MHz, CDCl_3_): δ [ppm] = 168.54 (N(C = O)_2_), 167.99 (C = O), 144.20 (C‐6′′), 143.05 (C‐4), 138.61 (C‐2), 137.56 (C‐3′′), 136.43 (C‐1), 134.40 (C‐2′′), 134.12 (C‐3′′′′′), 132.99 (C‐1′), 132.11 (C‐4′), 131.87 (C‐1′′′′′), 128.70 (C‐6), 128.60 (C‐5′), 127.06 (C‐3′), 126.29 (C‐5), 125.85 (C‐4′′), 125.37 (C‐6′), 124.37 (C‐3), 123.40 (C‐2′′′′′), 123.23 (CF_3_), 118.14 (C‐5′′), 117.66 (C‐2′), 106.04 (C‐1′′), 96.11 (C‐1′′′), 85.21 (C‐2′′′), 37.84 (C‐3′′′′), 30.39 (C‐1′′′′), 29.60 (C‐2′′′′), 21.64 (CH_3_), 20.25 (CH_3_); ^19^F NMR (376 MHz, CDCl_3_): δ [ppm] = −59.93; MS (ESI^+^): *m/z* = 622.48 [M + H]^+^; HRMS (ESI): *m/z* calcd for C_35_H_27_F_3_N_5_O_3_^+^: 622.2061 [M + H]^+^; found: 622.2034.

#### 4.2.28 3‐Ethynylimidazo[1,2‐b]pyridazine (13)

Triethylsilylacetylene (2.81 g, 3.60 mL, 20.0 mmol, 2.0 eq.) was added to a solution of **17** (1.98 g, 10.0 mmol, 1.0 eq.), Pd(PPh_3_)_2_Cl_2_ (351 mg, 0.500 mmol, 5 mol‐%) and CuI (95 mg, 0.500 mmol, 5 mol‐%) in MeCN (10 mL). The mixture was heated to reflux, and triethylamine (2.02 g, 2.80 mL, 20.0 mmol, 2.0 eq.) was added. The mixture was filtered over celite after 19 h and evaporated under reduced pressure. The residue was dissolved in THF (7.4 mL) and cooled to 0°C. A TBAF solution (11.0 mL, 11.0 mmol, 1.1 eq., 1 M in THF) was added dropwise to the cooled reaction mixture and stirred for 15 min. Afterward, the mixture was evaporated under reduced pressure. Flash chromatography (CH_2_Cl_2_/MeOH 99:1) gave the desired product **13** (1.33 g, 93%) as yellow crystals. *R*_f_ = 0.16 (CH_2_Cl_2_/MeOH 99:1); ^1^H NMR (500 MHz, CDCl_3_): δ [ppm] = 8.43–8.40 (m, 1H, H‐6), 7.97–7.96 (m, 1H, H‐2), 7.96–7.92 (m, 1H, H‐4), 7.10–7.05 (m, 1H, H‐5), 3.77 (s, 1H, H‐2′); ^13^C NMR (126 MHz, CDCl_3_): δ [ppm] = 143.96 (C‐6), 139.46 (C‐3), 138.96 (C‐2), 126.04 (C‐4), 117.93 (C‐5), 112.28 (C‐1), 87.36 (C‐2′), 70.65 (C‐1′); MS (ESI^+^): *m/z* = 143.96 [M + H]^+^; HRMS (ESI): *m/z* calcd for C_8_H_6_N_3_^+^: 144.0556 [M + H]^+^; found: 144.0553.

#### 4.2.29 3‐Iodo‐2,4‐dimethylbenzoic acid (14d)

3‐Iodo‐2,4‐dimethylbenzaldehyde (5.92 g, 22.8 mmol, 1.0 eq.) was dissolved in *tert*‐butanol (450 mL) and 2‐methyl‐2‐butene (114 mL). To this solution NaH_2_PO_4_ (18.9 g, 157 mmol, 6.9 eq.) in water (190 mL) was added over 15 min. After stirring at rt for 20 h, the solvent was removed under vacuum. The residue was dissolved in water and washed with petroleum ether (3 × 150 mL). The aqueous layer was acidified by addition of 2 M HCl and extracted with EtOAc (3 × 150 mL). The combined EtOAc layers were washed with water (150 mL) and brine (150 mL) and dried over Na_2_SO_4_. The mixture was filtered and evaporated under reduced pressure to give the pure product (5.34 g, 19.3 mmol, 85%) as a colorless powder. *R*_f_ = 0.12 (CH_2_Cl_2_/MeOH 97:3 + 0.1% FA); ^1^H NMR (300 MHz, CDCl_3_): δ [ppm] = 7.87−7.80 (m, 1H, H‐6), 7.20–7.13 (m, 1H, H‐5), 2.82 (s, 3H, CH_3_), 2.55 (s, 3H, CH_3_); ^13^C NMR (75 MHz, CDCl_3_): δ [ppm] = 172.63 (C = O), 147.77 (C‐2), 143.65 (C‐4), 130.67 (C‐1), 127.57 (C‐6), 127.00 (C‐5), 112.15 (C‐3), 31.19 (CH_3_), 28.08 (CH_3_); MS (ESI^+^): *m/z* = 277.21 [M + H]^+^; HRMS (ESI): *m/z* calcd for C_9_H_10_IO_2_^+^: 276.9720 [M + H]^+^; found: 276.9711.

#### 4.2.30 4‐((4‐Methylpiperazin‐1‐yl)methyl)‐3‐(trifluoromethyl)aniline (15a)

A solution of **23** (687 mg, 2.27 mmol, 1.0 eq.) in MeOH (5 mL) containing Pd/C (40 mg, 10 mol‐%) was stirred for 16 h under H_2_ atmosphere (1 bar). The mixture was filtered over celite and evaporated under reduced pressure to afford the desired product **15a** (622 mg, quant.) as a yellow oil. *R*_f_ = 0.26 (PE/EtOAc 7:3); ^1^H NMR (300 MHz, CDCl_3_): δ [ppm] = 7.49–7.41 (m, 1H, H‐6), 6.93–6.88 (m, 1H, H‐3), 6.82–6.74 (m, 1H, H‐5), 3.77 (bs, 2H, NH), 3.54 (s, 2H, CH_2_), 2.67–2.39 (m, 8H, H‐1′/H‐2′), 2.33 (s, 3H, CH_3_); MS (ESI^+^): *m/z* = 274.29 [M + H]^+^; HRMS (ESI): *m/z* calcd for C_13_H_19_F_3_N_3_^+^: 274.1526 [M + H]^+^; found: 274.1514.

#### 4.2.31 3‐Bromoimidazo[1,2‐*b*]pyridazine (17)

A solution of imidazo[1,2‐*b*]pyridazine **16** (5.96 g, 50.0 mmol, 1.0 eq.) and *N*‐bromosuccinimide (10.7 g, 60.0 mmol, 1.2 eq.) in DMF (90 mL) was heated at 90°C for 18 h. The reaction was quenched by adding water (150 mL). The aqueous layer was extracted with CH_2_Cl_2_ (5 x 100 mL), the combined organics were washed with brine and dried over Na_2_SO_4_. The mixture was filtered and evaporated in vacuo. Flash chromatography (PE/EtOAc 7:3) gave the desired product **17** (6.34 g, 64%) as yellow crystals. *R*_f_ = 0.09 (PE/EtOAc 4:1); ^1^H NMR (500 MHz, CDCl_3_): δ [ppm] = 8.46–8.40 (m, 1H, H‐6), 7.95–7.91 (m, 1H, H‐4), 7.78–7.74 (m, 1H, H‐2), 7.09–7.05 (m, 1H, H‐5); ^13^C NMR (126 MHz, CDCl_3_): δ [ppm] = 143.93 (C‐6), 139.79 (C‐3), 134.27 (C‐2), 126.01 (C‐4), 116.92 (C‐5), 100.74 (C‐1); MS (ESI^+^): *m/z* = 197.79 [M + H]^+^; HRMS (ESI): *m/z* calcd for C_6_H_5_BrN_3_^+^: 197.9661 [M + H]^+^; found: 197.9657.

#### 4.2.32 3‐Iodo‐2,4‐dimethylbenzaldehyde (20)

Dichloro(methoxy)methane (4.55 g, 3.50 mL, 39.6 mmol, 1.2 eq.) was added to a solution of TiCl_4_ (12.5 g, 7.25 mL, 66.0 mmol, 2.0 eq.) in CH_2_Cl_2_ (80 mL) at −78°C. After 10 min a solution of 2‐iodo‐1,3‐dimethylbenzene **19** (7.66 g, 4.75 mL, 33.0 mmol, 1.0 eq.) in CH_2_Cl_2_ (20 mL) was added. The mixture was stirred at −78°C for 2 h and then at rt for 4 h. The reaction was quenched by adding water (150 mL). The aqueous layer was extracted with CH_2_Cl_2_ (2 x 100 mL). The combined organics were washed with brine and dried over Na_2_SO_4_, filtered, and evaporated under reduced pressure to give the pure product (8.47 g, 99%) as a colorless powder. *R*_f_ = 0.19 (PE/EtOAc 7:3); ^1^H NMR (500 MHz, CDCl_3_): δ [ppm] = 10.18 (s, 1H, CHO), 7.71–7.66 (m, 1H, H‐6), 7.30–7.23 (m, 1H, H‐5), 2.84 (s, 3H, CH_3_), 2.56 (s, 3H, CH_3_); ^13^C NMR (126 MHz, CDCl_3_): δ [ppm] = 191.96 (CHO), 148.85 (C‐2), 144.19 (C‐4), 133.19 (C‐1), 131.47 (C‐6), 127.64 (C‐5), 112.61 (C‐3), 31.13 (CH_3_), 25.11 (CH_3_); MS (ESI^+^): *m/z* = 261.16 [M + H]^+^.

#### 4.2.33 1‐(Bromomethyl)‐4‐nitro‐2‐(trifluoromethyl)benzene (22)

Benzoyl peroxide (474 mg, 1.47 mmol, 10 mol‐%) was added portion wise to a heated solution of 1‐methyl‐4‐nitro‐2‐(trifluoromethyl)benzene **21** (3.00 g, 14.4 mmol, 1.0 eq.) and *N*‐bromosuccinimide (3.36 g, 16.1 mmol, 1.1 eq.) in acetic acid (20 mL). The reaction mixture was heated under reflux for 24 h, quenched with water, and extracted with EtOAc (4 x 50 mL). The combined organics were washed with brine and dried over Na_2_SO_4_. The mixture was filtered and evaporated in vacuo. Flash chromatography (PE/EtOAc 99:1) gave the desired product **22** (2.80 g, 9.86 mmol, 68%) as yellow oil. *R*_f_ = 0.40 (PE/EtOAc 9:1); ^1^H NMR (500 MHz, CDCl_3_): δ [ppm] = 8.56–8.51 (m, 1H, H‐3), 8.44–8.38 (m, 1H, H‐5), 7.87–7.80 (m, 1H, H‐6), 4.66 (s, 2H, CH_2_); ^13^C NMR (126 MHz, CDCl_3_): δ [ppm] = 146.46 (C‐4), 143.25 (C‐1), 134.31 (C‐6), 127.20 (C‐5), 124.72 (CF_3_), 122.07 (C‐3), 122.00 (C‐2), 26.41 (CH_2_); MS (ESI^+^): *m/z* = 284.45 [M + H]^+^; HRMS (ESI): *m/z* calcd for C_8_H_6_BrF_3_NO_2_^+^: 283.9529 [M + H]^+^; found: 283.9884.

#### 4.2.34 1‐Methyl‐4‐(4‐nitro‐2‐(trifluoromethyl)benzyl)piperazine (23)

To a solution of **22** (300 mg, 1.06 mmol, 1.0 eq.) and 1‐methylpiperazine (212 mg, 0.24 mL, 2.12 mmol, 2.0 eq.) in CH_2_Cl_2_ (2.1 mL), K_2_CO_3_ (147 mg, 1.06 mmol, 1.0 eq.) was added. After stirring the reaction mixture at rt for 3 h, water (10 mL) was added. The aqueous layer was extracted with CH_2_Cl_2_ (3 x 20 mL). The combined organics were washed with brine and dried over Na_2_SO_4_. The mixture was filtered and evaporated in vacuo. Flash chromatography (CH_2_Cl_2_/MeOH 95:5) gave the desired product **23** (290 mg, 90%) as yellow oil. *R*_f_ = 0.25 (CH_2_Cl_2_/MeOH 95:5); ^1^H NMR (300 MHz, CDCl_3_): δ [ppm] = 8.54–8.48 (m, 1H, H‐3), 8.42–8.34 (m, 1H, H‐5), 8.12–8.04 (m, 1H, H‐6), 3.76 (s, 2H, CH_2_), 2.72–2.47 (m, 8H, H‐1′/H‐2′), 2.37 (s, 3H, H‐3′); ^13^C NMR (75 MHz, CDCl_3_): δ [ppm] = 146.66 (C‐4), 145.69 (C‐1), 131.67 (C‐6), 129.59 (q, ^2^ *J*_CF_ = 30.0 Hz, C‐2), 126.56 (C‐5), 123.06 (q, ^1^ *J*_CF_ = 275.3 Hz, CF_3_), 121.68 (C‐3), 57.79 (CH_2_), 55.05 (C‐2′), 52.84 (C‐1′), 45.84 (C‐3′); MS (ESI^+^): *m/z* = 304.26 [M + H]^+^; HRMS (ESI): *m/z* calcd for C_13_H_17_F_3_N_3_O_2_^+^: 304.1267 [M + H]^+^; found: 304.1254.

#### 4.2.35 Tert‐Butyl (4‐iodo‐3‐(trifluoromethyl)phenyl)carbamate (25)

4‐Iodo‐3‐(trifluoromethyl)aniline **24** (2.92 g, 10.2 mmol, 1.0 eq.) was dissolved in THF (12 mL) and cooled to 0°C. A solution of Boc_2_O (2.45 g, 11.2 mmol, 1.2 eq.) in THF (3 mL) was slowly added. The reaction was allowed to warm up and stirred at rt for 12 h. The mixture was diluted with EtOAc (50 mL) and washed with brine (3 × 50 mL). The organic layer was dried over Na_2_SO_4_, filtered and evaporated in vacuo. Flash chromatography (PE/EtOAc 95:5) afforded the product **25** (2.81 g, 71%) as a colorless solid. *R*_f_ = 0.51 (PE/EtOAc 9:1); ^1^H NMR (500 MHz, CDCl_3_): δ [ppm] = 7.89–7.85 (m, 1H, H‐2), 7.72–7.69 (m, 1H, H‐6), 7.30–7.24 (m, 1H, H‐5), 6.59 (bs, 1H, NH), 1.52 (s, 9H, H‐8); ^13^C NMR (125 MHz, CDCl_3_): δ [ppm] = 152.32 (C = O), 142.50 (C‐1), 138.87 (C‐5), 134.16 (q, ^2^ *J*_CF_ = 31.0 Hz, C‐3), 122.65 (q, ^1^ *J*_CF_ = 273.8 Hz, CF_3_), 122.48 (C‐6), 117.81 (q, ^3^ *J*_CF_ = 5.5 Hz, C‐2), 81.93 (C‐4), 81.72 (C‐7), 28.39 (C‐8); MS (ESI^+^): *m/z* = 388.45 [M + H]^+^; HRMS (ESI): *m/z* calcd for C_12_H_14_F_3_INO_2_^+^: 388.0016 [M + H]^+^; found: 388.0001.

#### 4.2.36 Ethyl (E)‐3‐(4‐((*tert*‐butoxycarbonyl)amino)‐2‐(trifluoromethyl)phenyl)acrylate (26a)

Ethyl acrylate (776 mg, 0.85 mL, 7.75 mmol, 3.0 eq.) was added to a suspension of **25** (1.00 g, 2.58 mmol, 1.0 eq) and Pd(dppf)Cl_2_ (94 mg, 0.13 mmol, 5 mol‐%) in MeCN (20 mL). DIPEA (1.09 g, 1.51 mL, 7.75 mmol, 3.0 eq.) was added, and the reaction mixture was heated to reflux for 48 h. The mixture was diluted with MeCN (50 mL), filtered over celite and evaporated under reduced pressure. Flash chromatography gave **26a** (898 mg, 97%) as a colorless powder. *R*_f_ = 0.38 (PE/EtOAc 8:2); ^1^H NMR (300 MHz, CDCl_3_): δ [ppm] = 7.98 (d, ^3^ *J*_HH_ = 15.8 Hz, 1H, H‐1′), 7.76–7.71 (m, 1H, H‐2), 7.69–7.62 (m, 1H, H‐6), 7.61–7.54 (m, 1H, H‐5), 6.71 (bs, 1H, NH), 6.35 (d, ^3^ *J*_HH_ = 15.8 Hz, 1H, H‐2′), 4.26 (q, ^3^ *J*_HH_ = 7.2 Hz, 2H, H‐3′), 1.52 (s, 9H, H‐8), 1.33 (t, ^3^ *J*_HH_ = 7.2 Hz, 3H, H‐4′); ^13^C NMR (75 MHz, CDCl_3_): δ [ppm] = 166.51 (C = O(OEt)), 152.27 (C = O), 140.04 (C‐1′), 139.59 (C‐1), 129.02 (C‐2′), 128.96 (C‐4), 127.47 (C‐3), 127.43 (C‐5), 121.96 (C‐6), 121.12 (CF_3_), 120.93 (C‐2), 81.76 (C‐7), 60.84 (C‐3′), 28.37 (C‐8), 14.42 (C‐4′); MS (ESI^‐^): *m/z* = 358.37 [M‐H]^‐^; HRMS (ESI): *m/z* calcd for C_17_H_19_F_3_NO_4_^‐^: 358.1272 [M‐H]^‐^; found: 358.1270.

#### 4.2.37 Ethyl 3‐(4‐((*tert*‐butoxycarbonyl)amino)‐2‐(trifluoromethyl)phenyl)propanoate (26b)

A solution of **26a** (1.00 g, 2.78 mmol, 1.0 eq) in MeOH (20 mL) containing Pd/C (50 mg, 10 mol‐%) was stirred under H_2_ atmosphere (1 bar) for 16 h. The mixture was filtered over celite and evaporated under reduced pressure to afford the desired product **26b** (968 mg, 96%) as a colorless powder. *R*_f_ = 0.55 (PE/EtOAc 7:3); ^1^H NMR (300 MHz, CDCl_3_): δ [ppm] = 7.66–7.61 (m, 1H, H‐2), 7.53–7.43 (m, 1H, H‐6), 7.30–7.23 (m, 1H, H‐5), 6.55 (bs, 1H, NH), 4.14 (q, ^3^ *J*_HH_ = 7.1 Hz, 2H, H‐3′), 3.06 (t, ^3^ *J*_HH_ = 7.8 Hz, 2H, H‐1′), 2.57 (t, ^3^ *J*_HH_ = 8.0 Hz, 2H, H‐2′), 1.52 (s, 9H, H‐8), 1.25 (t, ^3^ *J*_HH_ = 7.2 Hz, 3H, H‐4′); ^13^C NMR (75 MHz, CDCl_3_): δ [ppm] = 172.73 (C = O(OEt)), 152.67 (C = O), 137.02 (C‐1), 133.54 (C‐4), 131.91 (C‐5), 129.23 (q, ^2^ *J*_CF_ = 31.0 Hz, C‐3), 124.17 (q, ^1^ *J*_CF_ = 272.3 Hz, CF_3_), 121.75 (C‐2), 116.34 (C‐6), 81.23 (C‐7), 60.69 (C‐3′), 36.04 (C‐2′), 28.42 (C‐8), 27.36 (C‐1′), 14.33 (C‐4′); MS (ESI^‐^): *m/z* = 360.39 [M‐H]^‐^; HRMS (ESI): *m/z* calcd for C_17_H_21_F_3_NO_4_^‐^: 360.1428 [M‐H]^‐^; found: 360.1427.

#### 4.2.38 Tert‐Butyl (E)‐(4‐(3‐hydroxyprop‐1‐en‐1‐yl)‐3‐(trifluoromethyl)phenyl)carbamate (27a)

DIBAL‐H (10.7 mL, 10.7 mmol, 4.0 eq, 1 M in THF) was added dropwise to a cooled solution of **26a** (960 mg, 2.67 mmol, 1.0 eq.) in THF (20 mL). After 15 min, the reaction was completed, and the mixture was diluted with THF (20 mL). Water (0.5 mL) was slowly added, followed by 15% NaOH solution (0.5 mL) and water (1 mL). The reaction mixture was stirred at rt for 15 min and was dried over MgSO_4_. The mixture was filtered and evaporated in vacuo. Flash chromatography (PE/EtOAc 8:2) afforded the desired product **27a** (789 mg, 93%) as a colorless oil. *R*_f_ = 0.20 (PE/EtOAc 7:3); ^1^H NMR (500 MHz, CDCl_3_): δ [ppm] = 7.66–7.64 (m, 1H, H‐2), 7.58–7.54 (m, 1H, H‐6), 7.54–7.47 (m, 1H, H‐5), 6.94–6.88 (m, 1H, H‐1′), 6.60 (bs, 1H, NH), 6.28 (dt, ^3^ *J*_HH_ = 15.7 Hz, ^3^ *J*_HH_ = 5.7 Hz, 1H, H‐2′), 4.33 (d, ^3^ *J*_HH_ = 5.7 Hz, 2H, H‐3′), 1.52 (s, 9H, H‐8); ^13^C NMR (75 MHz, CDCl_3_): δ [ppm] = 152.53 (C = O), 137.93 (C‐1), 131.62 (C‐4), 130.27 (C‐5), 128.38 (C‐2′), 128.18 (q, ^2^ *J*_CF_ = 31.0 Hz, C‐3), 126.55 (C‐1′), 124.14 (q, ^1^ *J*_CF_ = 273.8 Hz, CF_3_), 121.49 (C‐6), 115.76 (C‐2), 81.39 (C‐7), 63.80 (C‐3′), 28.41 (C‐8); ^19^F NMR (471 MHz, CDCl_3_): δ [ppm] = −59.62; MS (ESI^‐^): *m/z* = 316.29 [M‐H]^‐^; HRMS (ESI): *m/z* calcd for C_15_H_17_F_3_NO_3_^‐^: 316.1166 [M‐H]^‐^; found: 316.1164.

#### 4.2.39 Tert‐Butyl (4‐(3‐hydroxypropyl)‐3‐(trifluoromethyl)phenyl)carbamate (27b)

DIBAL‐H (8.80 mL, 8.80 mmol, 4.0 eq, 1 M in THF) was added dropwise to a cooled solution of **26b** (800 mg, 2.21 mmol, 1.0 eq.) in THF (20 mL). After 15 min, the reaction was completed, and the mixture was diluted with THF (20 mL). Water (0.5 mL) was slowly added, followed by 15% NaOH solution (0.5 mL) and water (1 mL). The reaction mixture was stirred at rt for 15 min and was dried over MgSO_4_. The mixture was filtered and evaporated in vacuo. Flash chromatography (PE/EtOAc 8:2) afforded the desired product **27b** (507 mg, 72%) as a colorless oil. *R*_f_ = 0.21 (PE/EtOAc 7:3); ^1^H NMR (400 MHz, CDCl_3_): δ [ppm] = 7.62–7.61 (m, 1H, H‐2), 7.49–7.47 (m, 1H, H‐6), 7.27–7.25 (m, 1H, H‐5), 6.58 (bs, 1H, NH), 3.70 (t, ^3^ *J*_HH_ = 6.4 Hz, 2H, H‐3′), 2.81 (t, ^3^ *J*_HH_ = 7.7 Hz, 2H, H‐1′), 1.85 (tt, ^3^ *J*_HH_ = 7.7 Hz, ^3^ *J*_HH_ = 6.4 Hz, 2H, H‐2′), 1.51 (s, 9H, H‐8); ^13^C NMR (100 MHz, CDCl_3_): δ [ppm] = 152.77 (C = O), 136.58 (C‐1), 135.01 (C‐4), 131.92 (C‐5), 129.05 (q, ^2^ *J*_CF_ = 30.0 Hz, C‐3), 124.40 (q, ^1^ *J*_CF_ = 272.3 Hz, CF_3_), 121.80 (C‐6), 116.25 (C‐2), 81.15 (C‐7), 62.44 (C‐3′), 34.66 (C‐1′), 28.42 (C‐2′, C‐8); ^19^F NMR (376 MHz, CDCl_3_): δ [ppm] = −59.89; MS (ESI^‐^): *m/z* = 318.31 [M‐H]^‐^; HRMS (ESI): *m/z* calcd for C_15_H_19_F_3_NO_3_^‐^: 318.1323 [M‐H]^‐^; found: 318.1322.

#### 4.2.40 Tert‐Butyl (E)‐(4‐(3‐(1,3‐dioxoisoindolin‐2‐yl)prop‐1‐en‐1‐yl)‐3‐(trifluoromethyl)phenyl)carbamate (28a)

To a mixture of **27a** (1.00 g, 3.15 mmol, 1.0 eq.), phthalimide (556 mg, 3.78 mmol, 1.2 eq.), and PPh_3_ (991 mg, 3.78 mmol, 1.2 eq.) in THF (45 mL), DEAD (1.72 mL, 3.78 mmol, 1.2 eq., 40% in toluene) was slowly added at 0°C. The reaction mixture was stirred at rt for 18 h and quenched with NaHCO_3_ solution. The aqueous layer was extracted with EtOAc (3 x 50 mL). The combined organics were washed with brine and dried over Na_2_SO_4_. The mixture was filtered and evaporated in vacuo. Flash chromatography (PE/EtOAc 8:2) afforded the desired product **28a** (1.03 g, 73%) as a colorless powder. *R*_f_ = 0.50 (CH_2_Cl_2_/MeOH 9:1); ^1^H NMR (500 MHz, CDCl_3_): δ [ppm] = 7.88–7.85 (m, 2H, H‐2′′), 7.78–7.71 (m, 2H, H‐3′′), 7.64–7.62 (m, 1H, H‐2), 7.52–7.44 (m, 2H, H‐5, H‐6), 6.98–6.91 (m, 1H, H‐1′), 6.63 (bs, 1H, NH), 6.15 (dt, ^3^ *J*_HH_ = 15.6 Hz, ^3^ *J*_HH_ = 6.5 Hz, 1H, H‐2′), 4.46 (dd, ^3^ *J*_HH_ = 6.4 Hz, ^4^ *J*_HH_ = 1.3 Hz, 2H, H‐3′), 1.51 (s, 9H, H‐8); ^13^C NMR (125 MHz, CDCl_3_): δ [ppm] = 168.04 (N(C = O)_2_), 152.47 (C = O), 138.11 (C‐1), 134.48 (C‐1′′), 134.38 (C‐4), 134.17 (C‐3′′), 132.75 (C‐5), 132.25 (C‐3), 129.74 (CF_3_), 129.30 (C‐1′), 128.42 (C‐5), 125.83 (C‐2′), 123.76 (C‐2′′), 121.30 (C‐6), 115.62 (C‐2), 82.41 (C‐7), 39.83 (C‐3′), 28.40 (C‐8); MS (ESI^‐^): *m/z* = 445.36 [M‐H]^‐^; HRMS (ESI): *m/z* calcd for C_23_H_22_F_3_N_2_O_4_^+^: 447.1526 [M + H]^+^; found: 447.1525.

#### 4.2.41 Tert‐Butyl (4‐(3‐(1,3‐dioxoisoindolin‐2‐yl)propyl)‐3‐(trifluoromethyl)phenyl)carbamate (28b)

To a mixture of **27b** (380 mg, 1.19 mmol, 1.0 eq.), phthalimide (210 mg, 1.43 mmol, 1.2 eq.), and PPh_3_ (375 mg, 1.43 mmol, 1.2 eq.) in THF (15 mL), DEAD (0.68 mL, 1.4 mmol, 1.2 eq., 40% in toluene) was slowly added at 0°C. The reaction mixture was stirred at rt for 18 h and quenched with NaHCO_3_ solution. The aqueous layer was extracted with EtOAc (3 x 50 mL). The combined organics were washed with brine and dried over Na_2_SO_4_. The mixture was filtered and evaporated in vacuo. Flash chromatography (PE/EtOAc 8:2) afforded the desired product **28b** (507 mg, 95%) as a colorless powder. *R*_f_ = 0.50 (CH_2_Cl_2_/MeOH 9:1); ^1^H NMR (500 MHz, CDCl_3_): δ [ppm] = 7.86–7.83 (m, 2H, H‐2′′), 7.72–7.69 (m, 2H, H3′′), 7.63–7.60 (m, 1H, H‐2), 7.50–7.40 (m, 1H, H‐6), 7.29–7.24 (m, 1H, H‐5), 6.57 (bs, 1H, NH), 3.76 (t, ^3^ *J*_HH_ = 7.2 Hz, 2H, H‐3′), 2.77 (t, ^3^ *J*_HH_ = 7.9 Hz, 2H, H‐1′), 1.97 (tt, ^3^ *J*_HH_ = 7.9 Hz, ^3^ *J*_HH_ = 7.2 Hz, 2H, H‐2′), 1.51 (s, 9H, H‐8); ^13^C NMR (126 MHz, CDCl_3_): δ [ppm] = 168.53 (N(C = O)_2_), 152.70 (C = O), 136.75 (C‐1), 134.48 (C‐1′′), 134.18 (C‐4), 134.09 (C‐3′′), 131.67 (C‐5), 128.96 (C‐3), 123.76 (CF_3_), 123.38 (C‐2′′), 121.76 (C‐6), 116.20 (C‐2), 81.24 (C‐7), 37.86 (C‐3′), 30.42 (C‐2′), 29.42 (C‐1′), 28.41 (C‐8); MS (ESI^‐^): *m/z* = 447.35 [M‐H]^‐^; HRMS (ESI): *m/z* calcd for C_23_H_22_F_3_N_2_O_4_^‐^: 447.1537 [M‐H]^‐^; found: 447.1535.

#### 4.2.42 Methyl 3‐iodo‐2‐methylbenzoate (31a)

Synthesized according to General Procedure A, White powder; 505 mg, 1.83 mmol, 96% yield. *R*_f_ = 0.64 (PE/EtOAc 8:2); ^1^H NMR (300 MHz, CDCl_3_): δ [ppm] = 8.01–7.94 (m, 1H, H‐4), 7.77–7.69 (m, 1H, H‐6), 6.96–6.88 (m, 1H, H‐5), 3.90 (s, 3H, OCH_3_), 2.66 (s, 3H, CH_3_); ^13^C NMR (75 MHz, CDCl_3_): δ [ppm] = 168.04 (C = O), 142.68 (C‐4), 141.61 (C‐2), 131.98 (C‐1), 130.07 (C‐6), 127.21 (C‐5), 104.22 (C‐3), 52.43 (OCH_3_), 26.63 (CH_3_); MS (ESI^+^): *m/z* = 277.14 [M + H]^+^; HRMS (ESI): *m/z* calcd for C_9_H_10_IO_2_^+^: 276.9720 [M + H]^+^; found: 276.9712.

#### 4.2.43 Methyl 3‐iodo‐4‐methylbenzoate (31b)

Synthesized according to General Procedure A, White powder; 500 mg, 1.81 mmol, 95% yield. *R*_f_ = 0.64 (PE/EtOAc 8:2); ^1^H NMR (300 MHz, CDCl_3_): δ [ppm] = 8.47–8.42 (m, 1H, H‐2), 7.92–7.85 (m, 1H, H‐6), 7.31–7.25 (m, 1H, H‐5), 3.89 (s, 3H, OCH_3_), 2.47 (s, 3H, CH_3_); ^13^C NMR (75 MHz, CDCl_3_): δ [ppm] = 165.69 (C = O), 146.90 (C‐4), 140.10 (C‐2), 129.63 (C‐5), 129.38 (C‐1/C‐6), 100.61 (C‐3), 52.36 (OCH_3_), 28.47 (CH_3_); MS (ESI^+^): *m/z* = 277.12 [M + H]^+^; HRMS (ESI): *m/z* calcd for C_9_H_10_IO_2_^+^: 276.9720 [M + H]^+^; found: 276.9713.

#### 4.2.44 Methyl 5‐iodo‐2‐methylbenzoate (31c)

Synthesized according to General Procedure A, White powder; 505 mg, 1.83 mmol, 96% yield. *R*_f_ = 0.64 (PE/EtOAc 8:2); ^1^H NMR (300 MHz, CDCl_3_): δ [ppm] = 8.24–8.19 (m, 1H, H‐2), 7.72–7.67 (m, 1H, H‐4), 7.02–6.95 (m, 1H, H‐5), 3.89 (s, 3H, OCH_3_), 2.53 (s, 3H, CH_3_); ^13^C NMR (75 MHz, CDCl_3_): δ [ppm] = 166.68 (C = O), 140.83 (C‐4), 139.96 (C‐6), 139.32 (C‐2), 133.64 (C‐5), 131.54 (C‐1), 90.03 (C‐3), 52.23 (CH_3_O), 21.44 (CH_3_); MS (ESI^+^): *m/z* = 277.09 [M + H]^+^; HRMS (ESI): *m/z* calcd for C_9_H_10_IO_2_^+^: 276.9720 [M + H]^+^; found: 276.9712.

#### 4.2.45 Methyl 3‐iodo‐2,4‐dimethylbenzoate (31d)

Synthesized according to General Procedure A, White powder; 511 mg, 1.76 mmol, 92% yield. *R*_f_ = 0.64 (PE/EtOAc 8:2); ^1^H NMR (300 MHz, CDCl_3_): δ [ppm] = 7.66–7.60 (m, 1H, H‐6), 7.15–7.09 (m, 1H, H‐5), 3.88 (s, 3H, OCH_3_), 2.72 (s, 3H, CH_3_), 2.52 (s, 3H, CH_3_); ^13^C NMR (100 MHz, CDCl_3_): δ [ppm] = 168.18 (C = O), 146.44 (C‐4), 142.40 (C‐2), 129.54 (C‐6), 129.18 (C‐1), 126.82 (C‐5), 111.73 (C‐3), 52.34 (OCH_3_), 30.95 (CH_3_), 27.81 (CH_3_); MS (ESI^+^): *m/z* = 291.12 [M + H]^+^; HRMS (ESI): *m/z* calcd for C_10_H_12_IO_2_^+^: 290.9876 [M + H]^+^; found: 290.9865.

#### 4.2.46 Methyl 3‐(imidazo[1,2‐b]pyridazin‐3‐ylethynyl)‐2‐methylbenzoate (32a)

Synthesized according to General Procedure B, Yellow solid; 513 mg, 1.76 mmol, 97%. *R*_f_ = 0.24 (CH_2_Cl_2_/MeOH 98:2); ^1^H NMR (500 MHz, DMSO‐d_6_): δ [ppm] = 8.74–8.68 (m, 1H, H‐6′), 8.51–8.11 (m, 1H, H‐2′), 8.30–8.18 (m, 1H, H‐4′), 7.83–7.80 (m, 1H, H‐6), 7.79–7.75 (m, 1H, H‐4), 7.43–7.35 (m, 2H, H‐5/H‐5′), 3.86 (s, 3H, OCH_3_), 2.74 (s, 3H, CH_3_); ^13^C NMR (126 MHz, DMSO‐d_6_): δ [ppm] = 167.16 (C = O), 145.06 (C‐6′), 139.88 (C‐2), 138.53 (C‐3′), 138.39 (C‐2′), 134.85 (C‐4), 130.98 (C‐1), 130.35 (C‐6), 126.26 (C‐5), 126.22 (C‐4′), 123.65 (C‐3), 119.03 (C‐5′), 107.58 (C‐1′), 96.57 (C‐1′′), 81.43 (C‐2′′), 52.24 (OCH_3_), 18.51 (CH_3_); MS (ESI^+^): *m/z* = 292.23 [M + H]^+^; HRMS (ESI): *m/z* calcd for C_17_H_14_N_3_O_2_^+^: 292.1081 [M + H]^+^; found: 292.1066.

#### 4.2.47 Methyl 3‐(imidazo[1,2‐b]pyridazin‐3‐ylethynyl)‐4‐methylbenzoate (32b)

Synthesized according to General Procedure B, Yellow solid; 463 mg, 1.59 mmol, 88%. *R*_f_ = 0.24 (CH_2_Cl_2_/MeOH 98:2); ^1^H NMR (500 MHz, DMSO‐d_6_): δ [ppm] = 8.75–8.69 (m, 1H, H‐6′), 8.36–8.18 (m, 1H, H‐2′), 8.28–8.22 (m, 1H, H‐4′), 8.07–8.04 (m, 1H, H‐2), 7.92–7.87 (m, 1H, H‐6), 7.54–7.50 (m, 1H, H‐5), 7.42–7.35 (m, 1H, H‐5′), 3.87 (s, 3H, CH_3_O), 2.59 (s, 3H, CH_3_); ^13^C NMR (126 MHz, DMSO‐d_6_): δ [ppm] = 165.42 (C = O), 145.08 (C‐6′), 144.88 (C‐4), 139.71 (C‐3′), 138.48 (C‐2′), 131.67 (C‐2), 130.38 (C‐5), 129.41 (C‐6), 127.70 (C‐1), 126.16 (C‐4′), 122.22 (C‐3), 119.07 (C‐5′), 111.87 (C‐1′), 95.97 (C‐1′′), 81.39 (C‐2′′), 52.28 (OCH_3_), 20.51 (CH_3_); MS (ESI^+^): *m/z* = 292.24 [M + H]^+^; HRMS (ESI): *m/z* calcd for C_17_H_14_N_3_O_2_^+^: 292.1081 [M + H]^+^; found: 292.1067.

#### 4.2.48 Methyl 5‐(imidazo[1,2‐b]pyridazin‐3‐ylethynyl)‐2‐methylbenzoate (32c)

Synthesized according to General Procedure B, Yellow solid; 411 mg, 1.41 mmol, 78%. *R*_f_ = 0.24 (CH_2_Cl_2_/MeOH 98:2); ^1^H NMR (500 MHz, DMSO‐d_6_): δ [ppm] = 8.74–8.68 (m, 1H, H‐6′), 8.50–8.11 (m, 1H, H‐2′), 8.30–8.21 (m, 1H, H‐4′), 8.00–7.97 (m, 1H, H‐2), 7.72–7.67 (m, 1H, H‐4), 7.45–7.41 (m, 1H, H‐5), 7.41–7.36 (m, 1H, H‐5′), 3.86 (s, 3H, OCH_3_), 2.55 (s, 3H, CH_3_); ^13^C NMR (126 MHz, DMSO‐d_6_): δ [ppm] = 166.38 (C = O), 144.96 (C‐6′), 140.34 (C‐6), 138.64 (C‐3′), 138.61 (C‐2′), 134.09 (C‐4), 132.59 (C‐2), 132.43 (C‐5), 130.00 (C‐1), 126.23 (C‐4′), 119.44 (C‐3), 119.00 (C‐5′), 97.03 (C‐1′′), 77.15 (C‐2′′), 52.17 (OCH_3_), 21.00 (CH_3_); MS (ESI^+^): *m/z* = 292.20 [M + H]^+^; HRMS (ESI): *m/z* calcd for C_17_H_14_N_3_O_2_^+^: 292.1081 [M + H]^+^; found: 292.1067.

#### 4.2.49 Methyl 3‐(imidazo[1,2‐b]pyridazin‐3‐ylethynyl)−2,4‐dimethylbenzoate (32d)

Synthesized according to General Procedure B, Yellow solid; 216 mg, 0.706 mmol, 39%. *R*_f_ = 0.24 (CH_2_Cl_2_/MeOH 98:2); ^1^H NMR (300 MHz, CDCl_3_): δ [ppm] = 8.61–8.55 (m, 1H, H‐6′), 8.27–8.18 (m, 1H, H‐4′), 8.17–8.00 (m, 1H, H‐2′), 7.83–7.76 (m, 1H, H‐6), 7.31–7.24 (m, 1H, H‐5′), 7.20–7.13 (m, 1H, H‐5), 3.90 (s, 3H, OCH_3_), 2.86 (s, 3H, CH_3_), 2.61 (s, 3H, CH_3_); ^13^C NMR (100 MHz, CDCl_3_): δ [ppm] = 168.09 (C = O), 144.89 (C‐6′), 143.99 (C‐4), 142.16 (C‐2), 138.08 (C‐3′), 134.63 (C‐2′), 130.47 (C‐6), 128.10 (C‐1), 127.95 (C‐4′), 126.84 (C‐5), 125.99 (C‐1′), 124.57 (C‐3), 117.74 (C‐5′), 96.21 (C‐1′′), 85.11 (C‐2′′), 52.09 (OCH_3_), 21.94 (CH_3_), 19.51 (CH_3_); MS (ESI^+^): *m/z* = 306.28 [M + H]^+^; HRMS (ESI): *m/z* calcd for C_18_H_16_N_3_O_2_^+^: 306.1237 [M + H]^+^; found: 306.1224.

## 5 Biological Methods

### 5.1 Cell Growth Inhibition Assay

The MDA‐MB‐231 breast cancer cell line and MRC‐5 cell line (human lung fibroblasts) were cultured in DMEM. All media were supplemented with stabilized 2 mM l‐glutamine (Sigma–Aldrich), 1% pen/strep solution (Invitrogen), and 10% fetal calf serum (FCS, Sigma–Aldrich). The cells were seeded in a 96 well plate in medium containing 10% FCS and let to resume growth overnight. The number of seeded cells per well were as indicated in the Figures or Tables. The next day, the medium was replaced by medium containing either 5% FCS or 0.1% FCS and the test compounds or DMSO as a control (max. 0.2% final concentration). The cells were grown for 3–4 days at 37°C in a humidified incubator containing 5% CO_2_, without further change of medium, before the detection was carried out using 3‐(4,5‐dimethylthiazol‐2‐yl)−2,5‐diphenyltetrazoliumbromide (MTT, Cat. No. M5655, Sigma–Aldrich) as previously described [34]. GI_50_ values and standard deviations were rounded to numbers with significant figures as previously recommended [35].

### 5.2 Colony Formation Assay

The colony formation assay was carried out according to Franken and coworkers [28]. In brief, MDA‐MB‐231 cells were seeded as single cells in 6‐well plates (1000 cells per well) in DMEM supplemented with 10% FCS and allowed to adhere to the bottom for 16 h. The medium was then exchanged for DMEM containing 0.4% FCS and the test compounds in concentrations as indicated in the figure. After 24 h, the medium was again exchanged for DMEM containing 10% FCS, and the plates were left in the cell incubator for 12–14 days. The wells were then rinsed carefully with PBS, fixed and stained using crystal violet. The images of each well were analyzed using ImageJ software (https://imagej.net/).

### 5.3 DSF‐Based Selectivity Screening against a Curated Kinase Library

The assay was performed as previously described [31, 36]. Briefly, recombinant protein kinase domains at a concentration of 2 μM were mixed with 10 μM compound in a buffer containing 20 mM HEPES (pH 7.5), and 500 mM NaCl. SYPRO Orange (5000×, Invitrogen) was added as a fluorescence probe (1 µl per mL). Subsequently, temperature‐dependent protein unfolding profiles were measured using the QuantStudio 5 realtime PCR machine (Thermo Fisher). Excitation and emission filters were set to 465 nm and 590 nm, respectively. The temperature was raised with a step rate of 3°C per minute. Data points were analyzed with the internal software (Thermal Shift SoftwareTM Version 1.4, Thermo Fisher) using the Boltzmann equation to determine the inflection point of the transition curve.

### 5.4 Microscale Thermophoresis (MST) Measurements

MST experiments were carried out on a Monolith NT.115 (NanoTemper) using standard capillaries (Nanotemper Technologies, GmbH, Munich, Germany) in MST buffer (PBS as provided in the labeling kit (see below), with additional NaCl added a final concentration of 150 mM, and 0.05% Tween20) according to the manufacturer's protocol. The protein solution (200 nM) in MST buffer was mixed 1:1 with dye solution (Monolith His‐Tag Labeling Kit RED‐tris‐NTA 2nd Generation, 100 nM in MST buffer), and the resultant labeled protein solution mixed with test compound prediluted in MST buffer additionally containing 2% DMSO. The final measuring solutions contained 50 nM (His)_6_‐ROR1 in MST buffer with 1% DMSO and a starting concentration of the test compound between 20 and 50 µM (according to the affinity of the compound observed in the first run) which was then used to make 16 serial dilutions (1:1). Standard settings as recommended by the manufacturer (40% LED/excitation power and medium (40%) MST power in NT. control) were used during the measurements. All measurements were performed in duplicates at 25°C. Data analysis and evaluation were performed using MO‐affinity analysis software (NanoTemper). The K_d_ values were calculated using a 1:1 binding model (“K_d_ model”) and indicated as mean values ± standard deviation from two independent measurements. (His)_6_‐ROR1 was expressed and purified as previously described [10].

## Funding

This work was supported by the Deutsche Forschungsgemeinschaft (grants INST 256/540‐1 FUGG, INST 256/472‐1 FUGG).

## Conflicts of Interest

The authors declare no conflicts of interest.

## Supporting information

Supplementary Material

## Data Availability

The data that support the findings of this study are available from the corresponding author upon reasonable request.
