# Supplementary material for: Structure‐Based Design, Synthesis, and Evaluation of Novel Ponatinib Derivatives With a Significantly Altered Selectivity Profile
Source: ChemMedChem. 2026 Jun 18;21(11):e202501007. doi: 10.1002/cmdc.202501007 (PMC13277965; doi:10.1002/cmdc.202501007)
Supplement: Supplementary file 1 — Supplementary Material [file CMDC-21-e202501007-s001.pdf]

Supporting Information

Structure-based design, synthesis, and evaluation of novel ponatinib derivatives with a significantly altered selectivity profile

Tobias Betzholz, Ting Liu, Andreas Krämer, Verena Dederer, Stefan Knapp, Sebastian Mathea, Christian Ducho* and Matthias Engel*

Table of Contents

1. Figure S1. Main Targets of ponatinib in the human kinome…………………………………………S2

2. Figure S2. Structures of all target compounds ……………………..……………………………………. S3

3. Figure S3. DSF results with compounds **5**, **10** and ponatinib…………..………………………………S5

4. Tables S1-S3: Yields of synthesis steps from the main part……..………………………………………S6

5. Scheme S1 and Table S4: Additional syntheses and corresponding yields……………………….… S7

6. Table S5. ΔTm values of the DSF kinase profiling with **5**, **10**, ponatinib and reference inhibitors...S11

7. Table S6. Binding affinity constants of the compounds without basic moiety towards ROR1……..S13

8. Table S7. Comparison of ΔTm values and inhibition strengths………………………………………S13

9. Figure S4. MST binding curves with ROR1……………………………………………………………..S14

10. NMR spectra……………………………………………………………………………………………S22

11. References of the Supporting Information…………………………………………………….……S101

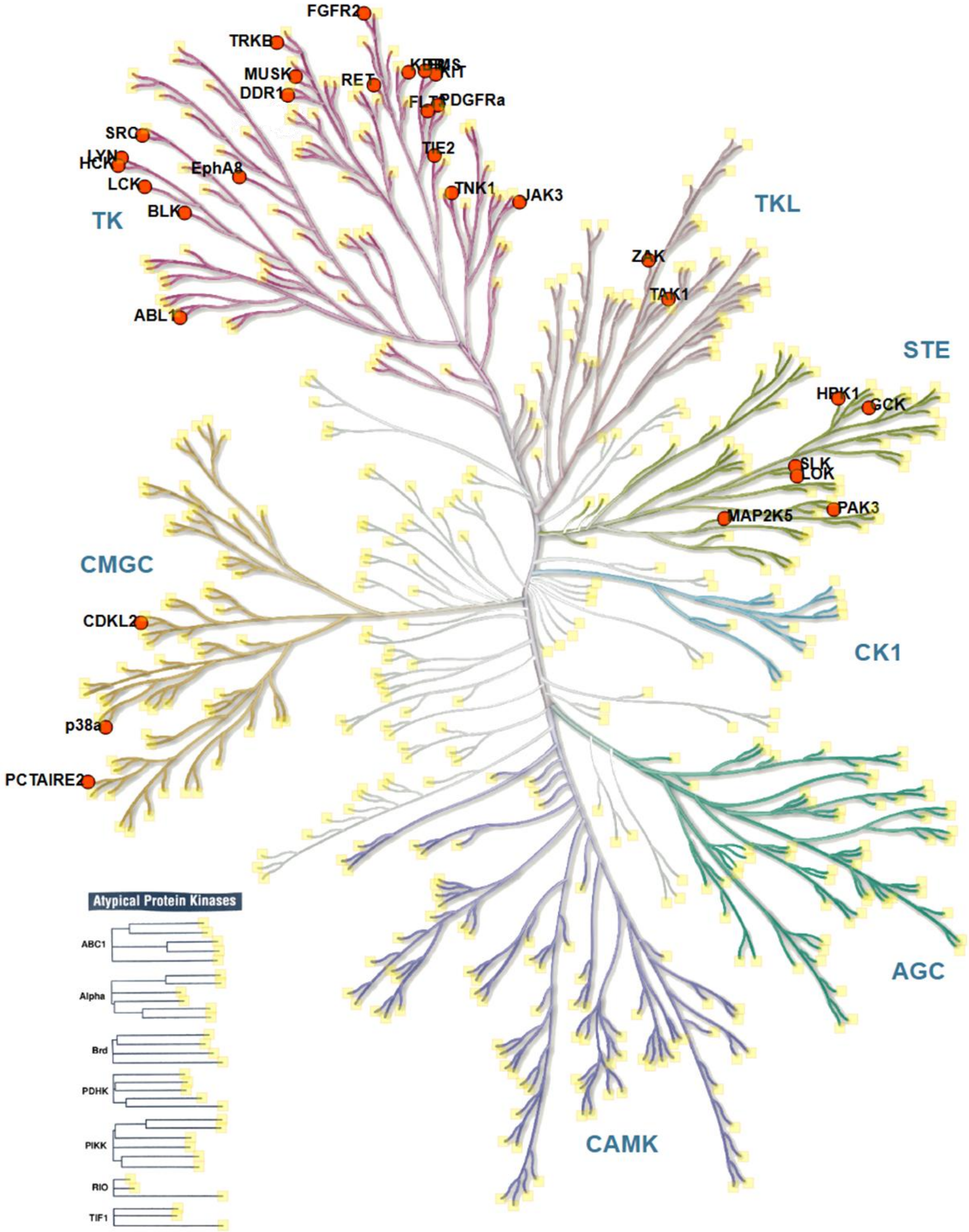

Figure S1. Main targets of ponatinib in the human kinome. Red dots denote kinases that are inhibited by more than 90% in the presence of 25 nM ponatinib (according to data from Hnatiuk et al.[S1]).

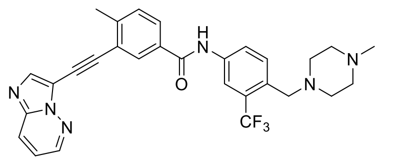

1

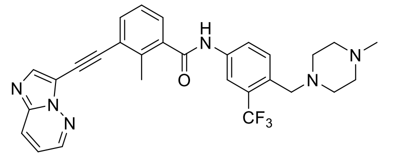

2

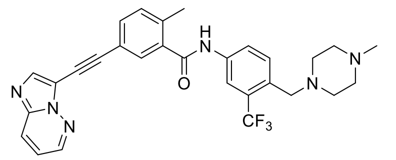

3

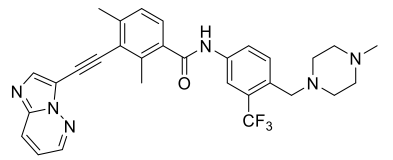

4

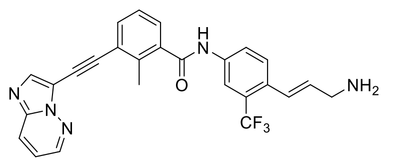

5

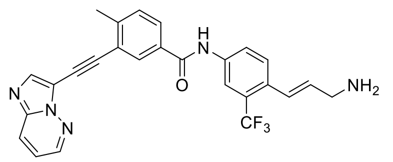

6

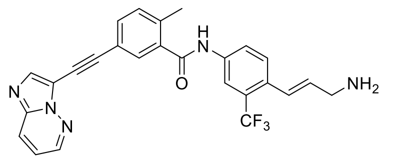

7

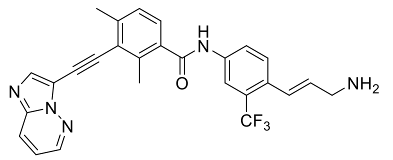

8

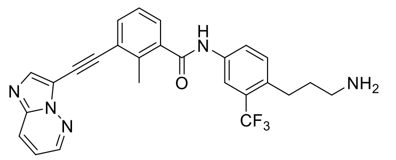

9

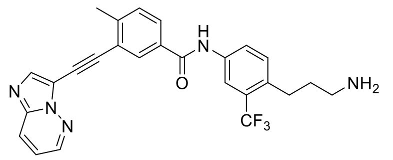

10

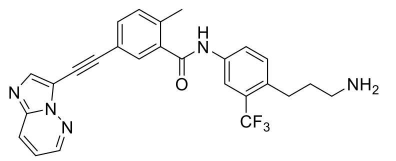

11

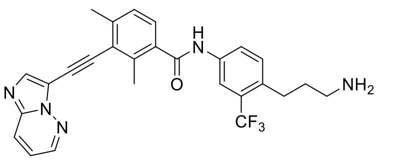

12

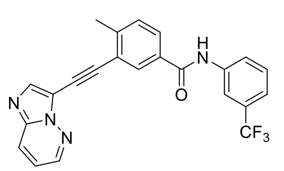

1S

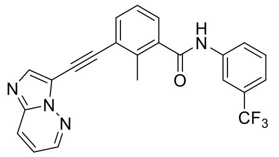

2S

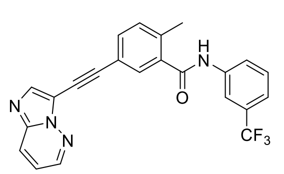

3S

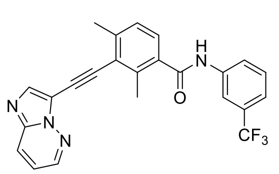

4S

Figure S2. Structures of all target compounds.

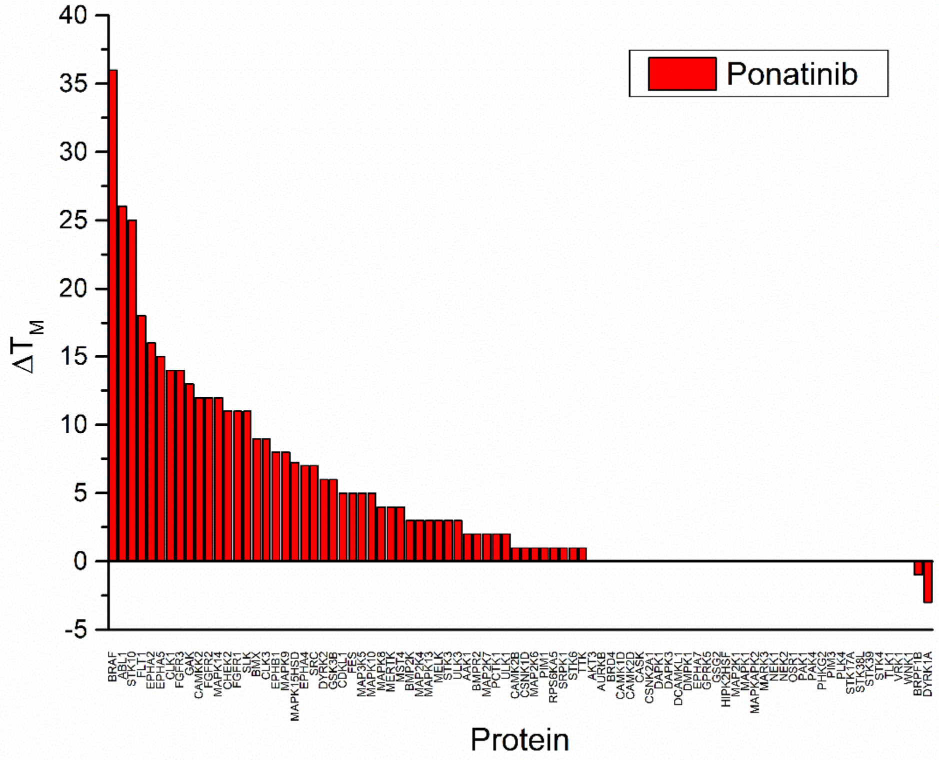
Ponatinib
ΔT_M
40
35
30
25
20
15
10
5
0
-5
Protein

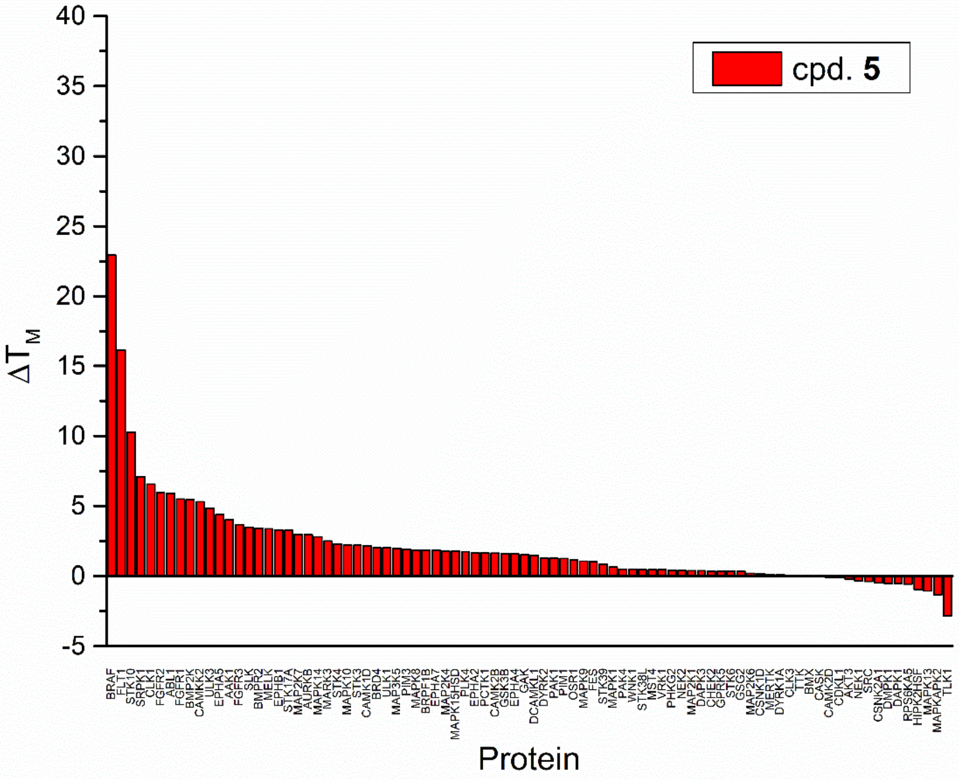
cpd. **5**
ΔT_M
40
35
30
25
20
15
10
5
0
-5
Protein

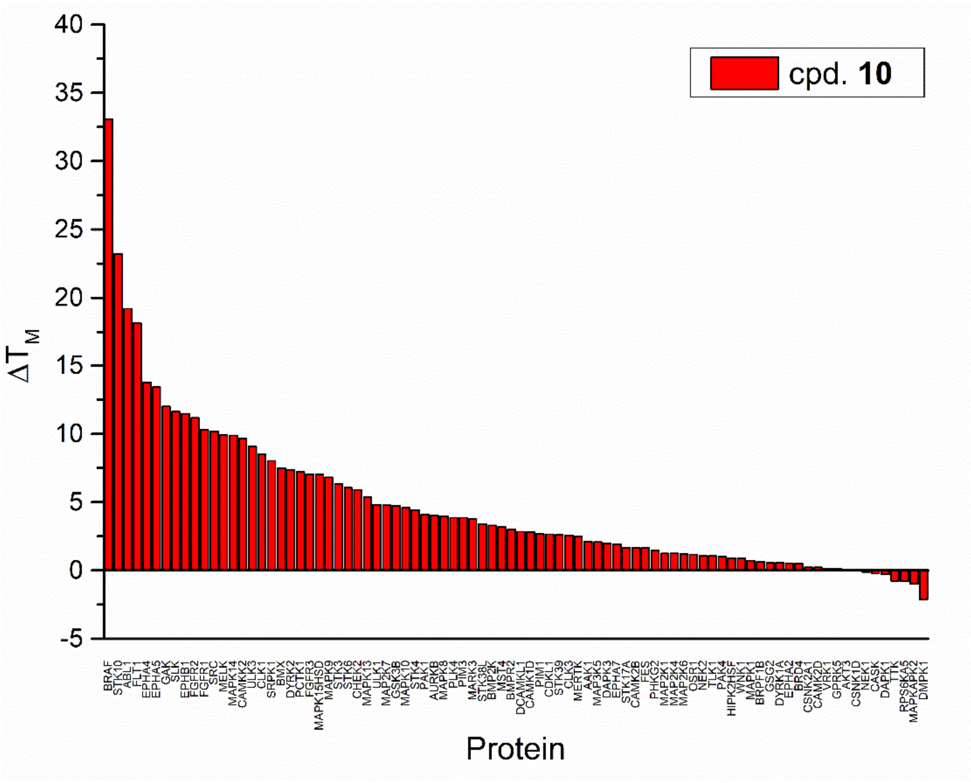

Figure S3. DSF results with compounds **5**, **10** and ponatinib. The ΔTm values are arranged in descending order.

Table S1. Yields for the Sonogashira coupling towards the target structures **1-4** and protected intermediates **5a-12a**.

Cpd. No.	X	Y	Yield
**1**	4-Methyl	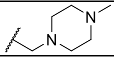	23%
**2**	2-Methyl	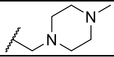	56%
**3**	6-Methyl	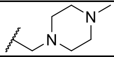	75%
**4**	2,4-Dimethyl	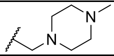	-
**5a**	2-Methyl	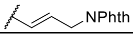	-
**6a**	4-Methyl	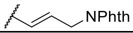	21%
**7a**	6-Methyl	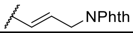	46%
**8a**	2,4-Dimethyl	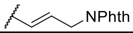	-
**9a**	2-Methyl	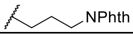	32%
**10a**	4-Methyl	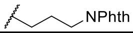	-
**11a**	6-Methyl	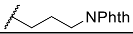	47%
**12a**	2,4-Dimethyl	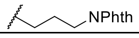	-

Table S2. Yields for the amide coupling towards the target structures **1-4** and protected intermediates **5a-12a**.

Cpd. No.	X	Y	Yield
**1**	4-Methyl	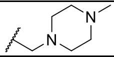	75%
**2**	2-Methyl	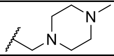	58%
**3**	6-Methyl	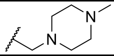	44%
**4**	2,4-Dimethyl	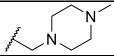	70%
**5a**	2-Methyl	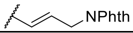	83%
**6a**	4-Methyl	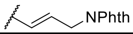	76%
**7a**	6-Methyl	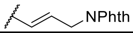	86%
**8a**	2,4-Dimethyl	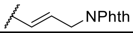	75%
**9a**	2-Methyl	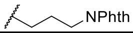	76%
**10a**	4-Methyl	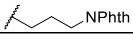	68%
**11a**	6-Methyl	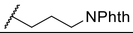	64%
**12a**	2,4-Dimethyl	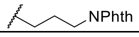	84%

Table S3. Yields for the deprotection (hydrazinolysis) towards the target compounds **5-12**.

Cpd. No.	X	Y	Yield
**5**	2-Methyl	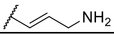	87%
**6**	4-Methyl	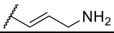	92%
**7**	6-Methyl	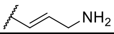	76%
**8**	2,4-Dimethyl	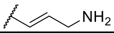	89%
**9**	2-Methyl	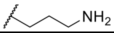	91%
**10**	4-Methyl	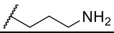	93%
**11**	6-Methyl	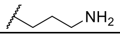	85%
**12**	2,4-Dimethyl	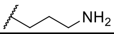	92%

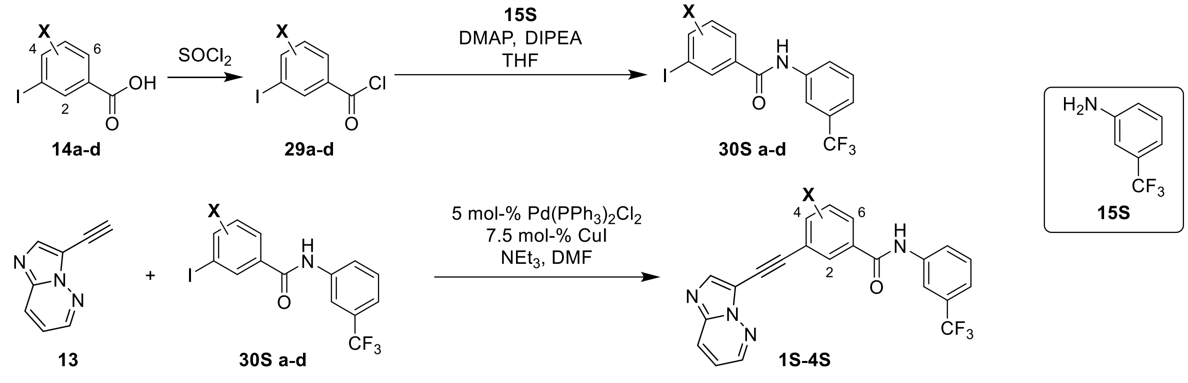

X = 2-, 4- or 6-methyl or 2,4-dimethyl

Scheme S1. Additional syntheses: Synthesis of truncated analogues **1S-4S** (also see Table S4 below).

Table S4. Yields for the synthesis of intermediates **30S a-d** and the Sonogashira reaction towards the truncated target compounds **1S-4S**.

Cpd. No.	X	Yield
**1S**	2-Methyl	98%
**2S**	4-Methyl	98%
**3S**	6-Methyl	88%
**4S**	2,4-Dimethyl	40%
**30Sa**	2-Methyl	75%
**30Sb**	4-Methyl	64%
**30Sc**	6-Methyl	55%
**30Sd**	2,4-Dimethyl	68%

General Procedure (**SA**) for the Synthesis of Intermediates **30S a-d**

The benzoic acids **14a-d** (3.80 mmol, 1.0 eq.) were heated under reflux in $SOCl_2$ (3.8 mL) for 1 h. The mixture was evaporated in vacuo, the residue was dissolved in THF (3.0 mL) and added dropwise to a solution of **15S** (3.88 mmol, 1.02 eq.), DMAP (0.200 mmol, 5 mol-%) and DIPEA (4.60 mmol, 1.2 eq.) in THF (7.0 mL). The mixture was stirred at rt for 22 h, quenched with water and extracted with EtOAc. The combined organics were washed with brine and dried over Na_2SO_4. The mixture was filtered and evaporated in vacuo. Flash chromatography (CH_2Cl_2/MeOH 95:5) gave the desired intermediates **30S a-d**.

General Procedure (**SB**) for the Synthesis of truncated analogues **1S-4S**

One equivalent of the intermediates **30S a-d** (0.500 mmol, 1.0 eq.), 3-ethynylimidazo[1,2-*b*]pyridazine **13** (0.600 mmol, 1.2 eq.), $Pd(PPh_3)_2Cl_2$ (25 μmol, 5 mol-%) and CuI (25 μmol, 5 mol-%) were dissolved in DMF (5 mL). To this mixture, NEt_3 (1.00 mmol, 2.0 eq.) was added. The reaction mixture was heated at reflux for 16 h. After cooling to rt, the mixture was filtered, washed with brine (3 x 25 mL) and evaporated under reduced pressure. Flash chromatography (CH_2Cl_2/MeOH 99:1) gave the desired products **1S-4S**.

3-(Imidazo[1,2-*b*]pyridazin-3-ylethynyl)-2-methyl-*N*-(3-(trifluoromethyl)phenyl)benzamide (**1S**)

Synthesized according to General Procedure SB, Yellow powder; 206 mg, 0.490 mmol, 98% yield. R_f = 0.07 (CH_2Cl_2/MeOH 99:1); ^{1}H NMR (500 MHz, DMSO-d_6): δ [ppm] = 10.77 (s, 1H, NH), 8.72-8.71 (m, 1H, H-6'), 8.28-8.26 (m, 1H, H-2'''), 8.26-8.24 (m, 1H, H-8'), 8.23-8.22 (m, 1H, H-2'), 7.95-7.94 (m, 1H, H-6), 7.73-7.71 (m, 1H, H-6'''), 7.62-7.59 (m, 1H, H-5'''), 7.58-7.56 (m, 1H, H-4), 7.47-7.46 (m, 1H, H-4'''), 7.44-7.41 (m, 1H, H-5), 7.39-7.37 (m, 1H, H-7'), 2.62 (s, 3H, CH_3); ^{13}C NMR (126 MHz, DMSO-d_6): δ [ppm] = 167.63 (C=O), 145.03 (C-6'), 139.82 (C-9'), 139.62 (C-1), 138.19 (C-2'), 137.76 (C-1'''), 136.74 (C-3), 132.71 (C-6'''), 130.01 (C-5'''), 129.48 (q, $^2J_{CF}$ = 31.6 Hz, C-3'''), 127.88 (C-4), 126.18 (C-5), 126.06 (C-8'), 124.10 (q, $^1J_{CF}$ = 272.2 Hz, CF_3), 123.17 (C-2), 122.19 (C-6), 120.05 (q, $^3J_{CF}$ = 3.4 Hz, C-4'''), 119.02 (C-7'), 115.64 (q, $^3J_{CF}$ = 3.7 Hz, C-2'''), 111.72 (C-3'), 96.61 (C-1''), 81.12 (C-2''), 17.73 (CH_3); ^{19}F-NMR (376 MHz, $CDCl_3$): δ [ppm] = -61.31; MS (ESI$^+$): m/z = 420.94 [M+H]$^+$; HRMS (ESI): *m/z* calcd for $C_{23}H_{16}F_3N_4O^+$: 421.1271 [M+H]$^+$; found: 421.1265.

3-(Imidazo[1,2-*b*]pyridazin-3-ylethynyl)-4-methyl-*N*-(3-(trifluoromethyl)phenyl)benzamide (**2S**)

Synthesized according to General Procedure SB, Yellow powder; 186 mg, 0.440 mmol, 88% yield. R_f = 0.07 (CH_2Cl_2/MeOH 99:1); ^{1}H NMR (500 MHz, DMSO-d_6): δ [ppm] = 10.60 (s, 1H, NH), 8.73-8.72 (m, 1H, H-6'), 8.27-8.25 (m, 1H, H-8'), 8.26-8.25 (m, 1H, H-2'''), 8.24-8.23 (m, 1H, H-2'), 8.23-8.21 (m, 1H, H-2), 8.10-8.08 (m, 1H, H-6'''), 7.96-7.94 (m, 1H, H-6), 7.62-7.59 (m, 1H, H-5'''), 7.55-7.54 (m, 1H, H-5), 7.46-7.45 (m, 1H, H-4'''), 7.40-7.37 (m, 1H, H-7'), 2.61 (s, 3H, CH_3); ^{13}C NMR (126 MHz, DMSO-d_6): δ [ppm] = 164.70 (C=O), 145.03 (C-6'), 143.53 (C-4), 139.87 (C-1), 139.64 (C-9'), 138.23 (C-2'), 132.16 (C-3), 130.17 (C-2), 130.06 (C-5), 129.84 (C-5'''), 129.34 (q, $^2J_{CF}$ = 31.6 Hz, C-3'''), 128.49 (C-6), 126.08 (C-8'), 124.15 (q, $^1J_{CF}$ = 272.2 Hz, CF_3), 123.79 (C-1'''), 121.78 (C-6'''), 119.98 (q, $^3J_{CF}$ = 3.4 Hz, C-4'''), 119.05 (C-7'), 116.41 (q, $^3J_{CF}$ = 4.0 Hz, C-2'''), 111.70 (C-3'), 96.38 (C-1''), 81.14 (C-2''), 20.38 (CH_3); ^{19}F-NMR (376 MHz, $CDCl_3$): δ [ppm] = -61.29; MS (ESI$^+$): m/z = 420.97 [M+H]$^+$; HRMS (ESI): *m/z* calcd for $C_{23}H_{16}F_3N_4O^+$: 421.1271 [M+H]$^+$; found: 421.1267.

5-(Imidazo[1,2-*b*]pyridazin-3-ylethynyl)-2-methyl-*N*-(3-(trifluoromethyl)phenyl)benzamide (**3S**)

Synthesized according to General Procedure SB, Yellow powder; 204 mg, 0.490 mmol, 98% yield. R_f = 0.07 (CH_2Cl_2/MeOH 99:1); ^{1}H NMR (500 MHz, DMSO-d_6): δ [ppm] = 10.74 (s, 1H, NH), 8.70-8.69 (m, 1H, H-6'), 8.27-8.25 (m, 1H, H-2'''), 8.25-8.23 (m, 1H, H-8'), 8.20-8.19 (m, 1H, H-2'), 7.98-7.96 (m, 1H, H-4), 7.77-7.76 (m, 1H, H-6), 7.66-7.64 (m, 1H, H-6'''), 7.62-7.58 (m, 1H, H-5'''), 7.47-7.45 (m, 1H, H-4'''), 7.44-7.42 (m, 1H, H-3), 7.39-7.36 (m, 1H, H-7'), 2.45 (s, 3H, CH_3); ^{13}C NMR (126 MHz, DMSO-d_6): δ [ppm] = 167.00 (C=O), 144.90 (C-6'), 139.86 (C-9'), 139.52 (C-1), 138.26 (C-2'), 137.21 (C-1'''), 136.84 (C-5), 132.39 (C-6'''), 131.45 (C-3), 130.01 (C-6), 129.81 (C-5'''), 129.43 (q, $^2J_{CF}$ = 31.5 Hz, C-3'''), 126.06 (C-8'), 124.13 (q, $^1J_{CF}$ = 271.9 Hz, CF_3), 123.32 (C-4), 120.03 (q, $^3J_{CF}$ = 3.7 Hz, C-4'''), 119.11 (C-2), 118.98 (C-7'), 115.84 (q, $^3J_{CF}$ = 3.9 Hz, C-2'''), 111.70 (C-3'), 97.43 (C-1''), 76.94 (C-2''), 19.44 (CH_3); ^{19}F-NMR (376 MHz, $CDCl_3$): δ [ppm] = -61.30; MS (ESI$^+$): m/z = 420.97 [M+H]$^+$; HRMS (ESI): *m/z* calcd for $C_{23}H_{16}F_3N_4O^+$: 421.1271 [M+H]$^+$; found: 421.1268.

3-(Imidazo[1,2-*b*]pyridazin-3-ylethynyl)-2,4-dimethyl-*N*-(3-(trifluoromethyl)phenyl)benzamide (**4S**)

Synthesized according to General Procedure SB, Yellow powder; 86.8 mg, 0.199 mmol, 40% yield. R_f = 0.07 (CH_2Cl_2/MeOH 99:1); ^{1}H NMR (500 MHz, $CDCl_3$): δ [ppm] = 8.62-8.57 (m, 1H, H-6'), 8.32-8.27 (m, 1H, H-4'), 8.08-8.02 (m, 1H, H-2'), 7.98-7.93 (m, 1H, H-2'''), 7.90-7.83 (m, 1H, H-6'''), 7.81 (bs, 1H, NH), 7.53-7.48 (m, 1H, H-5'''), 7.44-7.41 (m, 1H, H-4'''), 7.40-7.37 (m, 1H, H-5), 7.32-7.28 (m, 1H, H-5'), 7.20-7.16 (m, 1H, H-6), 2.71 (s, 3H, CH_3), 2.60 (s, 3H, CH_3); ^{13}C NMR (126 MHz, $CDCl_3$): δ [ppm] =

167.91 (C=O), 145.16 (C-6‘), 143.38 (C-4), 138.92 (C-2), 138.68 (C-3‘), 138.61 (C-1‘‘‘), 134.31 (C-1), 134.23 (C-2‘), 131.72 (q, 2*J*CF = 32.6 Hz, C-3‘‘‘), 129.86 (C-5‘‘‘), 127.20 (C-6), 127.00 (C-5), 125.20 (C-4‘), 123.95 (q, 1*J*CF = 274.2 Hz, CF3), 123.93 (C-3), 123.04 (C-6‘‘‘), 121.34 (q, 3*J*CF = 3.7 Hz, C-4‘‘‘), 119.93 (C-5‘), 116.74 (q, 3*J*CF = 3.4 Hz, C-2‘‘‘), 114.04 (C-1‘), 96.74 (C-2‘‘), 83.99 (C-1‘‘), 21.62 (CH_3), 18.64 (CH_3); ^{19}F-NMR (376 MHz, $CDCl_3$): δ [ppm] = -61.30; MS (ESI$^+$): m/z = 435.10 [M+H]$^+$; HRMS (ESI): *m/z* calcd for $C_{24}H_{18}F_3N_4O^+$: 435.1427 [M+H]$^+$; found: 435.1462.

3-Iodo-2-methyl-*N*-(3-(trifluoromethyl)phenyl)benzamide (**30S a**)

Synthesized according to General Procedure SA, Yellow powder; 1.16 g, 2.86 mmol, 75% yield. R_f = 0.07 (CH_2Cl_2/MeOH 99:1); ^{1}H NMR (500 MHz, $CDCl_3$): δ [ppm] = 7.93 (bs, 1H, NH), 7.92-7.91 (m, 1H, H-2‘), 7.90-7.88 (m, 1H, H-4), 7.80-7.78 (m, 1H, H-6), 7.49-7.45 (m, 1H, H-5‘), 7.42-7.41 (m, 1H, H-4‘), 7.33-7.32 (m, 1H, H-6‘), 6.91-6.88 (m, 1H, H-6), 2.49 (s, 3H, CH_3); ^{13}C NMR (126 MHz, $CDCl_3$): δ [ppm] = 167.77 (C=O), 141.22 (C-2), 138.71 (C-4), 138.14 (C-1‘), 137.12 (C-1), 131.51 (q, $^2J_{CF}$ = 32.6 Hz, C-3‘), 129.68 (C-5‘), 127.42 (C-5), 126.38 (C-6), 123.72 (q, $^1J_{CF}$ = 272.3 Hz, CF_3), 122.94 (C-6‘), 121.35 (q, $^3J_{CF}$ = 3.7 Hz, C-4‘), 116.64 (q, $^3J_{CF}$ = 3.7 Hz, C-2‘), 103.67 (C-3), 25.63 (CH_3).; ^{19}F-NMR (376 MHz, $CDCl_3$): δ [ppm] = -62.73; MS (ESI$^+$): m/z = 405.83 [M+H]$^+$; HRMS (ESI): *m/z* calcd for $C_{15}H_{12}F_3INO^+$: 405.9910 [M+H]$^+$; found: 405.9896.

3-Iodo-4-methyl-*N*-(3-(trifluoromethyl)phenyl)benzamide (**30S b**)

Synthesized according to General Procedure SA, Yellow powder; 980 mg, 2.42 mmol, 64% yield. R_f = 0.07 (CH_2Cl_2/MeOH 99:1); ^{1}H NMR (500 MHz, $CDCl_3$): δ [ppm] = 8.32 (bs, 1H, NH), 8.24-8.23 (m, 1H, H-2), 7.93-7.90 (m, 1H, H-2’), 7.85-7.83 (m, 1H, H-6), 7.70-7.68 (m, 1H, H-6’), 7.45-7.42 (m, 1H, H-5’), 7.39-7.37 (m, 1H, H-4’), 7.25-7.24 (m, 1H, H-5), 2.45 (s, 3H, CH_3); ^{13}C NMR (126 MHz, $CDCl_3$): δ [ppm] = 164.63 (C=O), 146.16 (C-4), 138.39 (C-1’), 137.67 (C-2), 133.53 (C-1), 131.53 (q, $^2J_{CF}$ = 32.6 Hz, C-3’), 129.89 (C-5), 129.72 (C-5’), 127.06 (C-6), 123.92 (q, $^1J_{CF}$ = 272.3 Hz, CF_3), 123.60 (C-6’), 121.36 (q, $^3J_{CF}$ = 3.7 Hz, C-4’), 117.25 (q, $^3J_{CF}$ = 3.9 Hz, C-2’), 101.16 (C-3), 28.33 (CH_3).; ^{19}F-NMR (376 MHz, $CDCl_3$): δ [ppm] = -62.73; MS (ESI$^+$): m/z = 405.84 [M+H]$^+$; HRMS (ESI): *m/z* calcd for $C_{15}H_{12}F_3INO^+$: 405.9910 [M+H]$^+$; found: 405.9898.

5-Iodo-2-methyl-*N*-(3-(trifluoromethyl)phenyl)benzamide (**30S c**)

Synthesized according to General Procedure SA, Yellow powder; 850 mg, 2.10 mmol, 55% yield. R_f = 0.07 (CH_2Cl_2/MeOH 99:1); ^{1}H NMR (500 MHz, $CDCl_3$): δ [ppm] = 7.91 (bs, 1H, NH), 7.83-7.81 (m, 1H, H-6), 7.79-7.75 (m, 1H, H-2’), 7.68-7.67 (m, 2H, H-4/H-6’), 7.51-7.48 (m, 1H, H-4’), 7.43-7.41 (m, 1H, H-5’), 7.03-7.01 (m, 1H, H-3), 2.43 (s, 3H, CH_3); ^{13}C NMR (126 MHz, $CDCl_3$): δ [ppm] = 166.52 (C=O), 138.30 (C-6), 139.61 (C-1’), 137.93 (C-4), 136.43 (C-2), 135.30 (C-1), 133.41 (C-3), 131.74 (q, $^2J_{CF}$ = 32.7 Hz, C-3’), 129.87 (C-5’), 123.12 (C-6’), 122.83 (CF_3), 121.52 (q, $^3J_{CF}$ = 3.6 Hz, C-4’), 116.83 (q, $^3J_{CF}$ = 3.4 Hz, C-2’), 90.36 (C-5), 19.59 (CH_3).; ^{19}F-NMR (376 MHz, $CDCl_3$): δ [ppm] = -62.74; MS (ESI$^+$): m/z = 405.80 [M+H]$^+$; HRMS (ESI): *m/z* calcd for $C_{15}H_{12}F_3INO^+$: 405.9910 [M+H]$^+$; found: 405.9896.

3-Iodo-2,4-dimethyl-*N*-(3-(trifluoromethyl)phenyl)benzamide (**30S d**)

Synthesized according to General Procedure SA, Yellow powder; 1.03 g, 2.46 mmol, 68% yield. R_f = 0.07 (CH_2Cl_2/MeOH 99:1); ^{1}H NMR (500 MHz, $CDCl_3$): δ [ppm] = 7.94-7.89 (m, 1H, H-2‘), 7.85-7.78 (m, 1H, H-6), 7.58 (bs, 1H, NH), 7.52-7.46 (m, 1H, H-5‘), 7.44-7.40 (m, 1H, H-4‘), 7.33-7.28 (m, 1H, H-6‘), 7.17-7.12 (m, 1H, H-5), 2.60 (s, 3H, CH_3), 2.52 (s, 3H, CH_3); ^{13}C NMR (126 MHz, $CDCl_3$): δ [ppm] = 167.96 (C=O), 145.15 (C-2), 141.40 (C-4), 139.66 (C-1‘), 138.41 (C-1), 134.65 (C-5‘), 131.74 (q, $^2J_{CF}$ = 32.7 Hz, C-3’), 127.13 (C-5), 127.10 (C-6), 126.05 (C-6‘), 124.37 (q, $^1J_{CF}$ = 293.1 Hz, CF_3), 121.41 (q, $^3J_{CF}$ = 3.6 Hz, C-4’), 116.62 (q, $^3J_{CF}$ = 3.4 Hz, C-2’), 89.35 (C-3), 30.58 (CH_3), 27.22 (CH_3).; ^{19}F-NMR (376 MHz, $CDCl_3$): δ [ppm] = -62.73; MS (ESI$^+$): m/z = 419.99 $[M+H]^+$; HRMS (ESI): *m/z* calcd for $C_{16}H_{14}F_3INO^+$: 420.0067 $[M+H]^+$.

Table S5. ΔTm values of the DSF kinase profiling with **5**, **10**, ponatinib and reference inhibitors.

Protein	10 [°C]	5 [°C]	1 (Ponatinib) [°C]	Reference	Reference Shift [°C]	Reference [nM]
AAK1	2.1	4.0	2.0	Staurosporine	15.3	1.2
ABL1	19.2	5.9	26.0	Staurosporine	8.6	60.0
AKT3	0.1	-0.2	0.0	Staurosporine	7,0	5.0
AurA	6.1	0.4	1.0	Staurosporine	16.5	1.5
AurB	4.0	3.0	0.0	Staurosporine	8.0	5.0
BIKE	3.3	5.5	3.0	Staurosporine	18.4	1.0
BMPR2	3.0	3.4	2.0	Staurosporine	3.0	670.0
BMX	7.5	0.0	9.0	Staurosporine	6.8	170.0
BRAF	33.1	22.9	36.0	Dabrafenib	26.6	0.7
BRD4	0.5	2.0	0.0	JQ1	7.0	60.0
BRPF1	0.7	1.8	-1.0	GSK6853	14.0	20.0
CAMK1D	2.8	2.2	0.0	Staurosporine	8.9	0.4
CAMK2B	1.7	1.6	1.0	Staurosporine	11.3	0.1
CAMK2D	0.2	-0.1	0.0	Staurosporine	15.8	0.5
CAMK4	1.8	n.d.		Staurosporine	8.2	141.0
CAMKK2	9.7	5.3	12.0	Staurosporine	23.5	0.1
CASK	-0.2	0.0	0.0	Staurosporine	5.2	19.0
CDKL1	2.7	-0.1	5.0	CEP-32496	6.6	<0.1
CHK2	5.9	0.4	11.0	Staurosporine	17.1	0.1
CK1d	0.1	0.2	1.0	PF-670462	9.0	8.0
CK2a1	0.2	-0.5	0.0	Similtasertib	14.6	1.0
CLK1	8.5	6.6	14.0	Staurosporine	15.7	4.0
CLK3	2.6	0.1	9.0	CLK-T3	15.0	110.0
CSNK1EA	-1.0	0.0		PF-670462	8.0	14.0
DAPK1	-0.3	-0.5	0.0	Staurosporine	9.0	4.0
DAPK3	2.0	0.4	0.0	Staurosporine	16.2	1.0
DCAMKL1	2.8	1.5	0.0	Staurosporine	11.4	120.0
DMPK1	-2.1	-0.5	0.0	Staurosporine	9.3	22.7
DRAK1	1.7	3.3	0.0	Staurosporine	7.7	14.0
DYRK1A	0.6	0.1	-3.0	Staurosporine	9.9	4.0
DYRK2	7.4	1.3	6.0	Staurosporine	7.3	280.0
EPHA2	0.5	1.7	16.0	Staurosporine	7.1	53.0
EPHA4	13.8	1.6	7.0	Staurosporine	5.3	7.4
EPHA5	13.5	4.4	15.0	Staurosporine	6.9	19.0
EPHA7	1.9	1.8	0.0	Staurosporine	10.5	30.4
EPHB1	11.5	3.3	8.0	Staurosporine	6.4	25.0
Erk2	0.7	0.7	0.0	GDC-0994	7.5	1.0
ERK7	7.1	1.8	7.3	Staurosporine	14.0	5.5
FES	1.7	1.0	5.0	Staurosporine	7.5	1.7
FGFR1	10.3	5.5	11.0	Staurosporine	5.7	3.2
FGFR2	11.2	6.0	12.0	Staurosporine	8.3	3.3
FGFR3	7.1	3.7	14.0	Staurosporine	11.7	25.0
FLT1	18.1	16.2	18.0	Staurosporine	12.0	11.0

GAK	12.0	1.5	13.0	Staurosporine	8.8	17.0
GPRK5	0.2	0.4	0.0	Staurosporine	5.9	150.0
GSK3B	4.7	1.6	6.0	Staurosporine	9.0	4.3
Haspin	0.6	0.4	0.0	Staurosporine	8.9	50.0
HIPK2	0.9	-1.0	0.0	GW779439X	11.6	<0.1
JNK1	4.0	1.8	4.0	Staurosporine	7.7	220.0
JNK2	6.8	1.1	8.0	SBI-0069279	8.6	<0.1
JNK3	4.6	2.3	5.0	CEP-32496	8.8	<0.1
LOK	23.2	10.3	25.0	Staurosporine	23.5	<0.1
MAP2K1	1.3	0.4	0.0	Staurosporine	3.2	24.0
MAP2K6	1.2	0.2	1.0	Staurosporine	11.3	1.0
MAP2K7	4.8	3.0	2.0	Staurosporine	7.0	440.0
MAP3K5	2.1	2.0	5.0	Staurosporine	15.5	24.0
MAPKAPK2	-1.0	-1.3	0.0	Staurosporine	4.0	0.0
MARK3	3.8	2.5	0.0	Staurosporine	18.0	0.5
MEK4	1.2	1.8	3.0	Staurosporine	10.5	1.0
MELK	9.9	3.4	3.0	Staurosporine	13.2	0.7
MER	2.5	0.1	4.0	Staurosporine	5.5	6.4
MSK1_b	-0.8	-0.5	1.0	Staurosporine	15.6	5.0
MSSK1	9.7	5.0		Staurosporine	3.6	1750.0
MST1	4.4	2.3	0.0	Staurosporine	15.5	1.0
MST2	2.6	0.8	0.0	Staurosporine	13.4	0.2
MST2	6.3	2.2	3.0	Staurosporine	13.4	0.2
MST4	3.2	0.4	4.0	Staurosporine	6.0	6.7
NDR2	3.4	0.5	0.0	Staurosporine	11.6	1.4
NEK2	1.1	0.4	0.0	VN2010	9.7	<0.1
OSR1	1.2	1.2	0.0	Staurosporine	5.7	91.0
p38a	9.9	2.8	12.0	Doramapimod	19.8	1.0
p38d	5.4	-1.0	3.0	Doramapimod	0.0	1.0
PAK1	4.1	1.3	0.0	Staurosporine	7.3	0.3
PAK4	1.0	0.5	0.0	Staurosporine	12.2	6.3
PCTAIRE1	7.2	1.7	2.0	Staurosporine	9.0	14.0
PHKG2	1.5	0.4	0.0	Staurosporine	21.2	0.1
PIM1	2.7	1.3	1.0	Staurosporine	11.9	3.0
PIM3	3.9	1.9	0.0	Staurosporine	19.0	0.1
PLK4	3.9	1.8	0.0	Staurosporine	18.0	4.0
SLK	11.7	3.5	11.0	Staurosporine	16.9	3.9
SRC	10.2	-0.4	7.0	Staurosporine	5.0	2.3
SRPK1	8.1	7.1	1.0	Staurosporine	7.0	120.0
SRPK2	4.8	2.7		Staurosporine	n.d	420.0
TLK1	1.1	-2.9	0.0	Staurosporine	8.9	44.0
TTK	-0.7	0.0	1.0	Staurosporine	9.0	61.0
ULK1	4.8	2.0	2.0	Staurosporine	12.0	<0.1
ULK3	9.1	4.8	3.0	Staurosporine	17.3	1.5
VRK1	0.2	0.4	0.0	MR1	4.5	0.0
WNK1	0.9	0.5	0.0	MRIA5	4.6	0.0

Table S6. Binding affinity constants of the truncated compounds without basic moiety towards ROR1 as determined by MST.

**Cpd. No.**	K_d [µM]
**1S**	2.7
**2S**	55
**3S**	60
**4S**	15

Table S7. Comparison of ΔTm values and inhibition strengths.

Target kinase of cpd. **5**	[a]% inhibition at 23.5 nM Ponatinib	ΔTm (Ponatinib) [°C]	ΔTm (cpd. **5**) [°C]
B-Raf	85	36	22,9
Flt-1	67	18	16,2
STK10	99,95	25	10,3
SRPK1	2	1	7,1

[a]Values taken from ref. [S1]

MST curves

ROR1-Cpd. **1** (ponatinib)

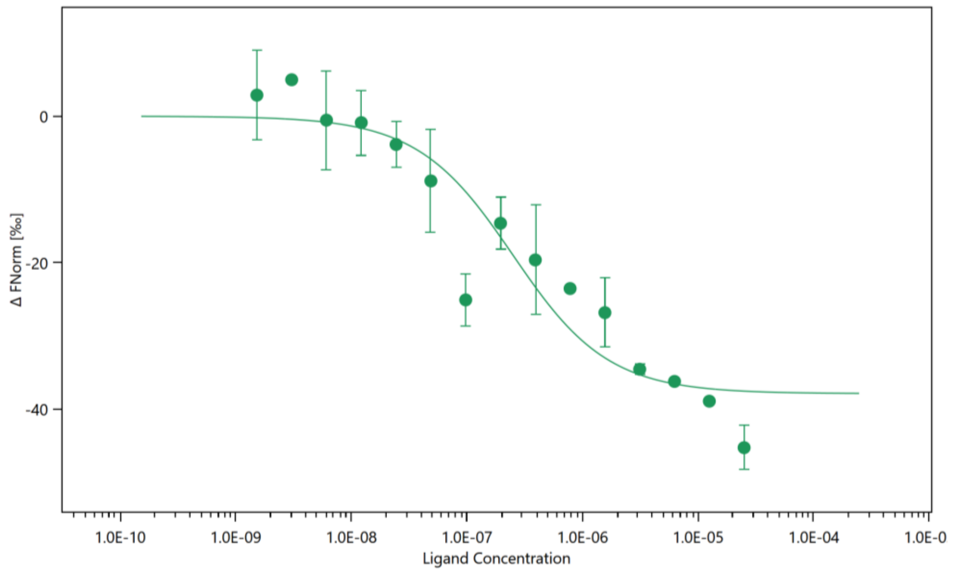

ROR1-Cpd. **2**

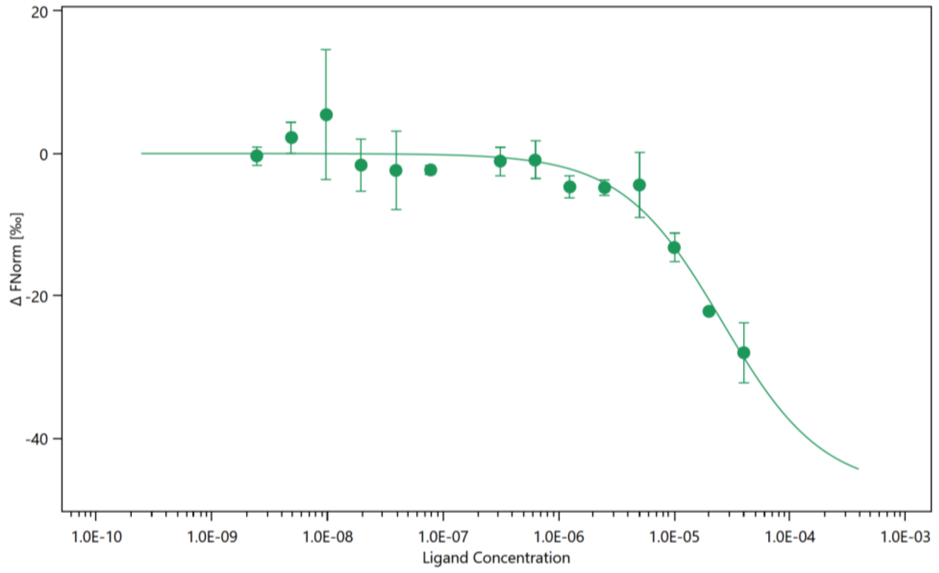

ROR1-Cpd. **3**

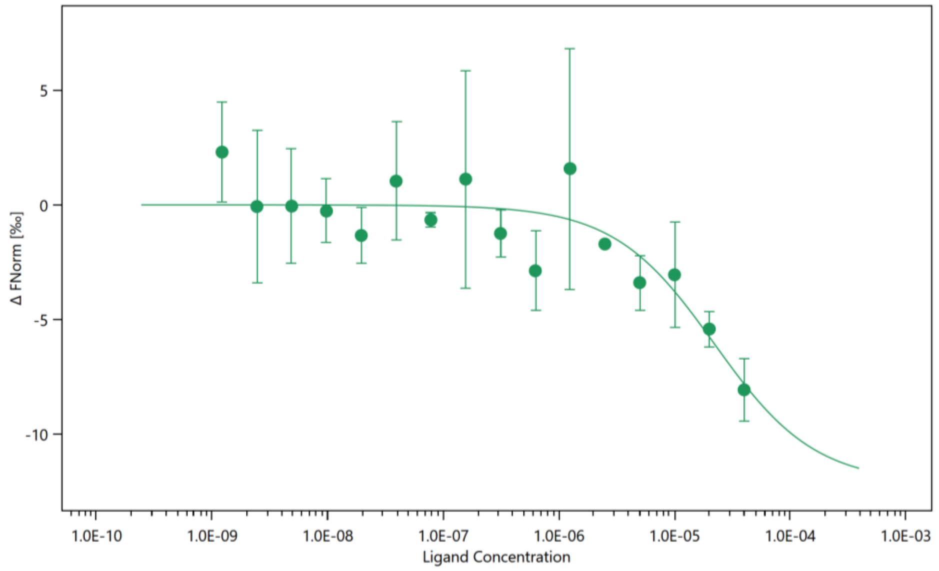

ROR1-Cpd. **4**

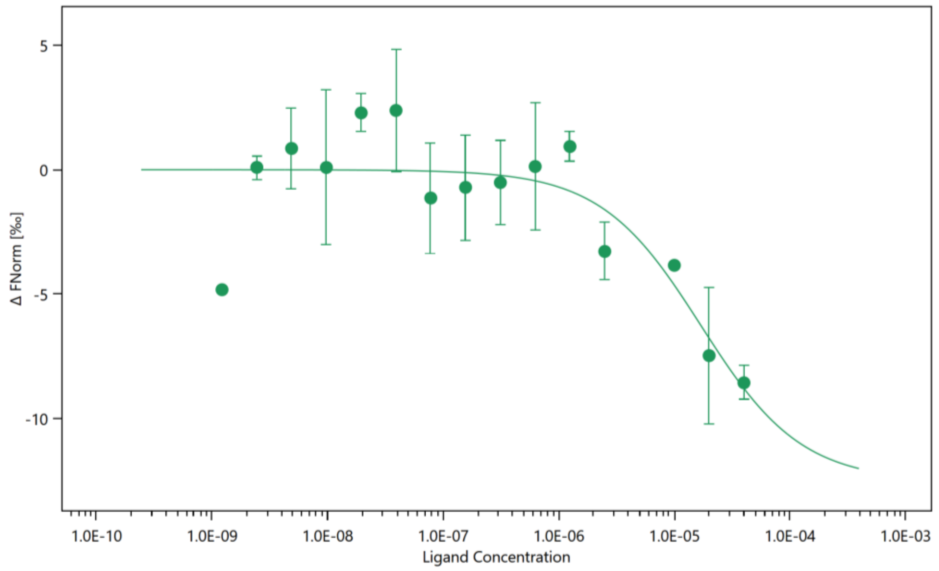

ROR1-Cpd. **5**

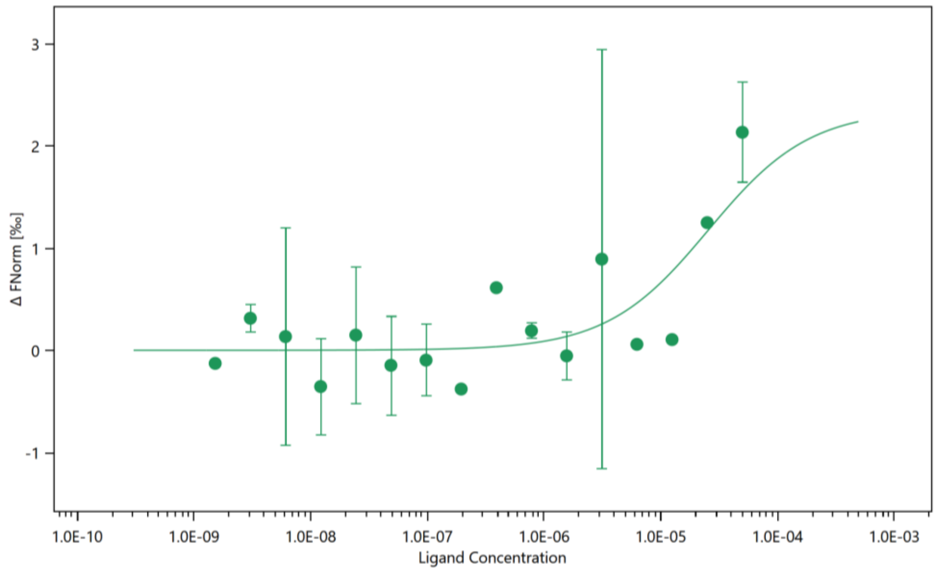

ROR1-Cpd. **6**

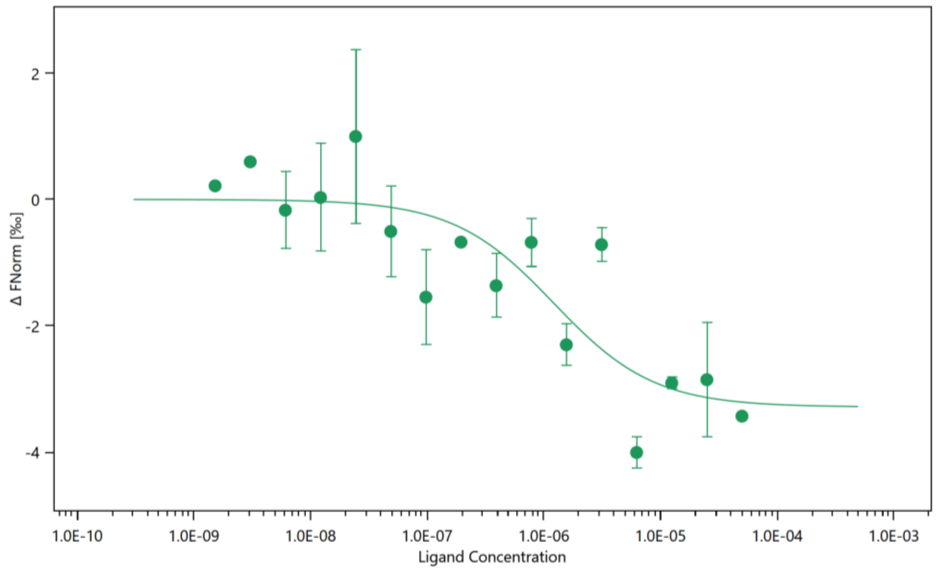

ROR1-Cpd. **7**

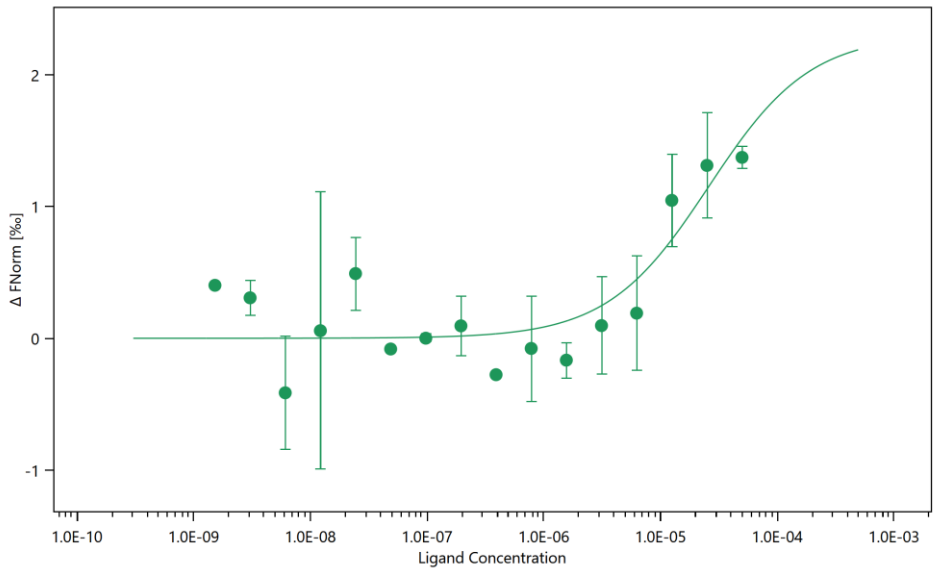

ROR1-Cpd. **8**

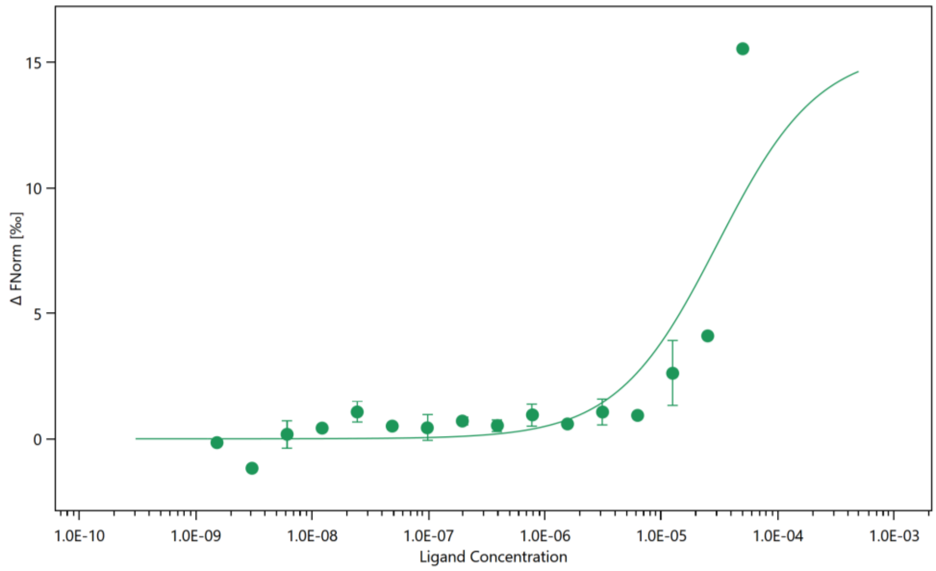

ROR1-Cpd. **9**

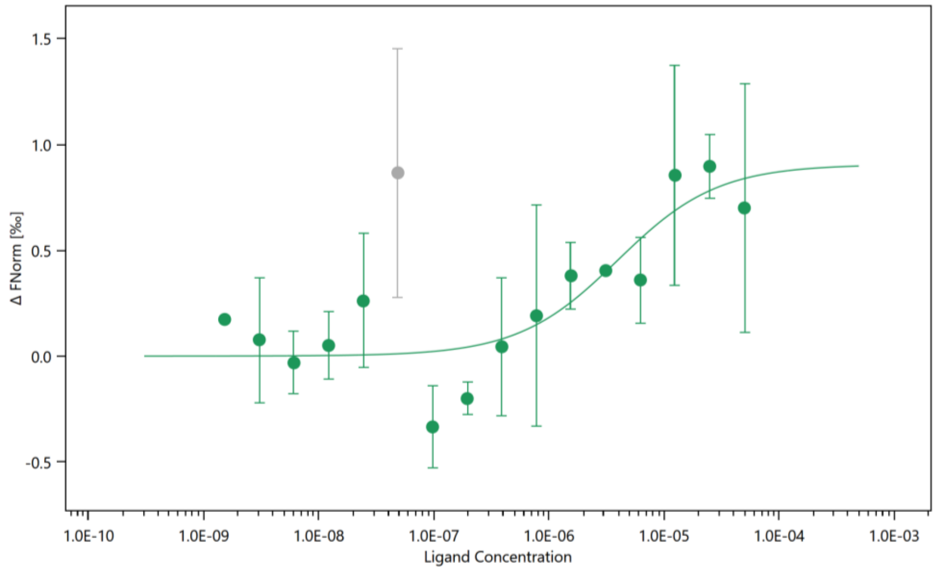

ROR1-Cpd. **10**

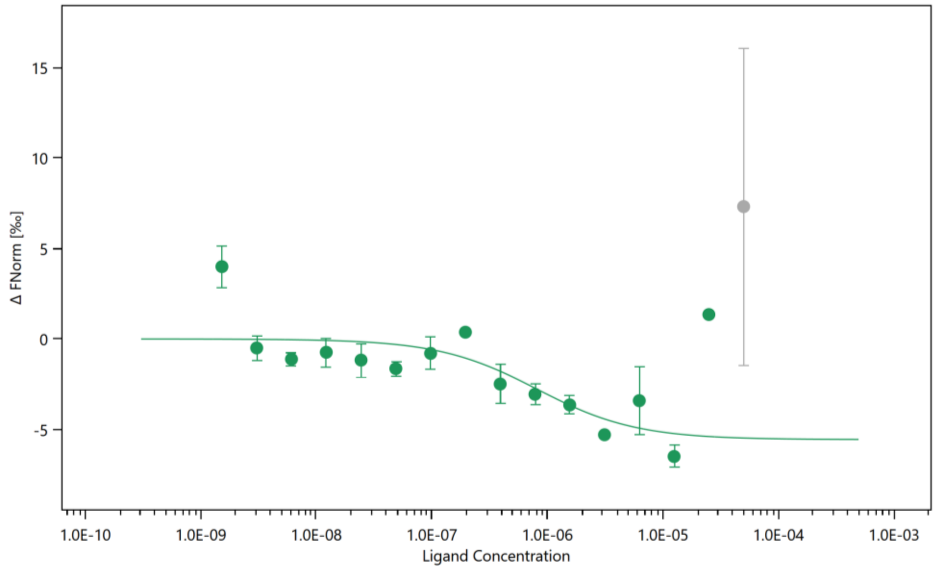

ROR1-Cpd. **11**

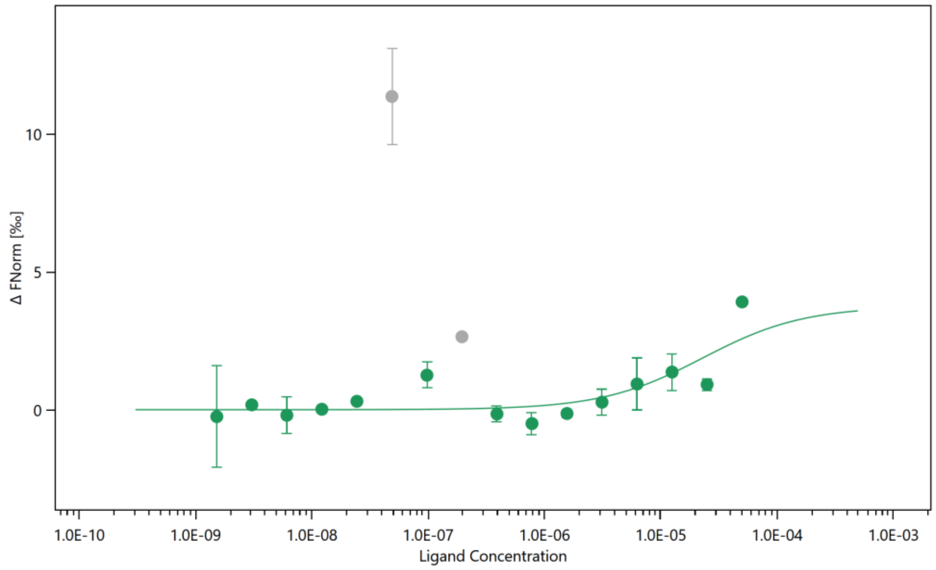

ROR1-Cpd. **12**

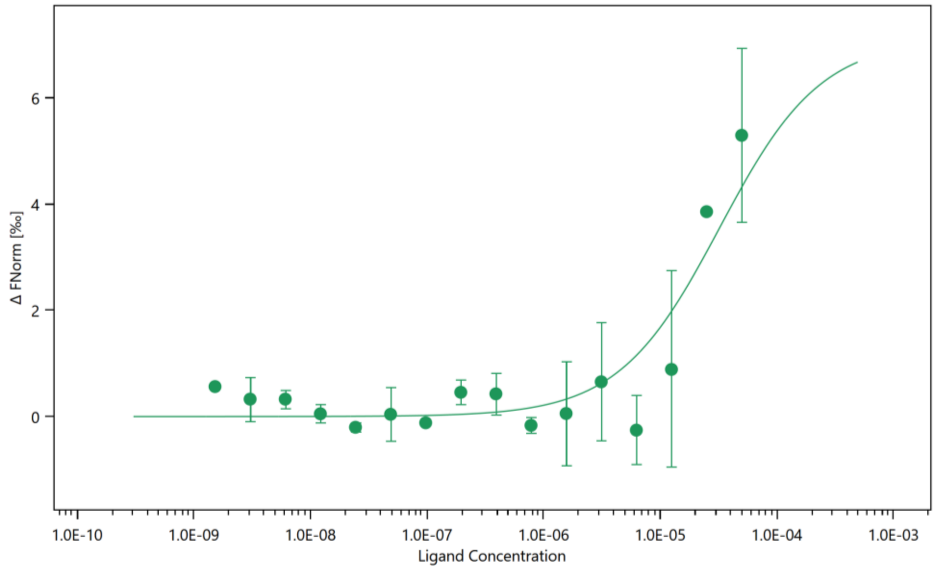

ROR1-Cpd. **1S**

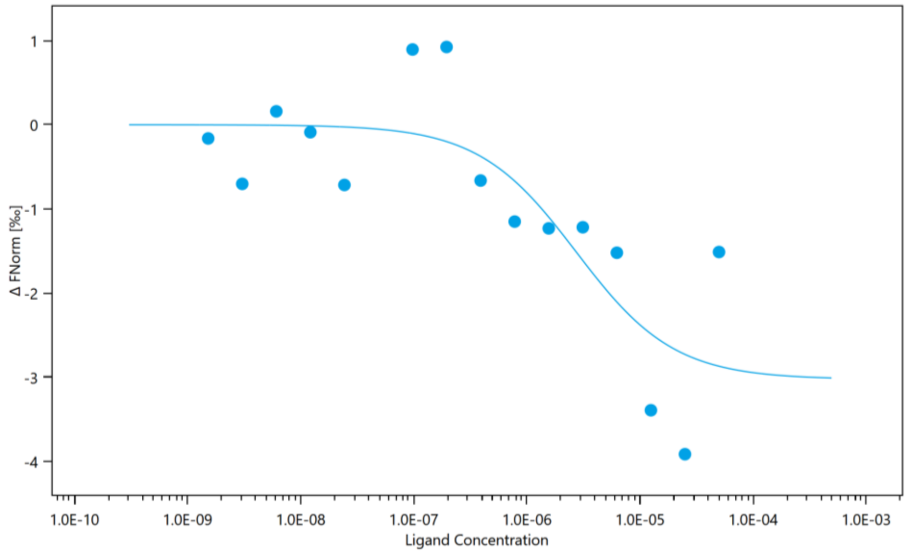

ROR1-Cpd. **2S**

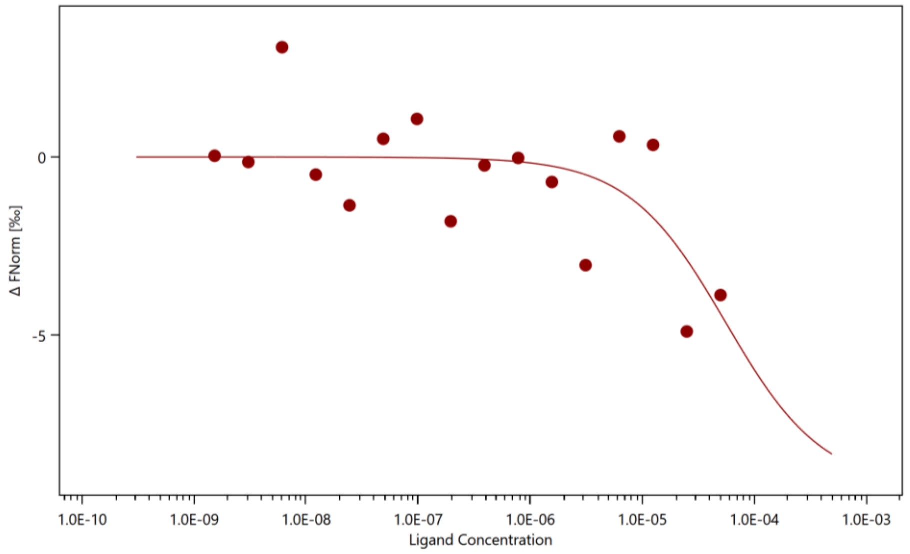

ROR1-Cpd. **3S**

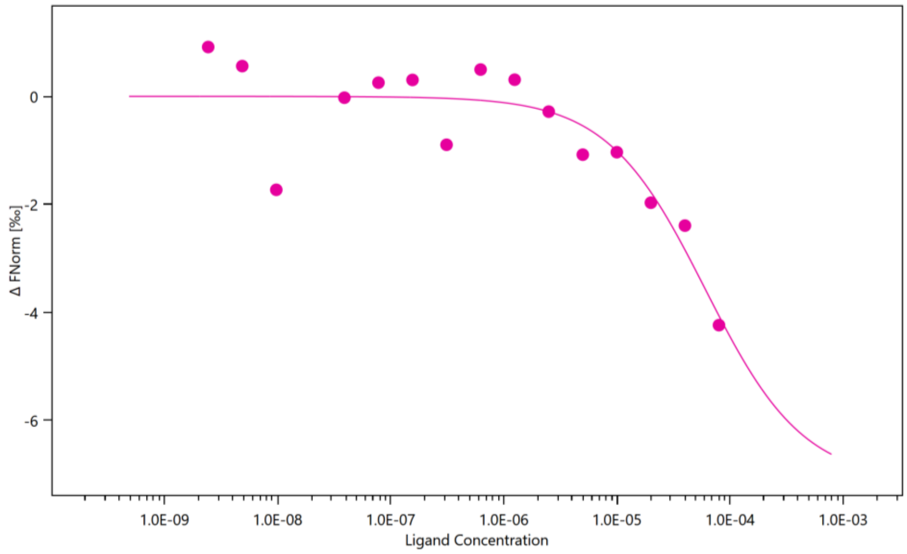

ROR1-Cpd. **4S**

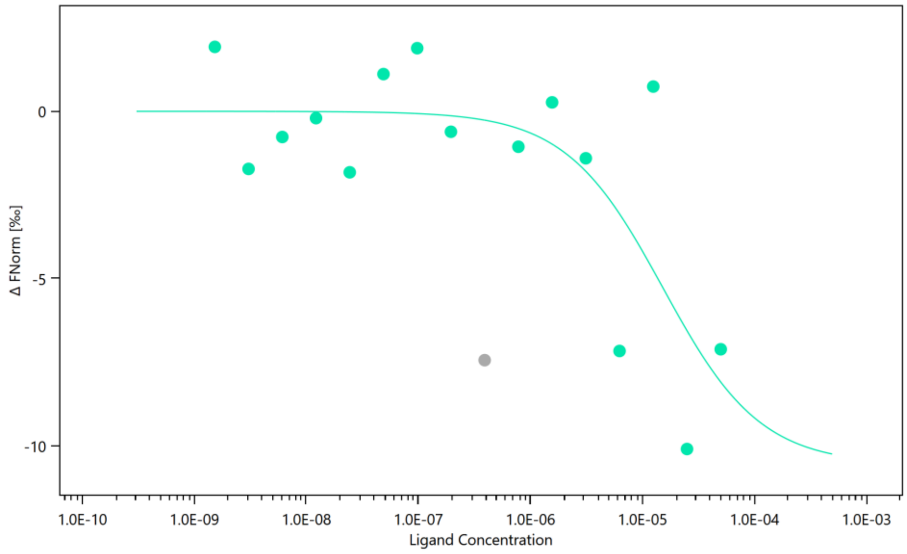

Figure S4. MST binding curves of the target compounds with ROR1.

NMR spectra

3-(Imidazo[1,2-*b*]pyridazin-3-ylethynyl)-4-methyl-*N*-(4-((4-methylpiperazin-1-yl)methyl)-3-(trifluoromethyl)phenyl)benzamide (**1**)

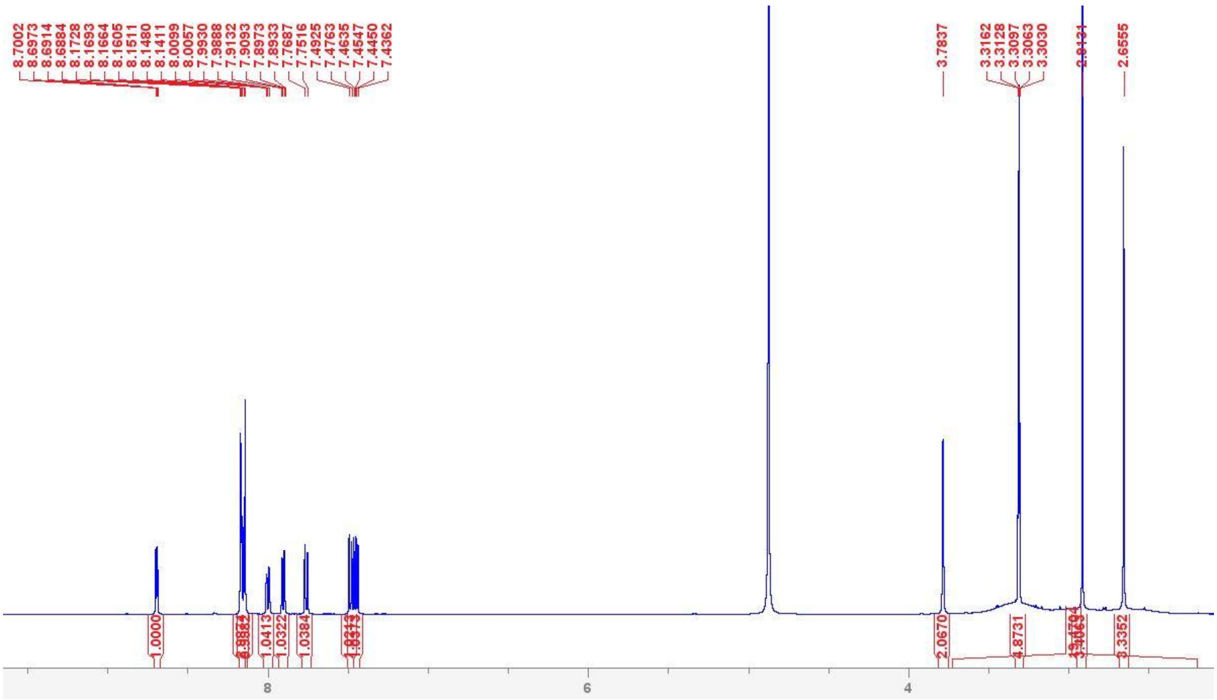

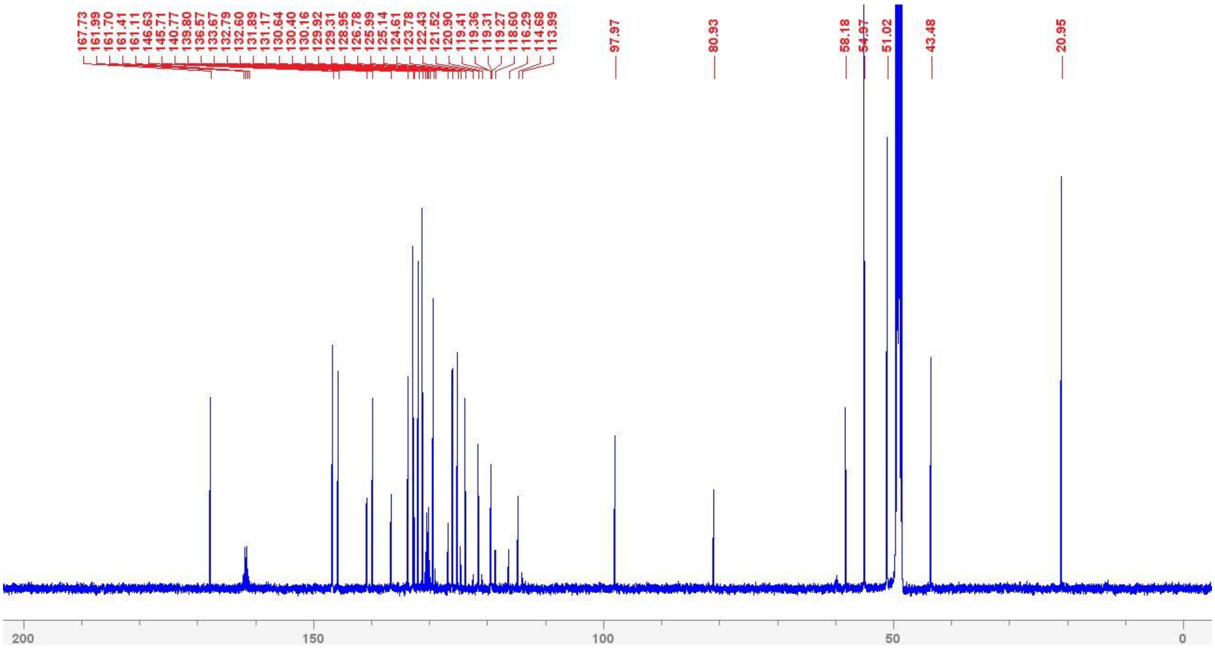

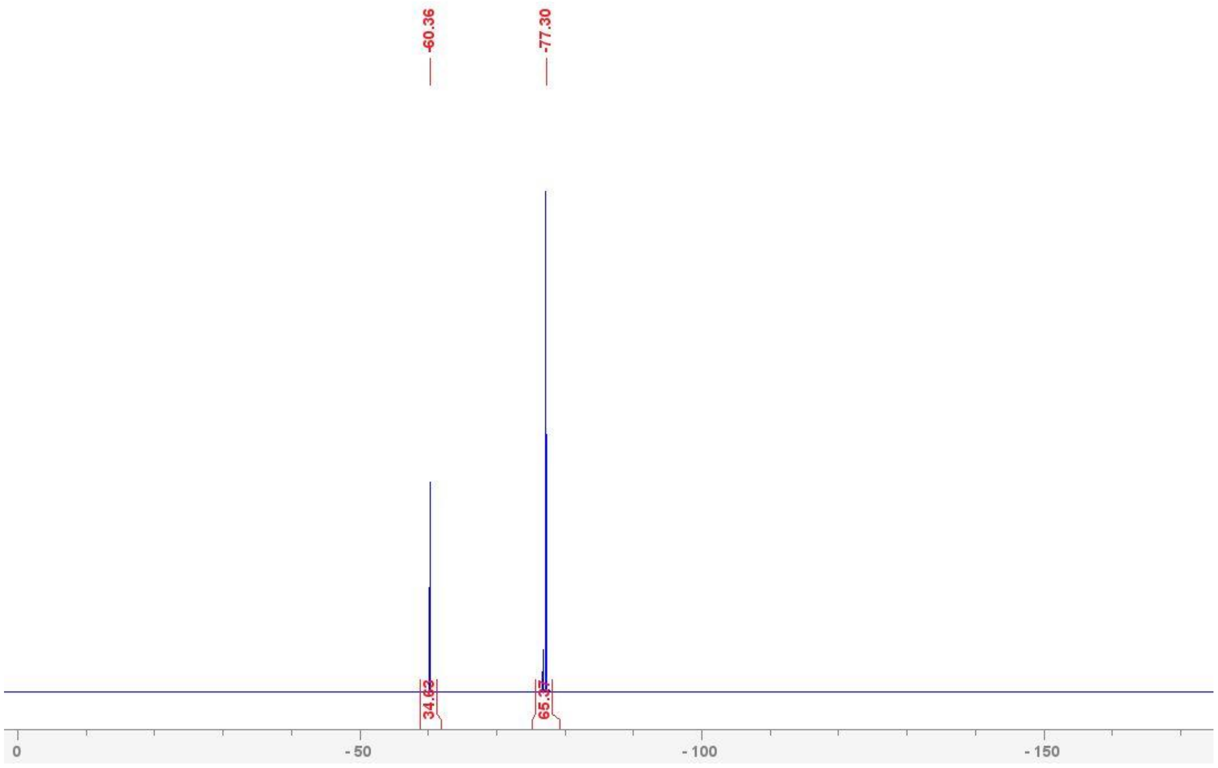
-60.36
-77.30
34.62
65.3
0
-50
-100
-150

3-(Imidazo[1,2-*b*]pyridazin-3-ylethynyl)-2-methyl-*N*-(4-((4-methylpiperazin-1-yl)methyl)-3-(trifluoromethyl)phenyl)benzamide (**2**)

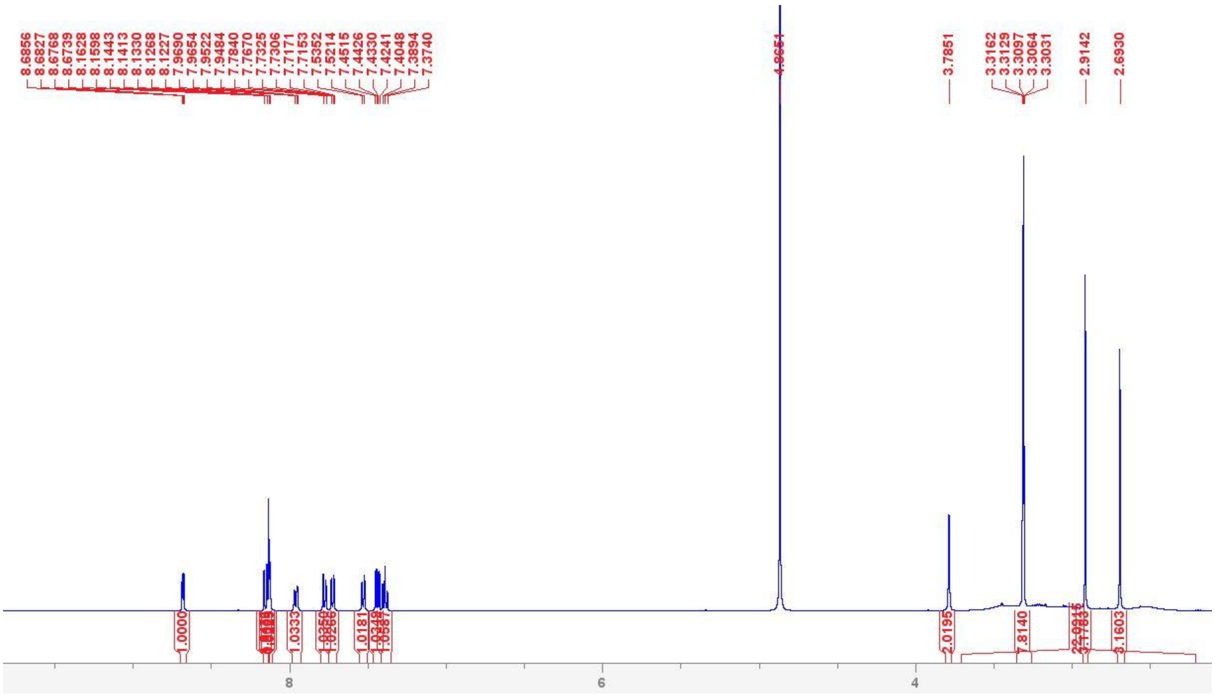

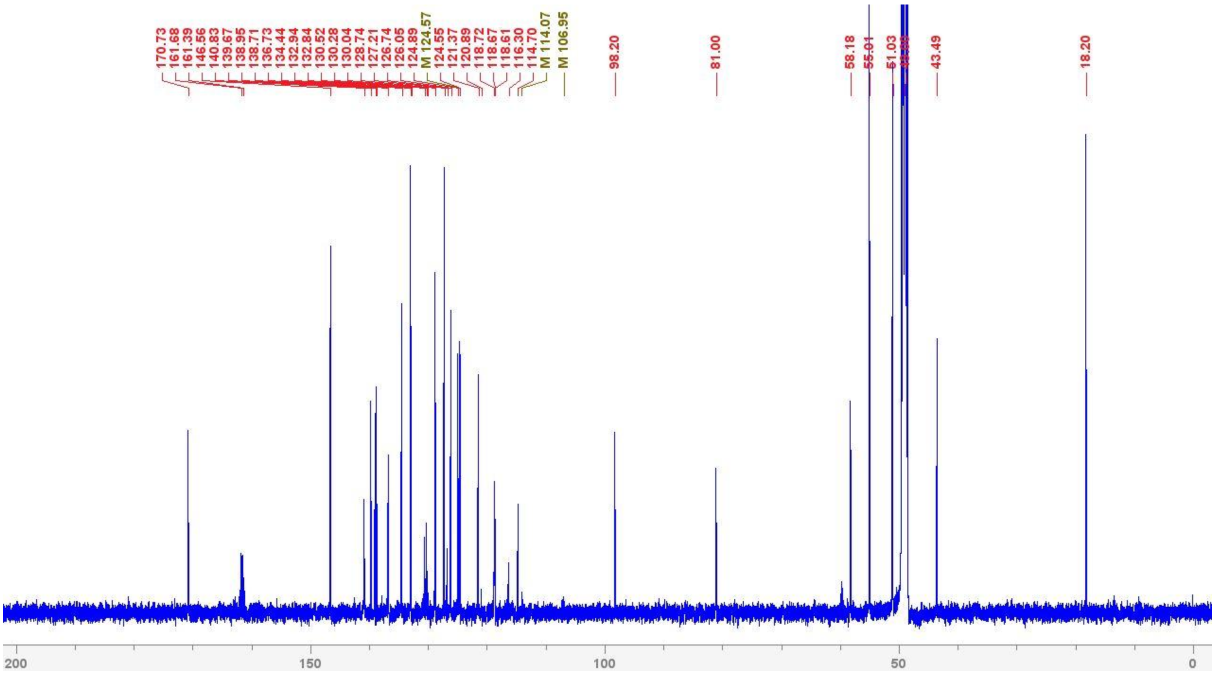

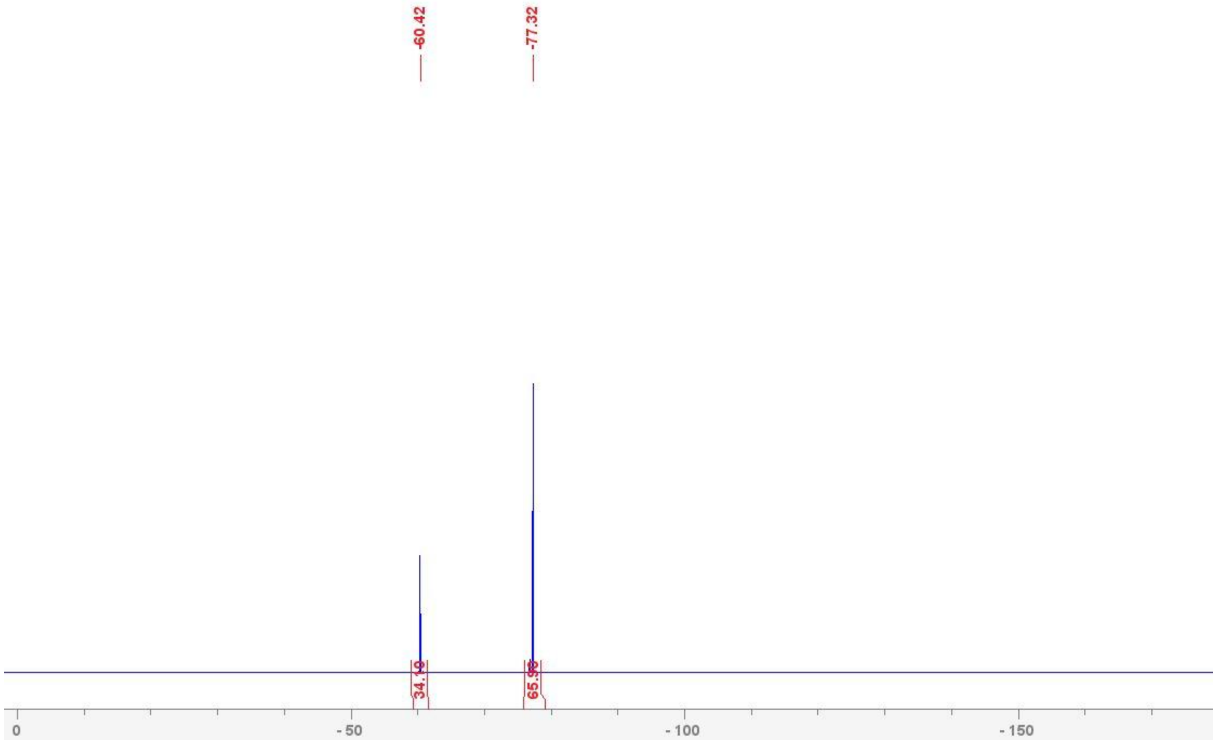

-60.42
-77.32
34.10
65.90
0
- 50
- 100
- 150

5-(Imidazo[1,2-*b*]pyridazin-3-ylethynyl)-2-methyl-*N*-(4-((4-methylpiperazin-1-yl)methyl)-3-(trifluoromethyl)phenyl)benzamide (**3**)

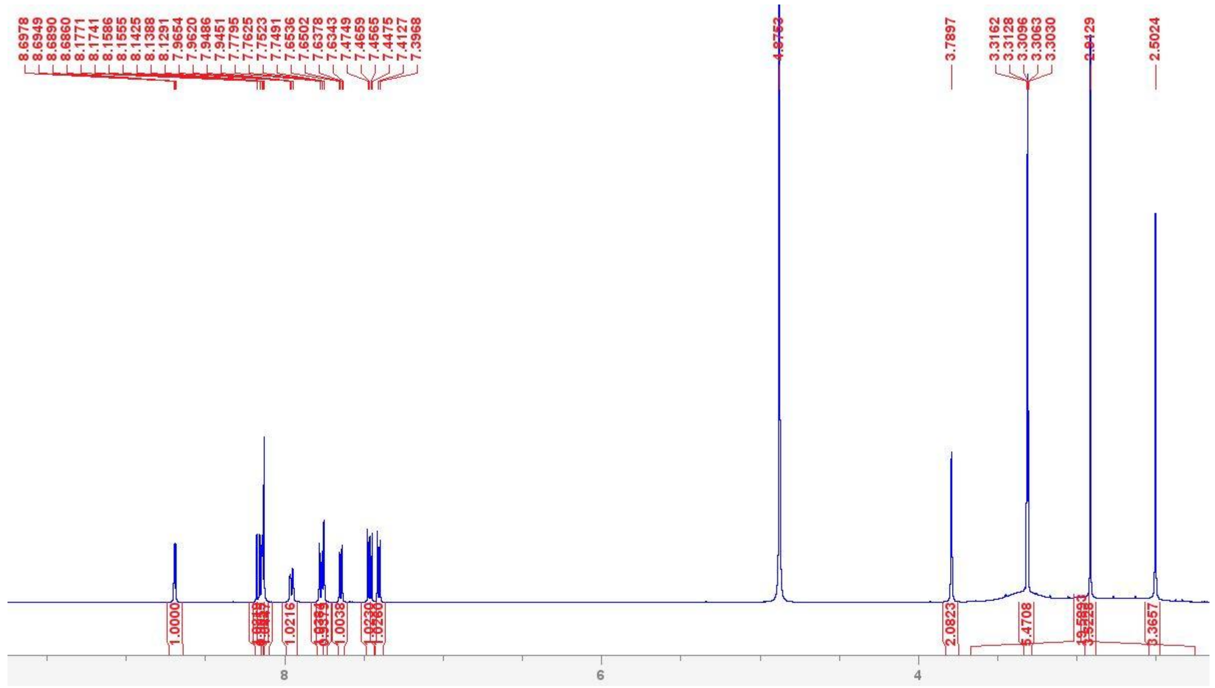

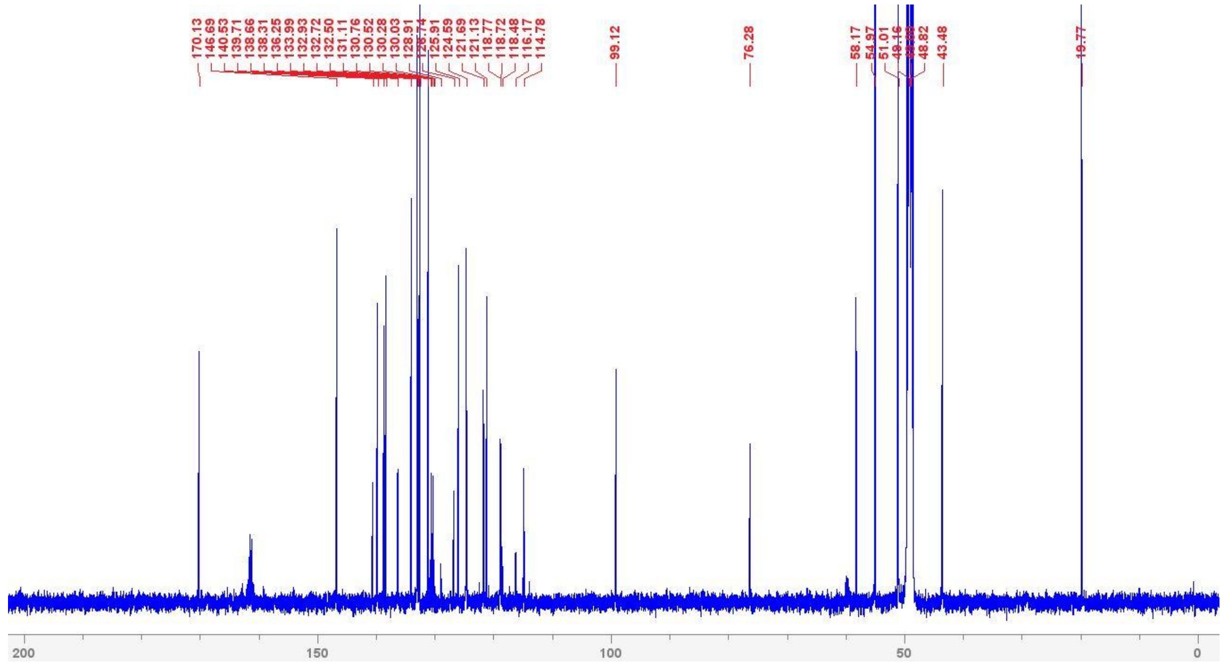

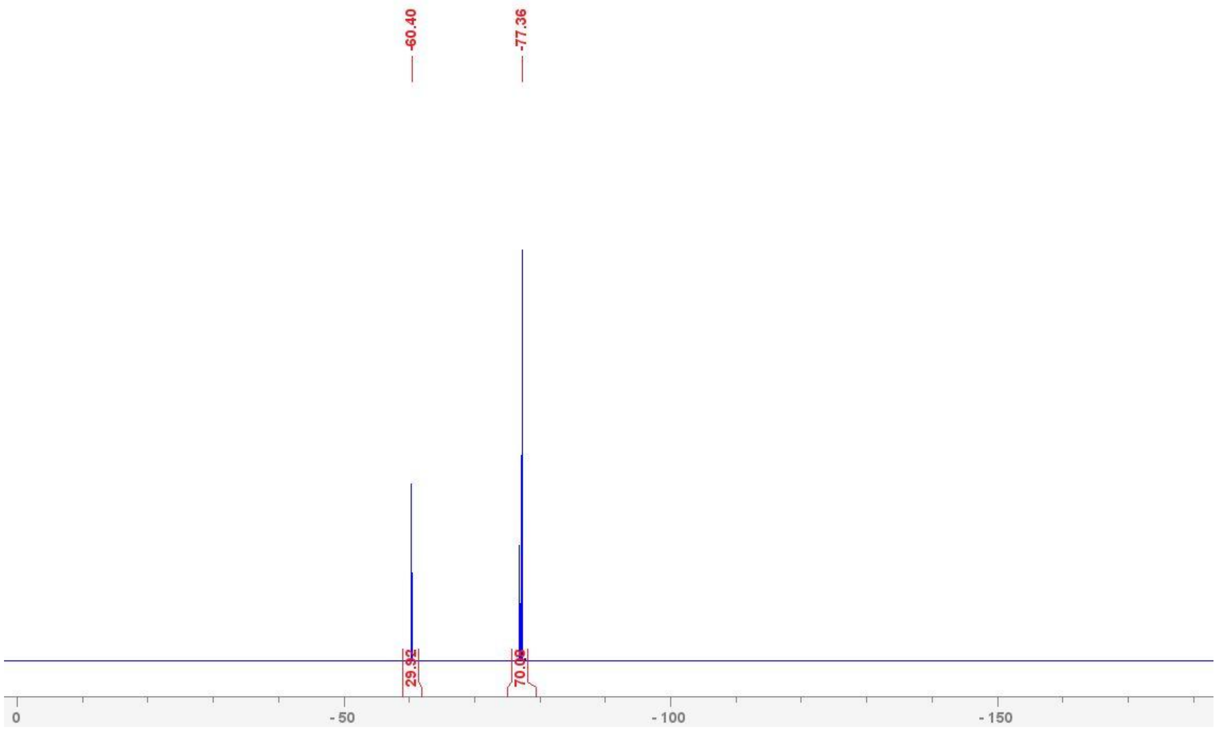
-60.40
-77.36
29.92
70.08
0
-50
-100
-150

3-(Imidazo[1,2-*b*]pyridazin-3-ylethynyl)-2,4-dimethyl-*N*-(4-((4-methylpiperazin-1-yl)methyl)-3-(trifluoromethyl)phenyl)benzamide (**4**)

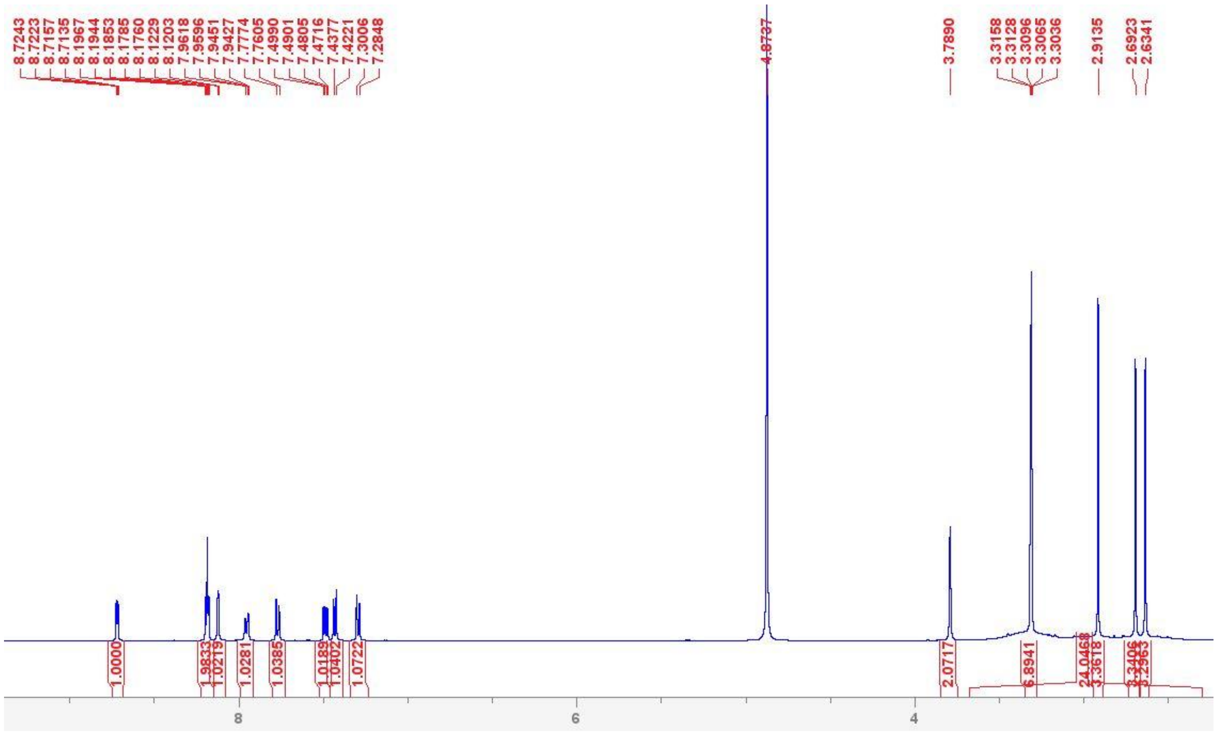

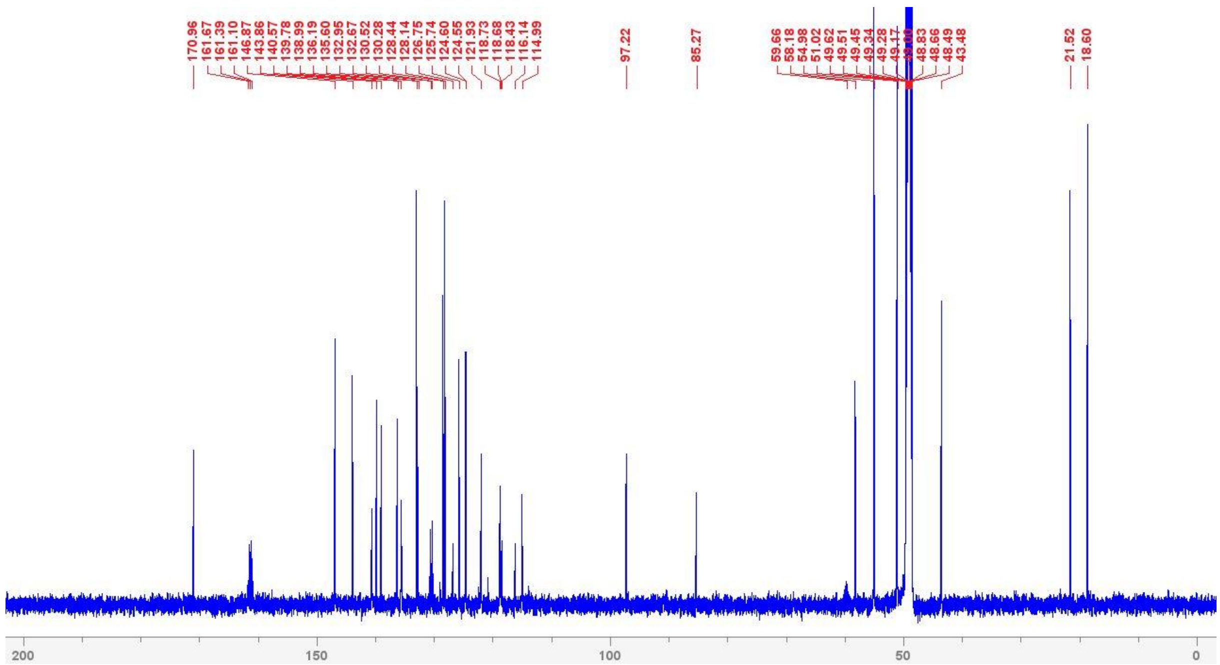

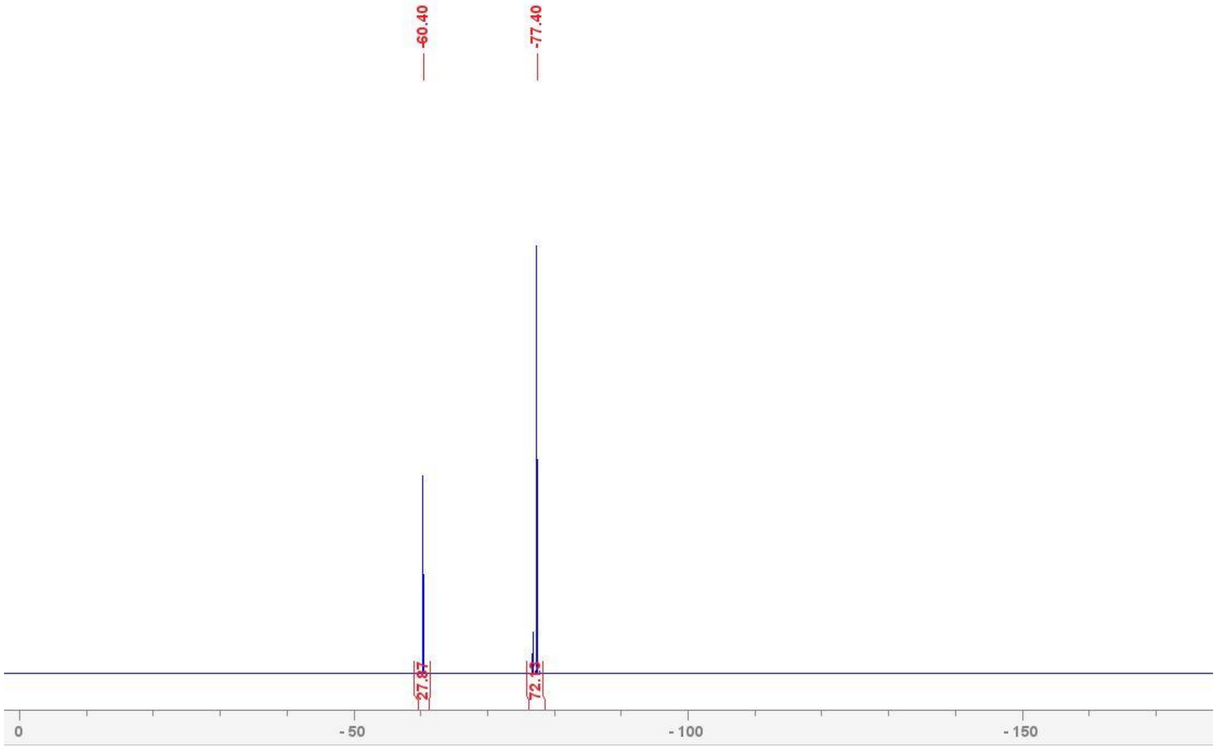
-60.40
-77.40
27.87
72.13
0
-50
-100
-150

(*E*)-*N*-(4-(3-aminoprop-1-en-1-yl)-3-(trifluoromethyl)phenyl)-3-(imidazo[1,2-*b*]pyridazin-3-ylethynyl)-2-methylbenzamide (**5**)

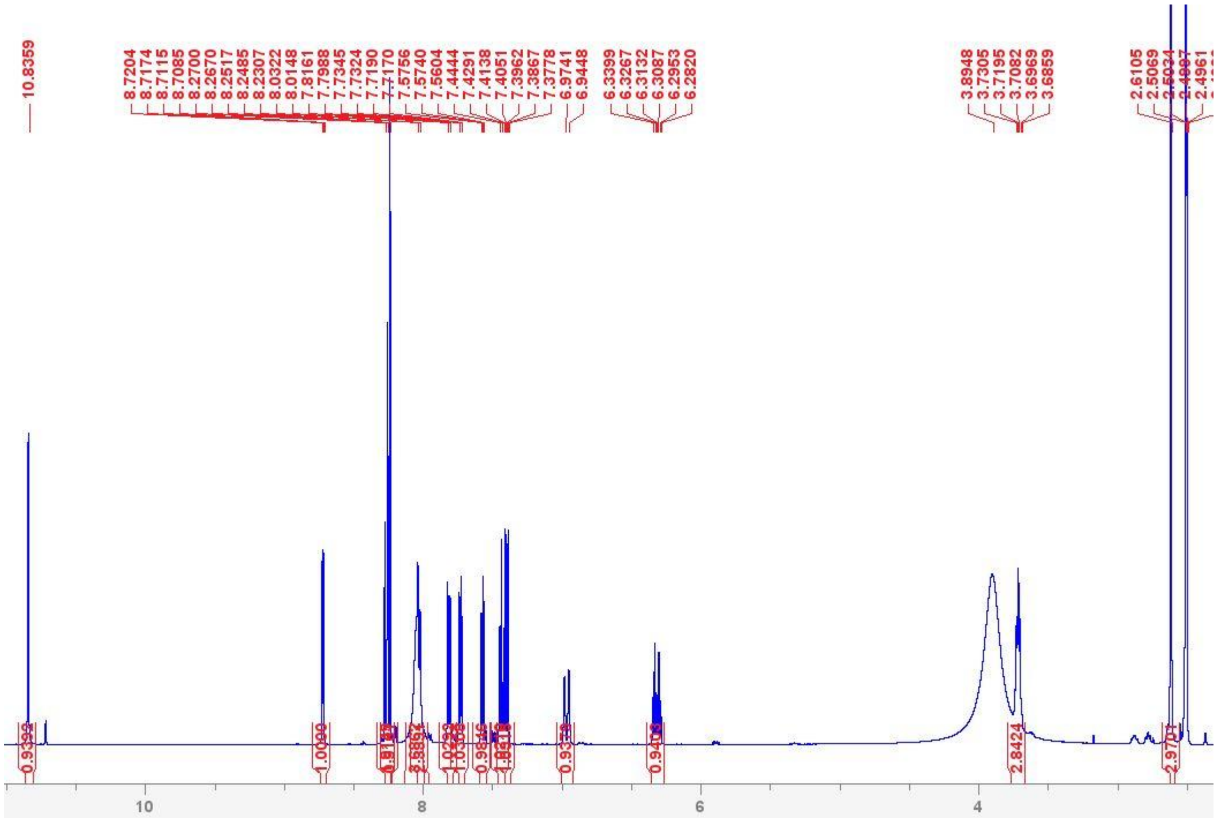

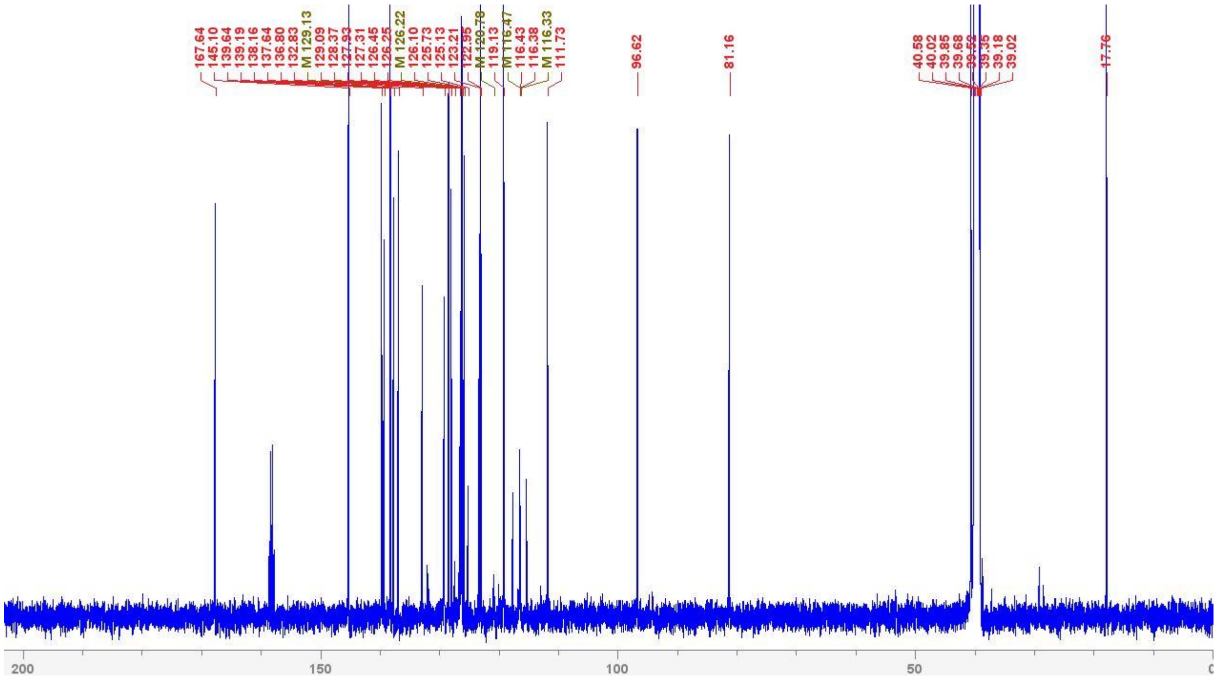

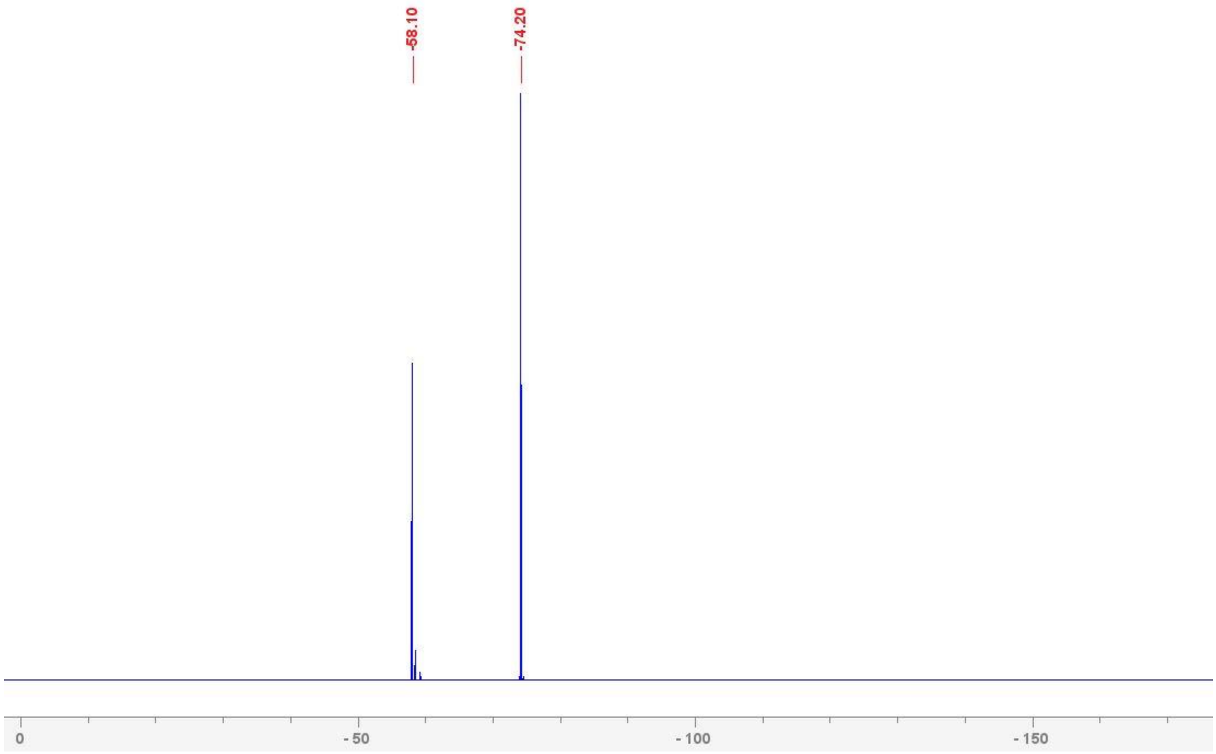
-58.10
-74.20
0
- 50
- 100
- 150

(*E*)-*N*-(4-(3-(1,3-Dioxoisoindolin-2-yl)prop-1-en-1-yl)-3-(trifluoromethyl)phenyl)-3-(imidazo[1,2-*b*]pyridazin-3-ylethynyl)-2-methylbenzamide (**5a**)

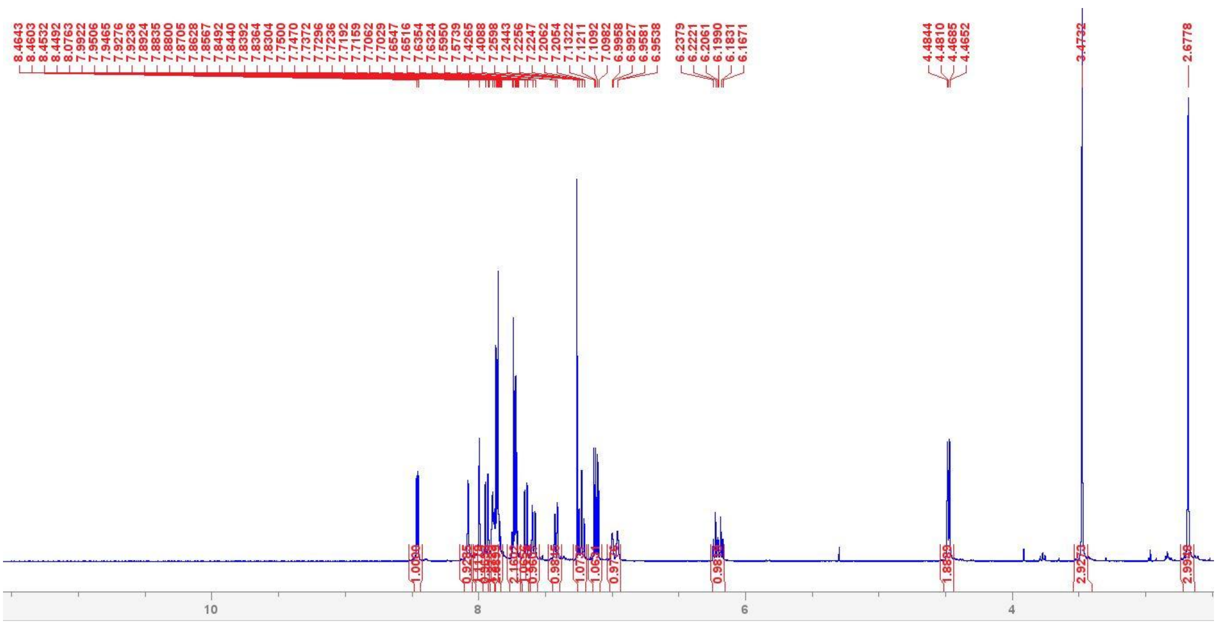

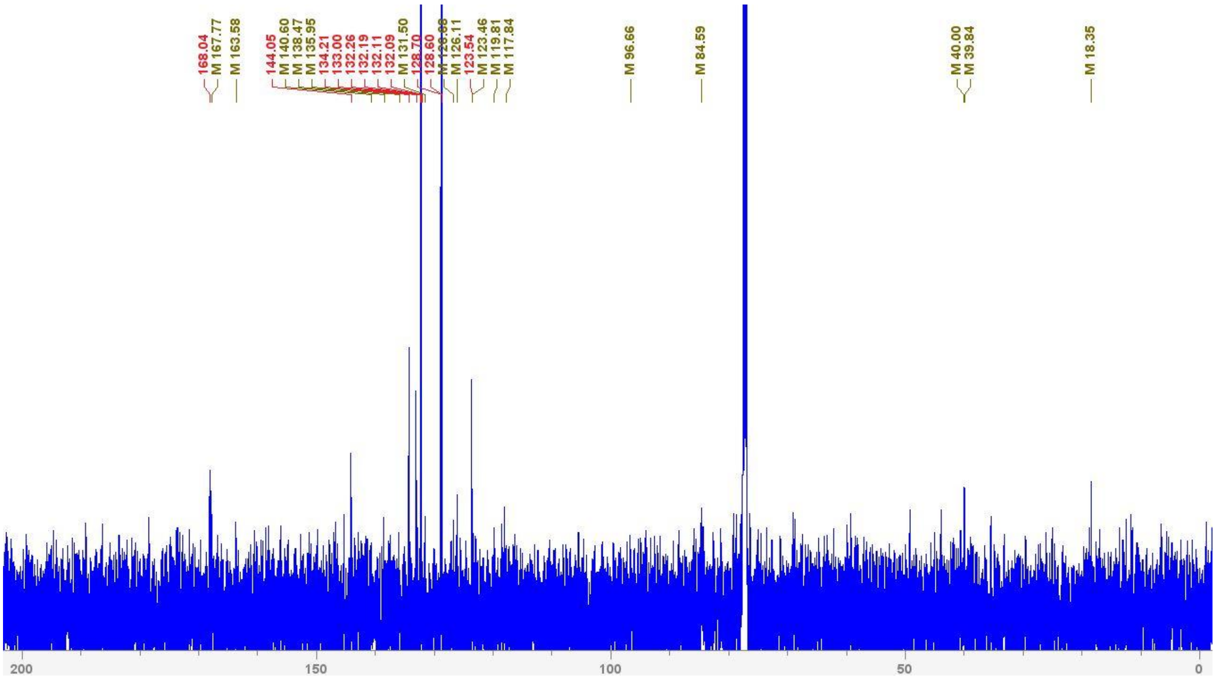

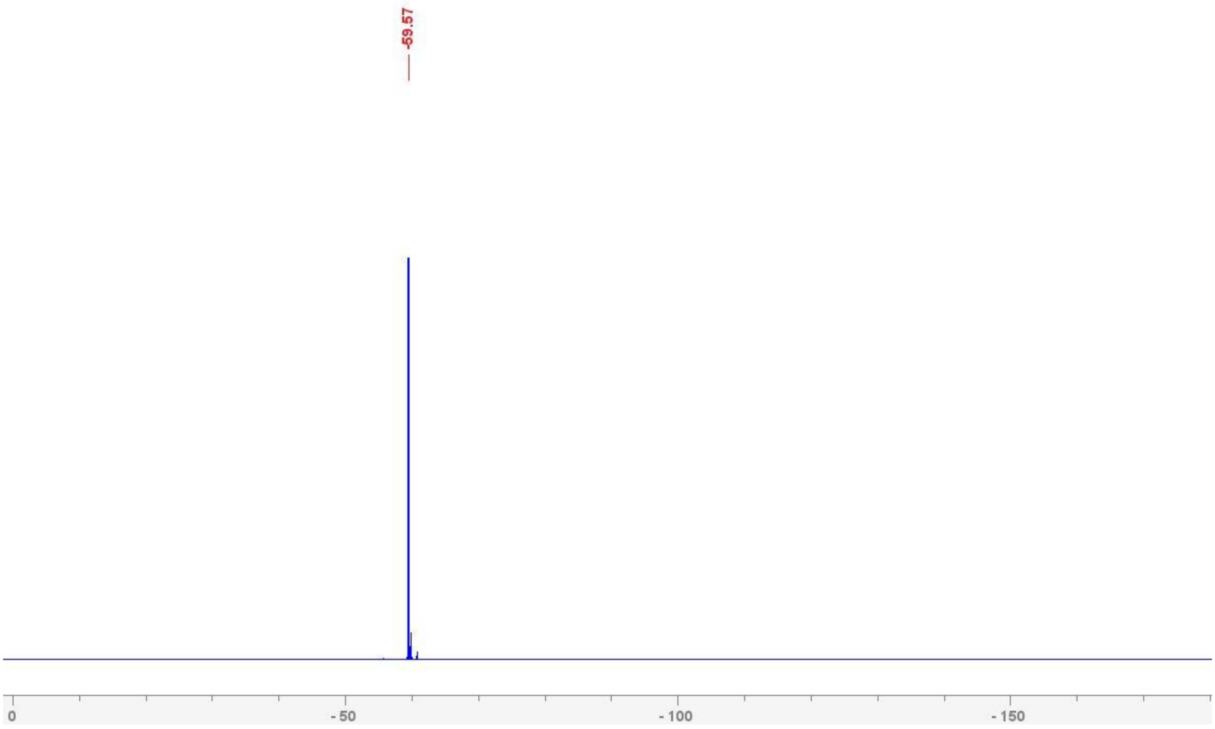

-59.57
0
- 50
- 100
- 150

(*E*)-*N*-(4-(3-aminoprop-1-en-1-yl)-3-(trifluoromethyl)phenyl)-3-(imidazo[1,2-*b*]pyridazin-3-ylethynyl)-4-methylbenzamide (**6**)

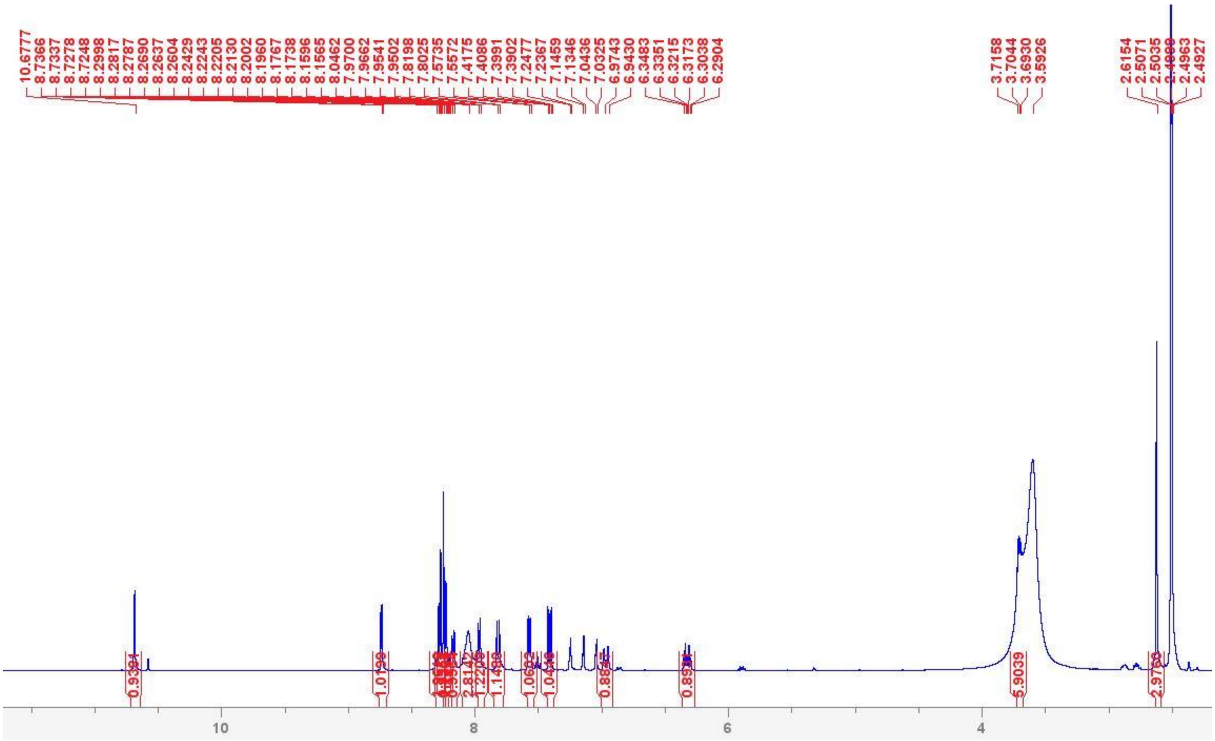

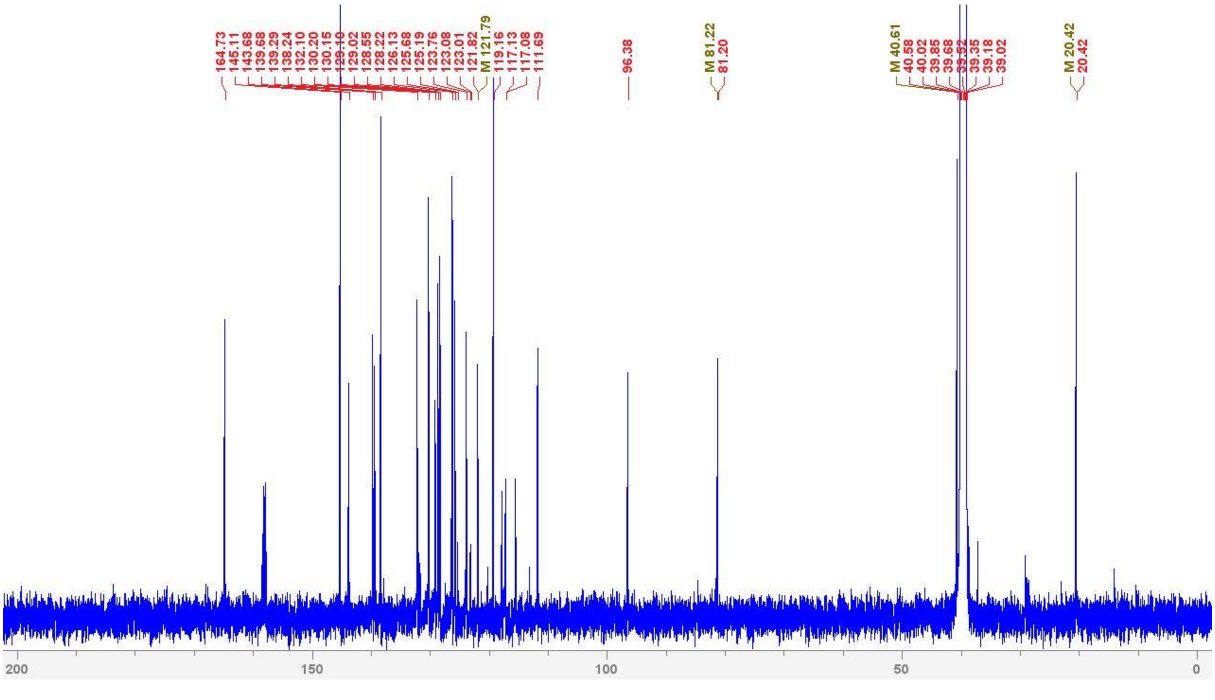

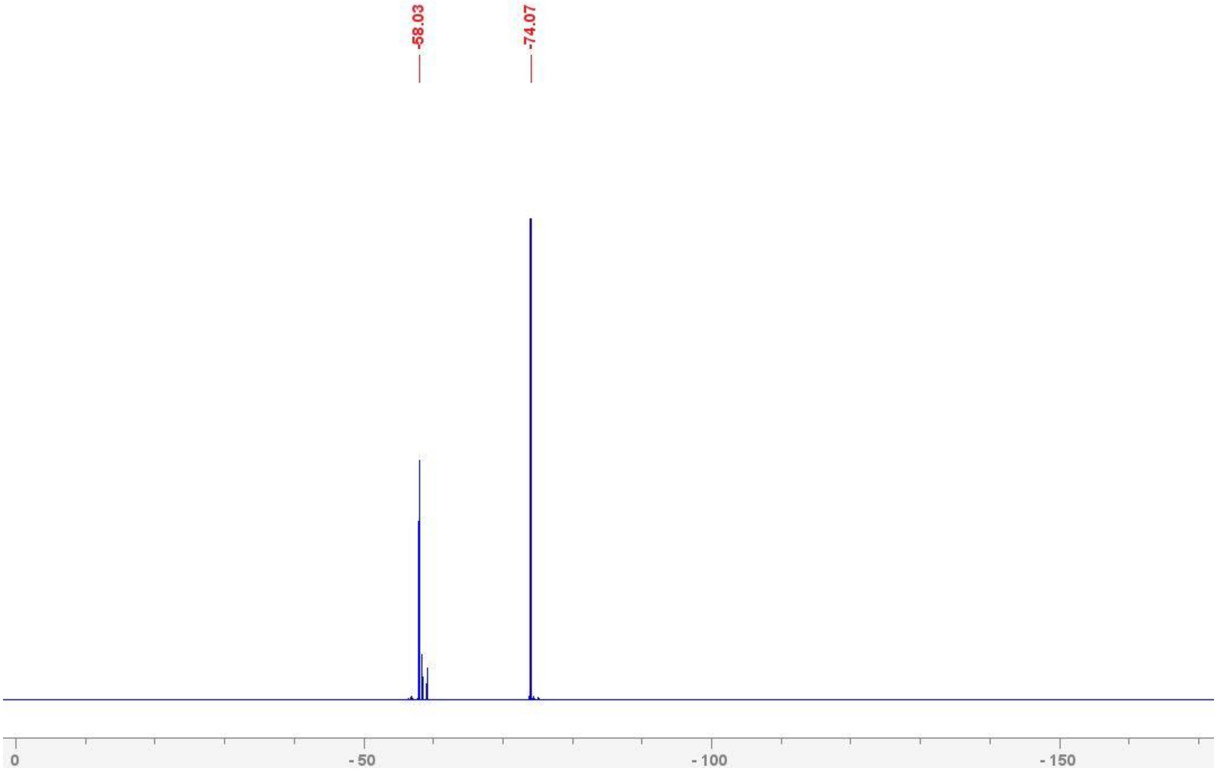

-58.03
-74.07
0
- 50
- 100
- 150

(*E*)-*N*-(4-(3-(1,3-dioxoisoindolin-2-yl)prop-1-en-1-yl)-3-(trifluoromethyl)phenyl)-3-(imidazo[1,2-*b*]pyridazin-3-ylethynyl)-4-methylbenzamide (**6a**)

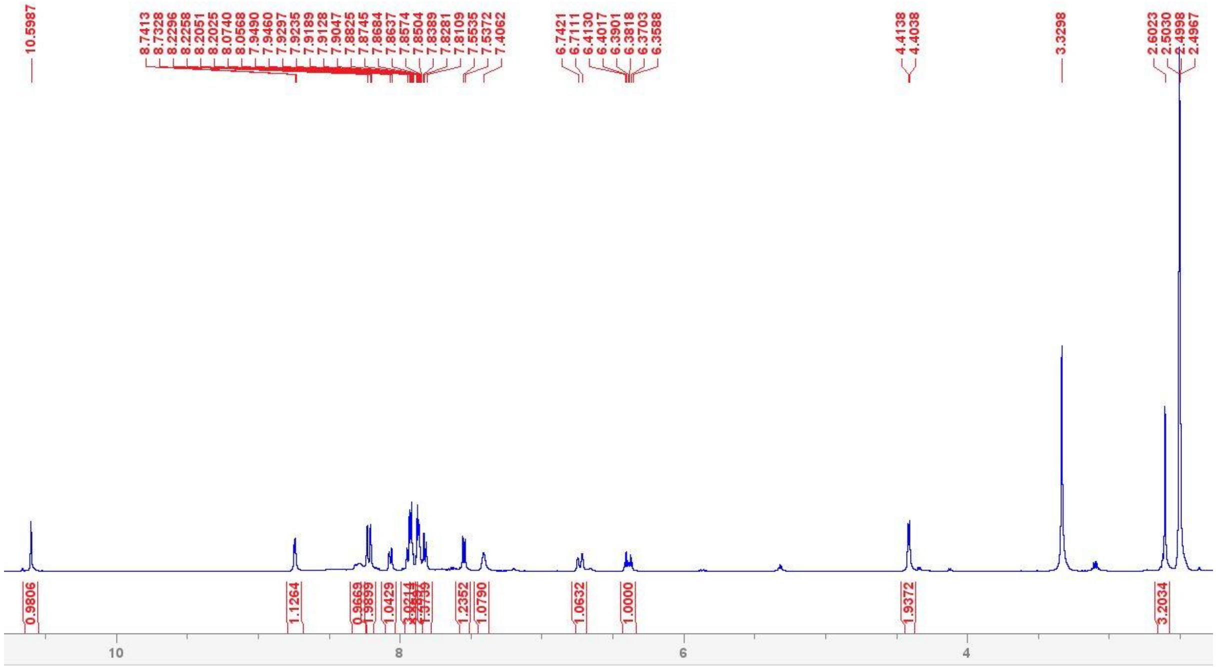

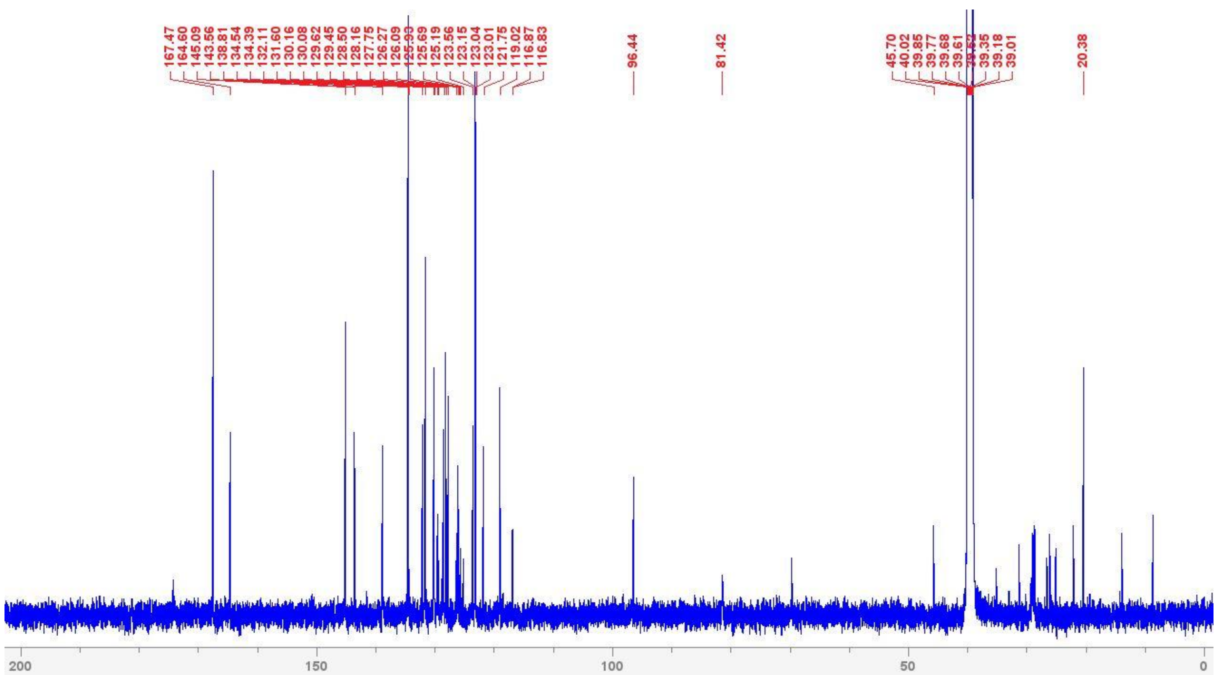

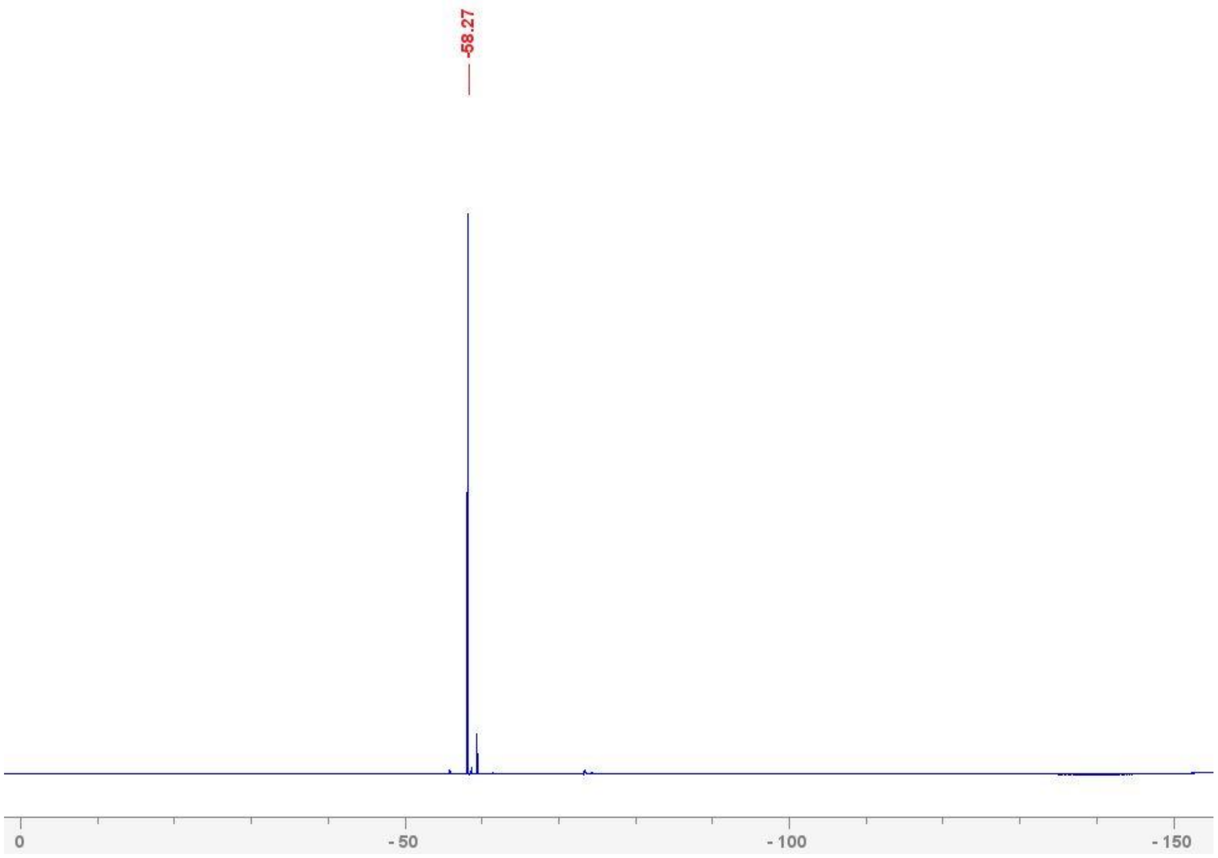
-58.27
0
- 50
- 100
- 150

(*E*)-*N*-(4-(3-aminoprop-1-en-1-yl)-3-(trifluoromethyl)phenyl)-3-(imidazo[1,2-*b*]pyridazin-3-ylethynyl)-6-methylbenzamide (**7**)

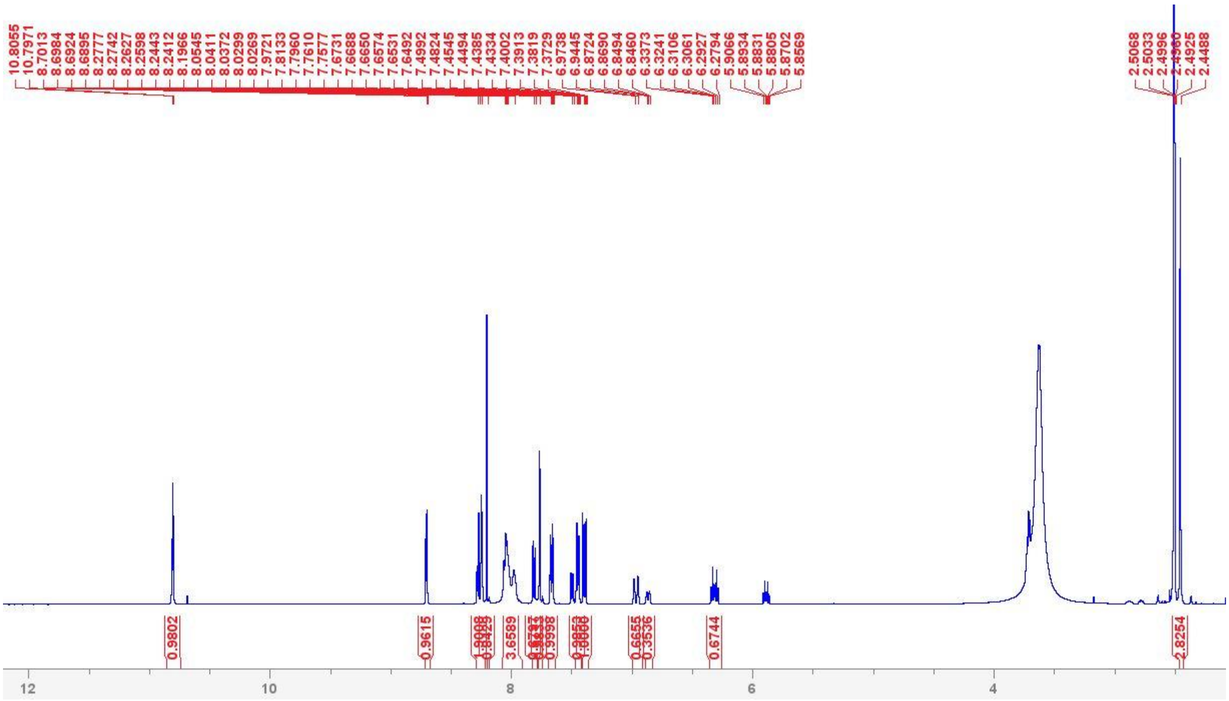

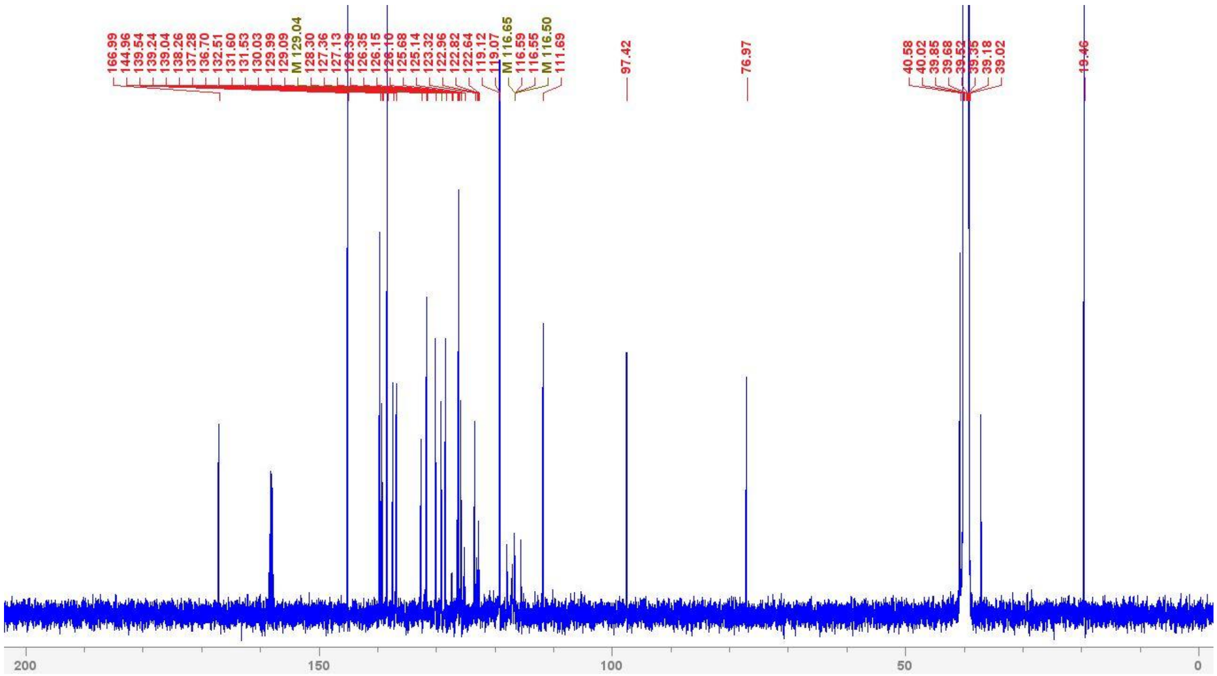

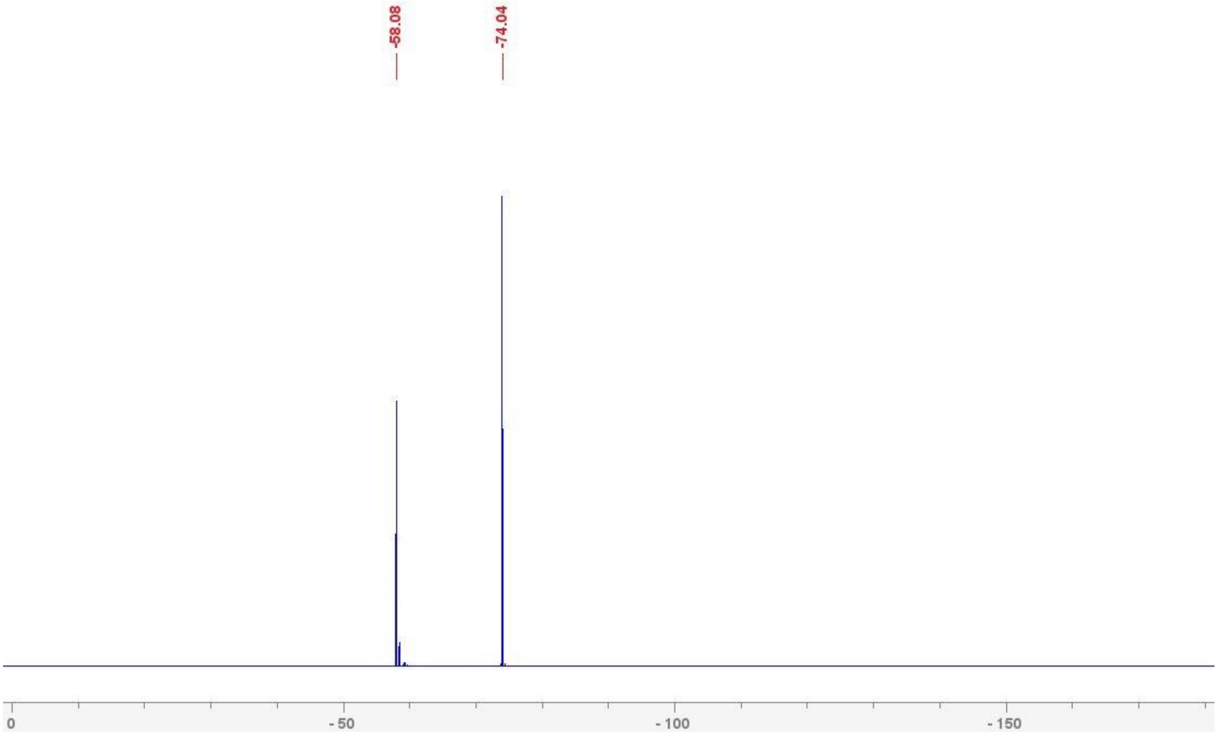
-58.08
-74.04
0
- 50
- 100
- 150

(*E*)-*N*-(4-(3-(1,3-dioxoisoindolin-2-yl)prop-1-en-1-yl)-3-(trifluoromethyl)phenyl)-5-(imidazo[1,2-*b*]pyridazin-3-ylethynyl)-2-methylbenzamide (**7a**)

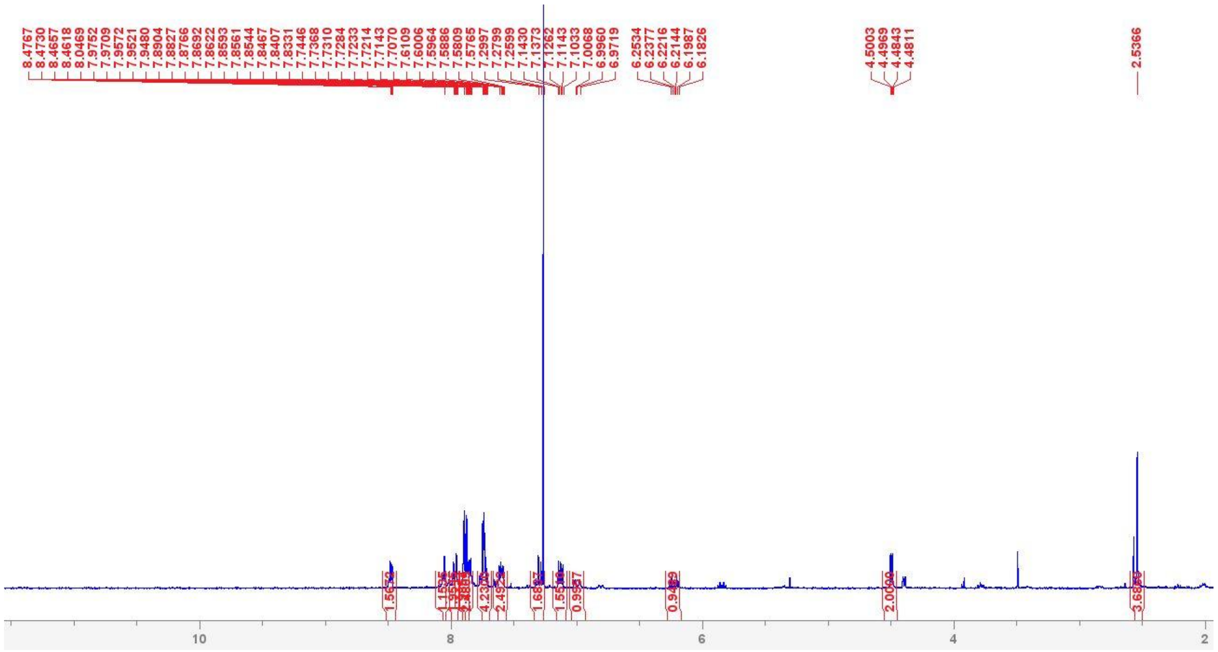

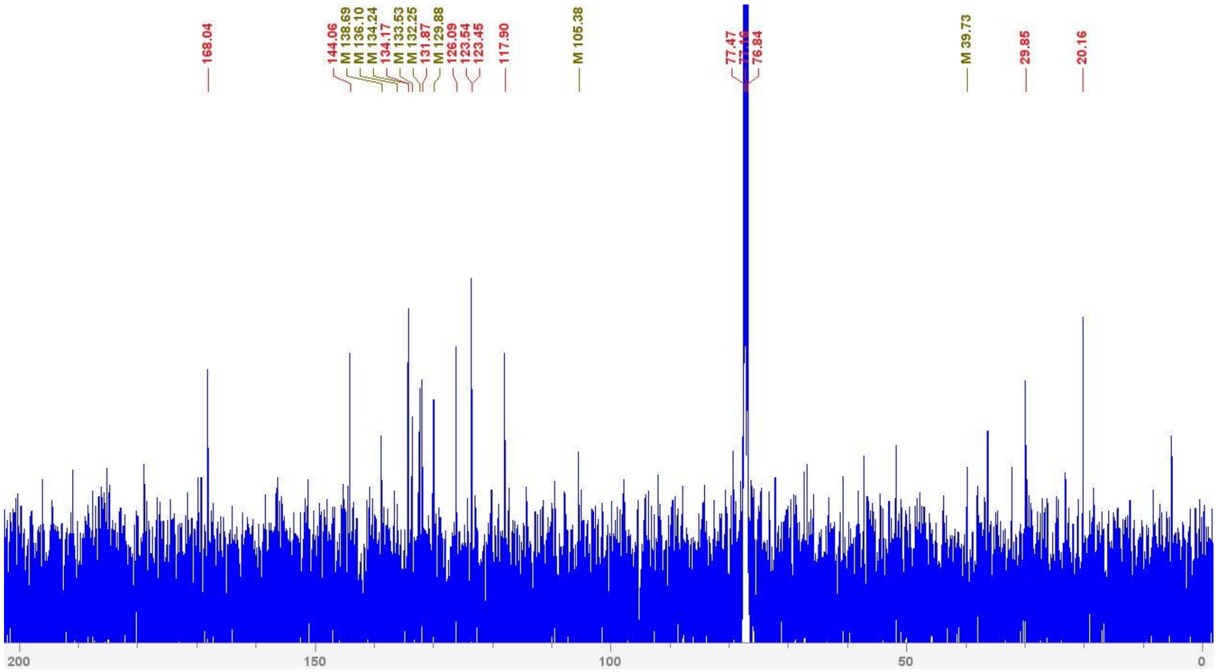

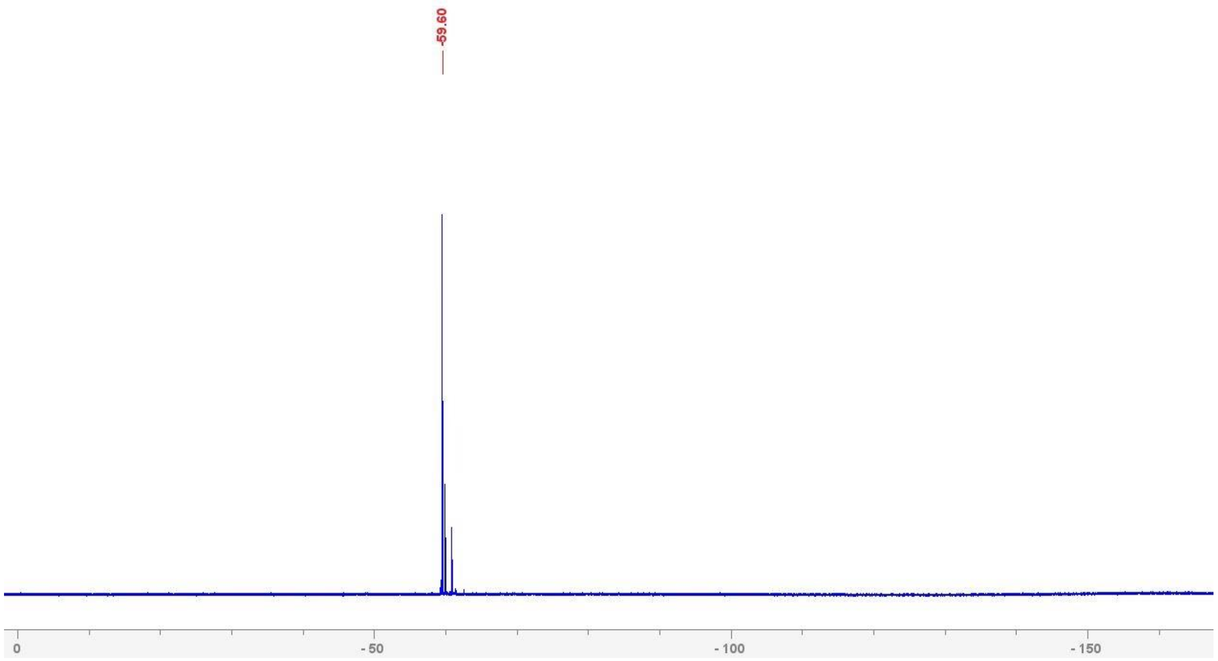
-59.60
0
- 50
- 100
- 150

(*E*)-*N*-(4-(3-aminoprop-1-en-1-yl)-3-(trifluoromethyl)phenyl)-3-(imidazo[1,2-*b*]pyridazin-3-ylethynyl)-2,4-dimethylbenzamide (**8**)

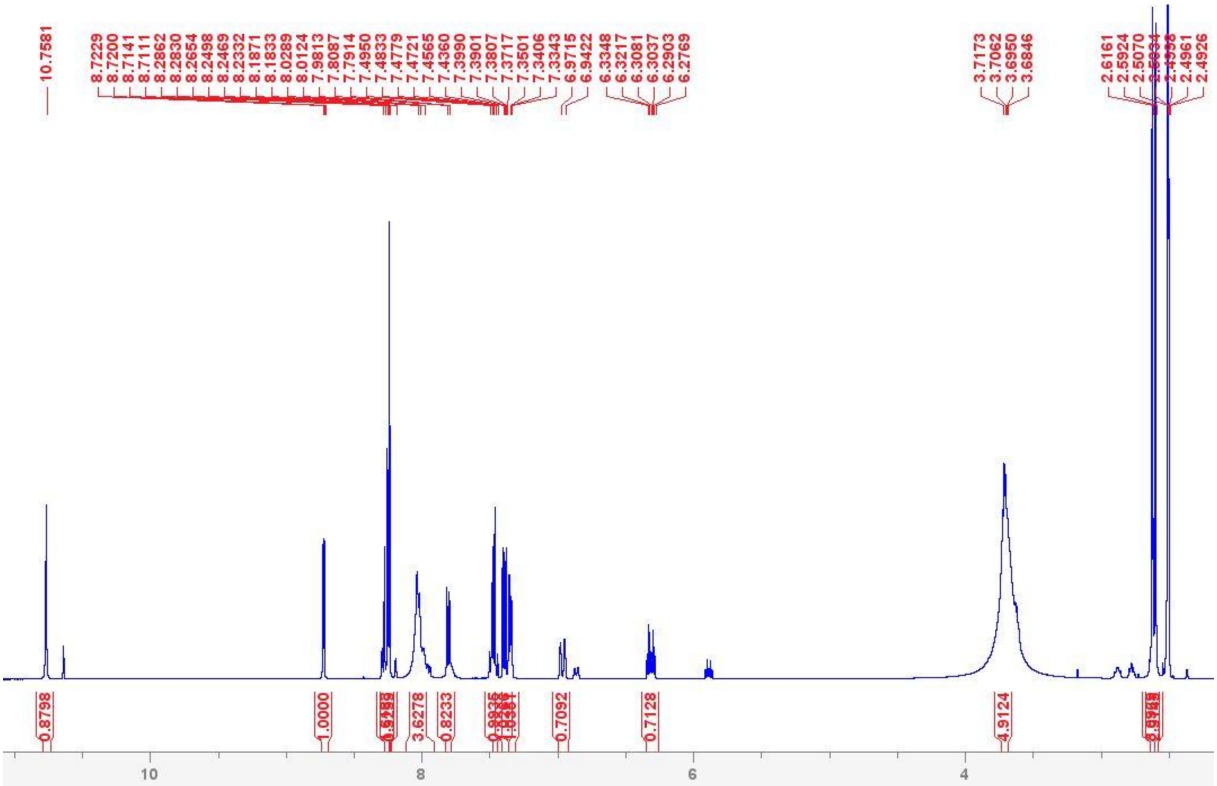

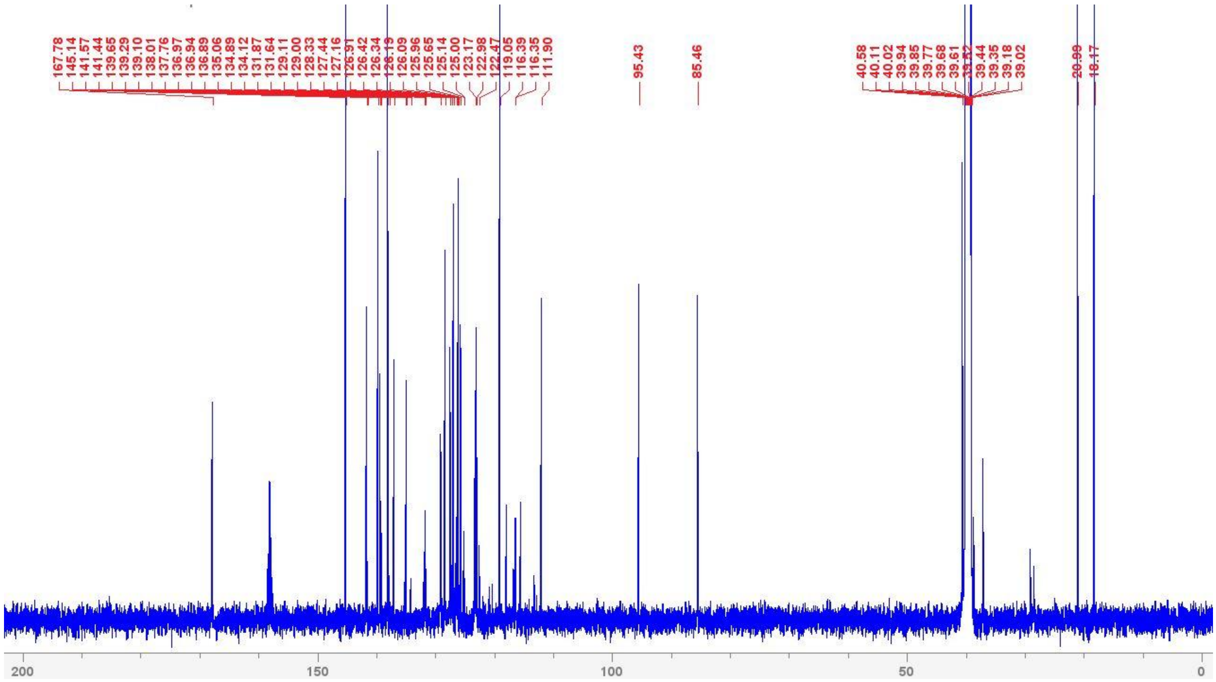

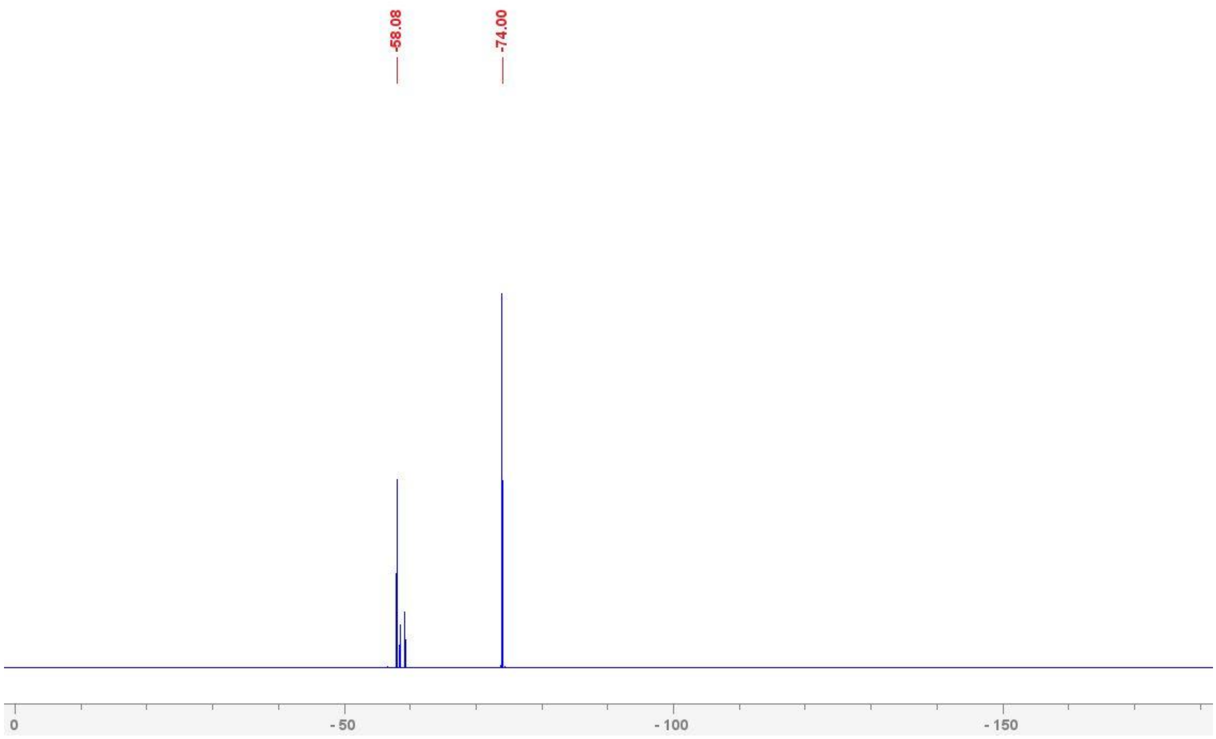
-58.08
-74.00
0
- 50
- 100
- 150

(*E*)-*N*-(4-(3-(1,3-dioxoisoindolin-2-yl)prop-1-en-1-yl)-3-(trifluoromethyl)phenyl)-3-(imidazo[1,2-*b*]pyridazin-3-ylethynyl)-2,4-dimethylbenzamide (**8a**)

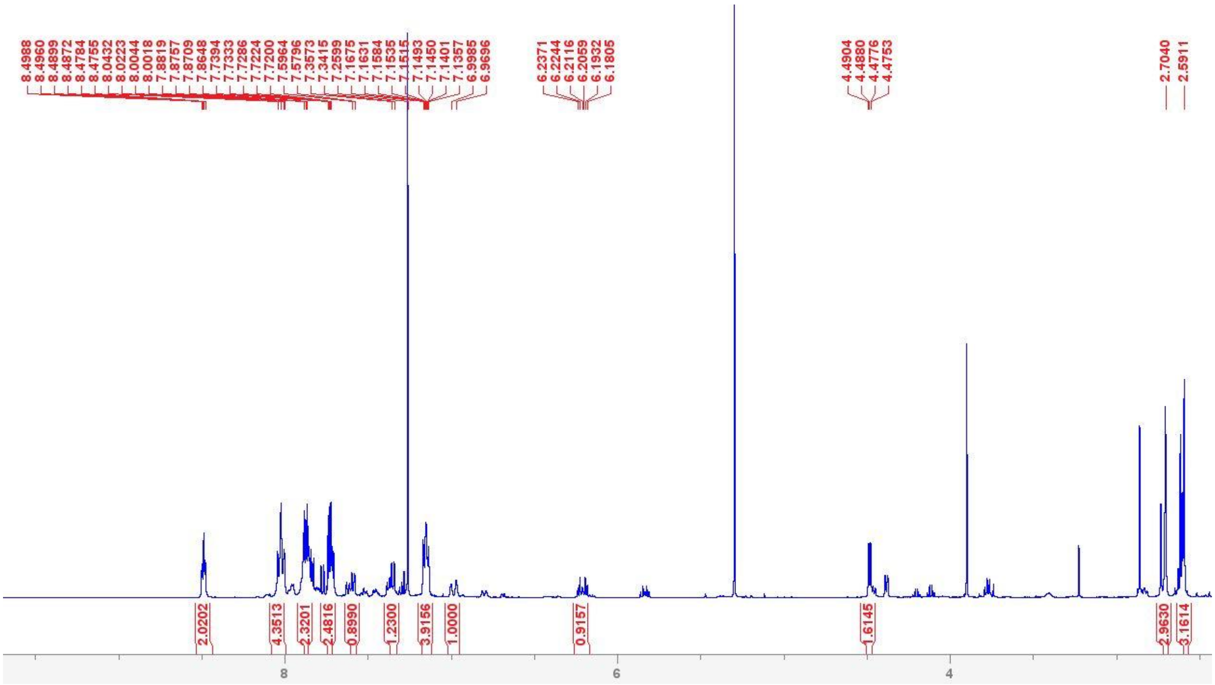

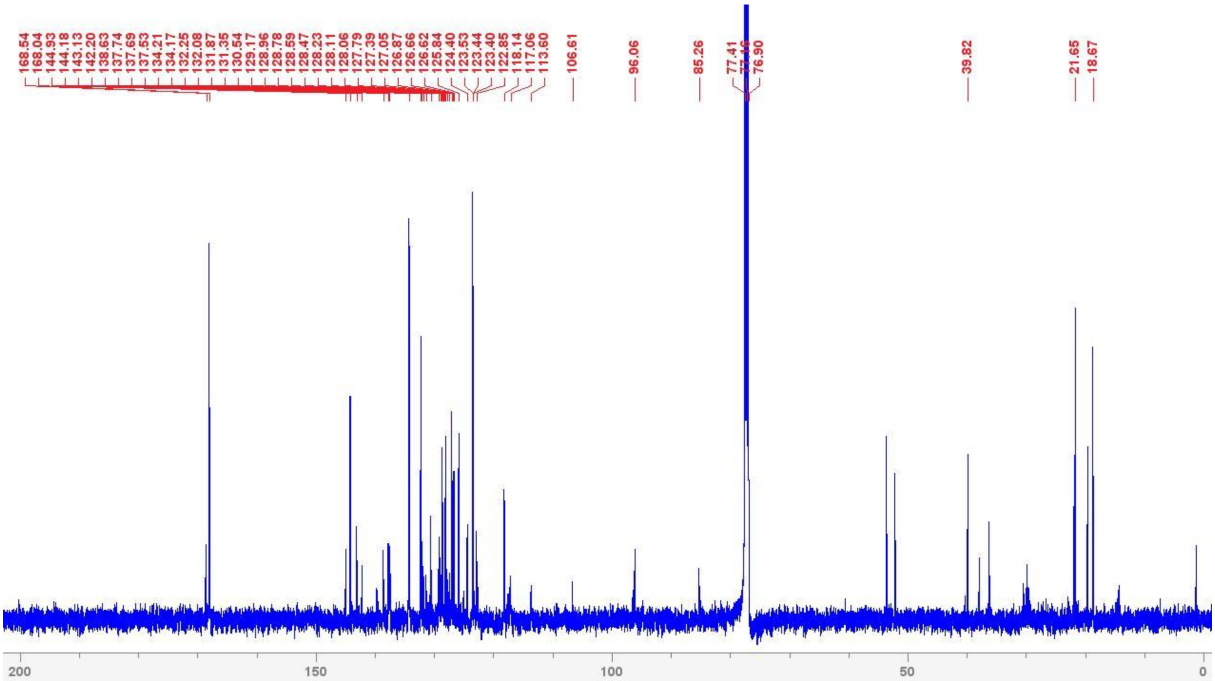

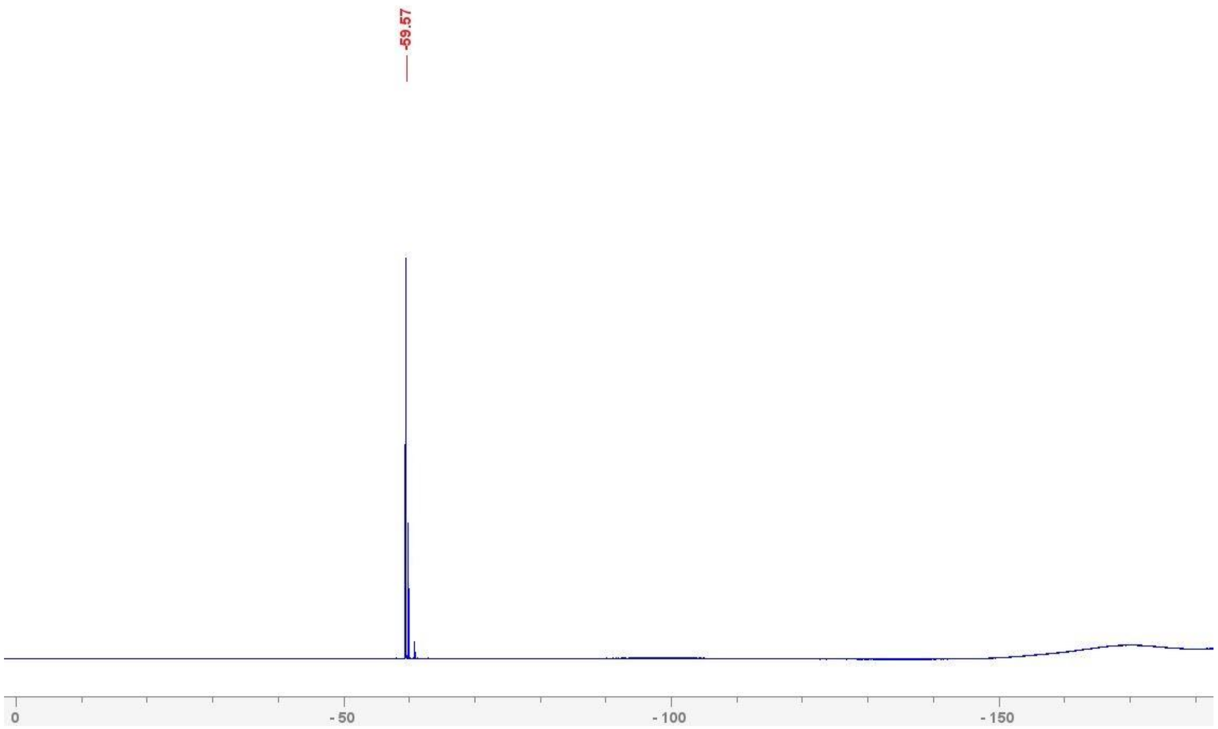
-59.57
0
- 50
- 100
- 150

N-(4-(3-aminopropyl)-3-(trifluoromethyl)phenyl)-3-(imidazo[1,2-*b*]pyridazin-3-ylethynyl)-2-methylbenzamide (**9**)

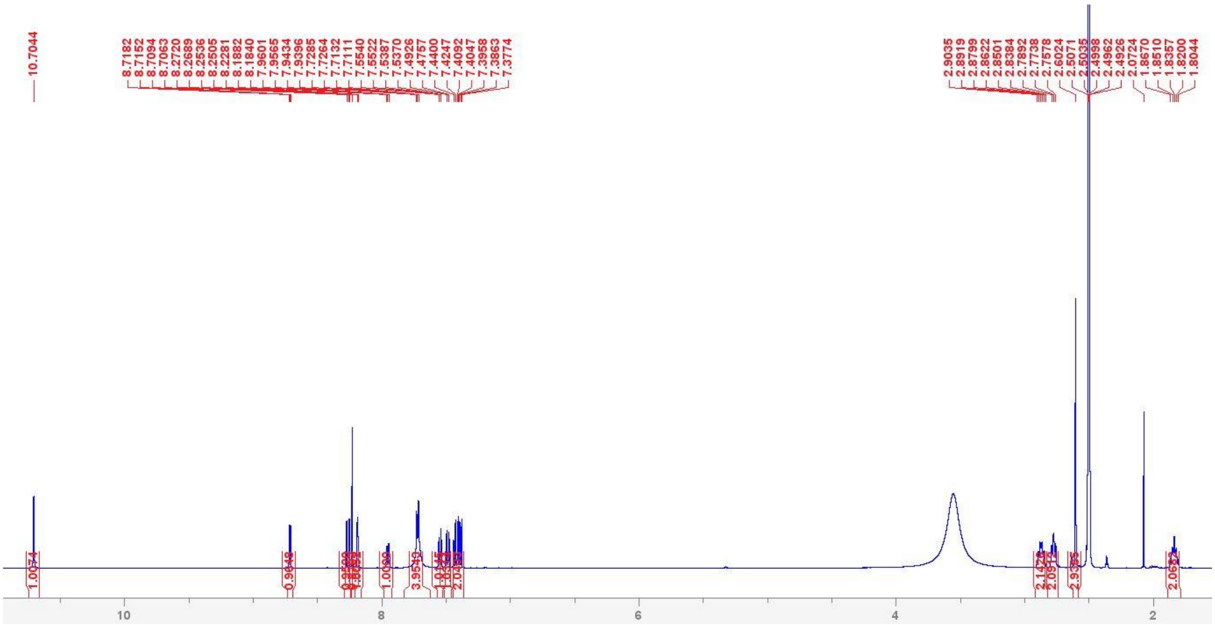

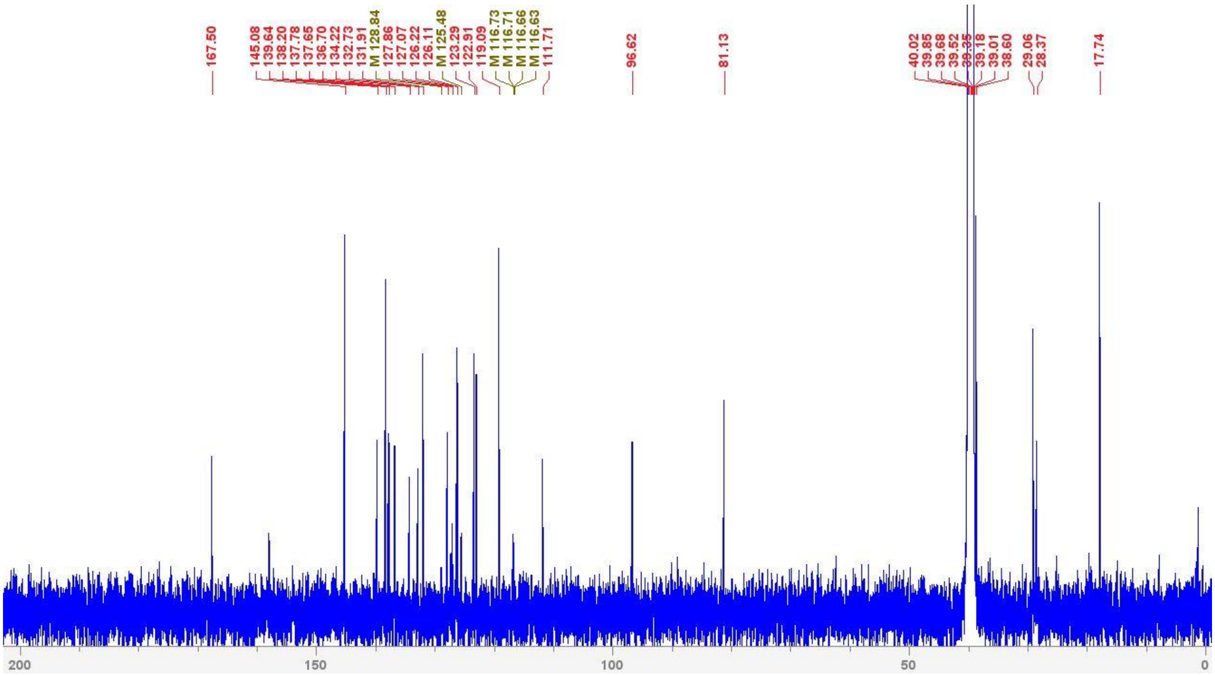

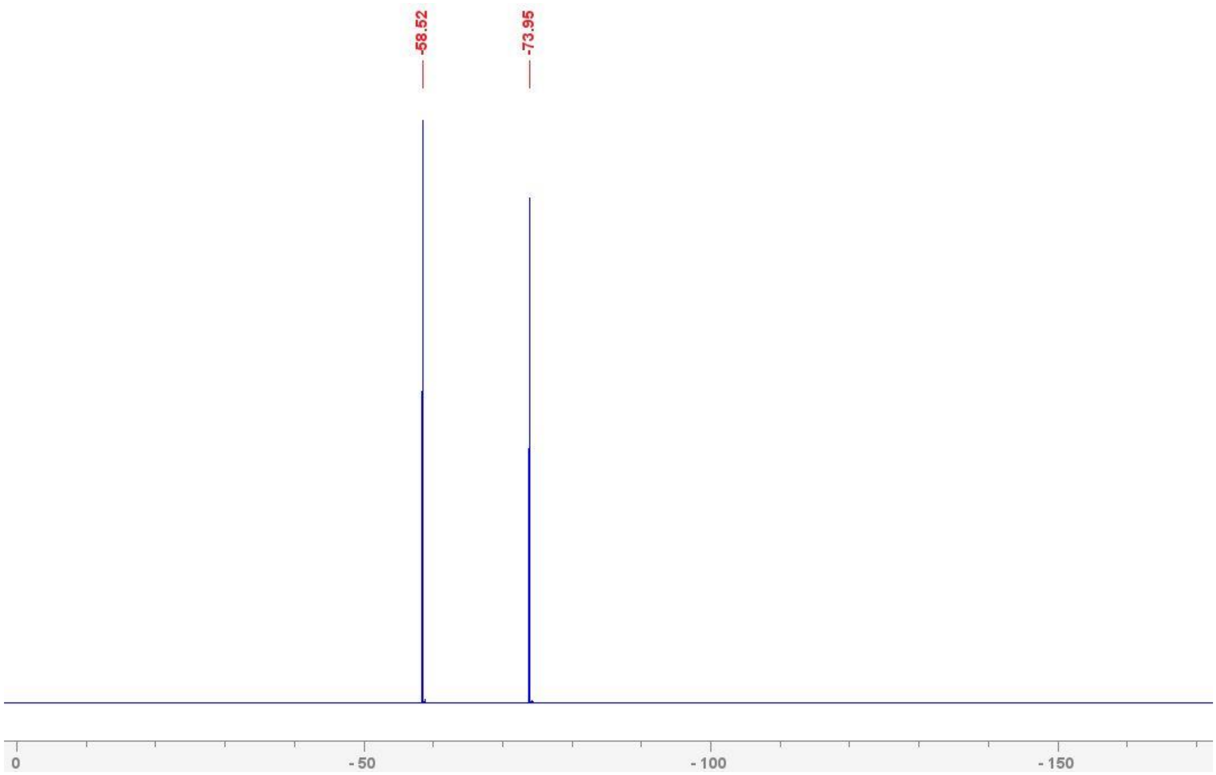
-58.52
-73.95
0
- 50
- 100
- 150

N-(4-(3-(1,3-Dioxoisoindolin-2-yl)propyl)-3-(trifluoromethyl)phenyl)-3-(imidazo[1,2-*b*]pyridazin-3-ylethynyl)-2-methylbenzamide (**9a**)

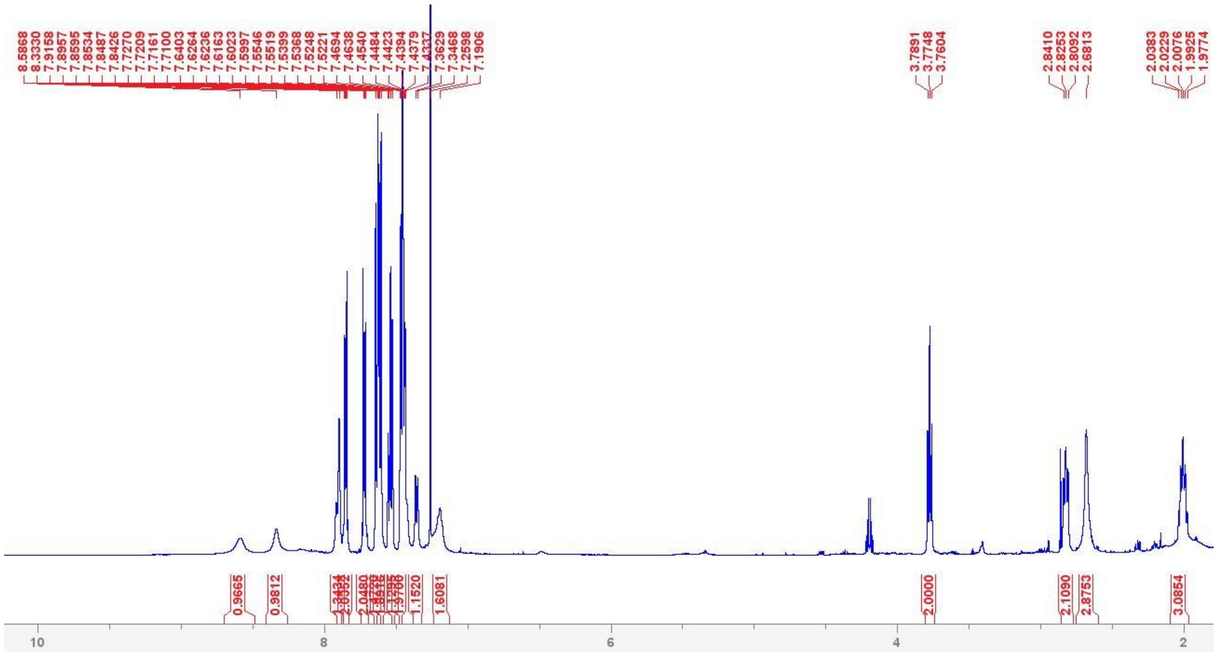

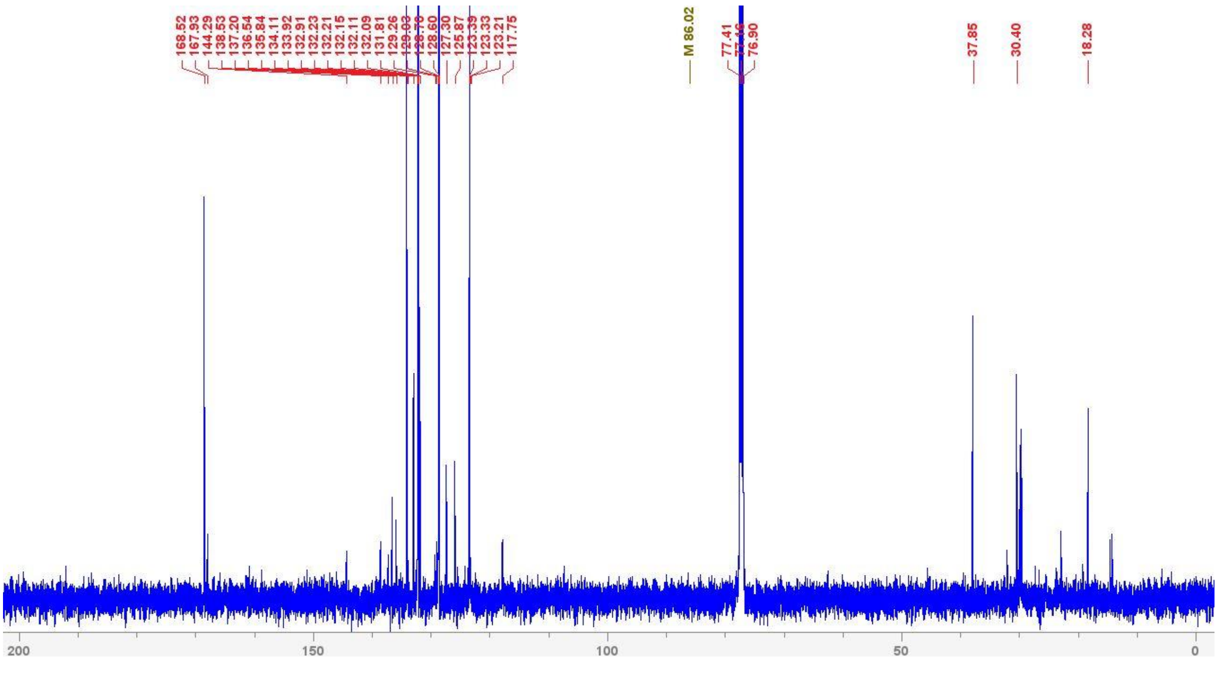

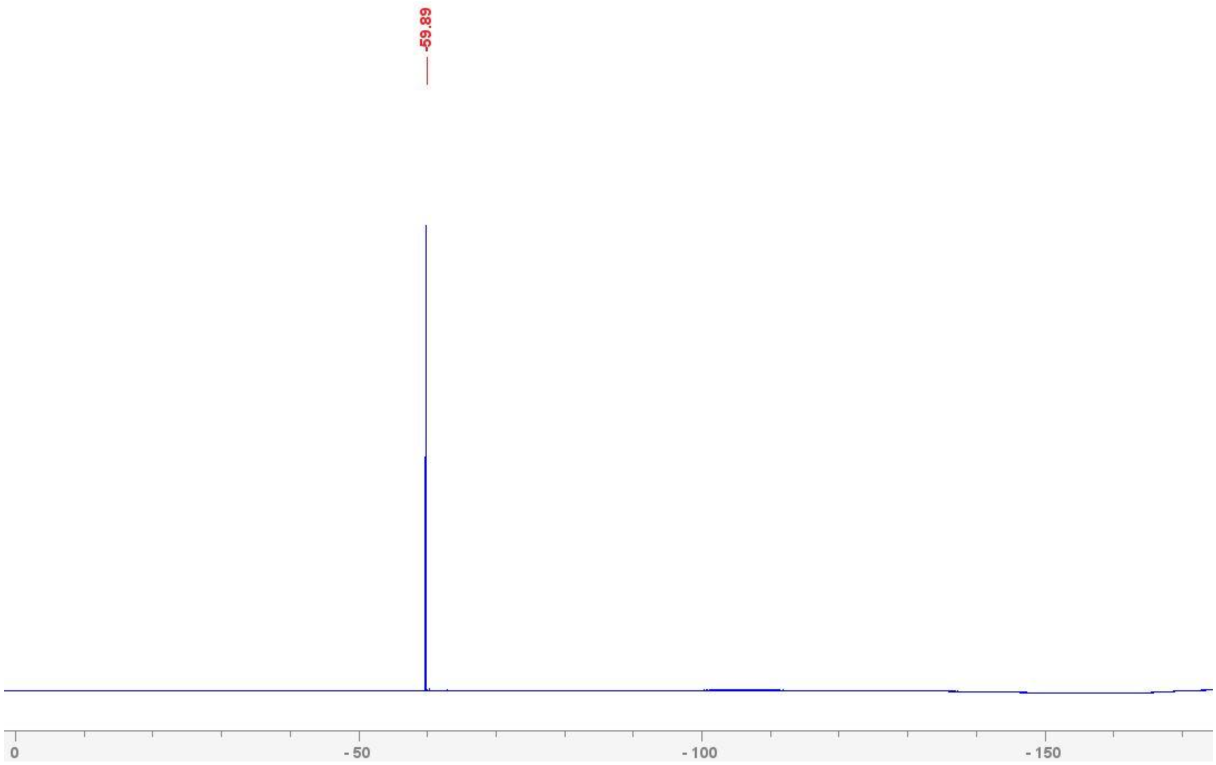
-59.89
0
- 50
- 100
- 150

N-(4-(3-aminopropyl)-3-(trifluoromethyl)phenyl)-3-(imidazo[1,2-*b*]pyridazin-3-ylethynyl)-4-methylbenzamide (**10**)

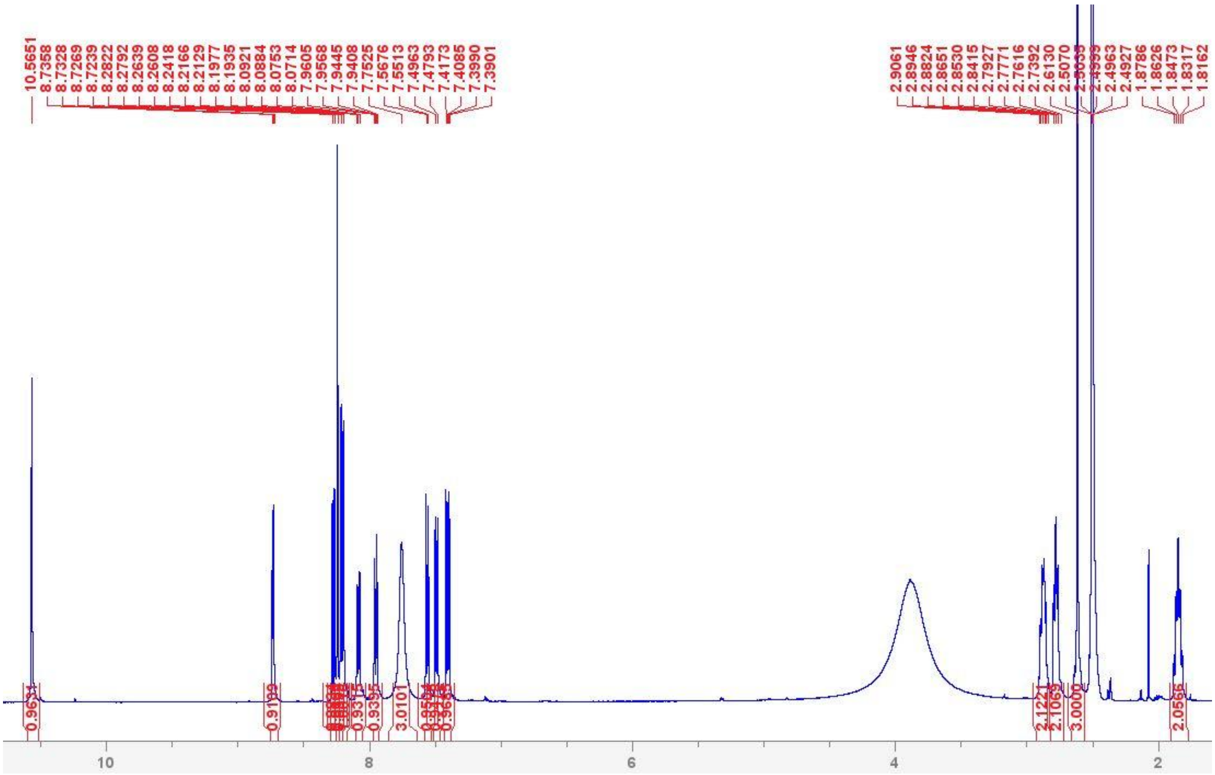

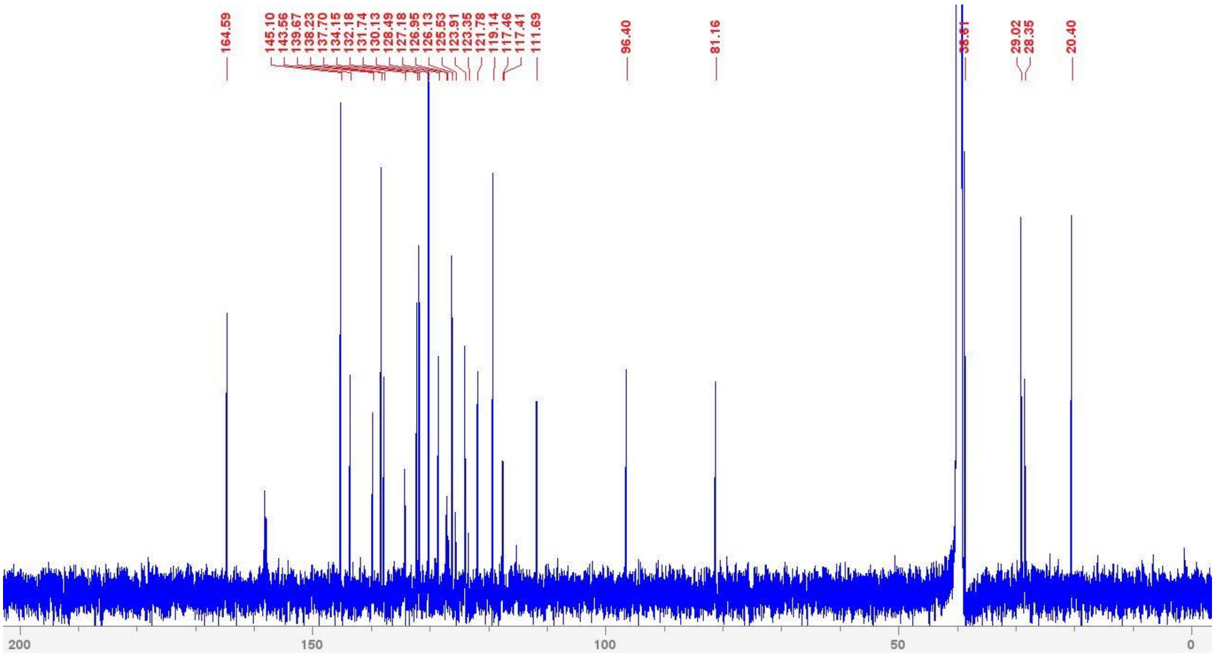

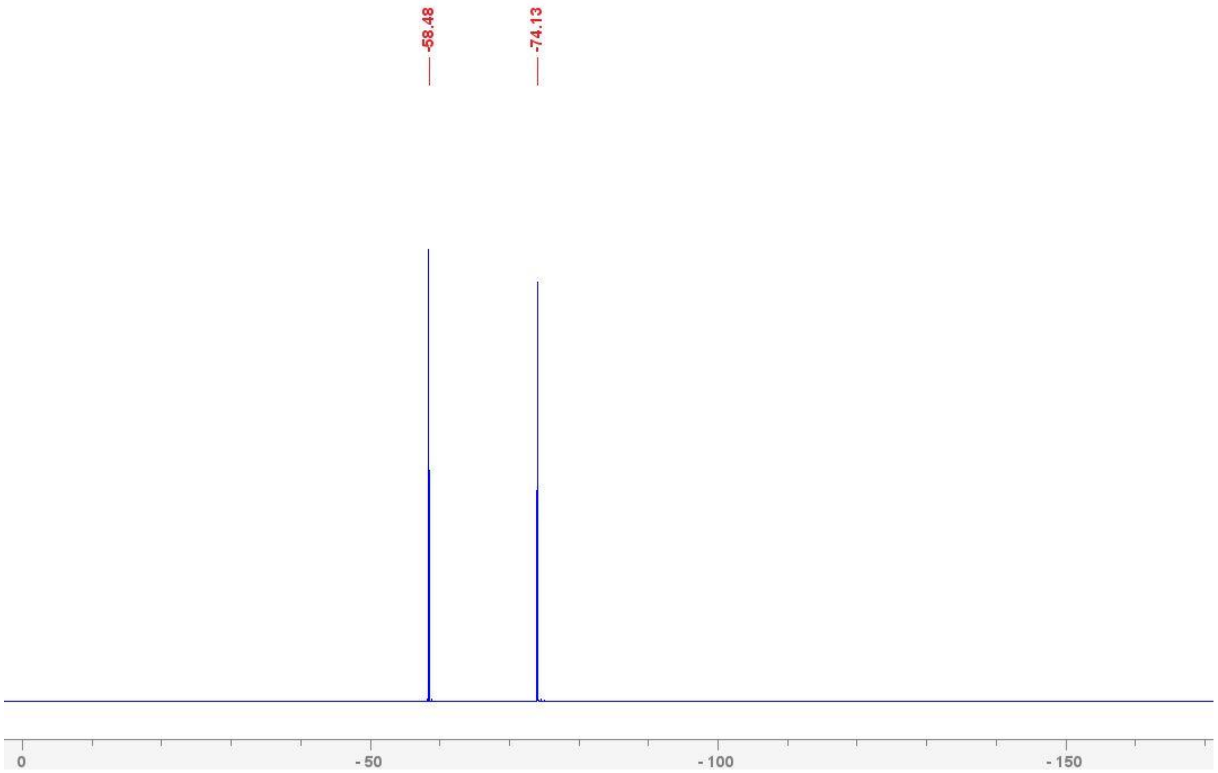
-58.48
-74.13
0
- 50
- 100
- 150

N-(4-(3-(1,3-Dioxoisoindolin-2-yl)propyl)-3-(trifluoromethyl)phenyl)-3-(imidazo[1,2-*b*]pyridazin-3-ylethynyl)-4-methylbenzamide (**10a**)

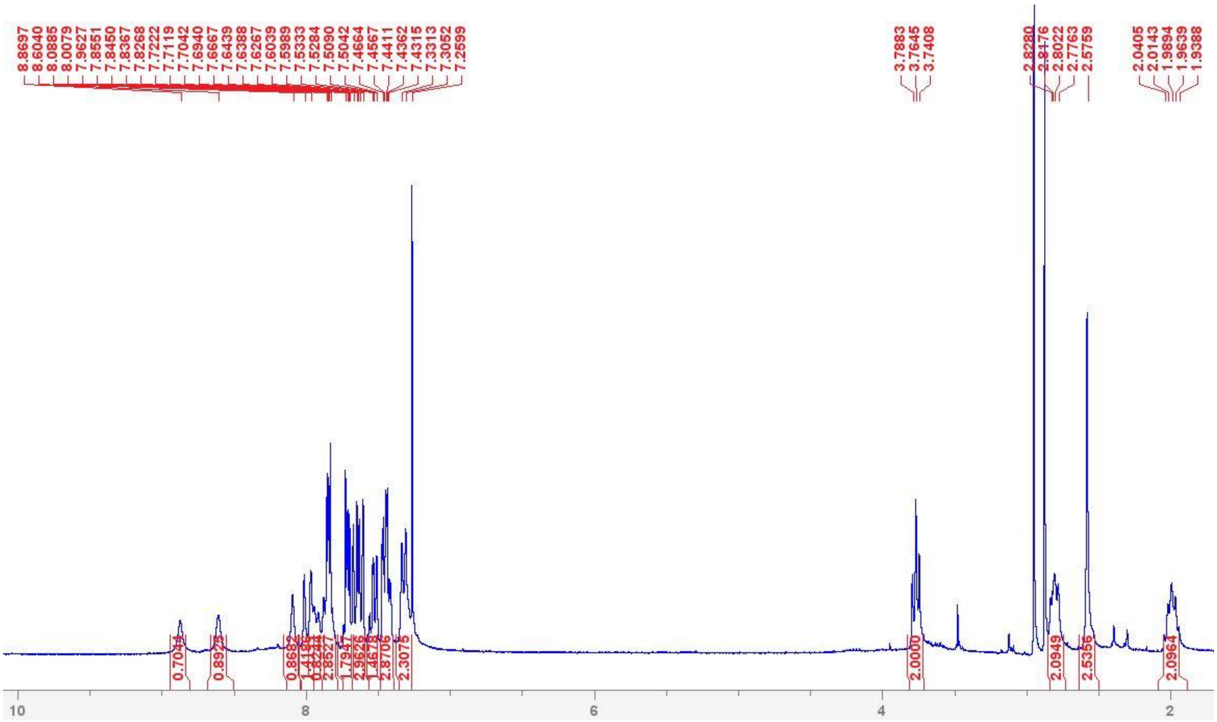

N-(4-(3-aminopropyl)-3-(trifluoromethyl)phenyl)-3-(imidazo[1,2-*b*]pyridazin-3-ylethynyl)-6-methylbenzamide (**11**)

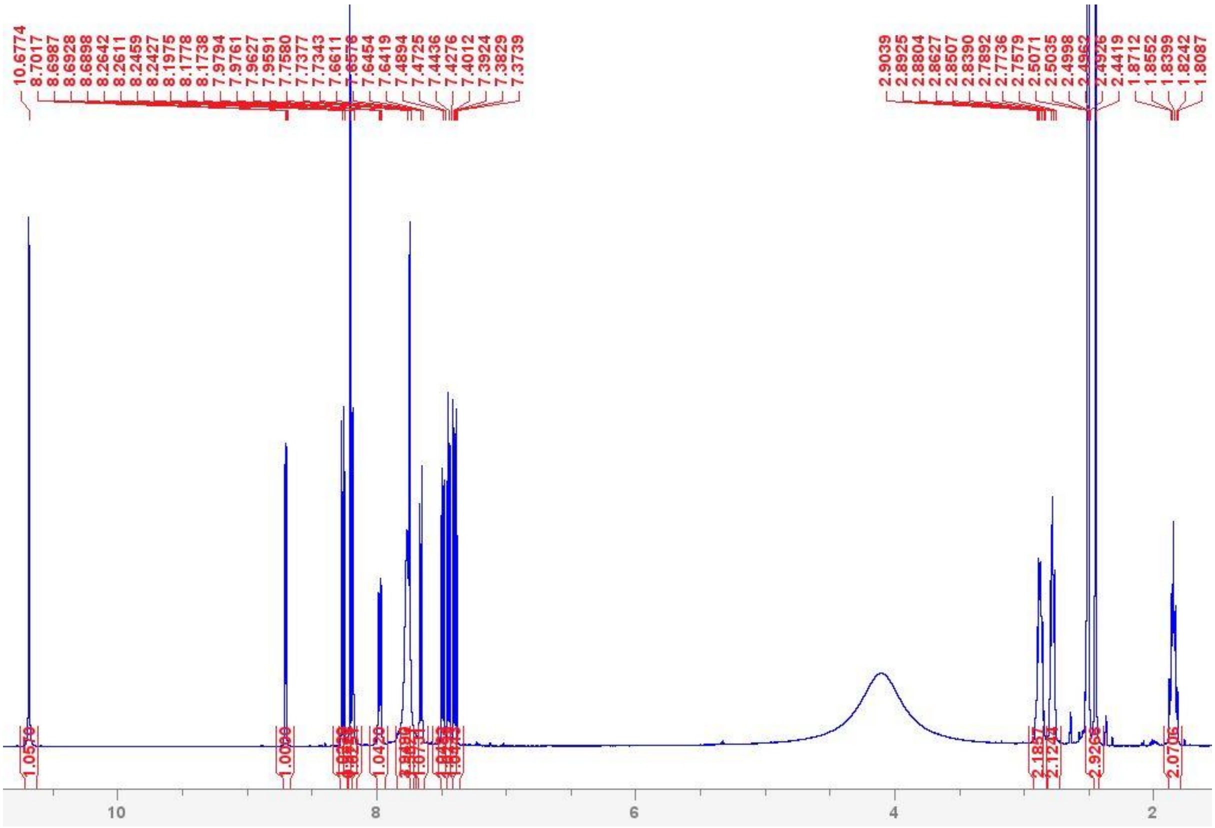

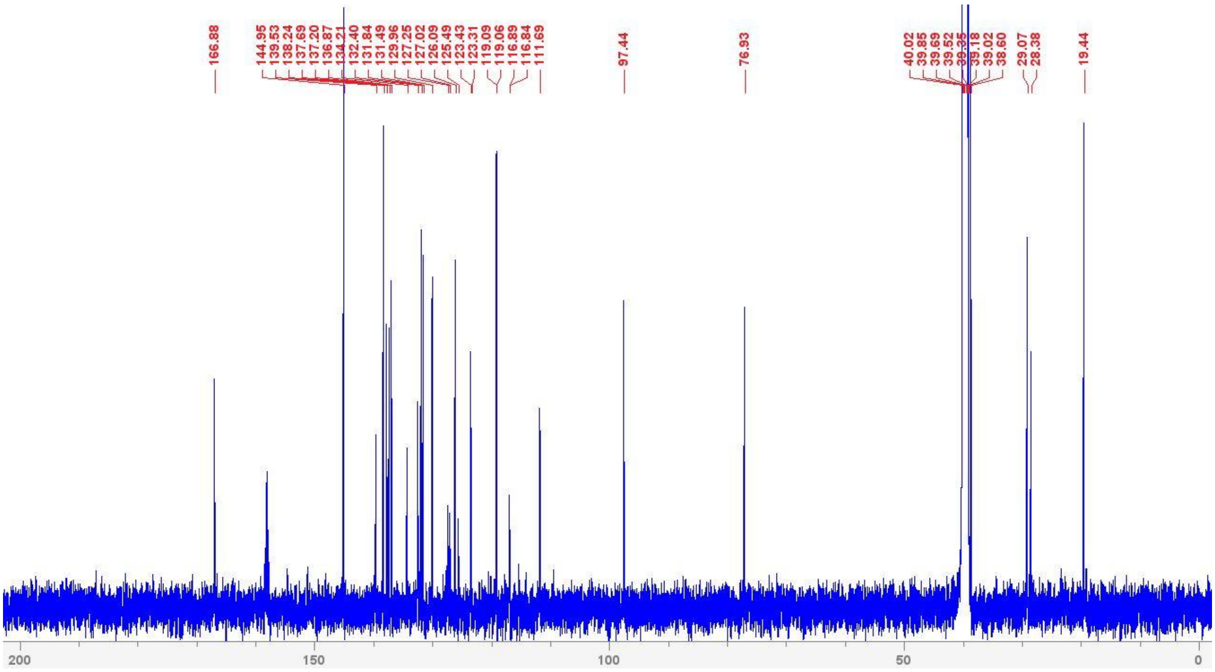

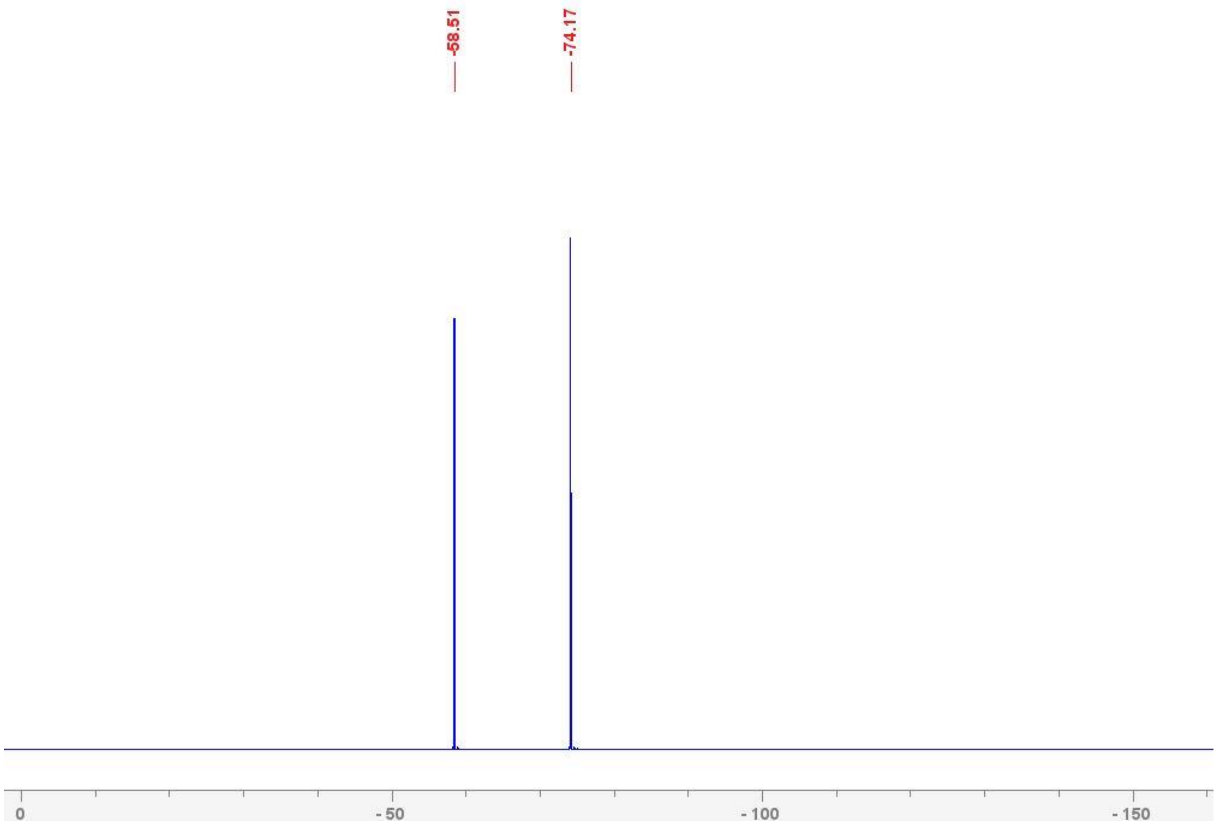

-58.51
-74.17
0
- 50
- 100
- 150

N-(4-(3-(1,3-Dioxoisoindolin-2-yl)propyl)-3-(trifluoromethyl)phenyl)-3-(imidazo[1,2-*b*]pyridazin-3-ylethynyl)-6-methylbenzamide (**11a**)

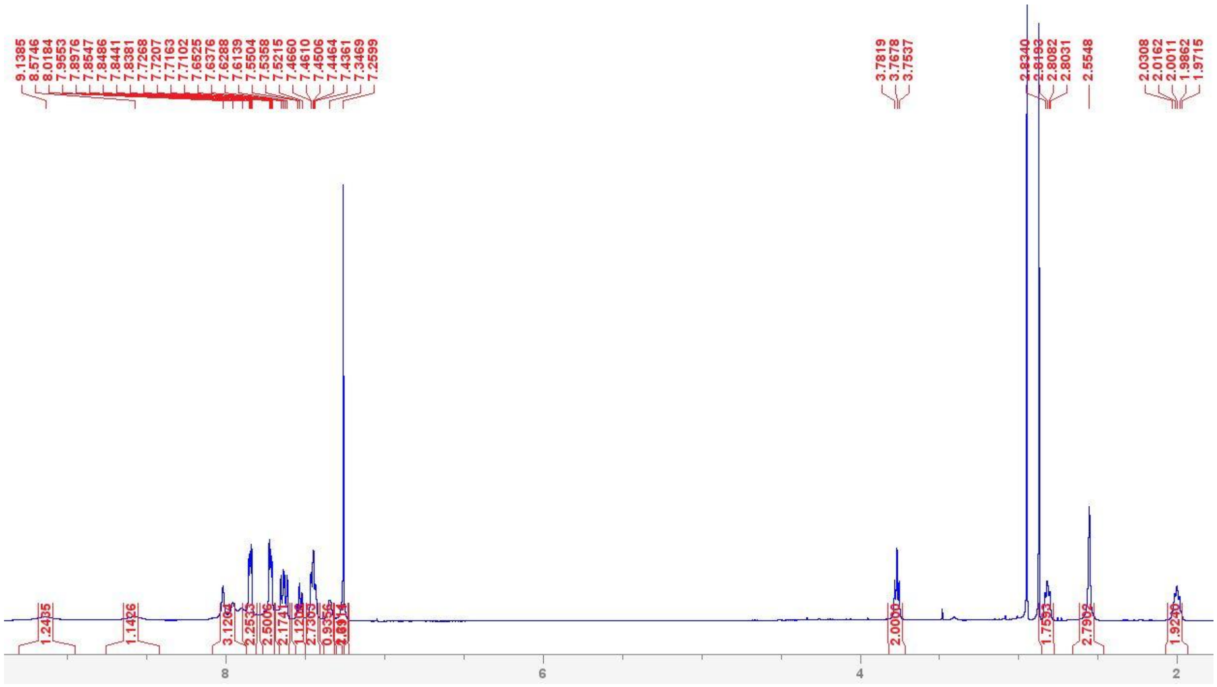

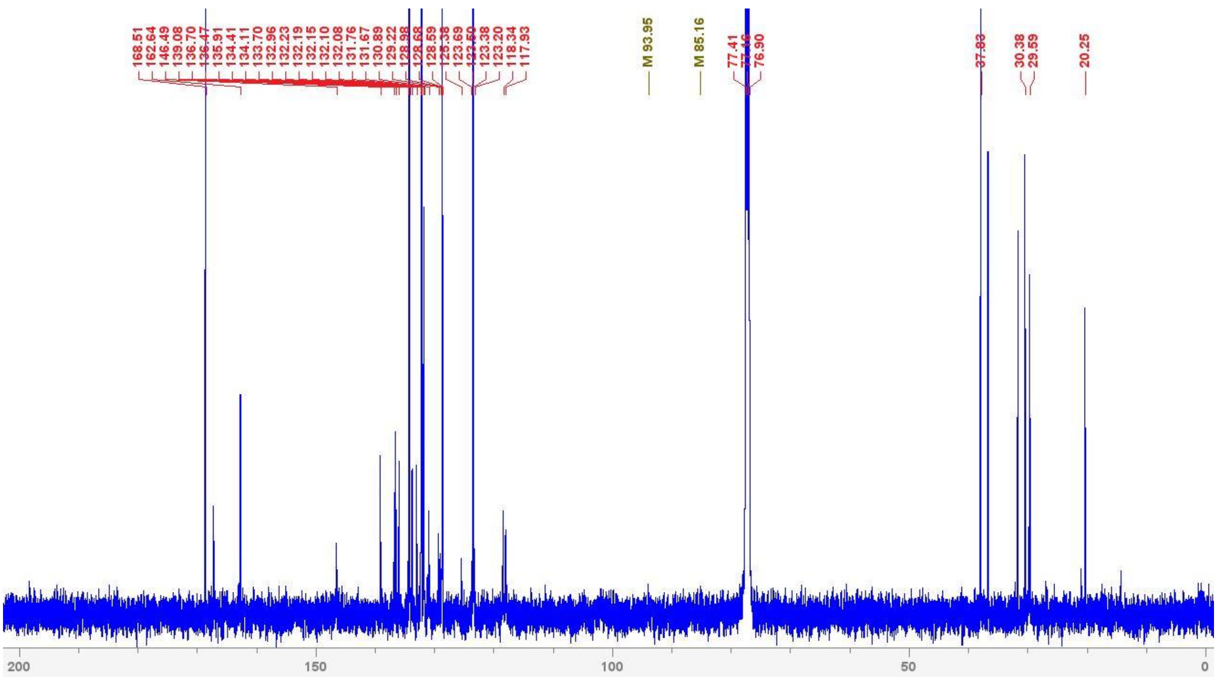

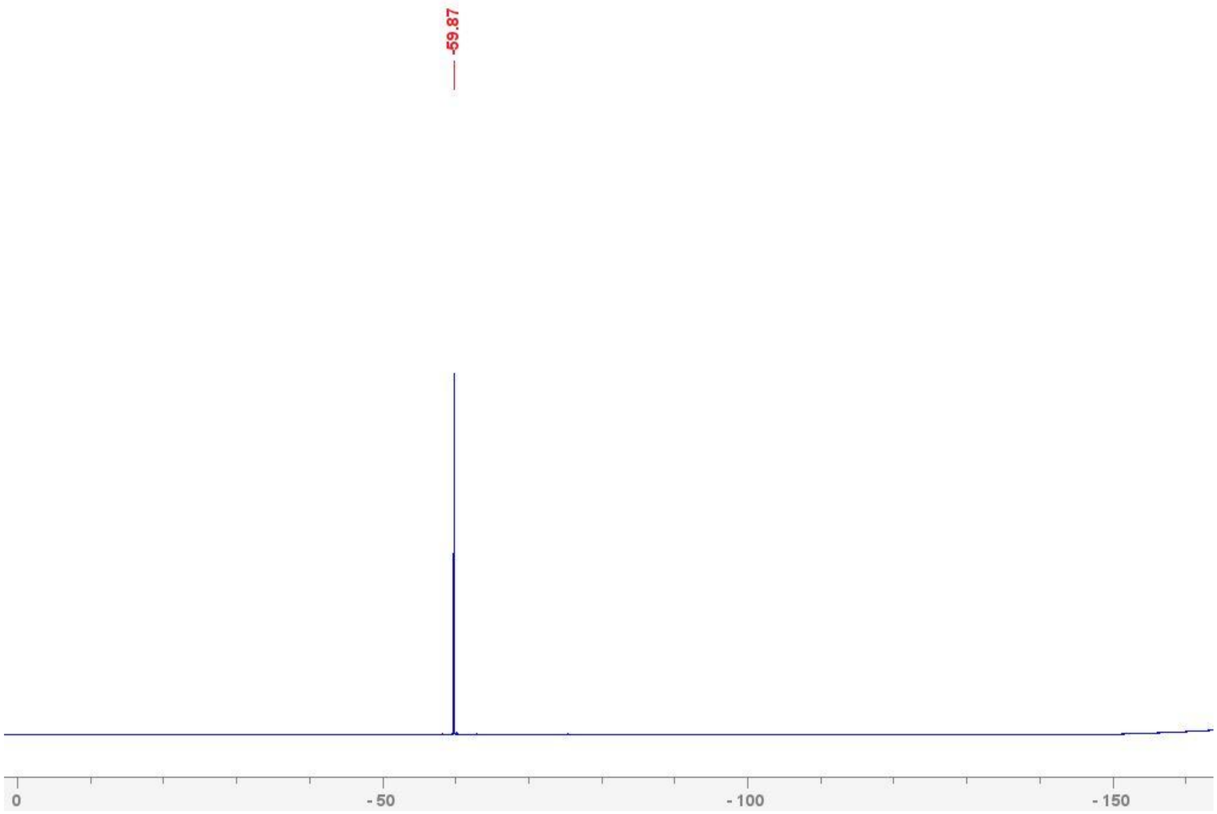
-59.87
0
- 50
- 100
- 150

N-(4-(3-aminopropyl)-3-(trifluoromethyl)phenyl)-3-(imidazo[1,2-*b*]pyridazin-3-ylethynyl)-2,4-dimethylbenzamide (**12**)

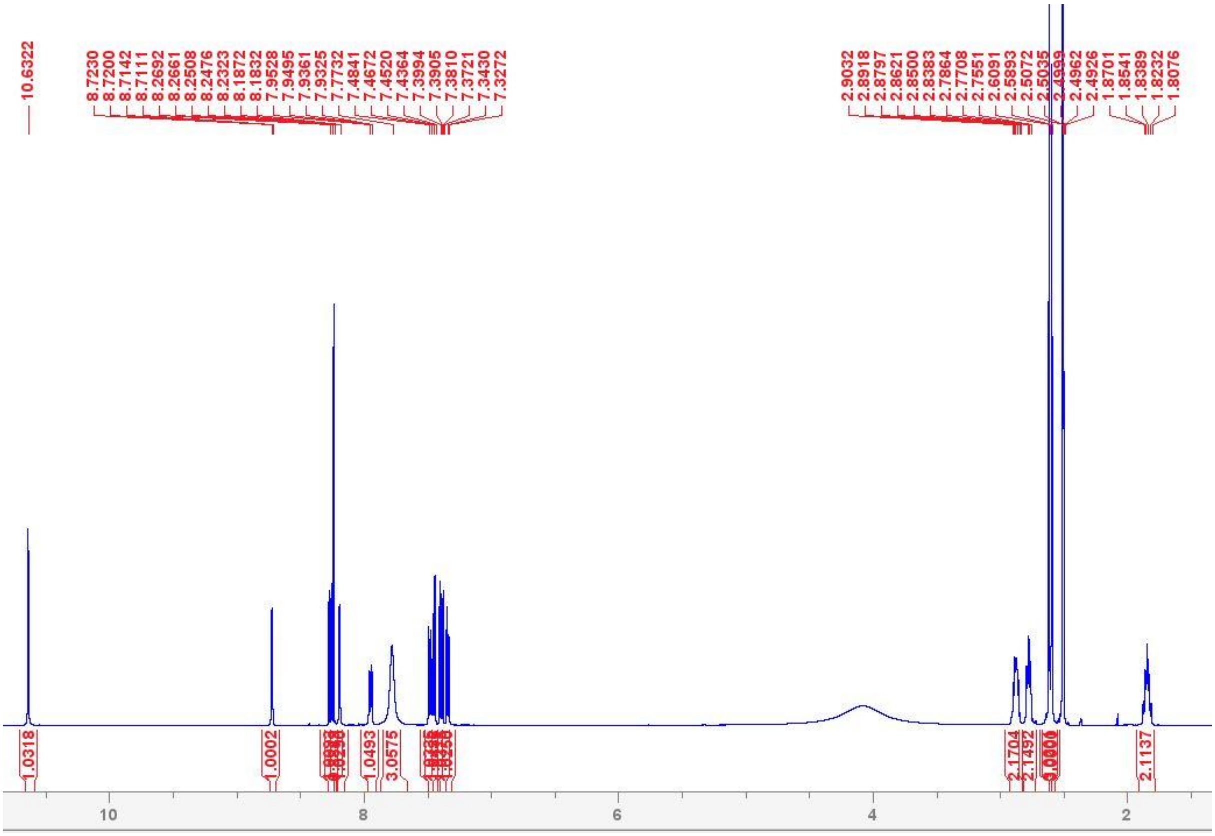

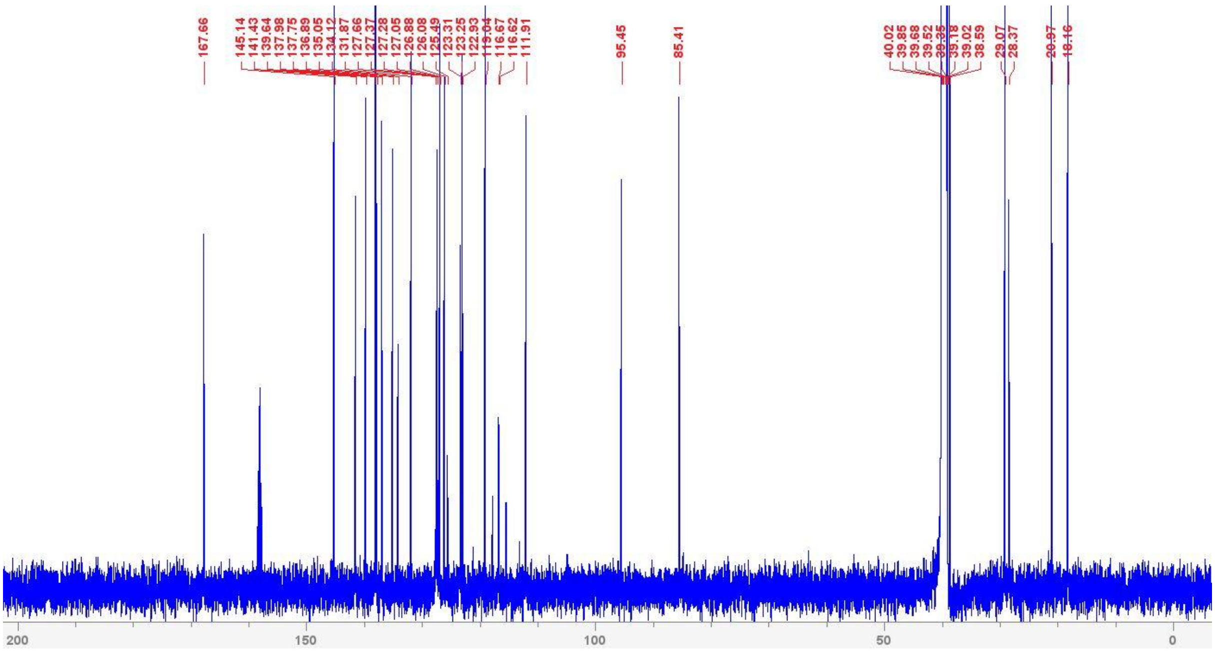

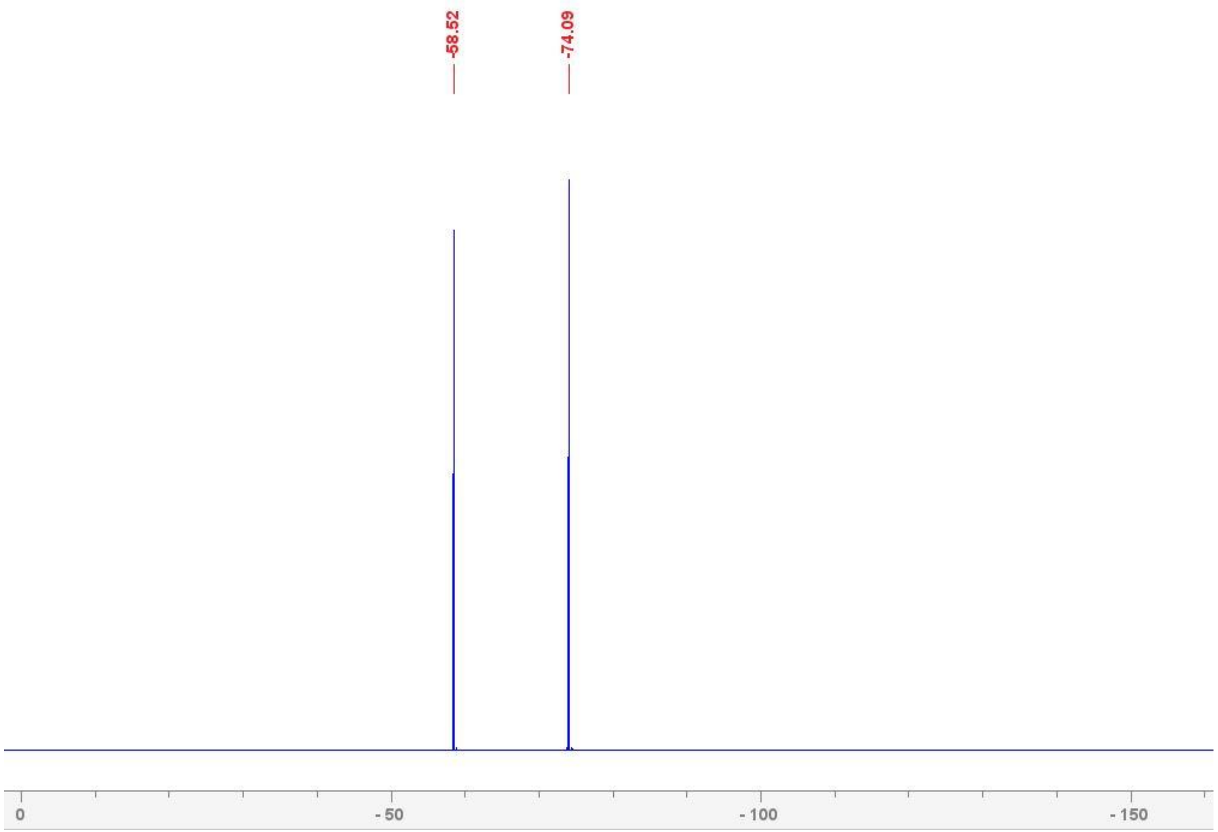
-58.52
-74.09
0
- 50
- 100
- 150

N-(4-(3-(1,3-Dioxoisoindolin-2-yl)propyl)-3-(trifluoromethyl)phenyl)-3-(imidazo[1,2-*b*]pyridazin-3-ylethynyl)-2,4-dimethylbenzamide (**12a**)

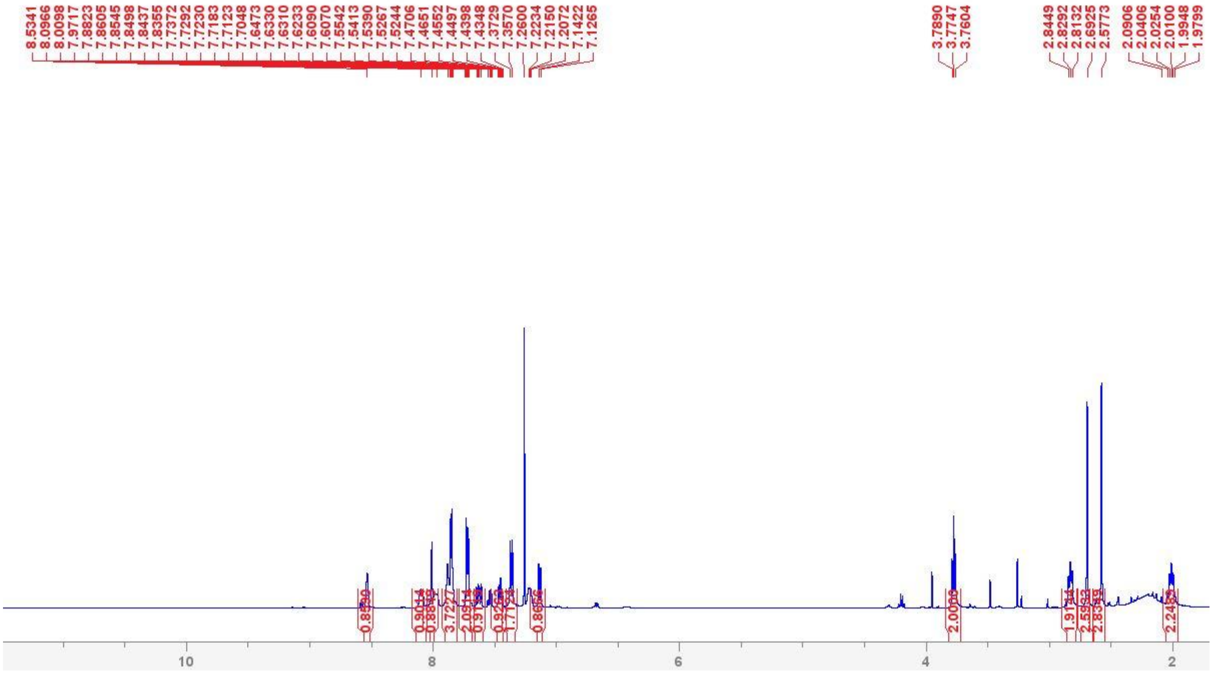

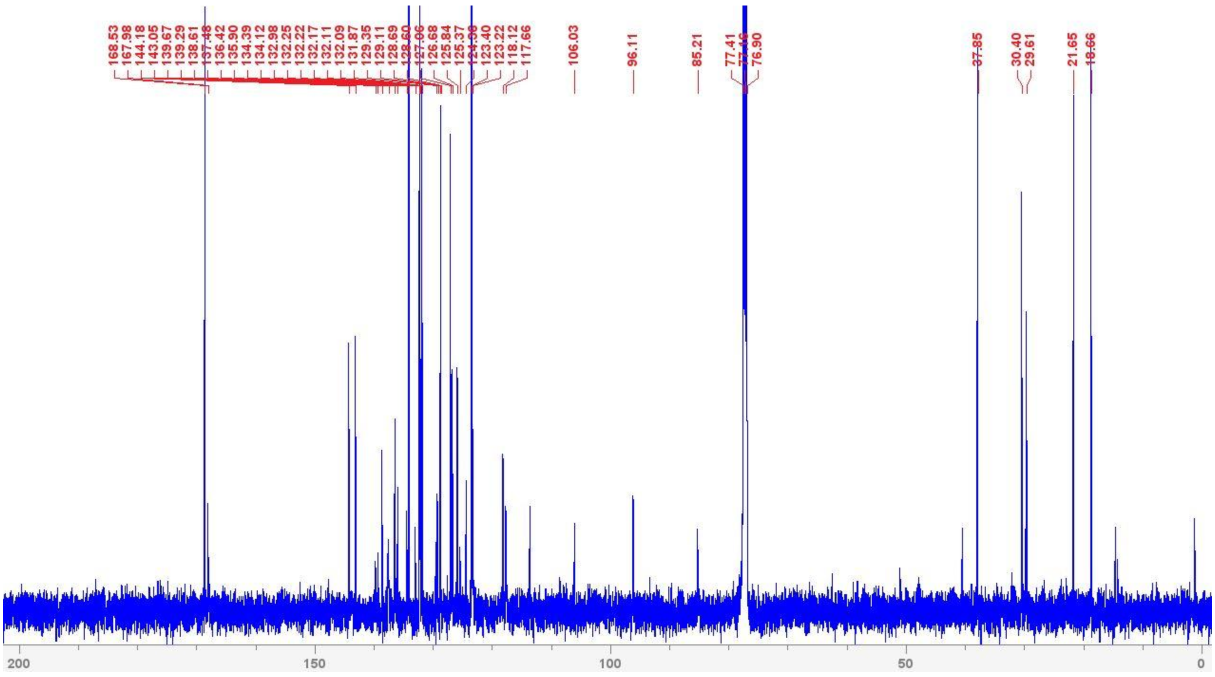

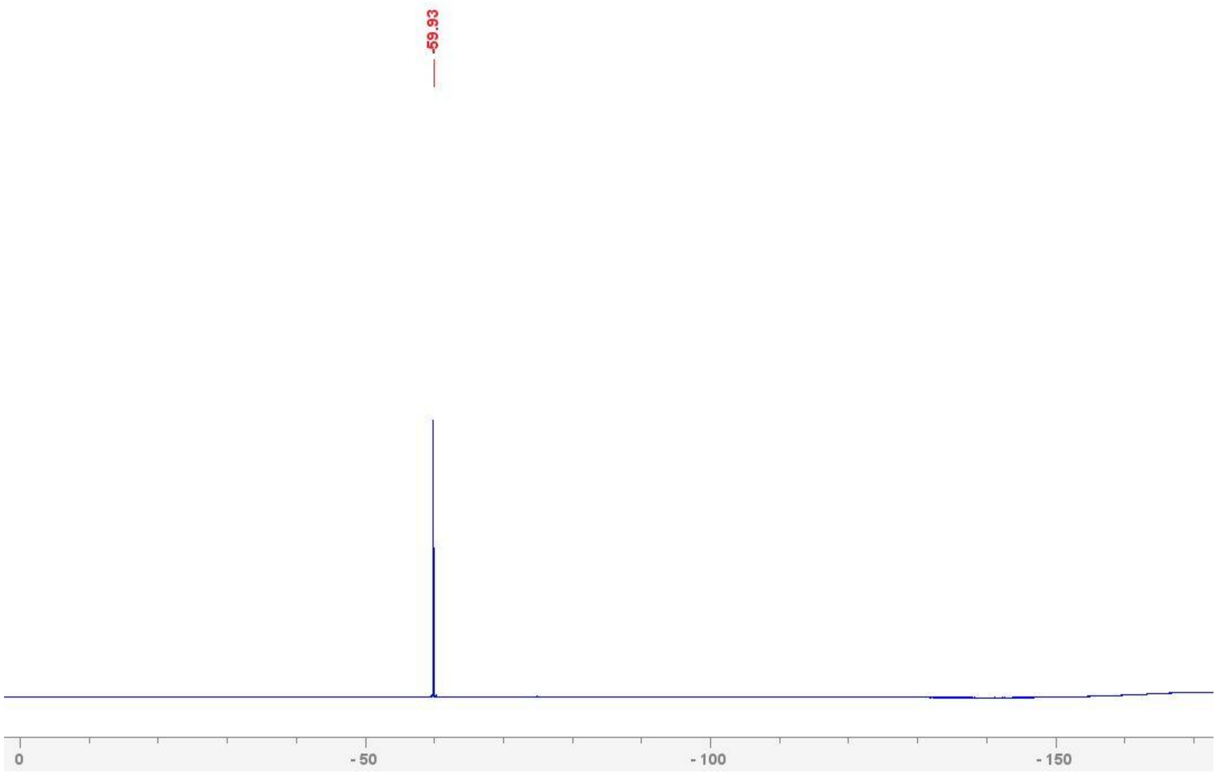
-59.93
0
- 50
- 100
- 150

3-Ethynylimidazo[1,2-*b*]pyridazine (**13**)

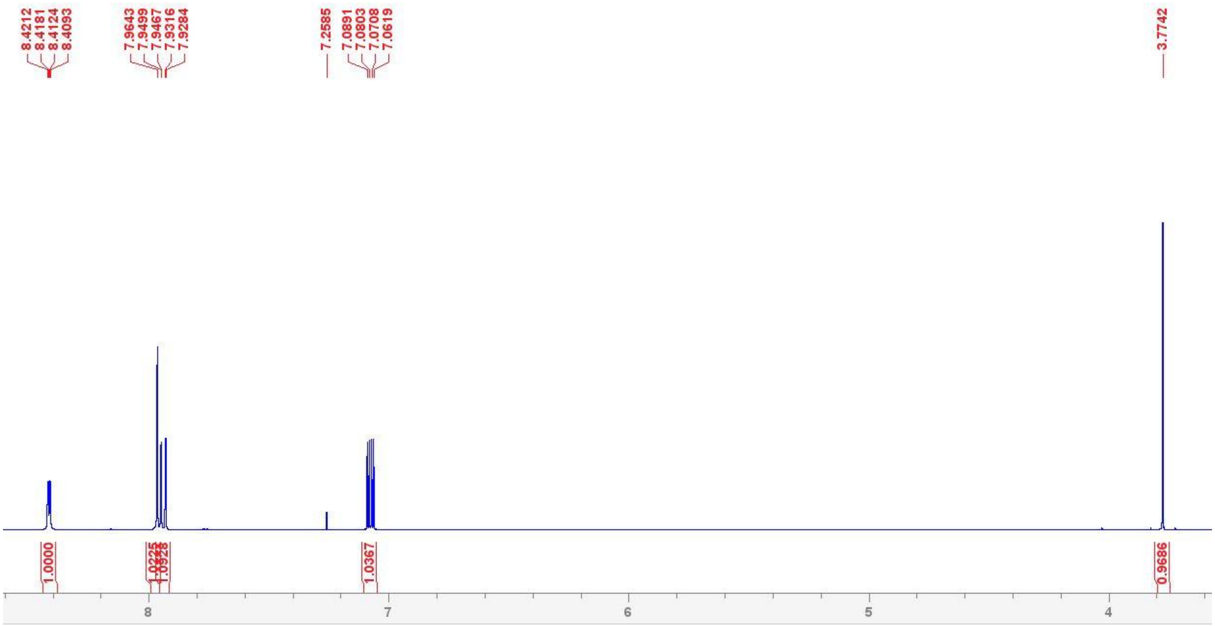

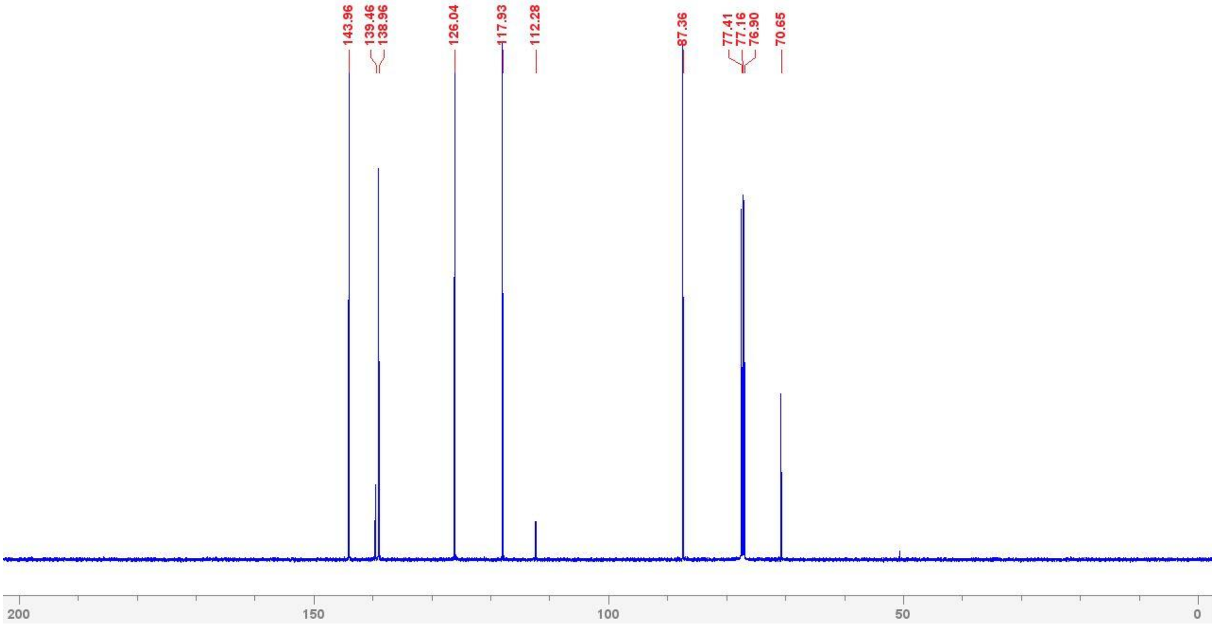

3-Iodo-2,4-dimethylbenzoic acid (**14d**)

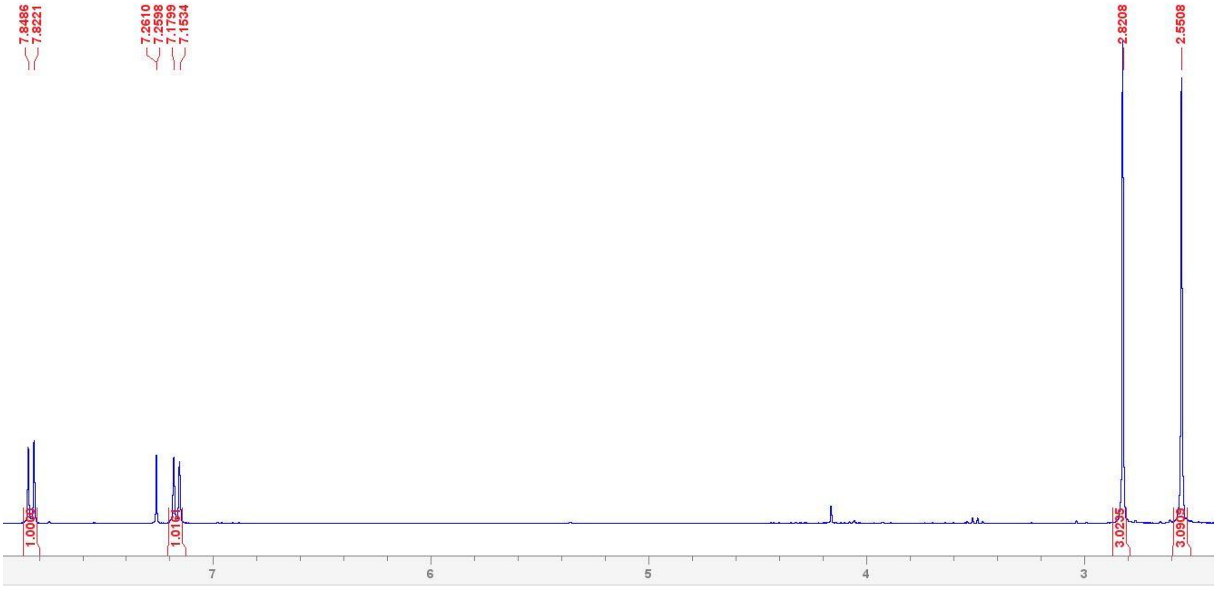

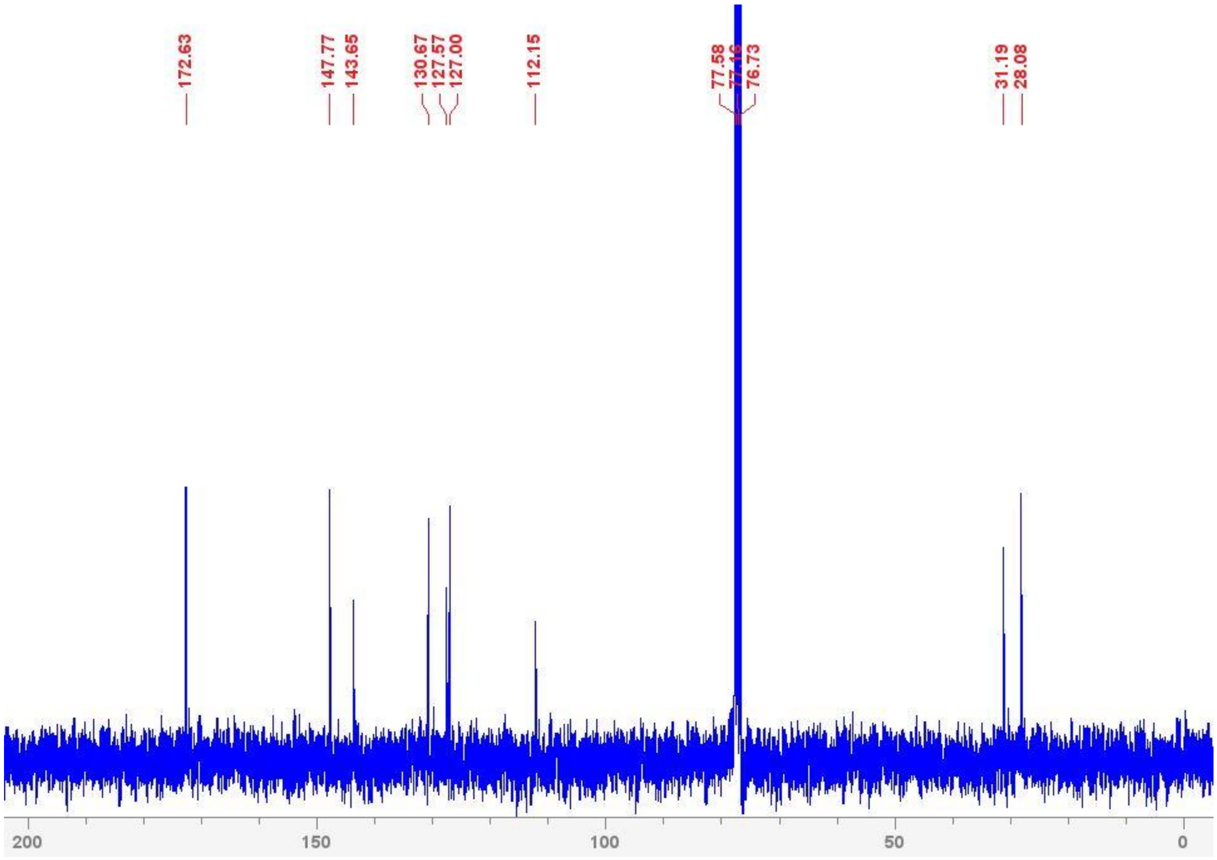

4-((4-Methylpiperazin-1-yl)methyl)-3-(trifluoromethyl)aniline (**15a**)

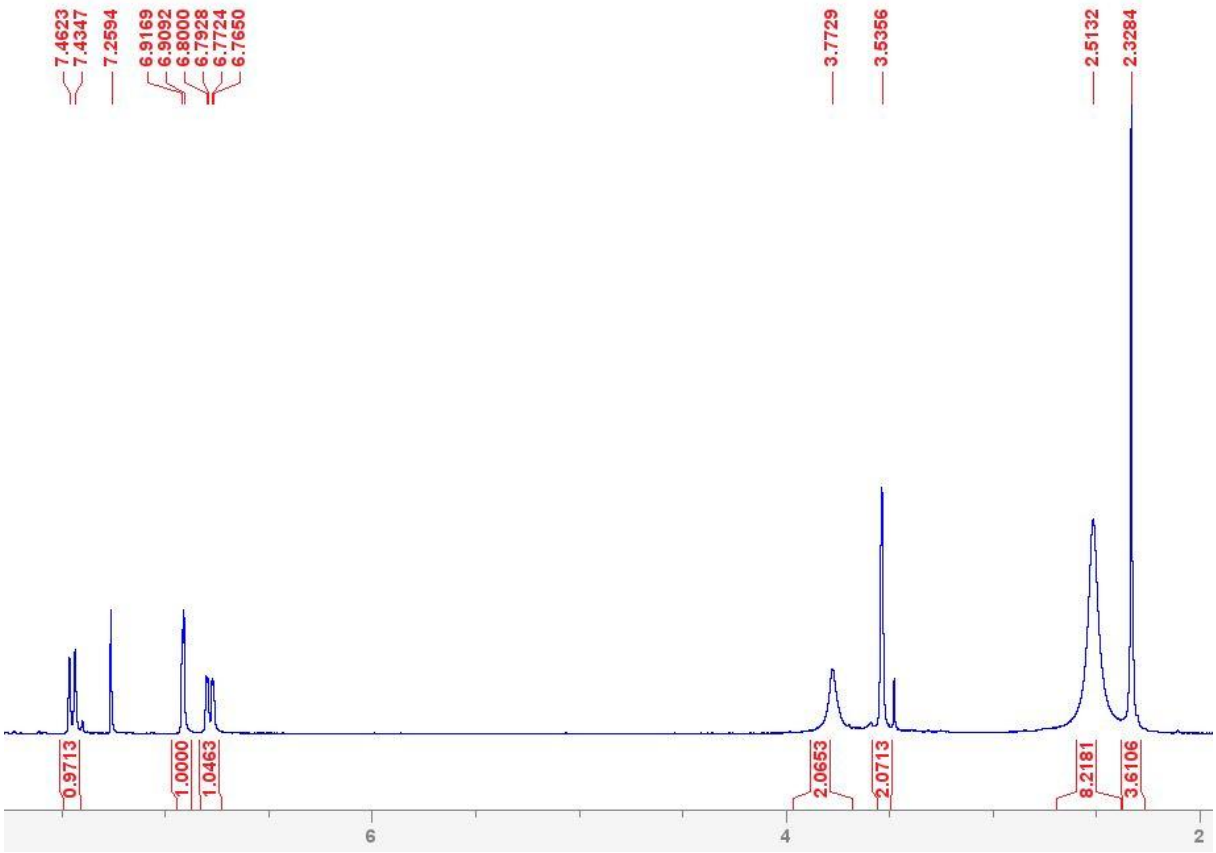

3-Bromoimidazo[1,2-*b*]pyridazine (**17**)

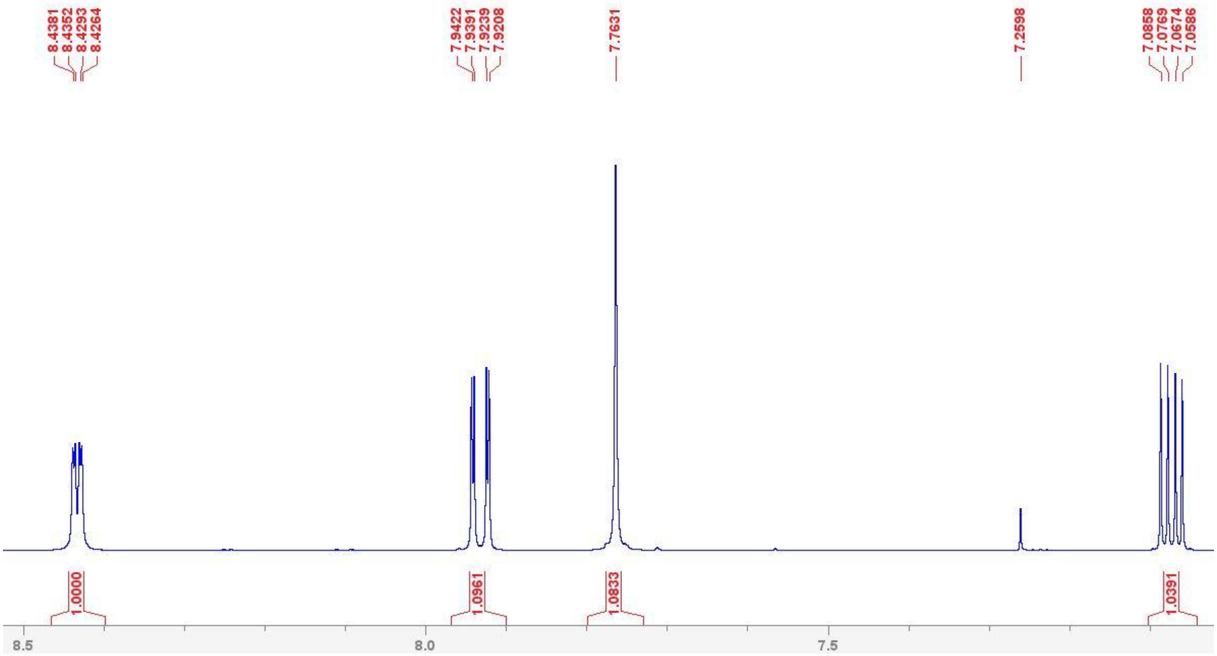

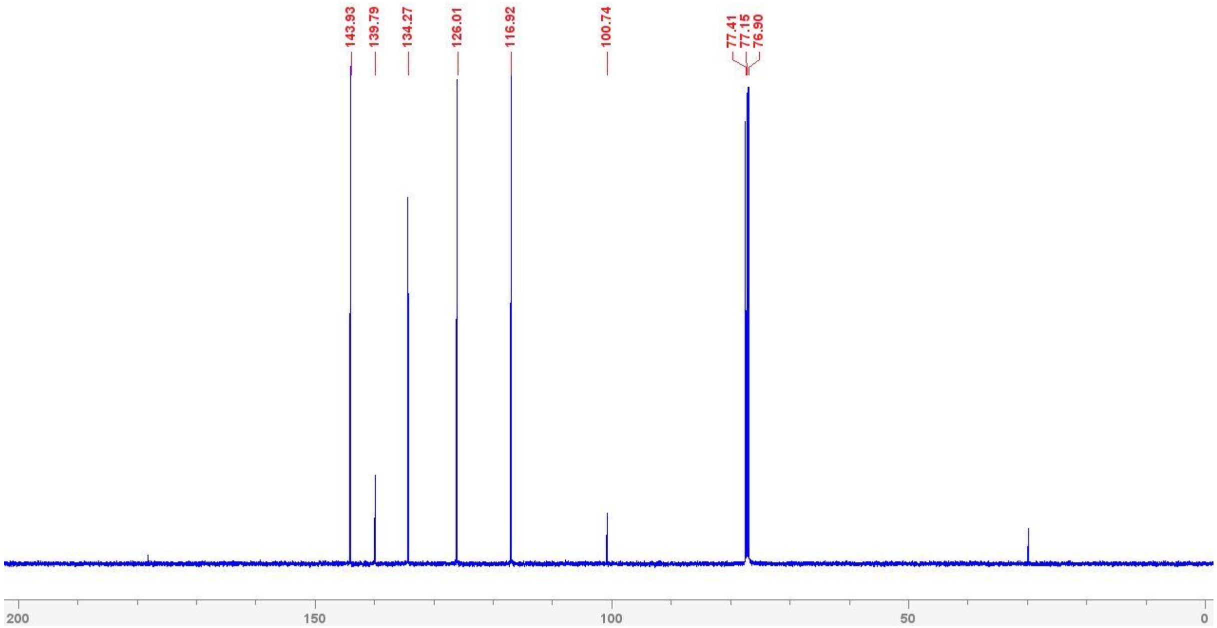

3-Iodo-2,4-dimethylbenzaldehyde (**20**)

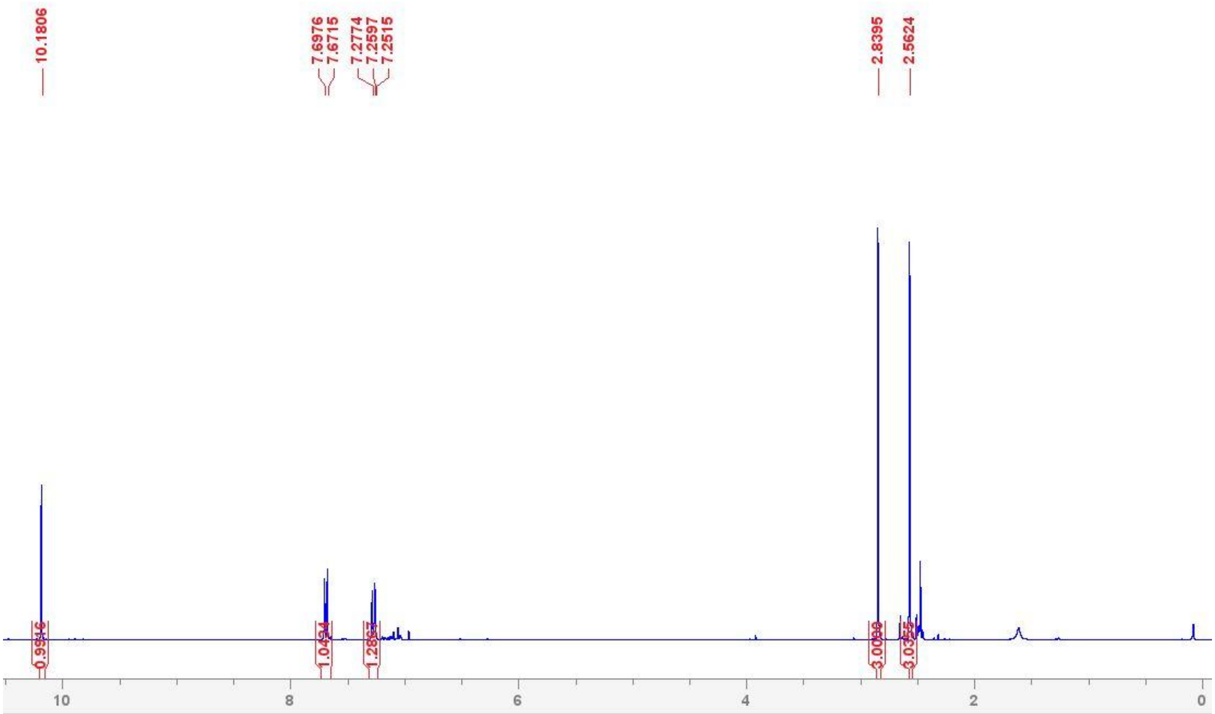

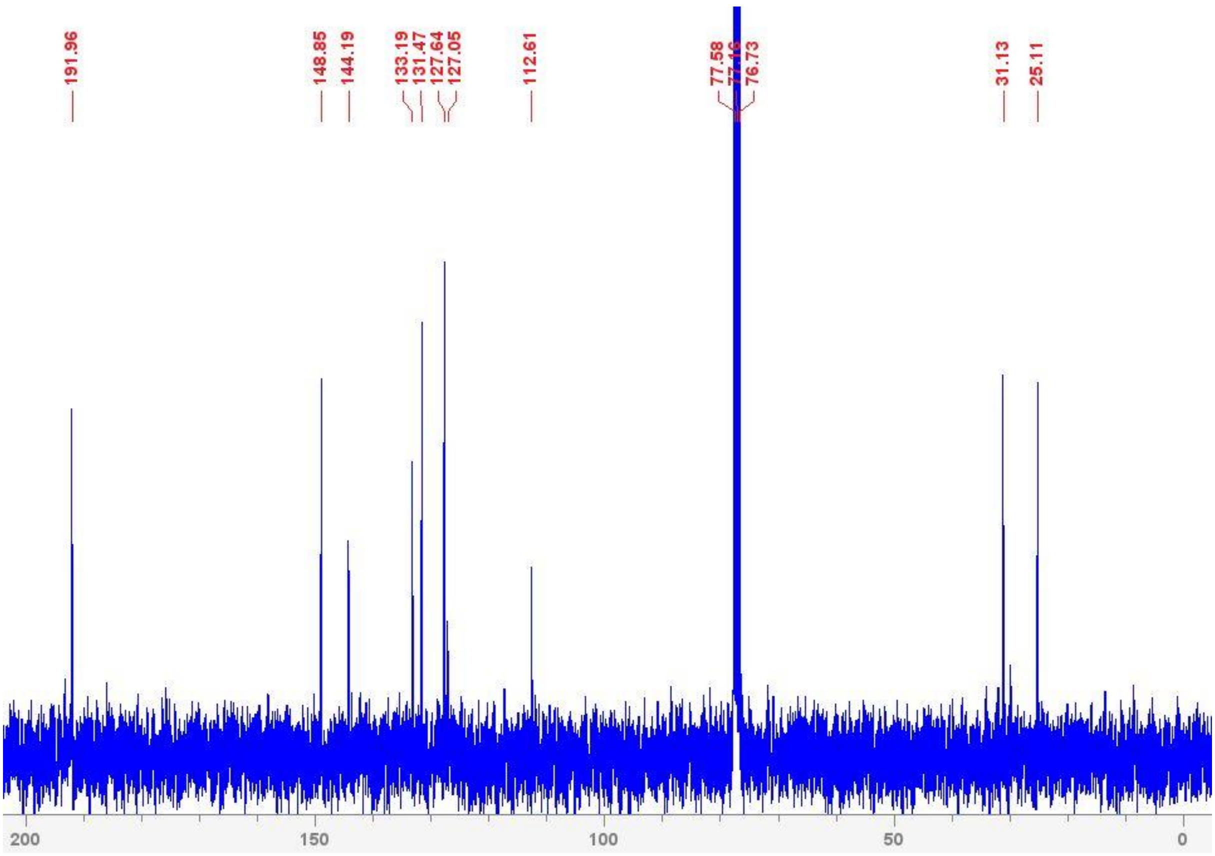

1-(Bromomethyl)-4-nitro-2-(trifluoromethyl)benzene (**22**)

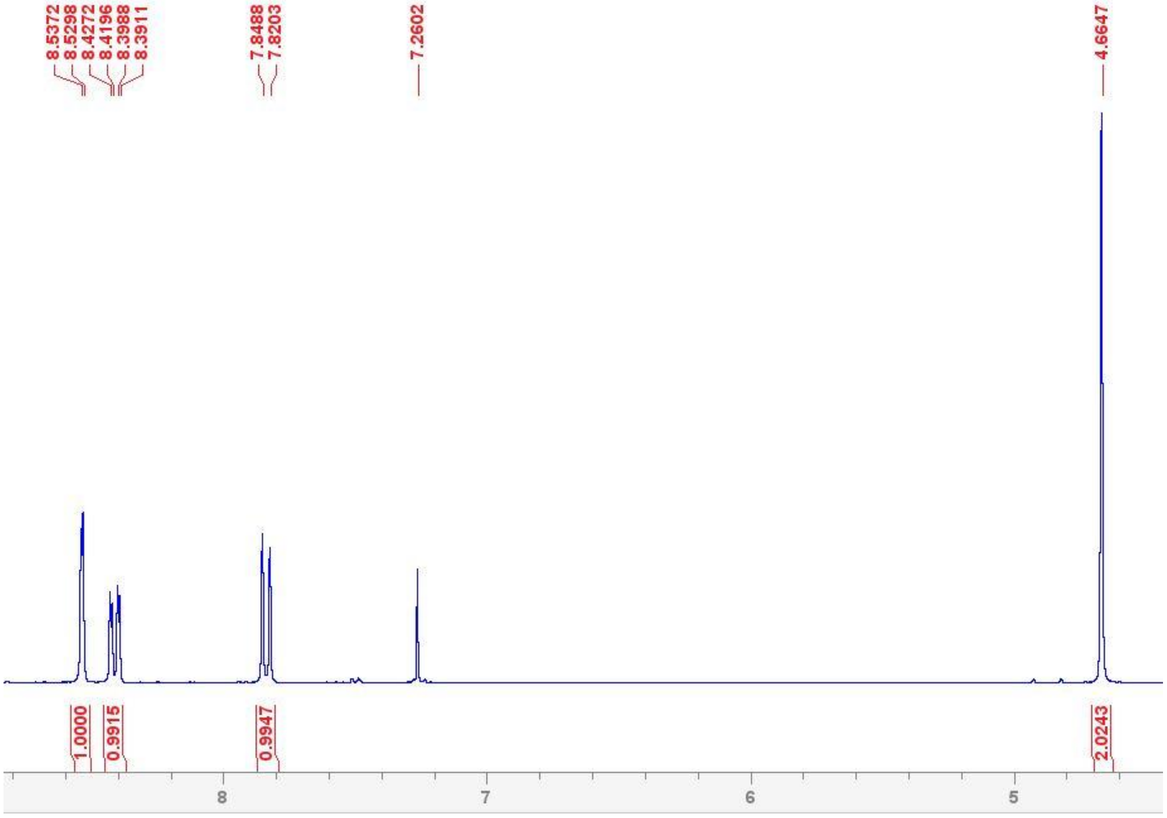

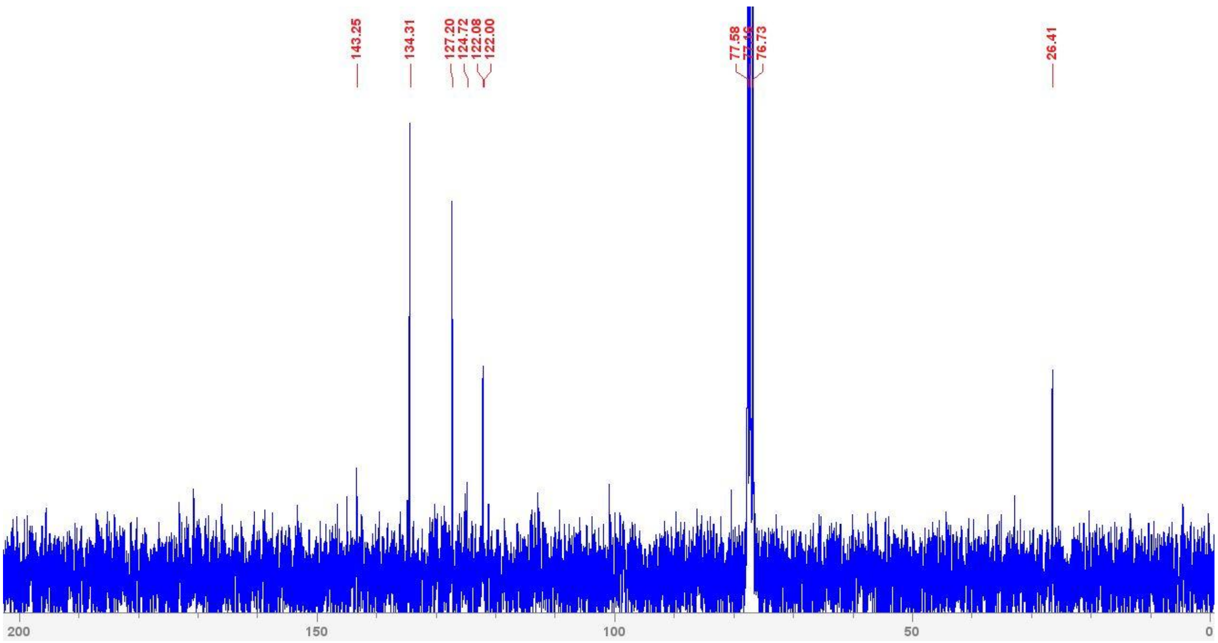

1-Methyl-4-(4-nitro-2-(trifluoromethyl)benzyl)piperazine (**23**)

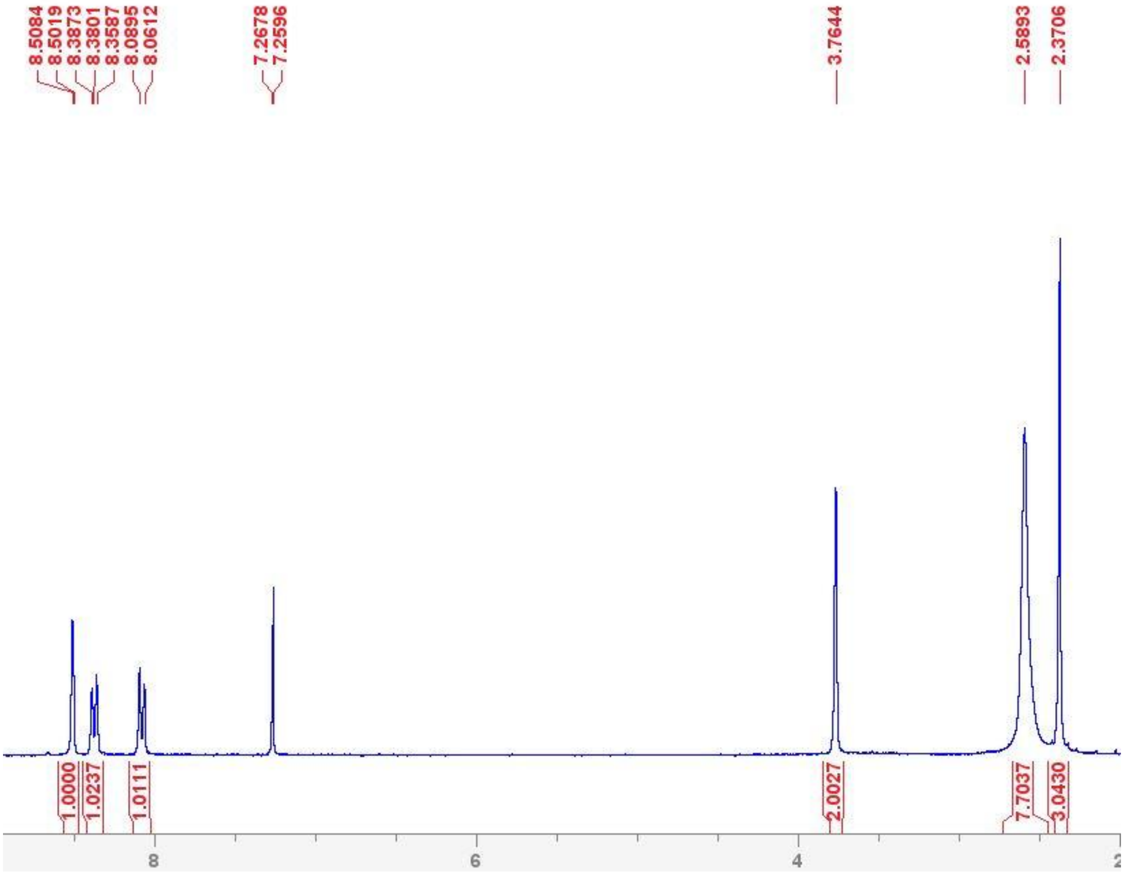

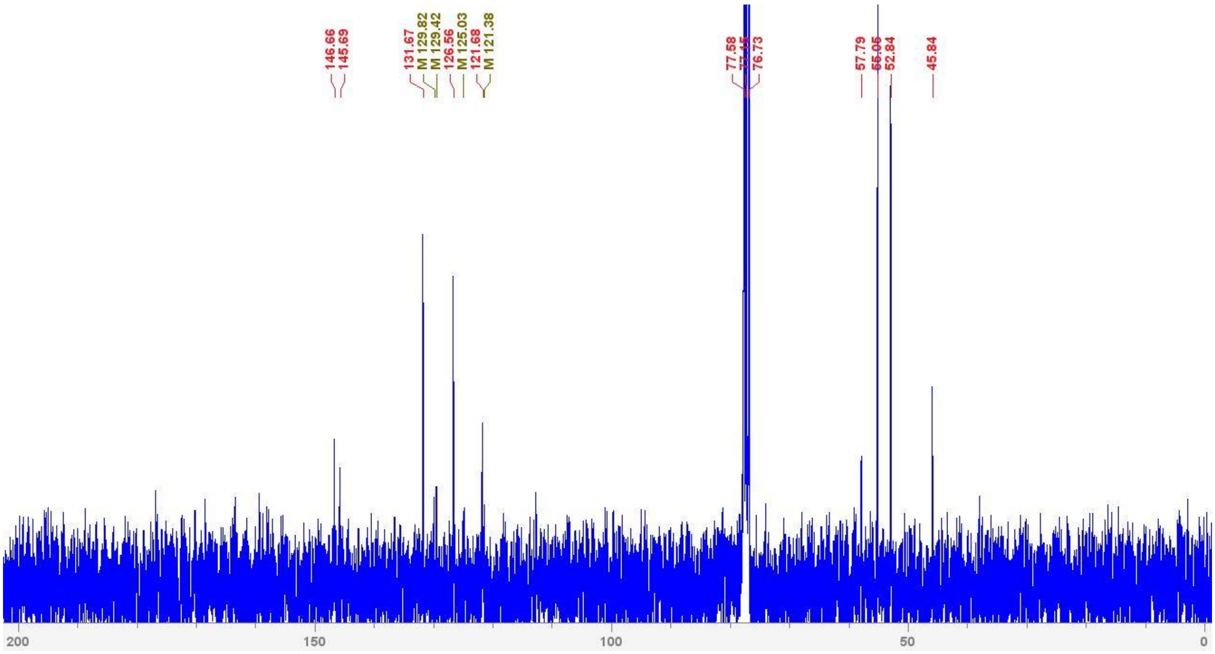

tert-Butyl (4-iodo-3-(trifluoromethyl)phenyl)carbamate (**25**)

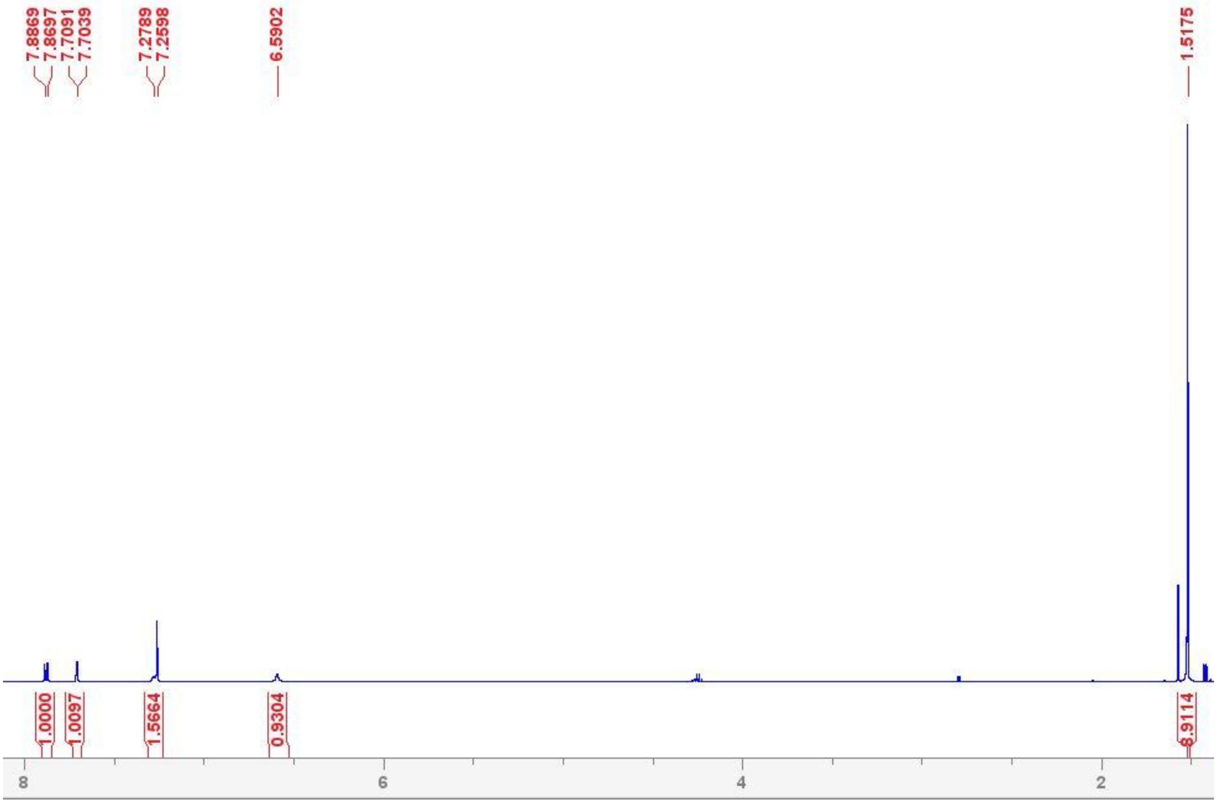

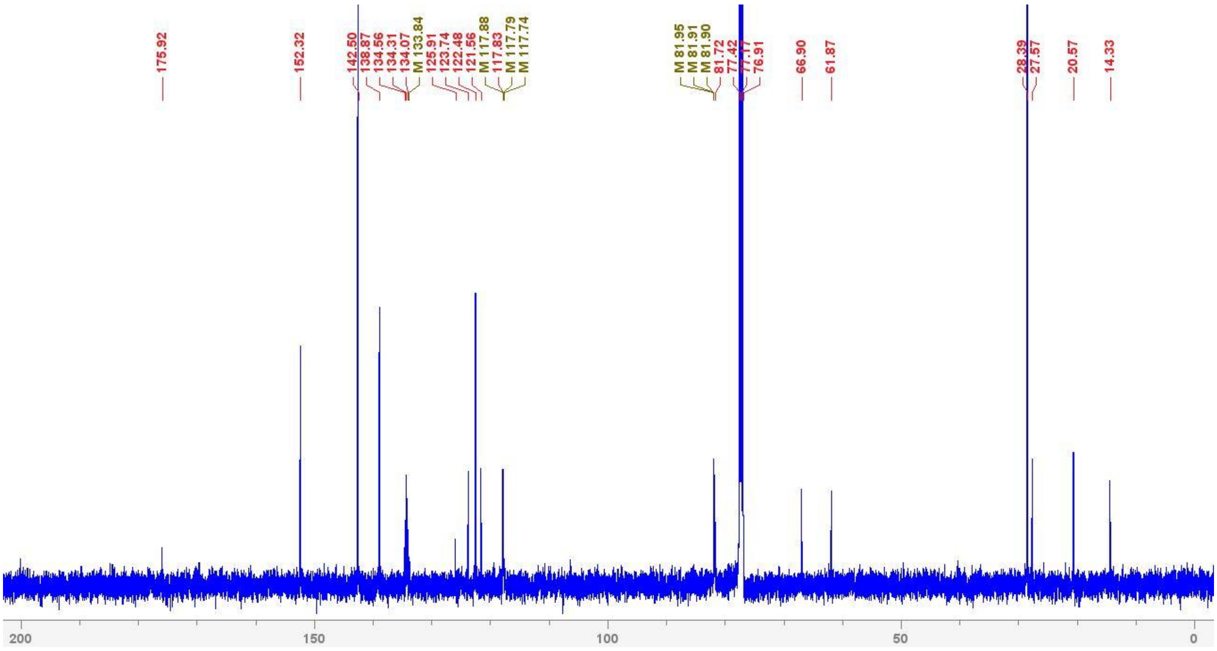

Ethyl (*E*)-3-(4-((*tert*-butoxycarbonyl)amino)-2-(trifluoromethyl)phenyl)acrylate (**26a**)

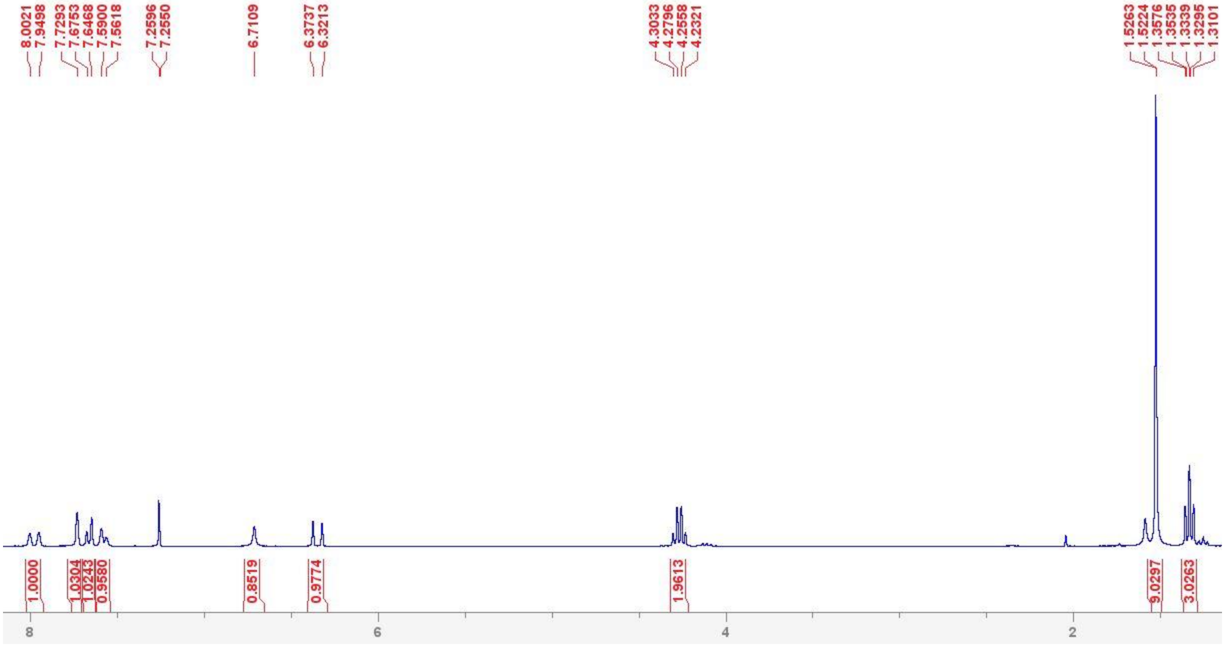

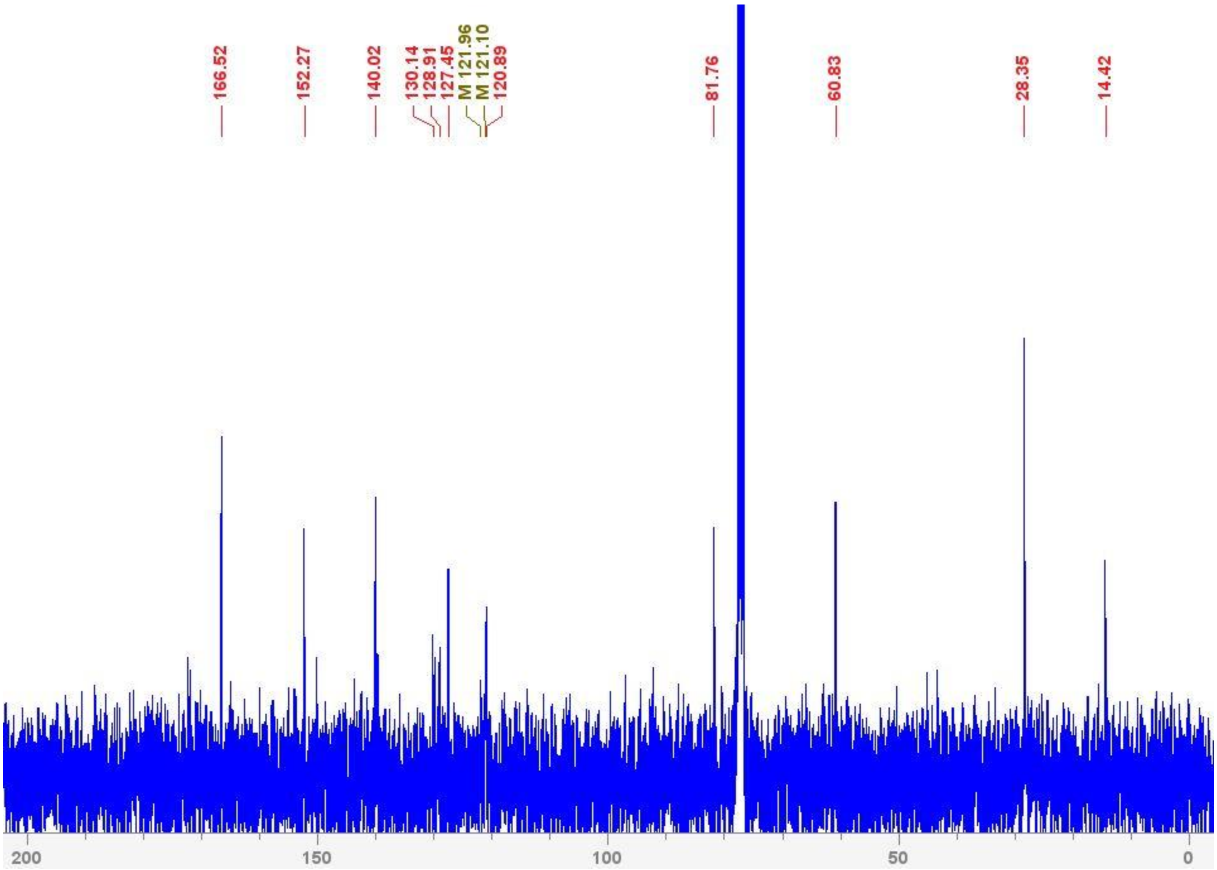

Ethyl 3-(4-((*tert*-butoxycarbonyl)amino)-2-(trifluoromethyl)phenyl)propanoate (**26b**)

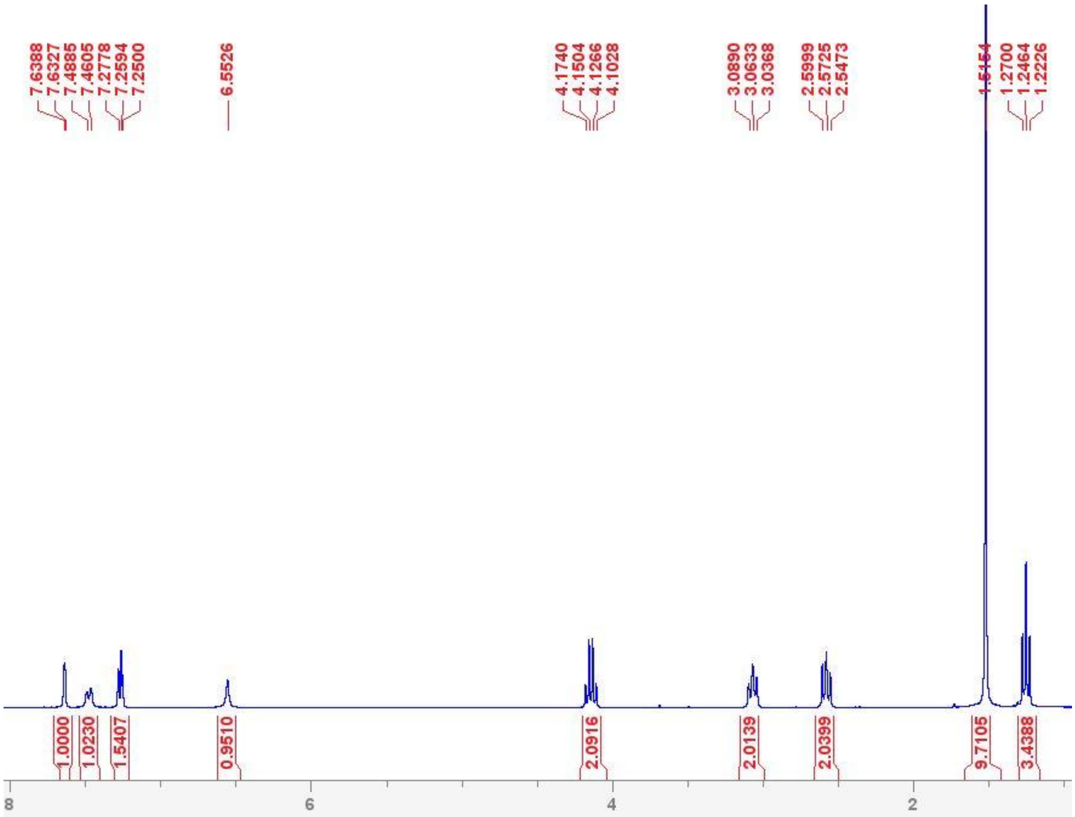

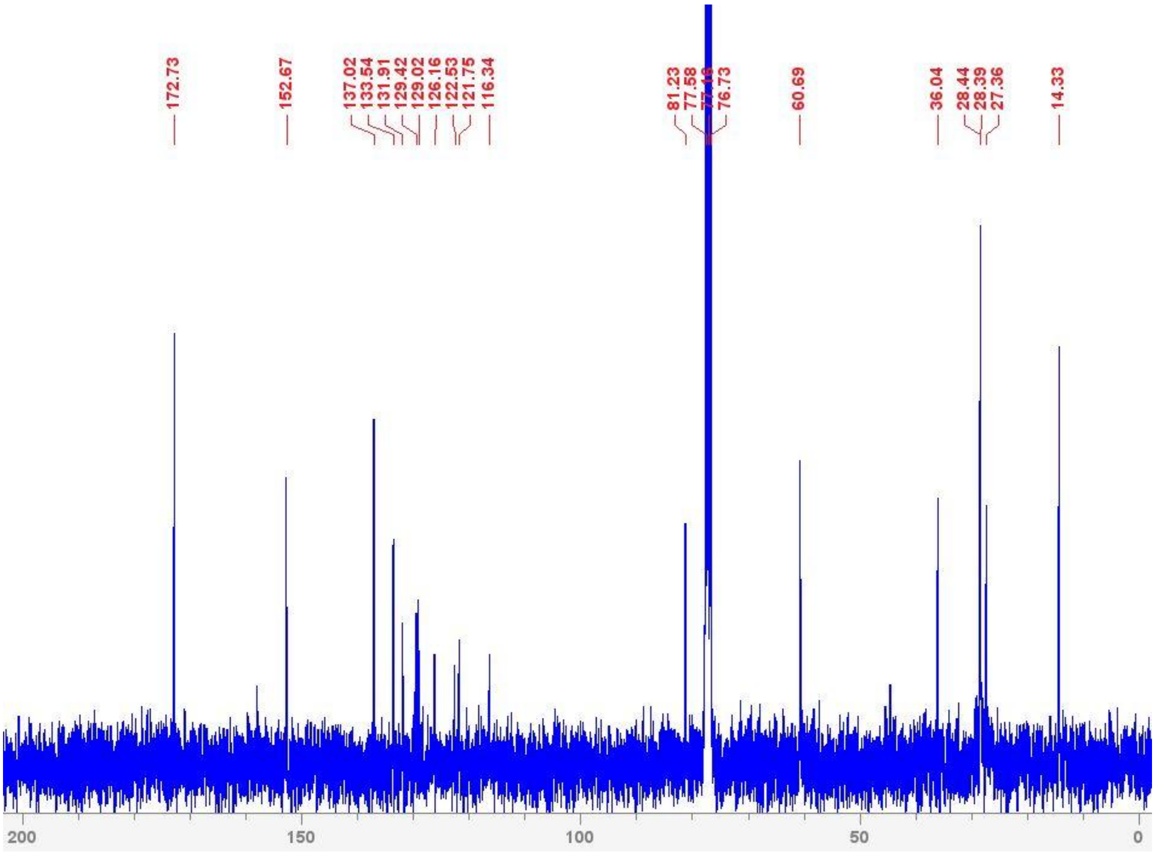

tert-Butyl (*E*)-(4-(3-hydroxyprop-1-en-1-yl)-3-(trifluoromethyl)phenyl)carbamate (**27a**)

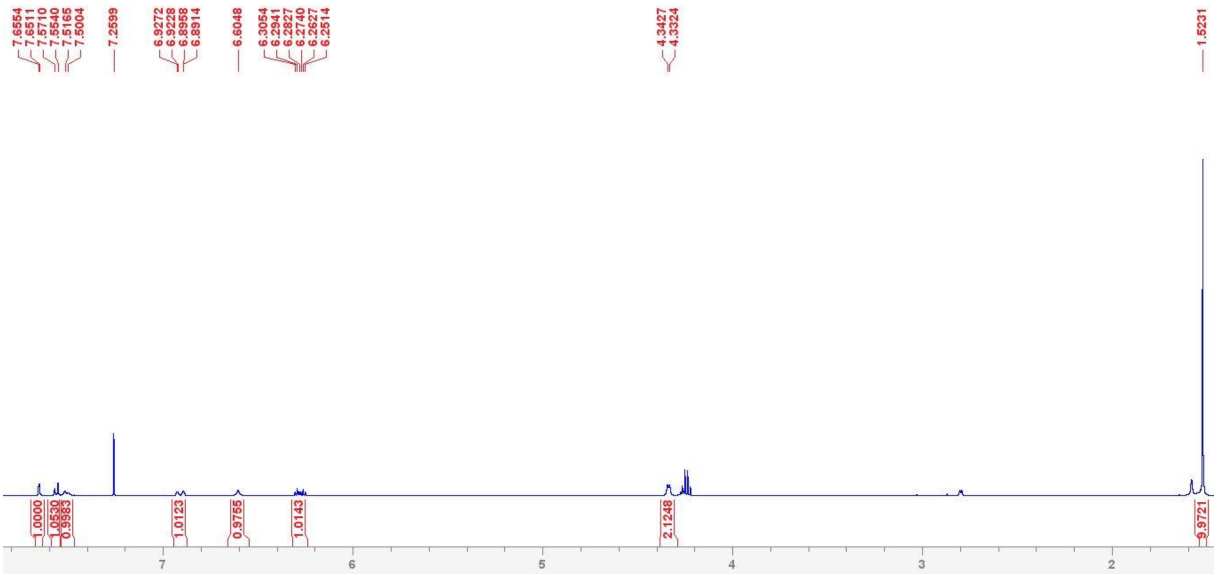

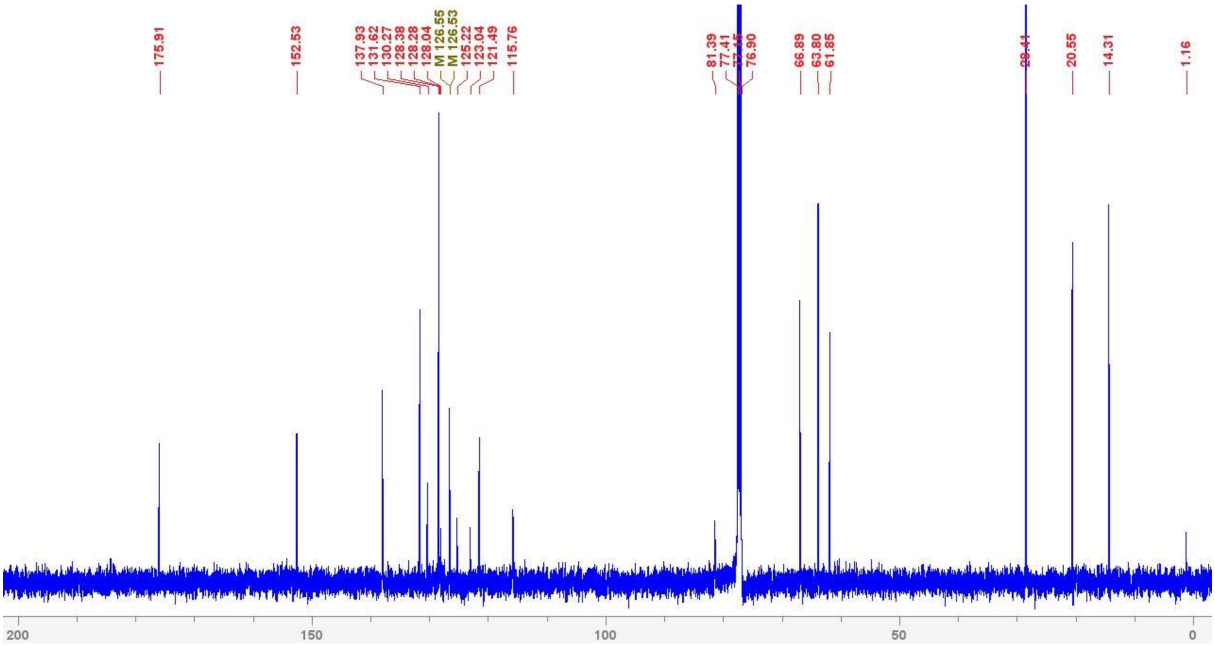

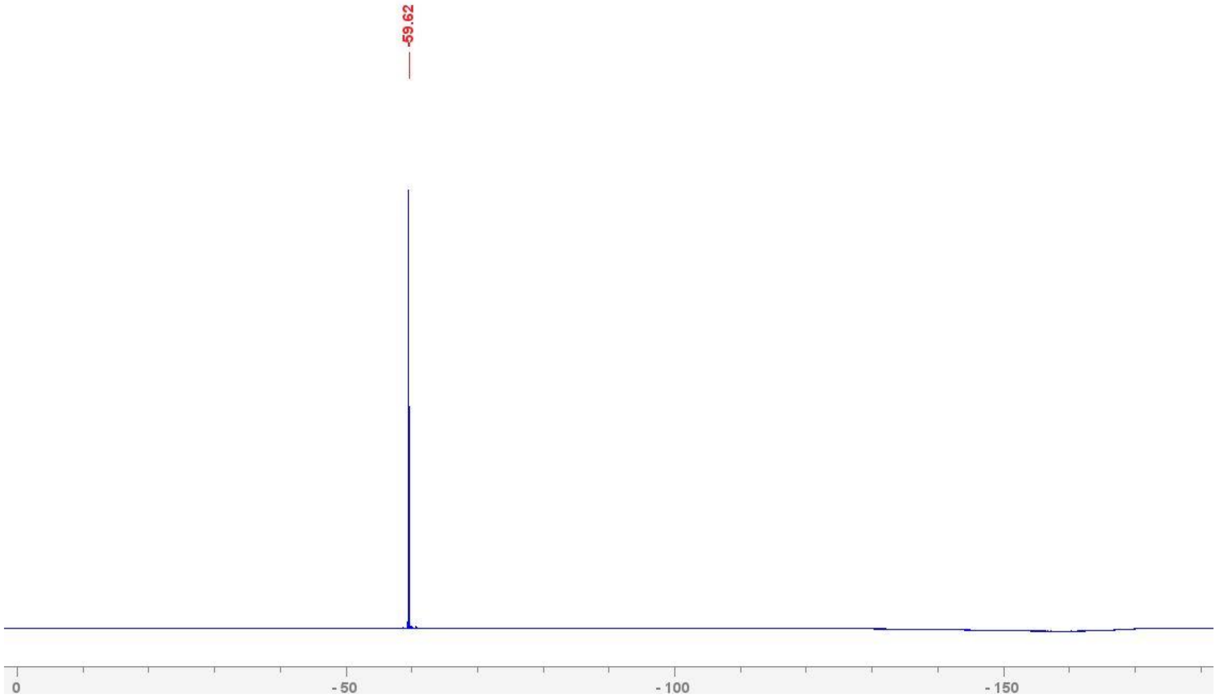

-59.62
0
- 50
- 100
- 150

tert-Butyl (4-(3-hydroxypropyl)-3-(trifluoromethyl)phenyl)carbamate (**27b**)

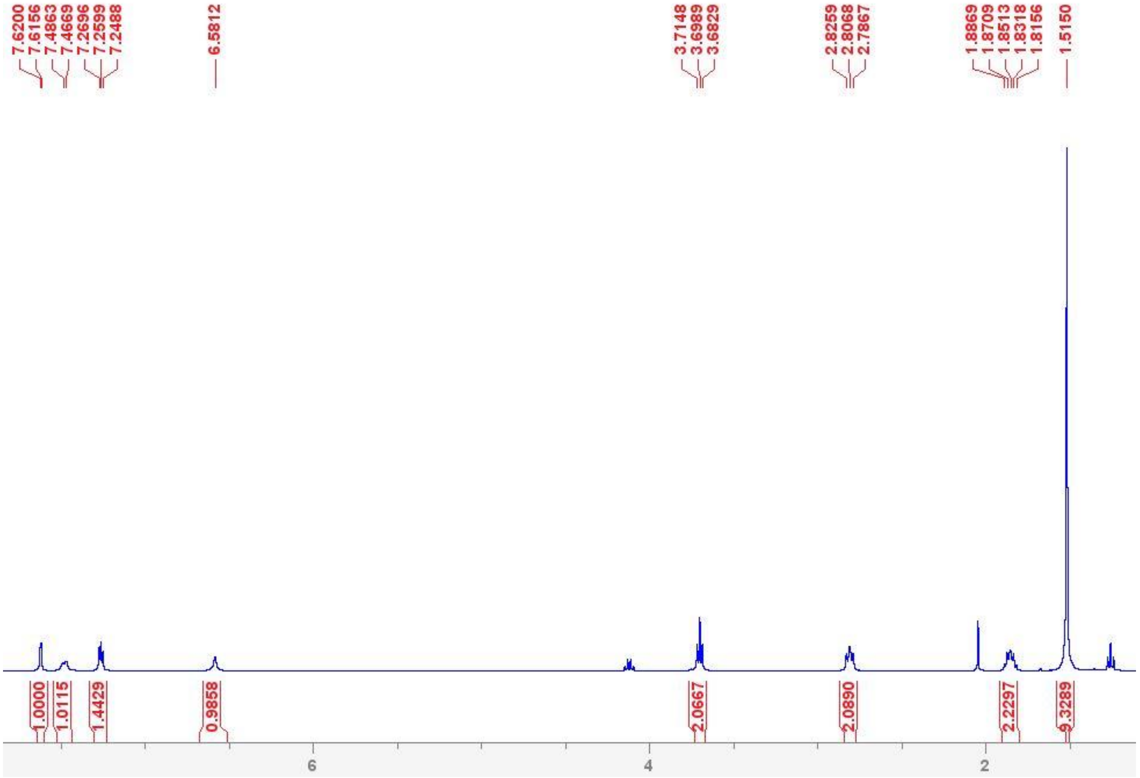

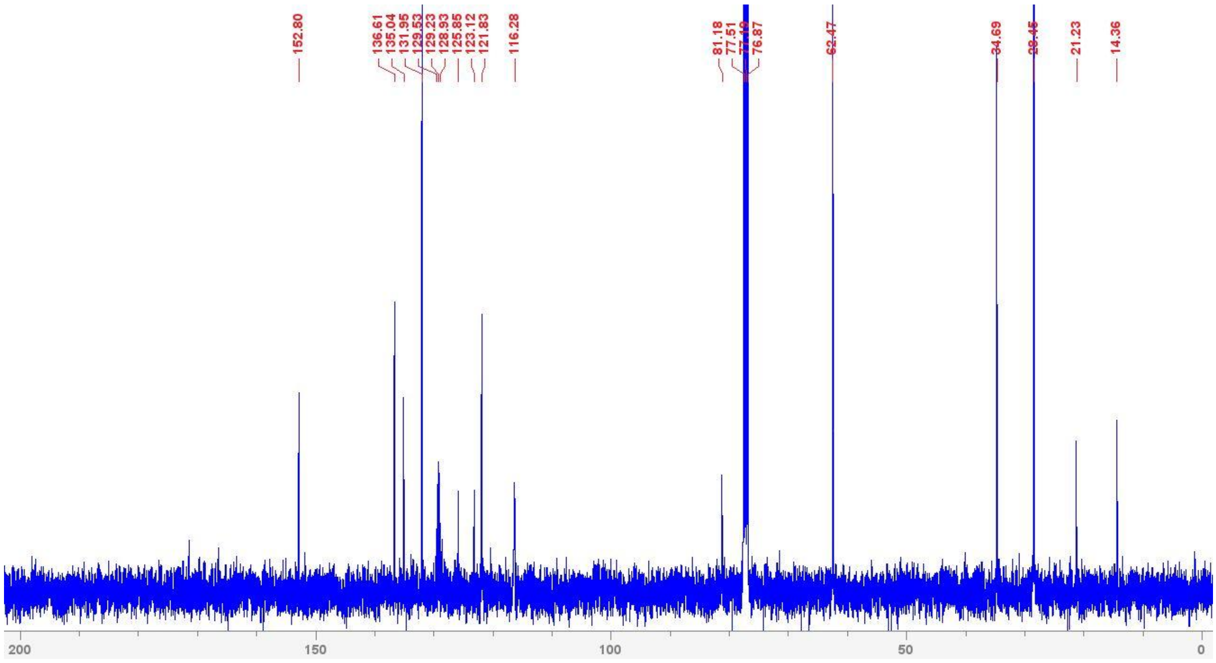

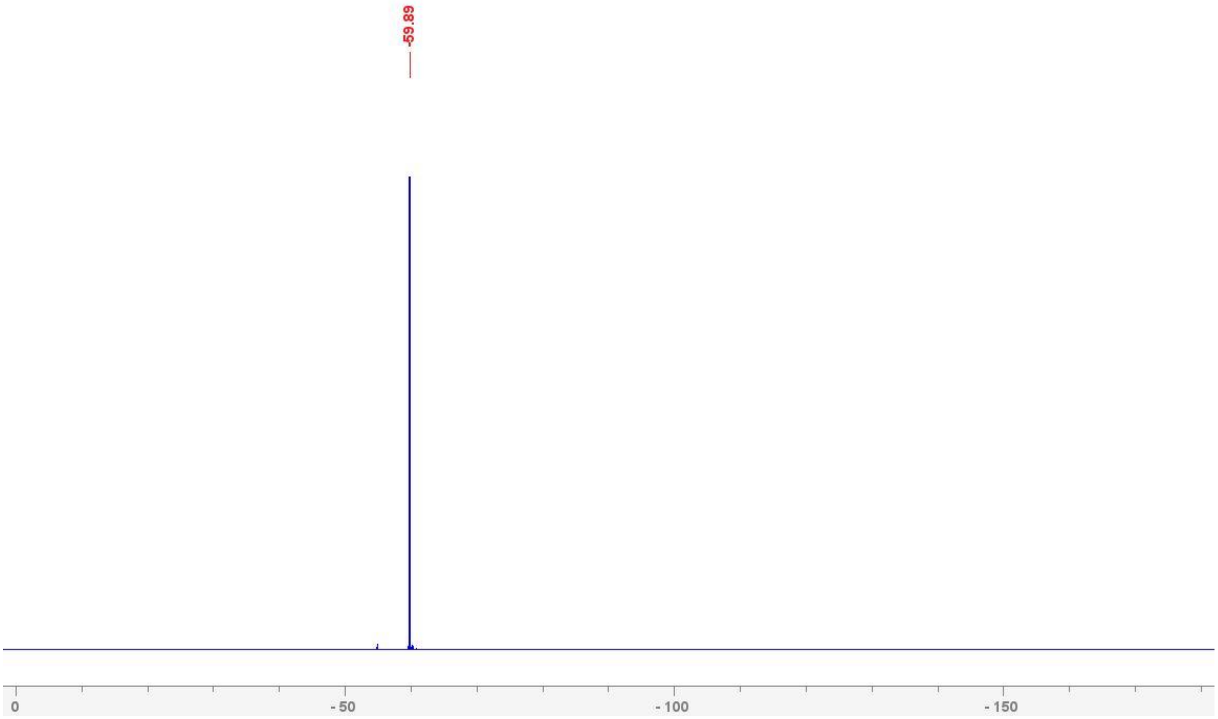

-59.89
0
- 50
- 100
- 150

tert-Butyl (*E*)-(4-(3-(1,3-dioxoisoindolin-2-yl)prop-1-en-1-yl)-3-(trifluoromethyl)phenyl)carbamate (**28a**)

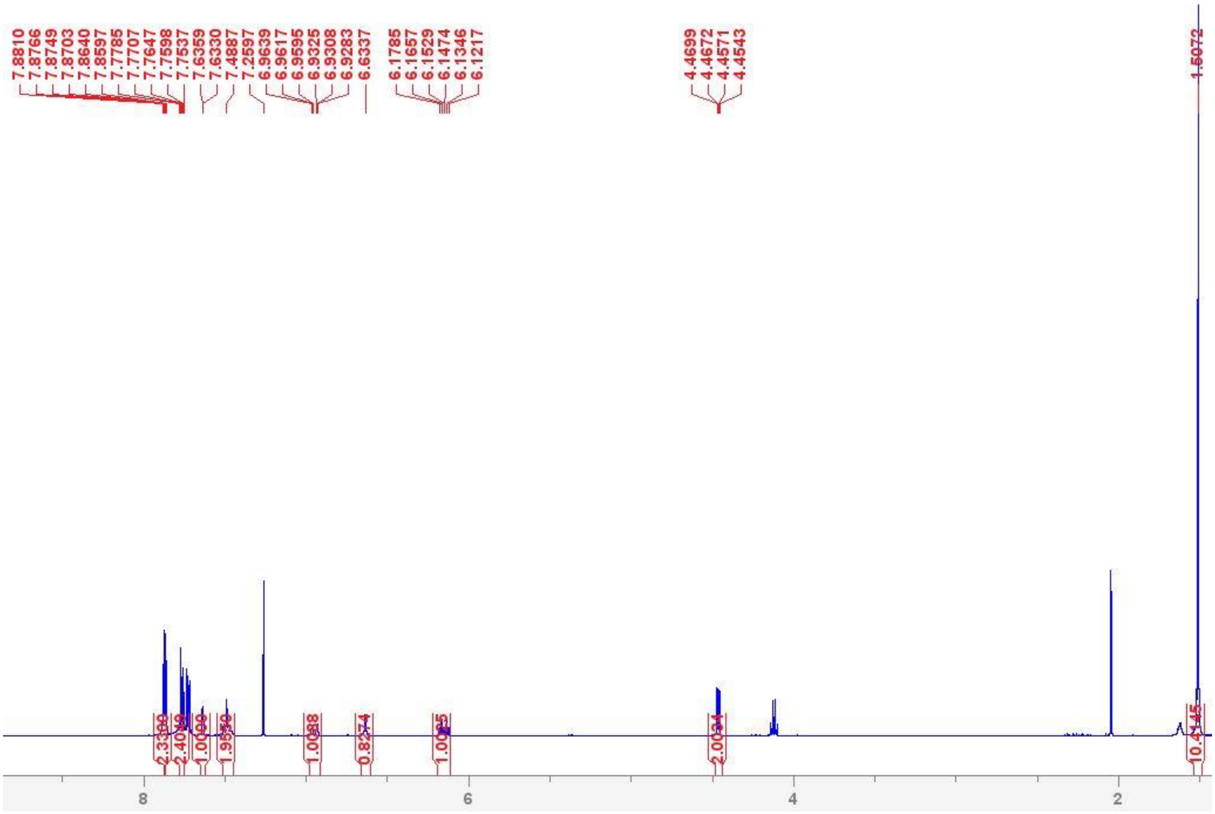

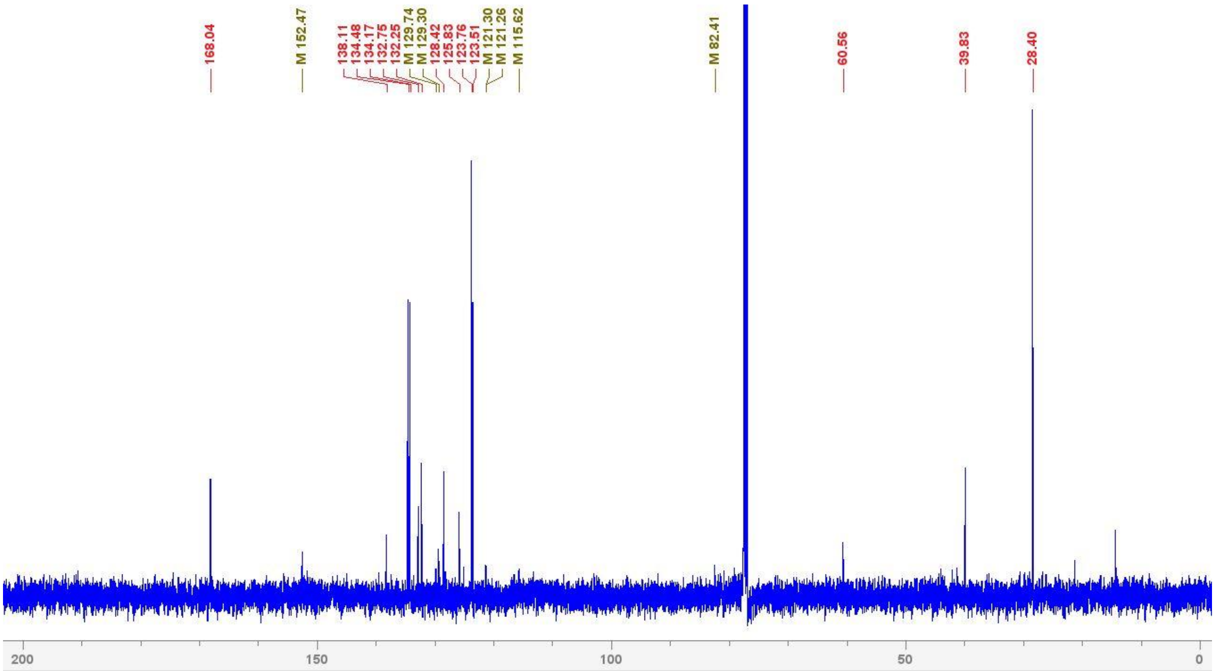

tert-Butyl (4-(3-(1,3-dioxoisoindolin-2-yl)propyl)-3-(trifluoromethyl)phenyl)carbamate (**28b**)

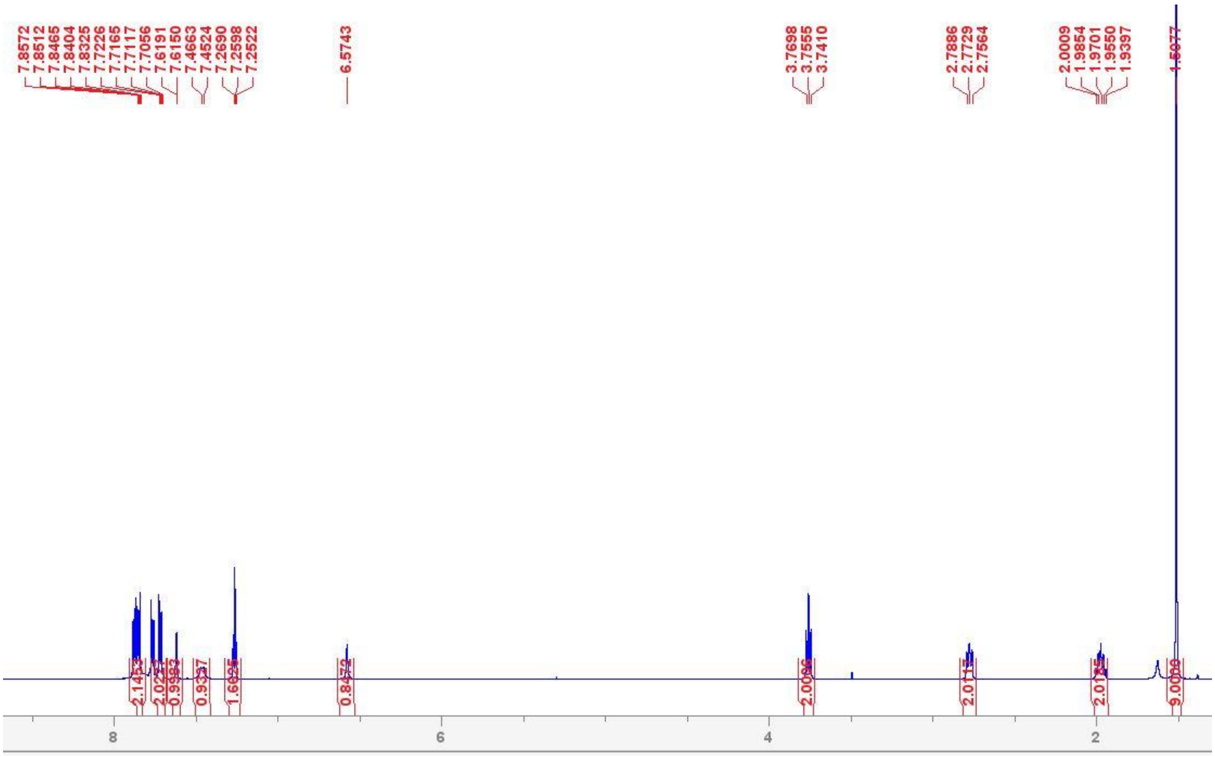

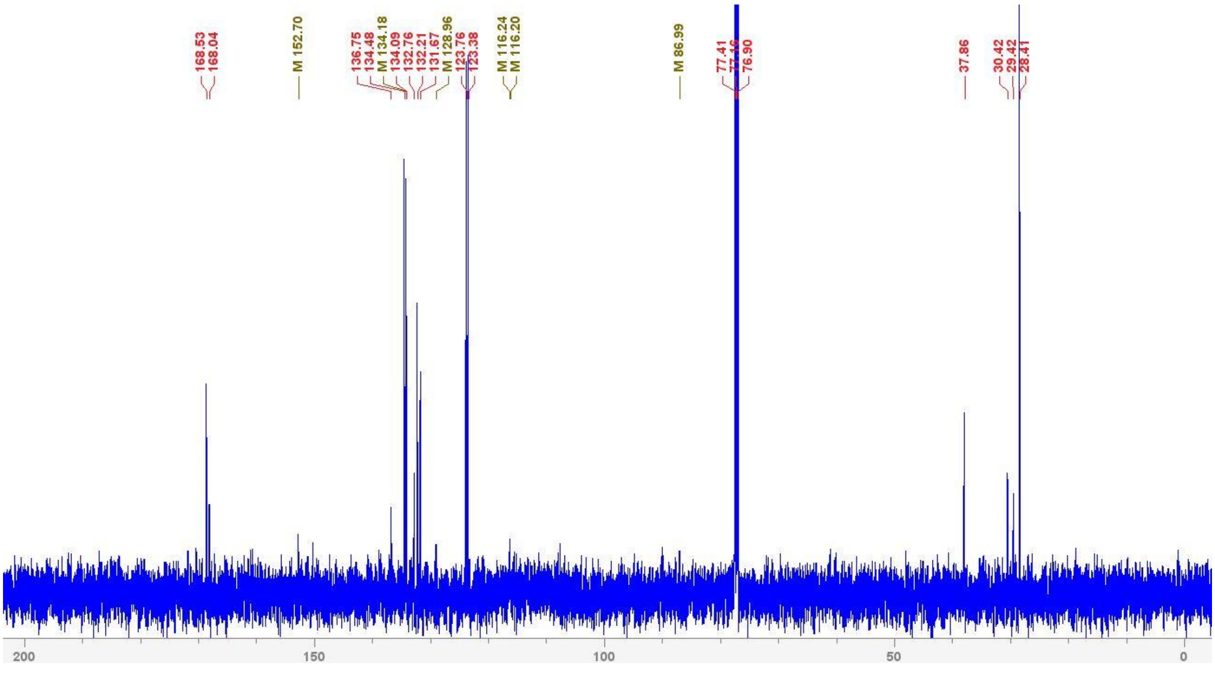

Methyl 3-iodo-2-methylbenzoate (**31a**)

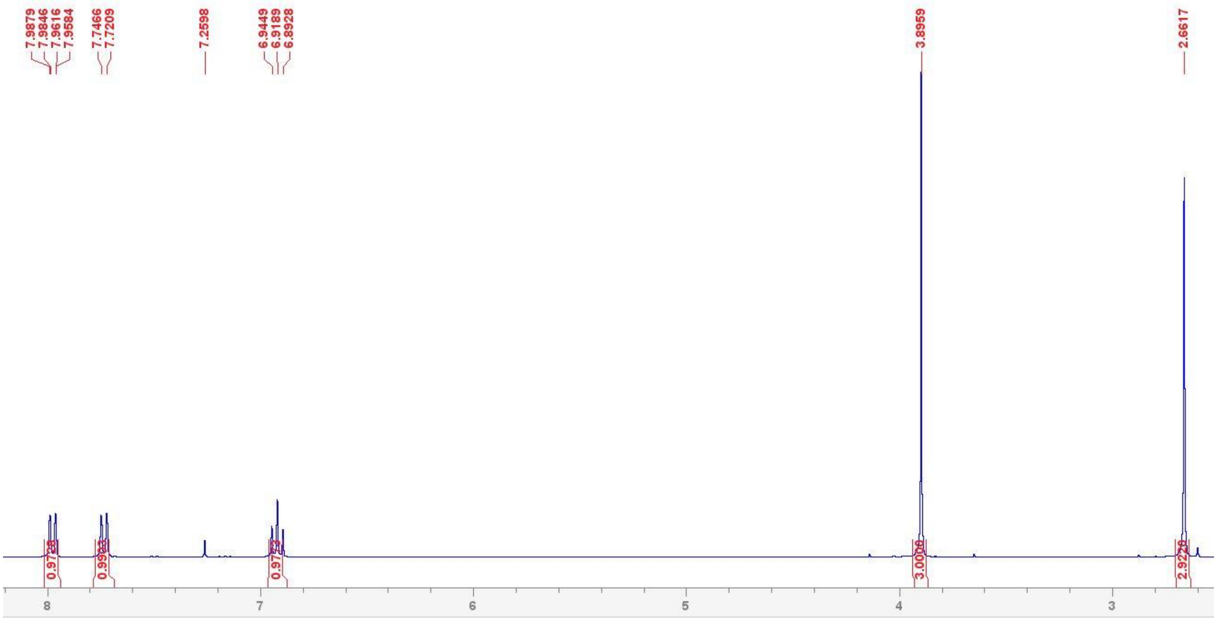

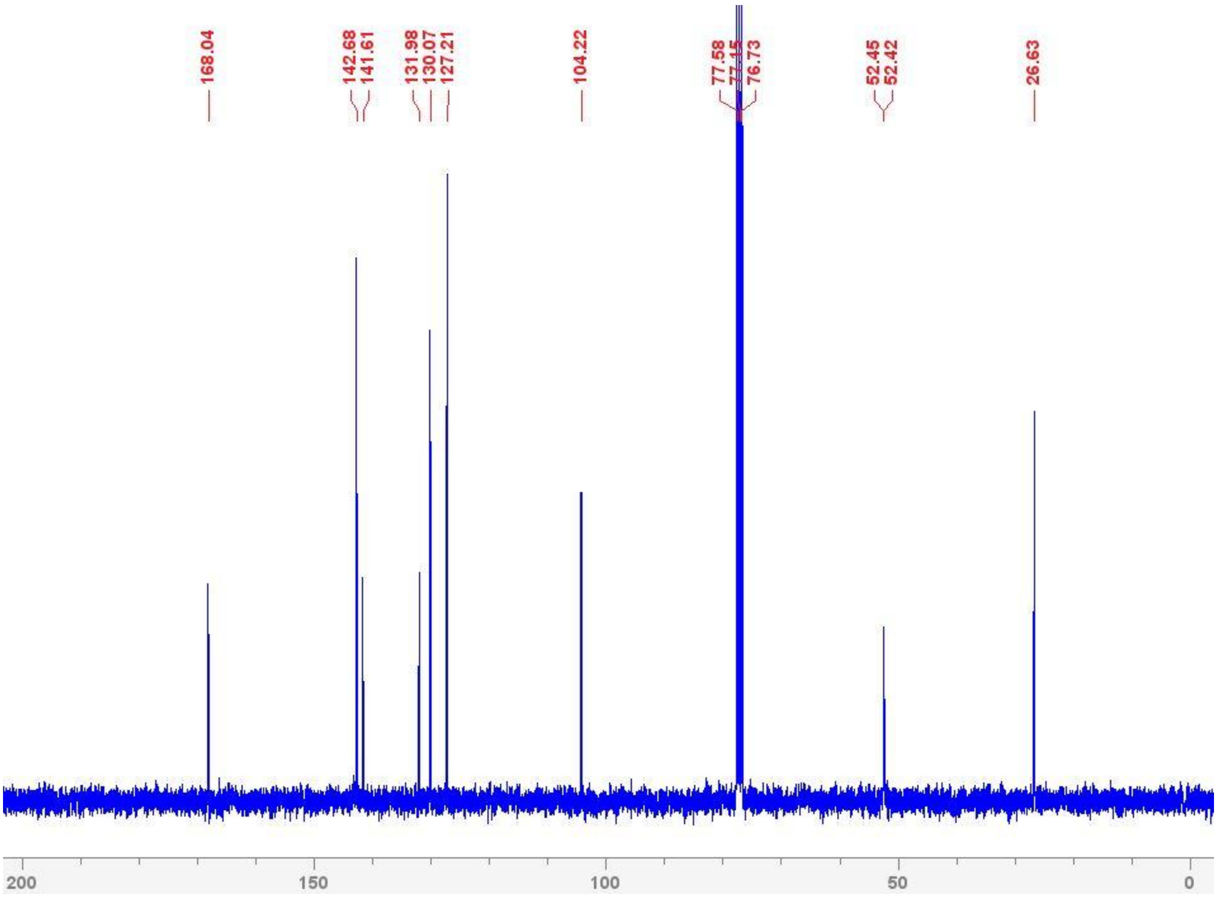

Methyl 3-iodo-4-methylbenzoate (**31b**)

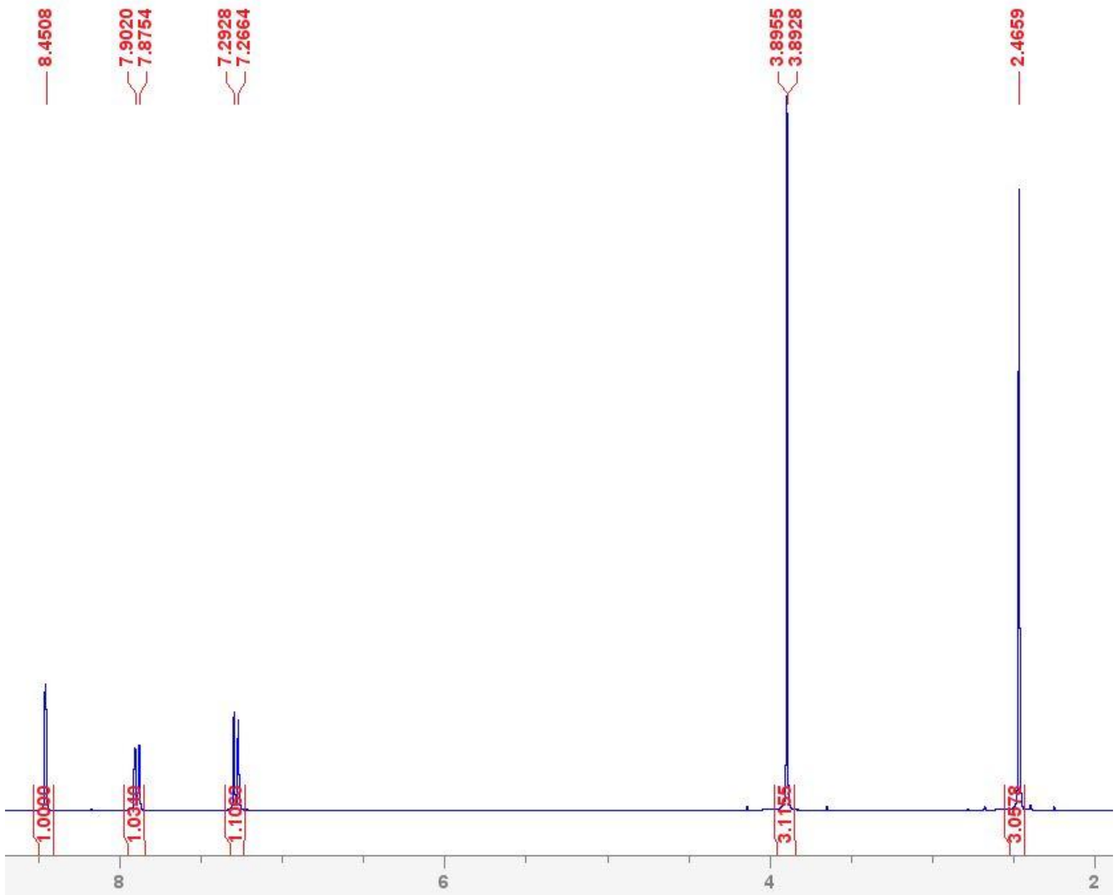

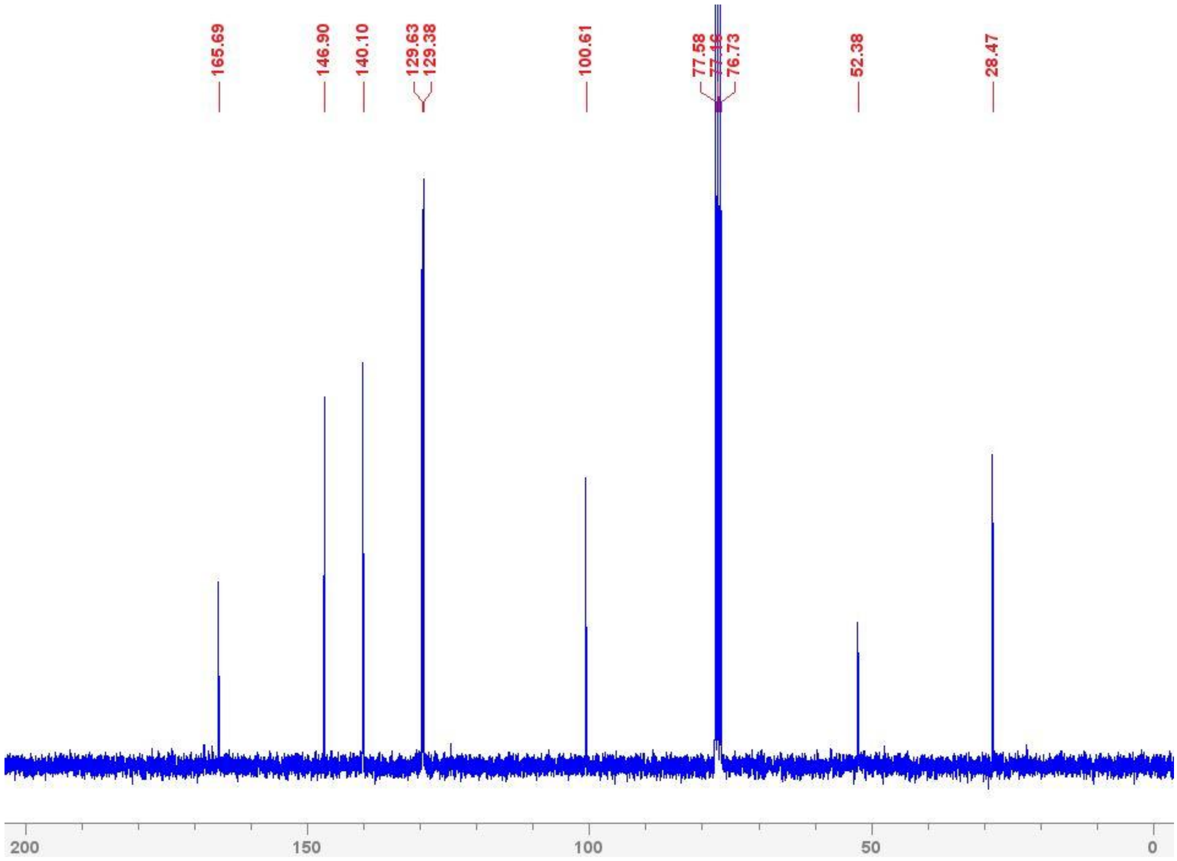

Methyl 5-iodo-2-methylbenzoate (**31c**)

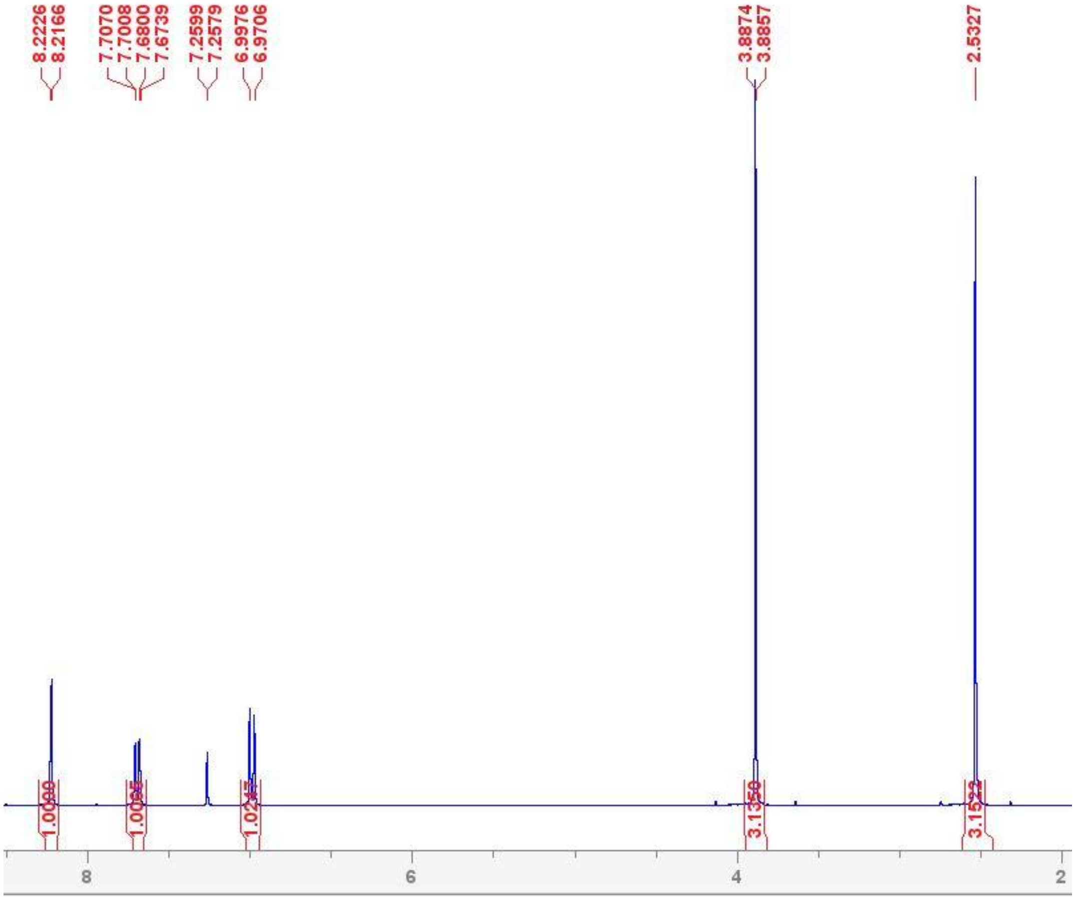

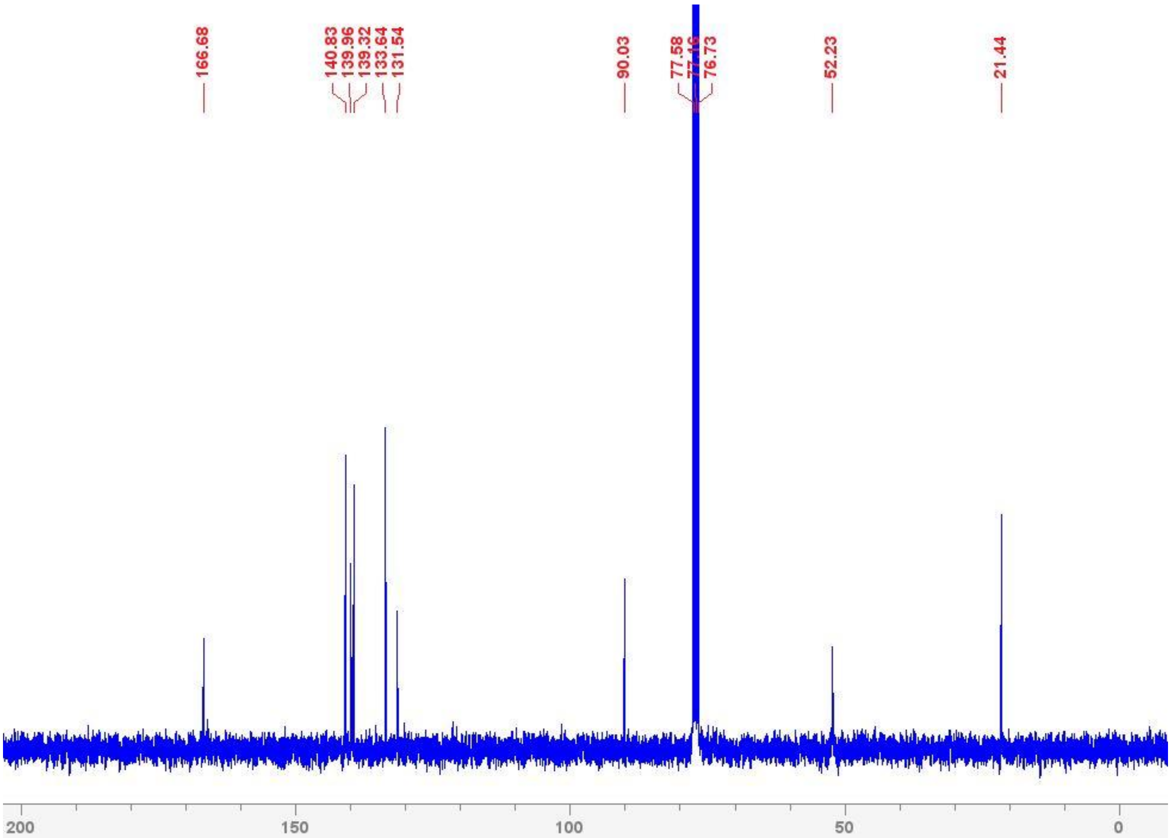

Methyl 3-iodo-2,4-dimethylbenzoate (**31d**)

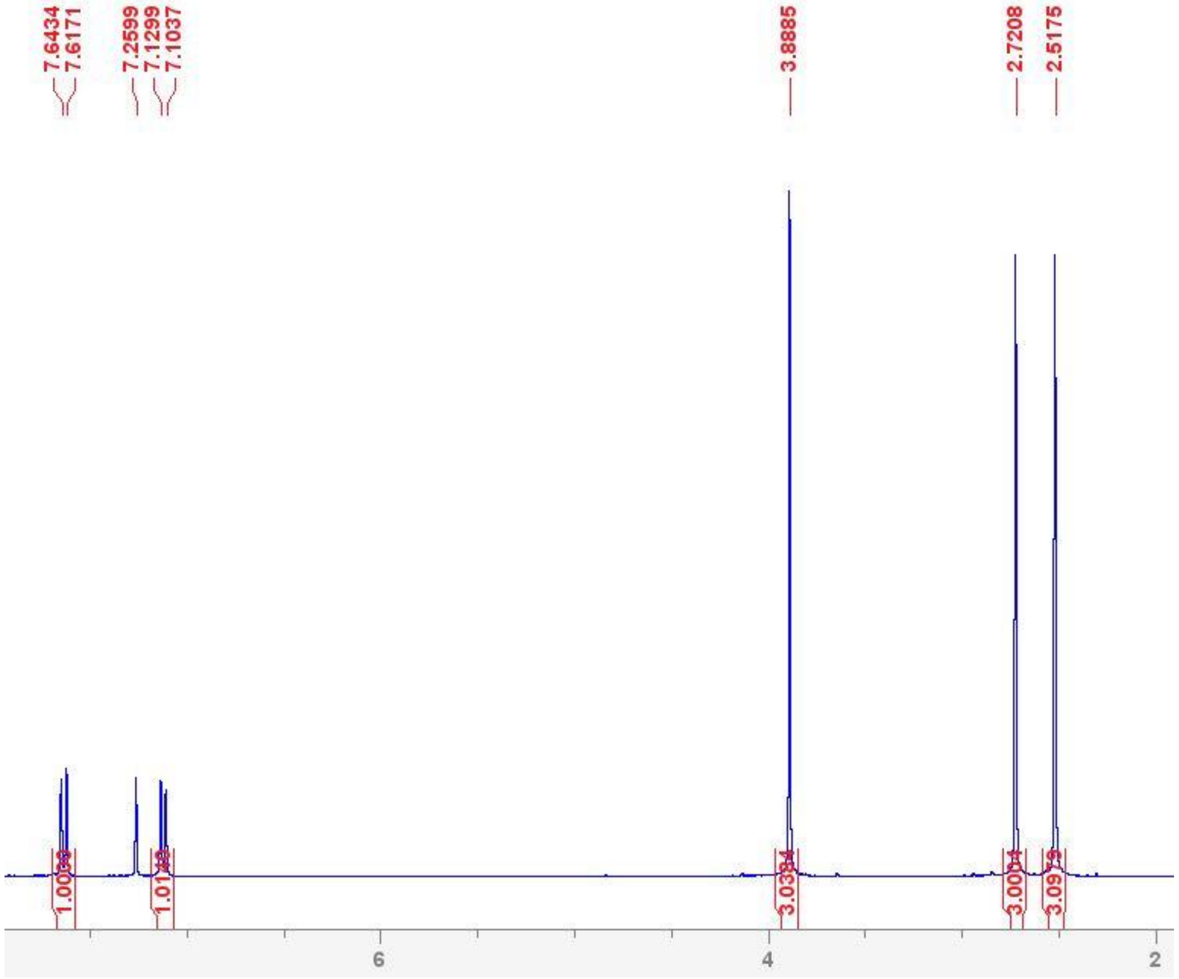

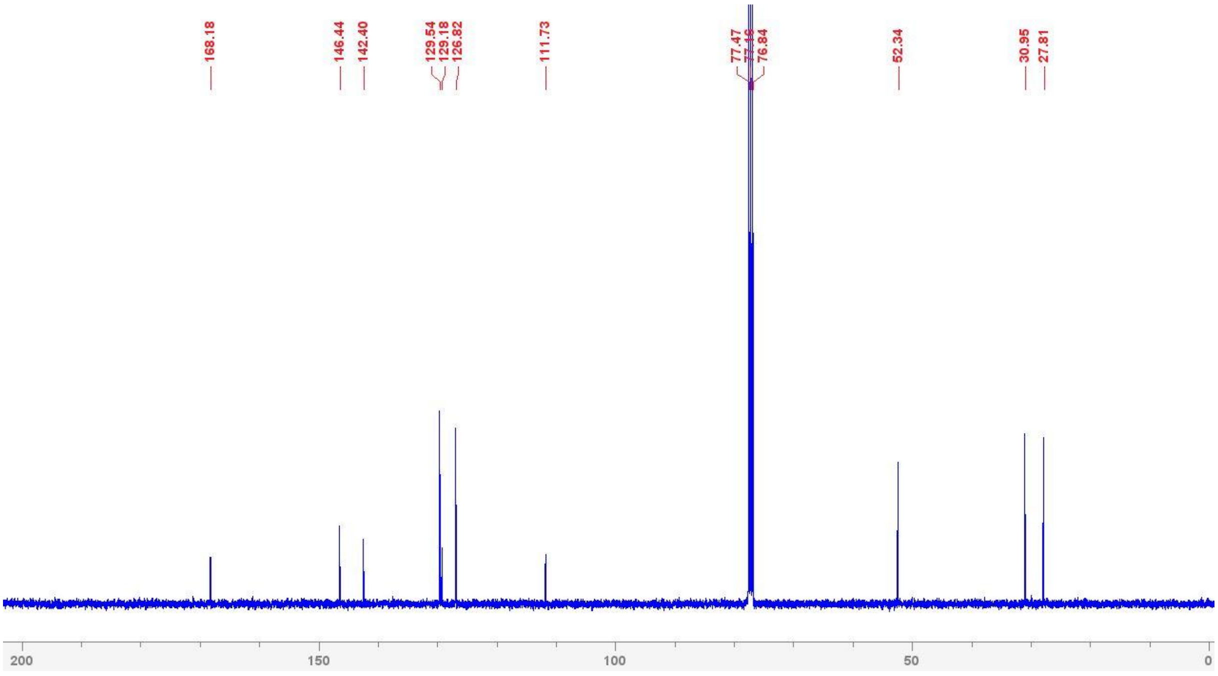

Methyl 3-(imidazo[1,2-*b*]pyridazin-3-ylethynyl)-2-methylbenzoate (**32a**)

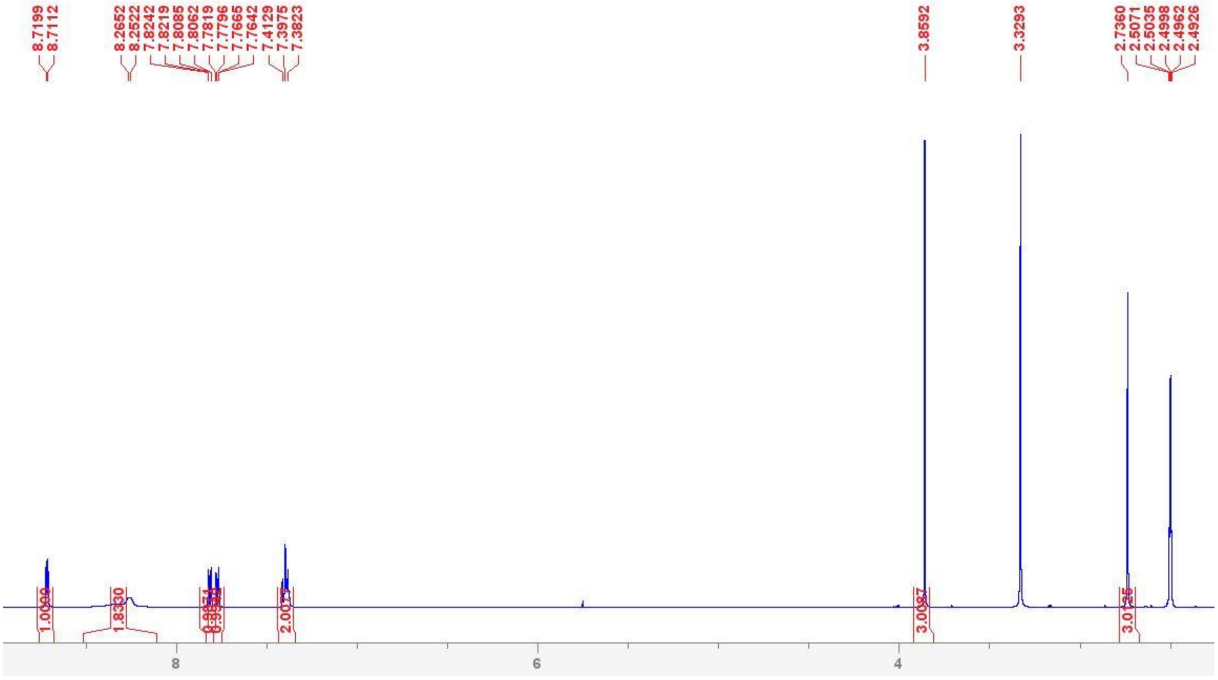

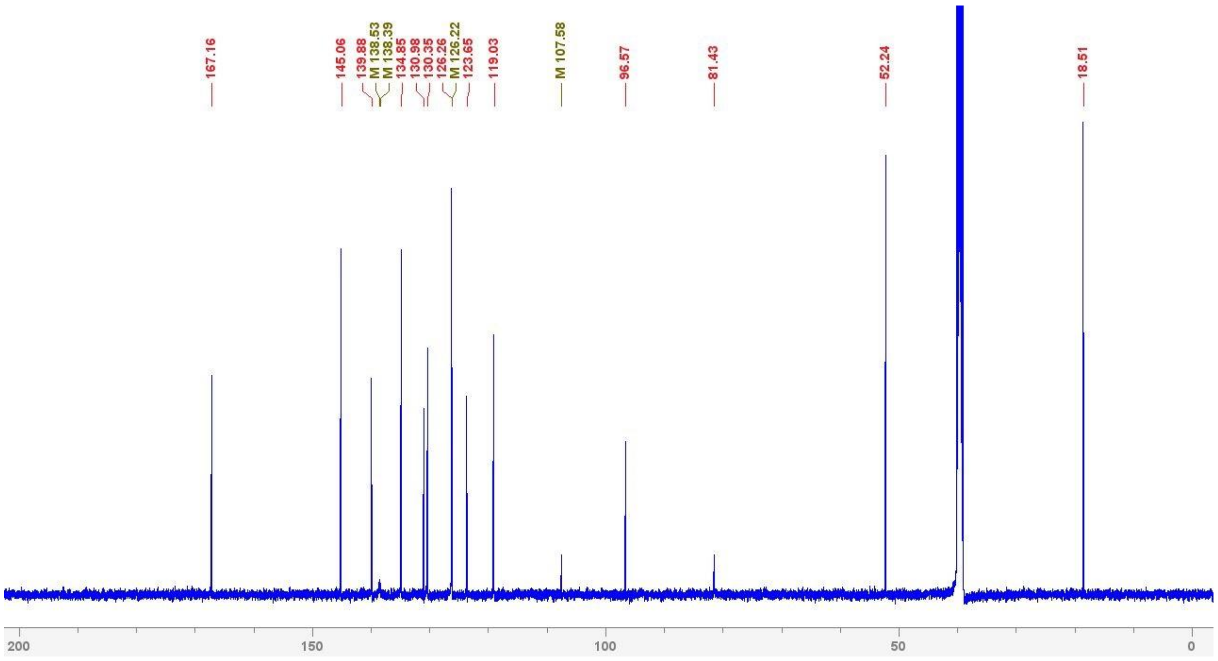

Methyl 3-(imidazo[1,2-*b*]pyridazin-3-ylethynyl)-4-methylbenzoate (**32b**)

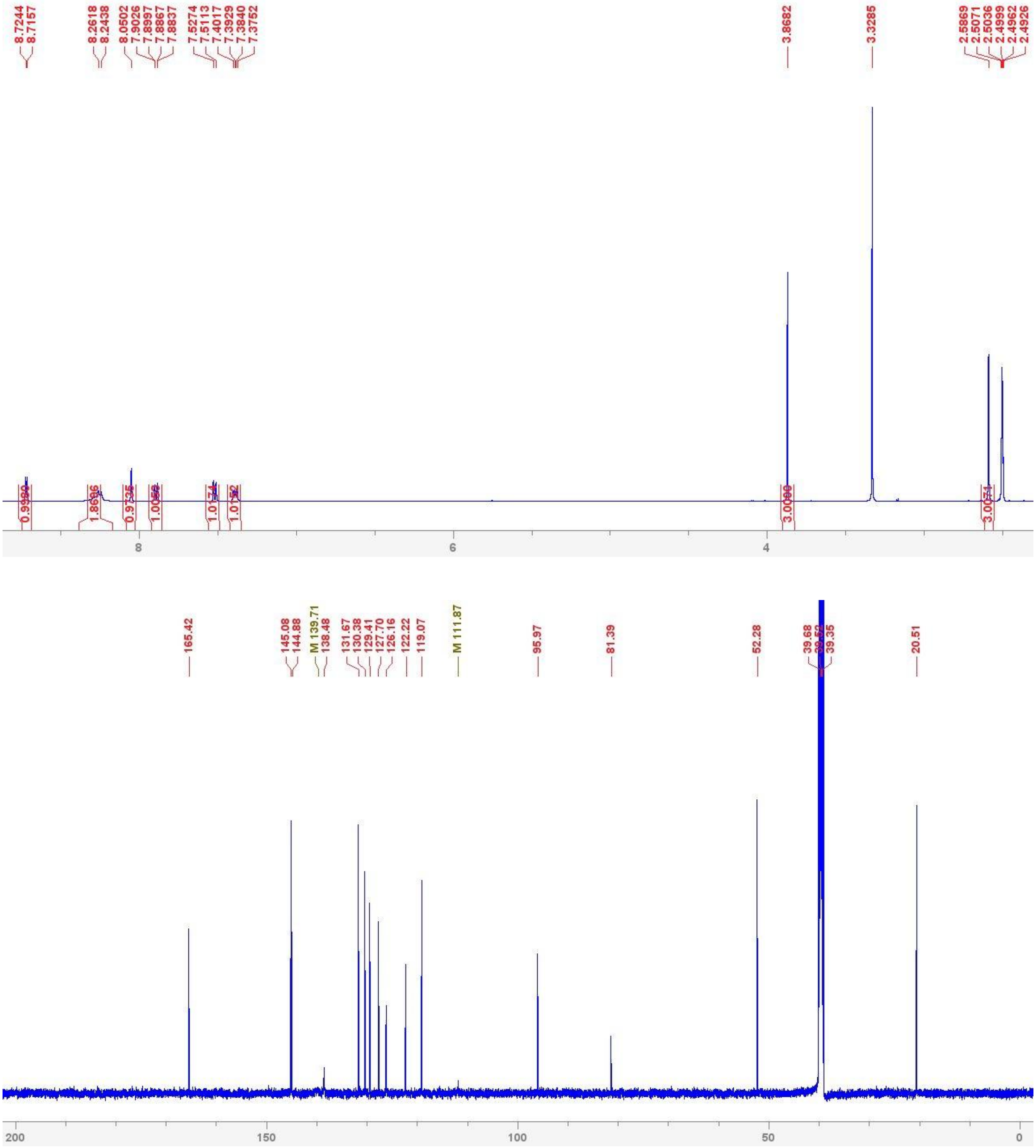

Methyl 5-(imidazo[1,2-*b*]pyridazin-3-ylethynyl)-2-methylbenzoate (**32c**)

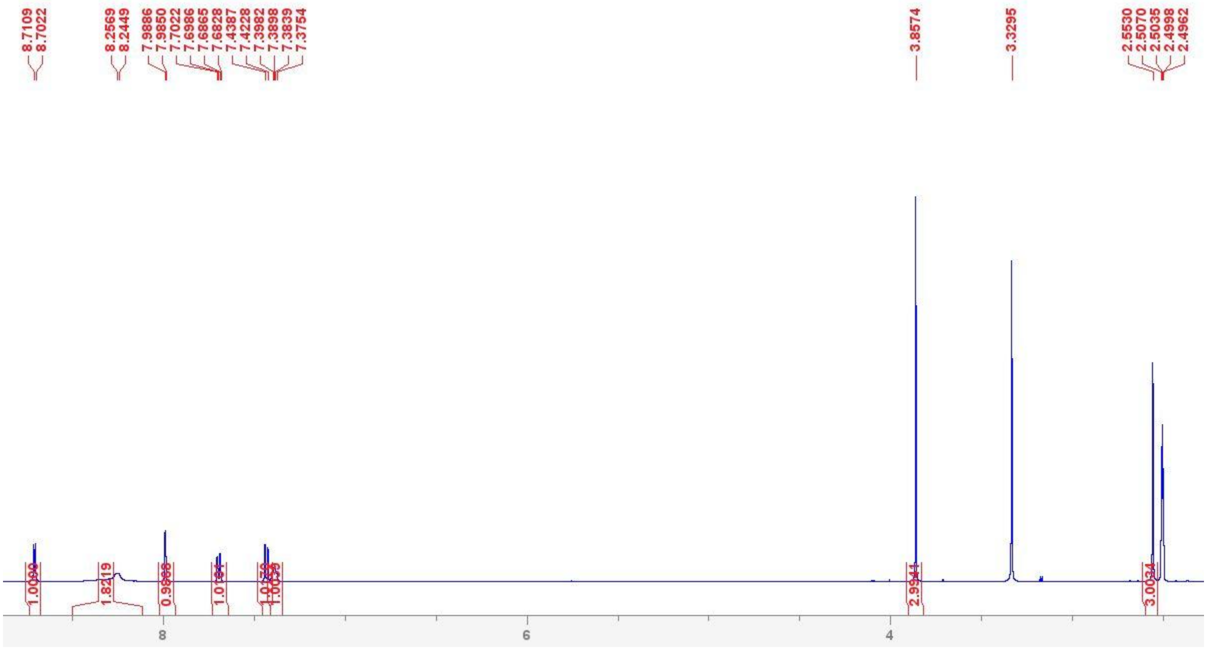

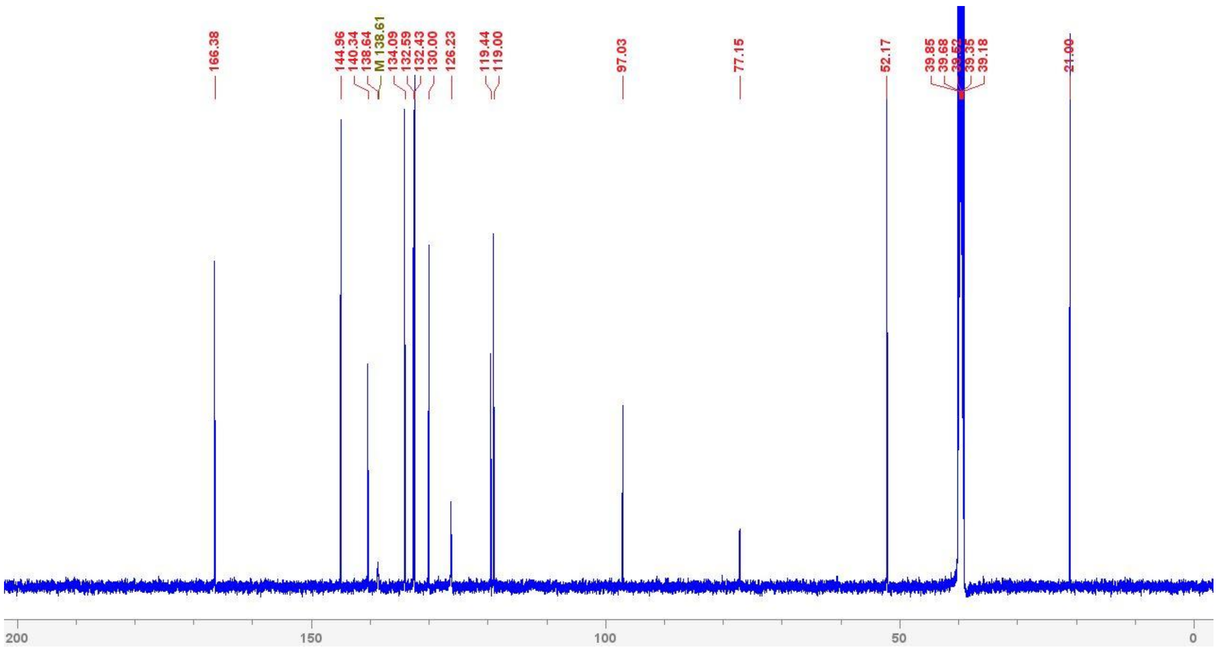

Methyl 3-(imidazo[1,2-*b*]pyridazin-3-ylethynyl)-2,4-dimethylbenzoate (**32d**)

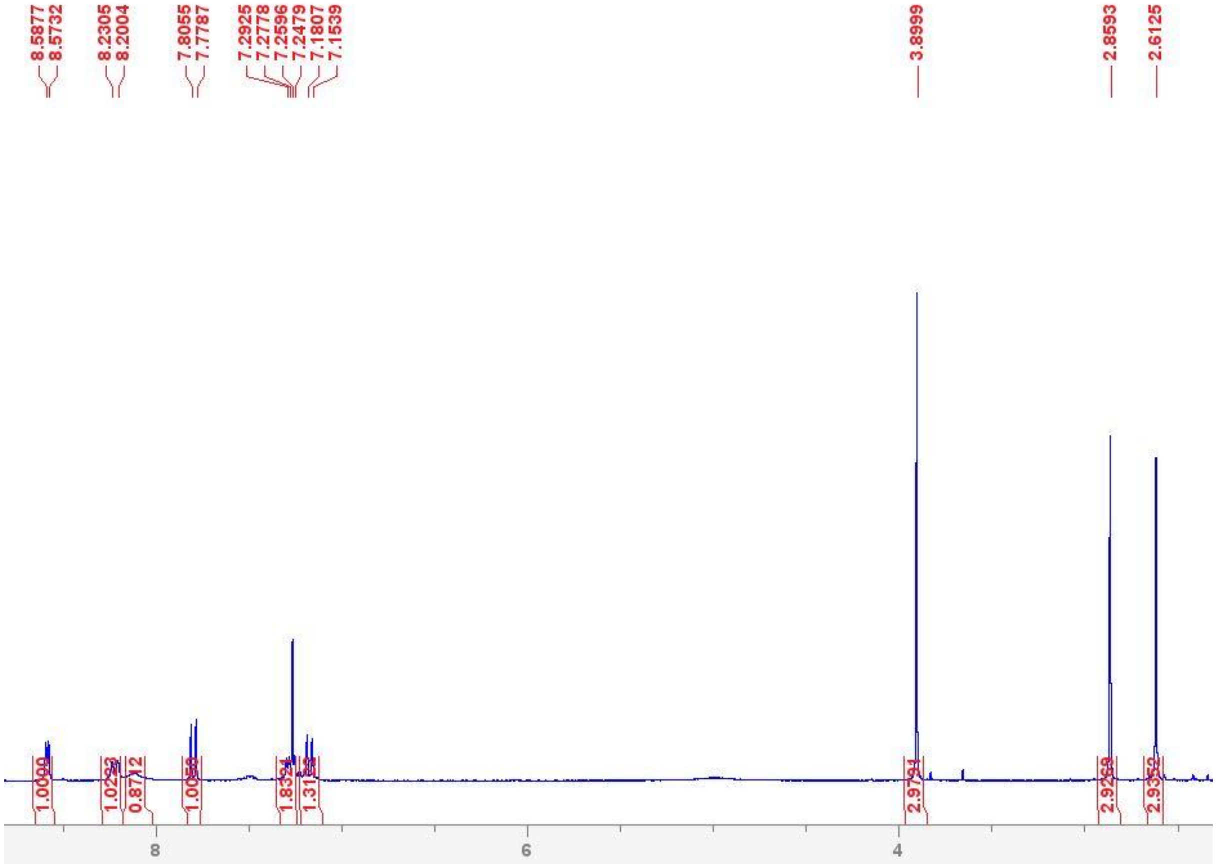

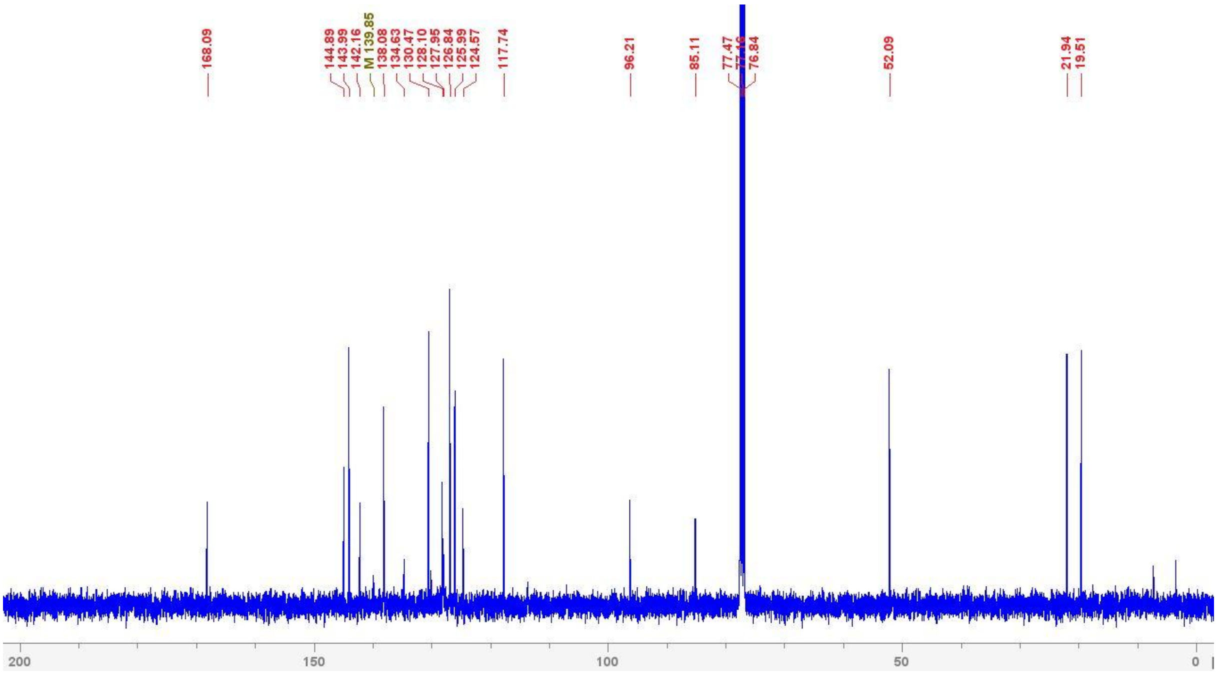

3-(Imidazo[1,2-*b*]pyridazin-3-ylethynyl)-2-methyl-*N*-(3-(trifluoromethyl)phenyl)benzamide (**1S**)

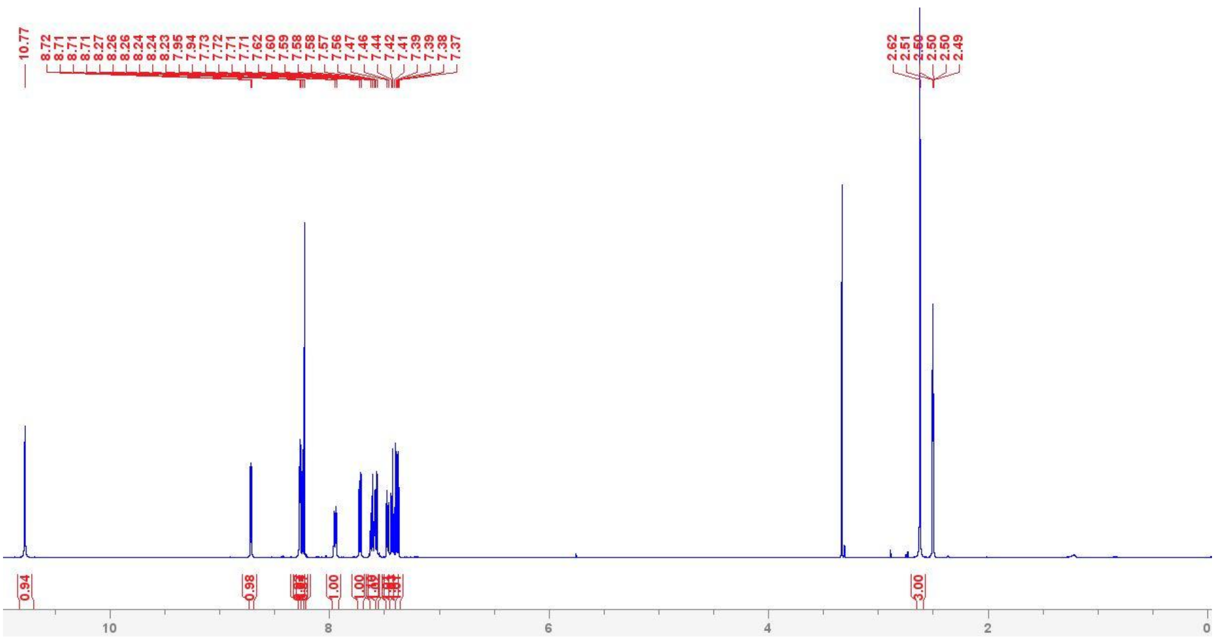

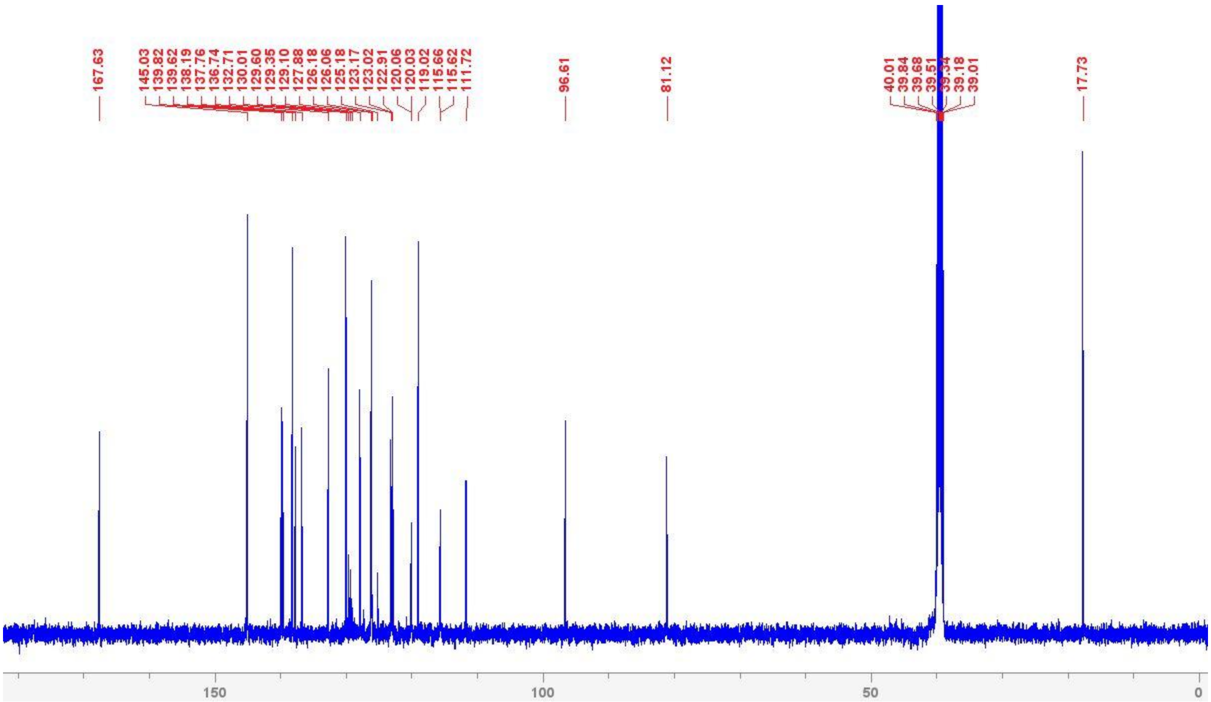

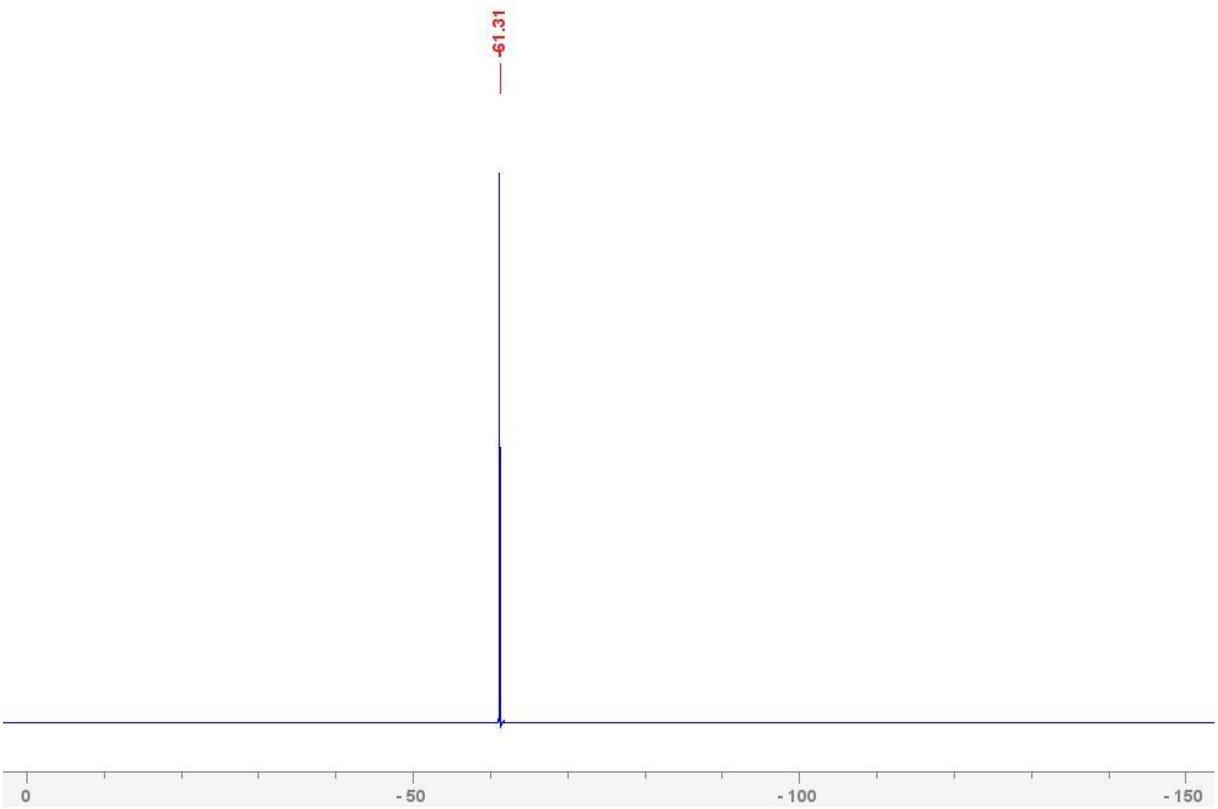
-61.31
0
- 50
- 100
- 150

3-(Imidazo[1,2-*b*]pyridazin-3-ylethynyl)-4-methyl-*N*-(3-(trifluoromethyl)phenyl)benzamide (**2S**)

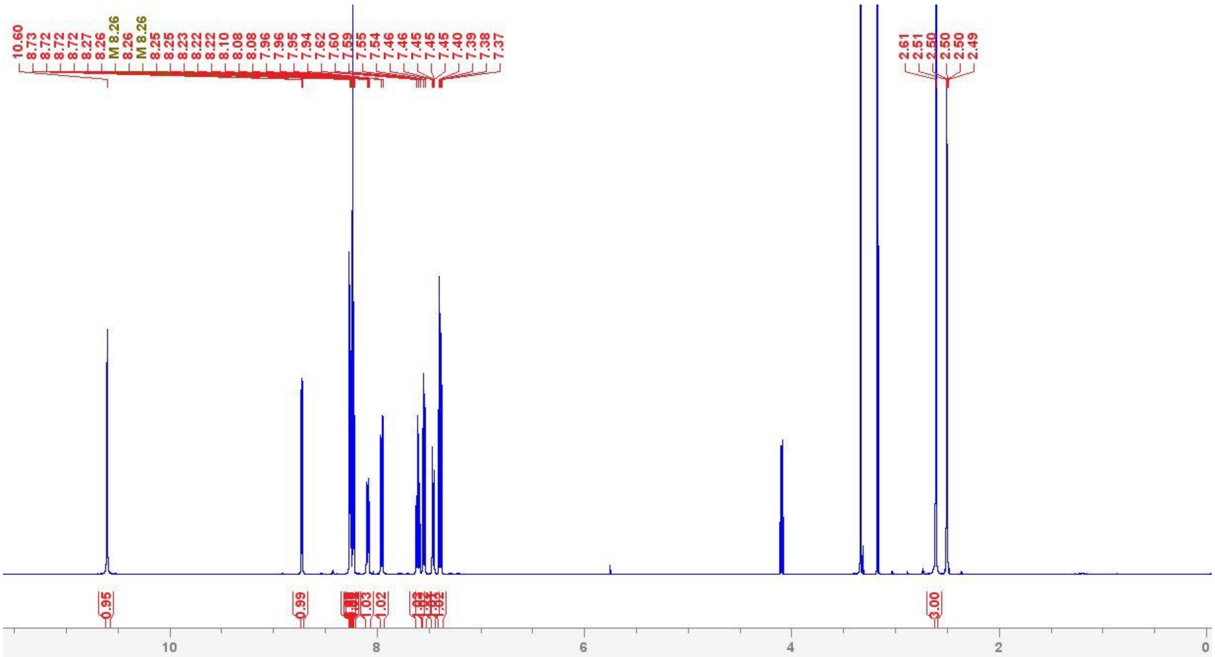

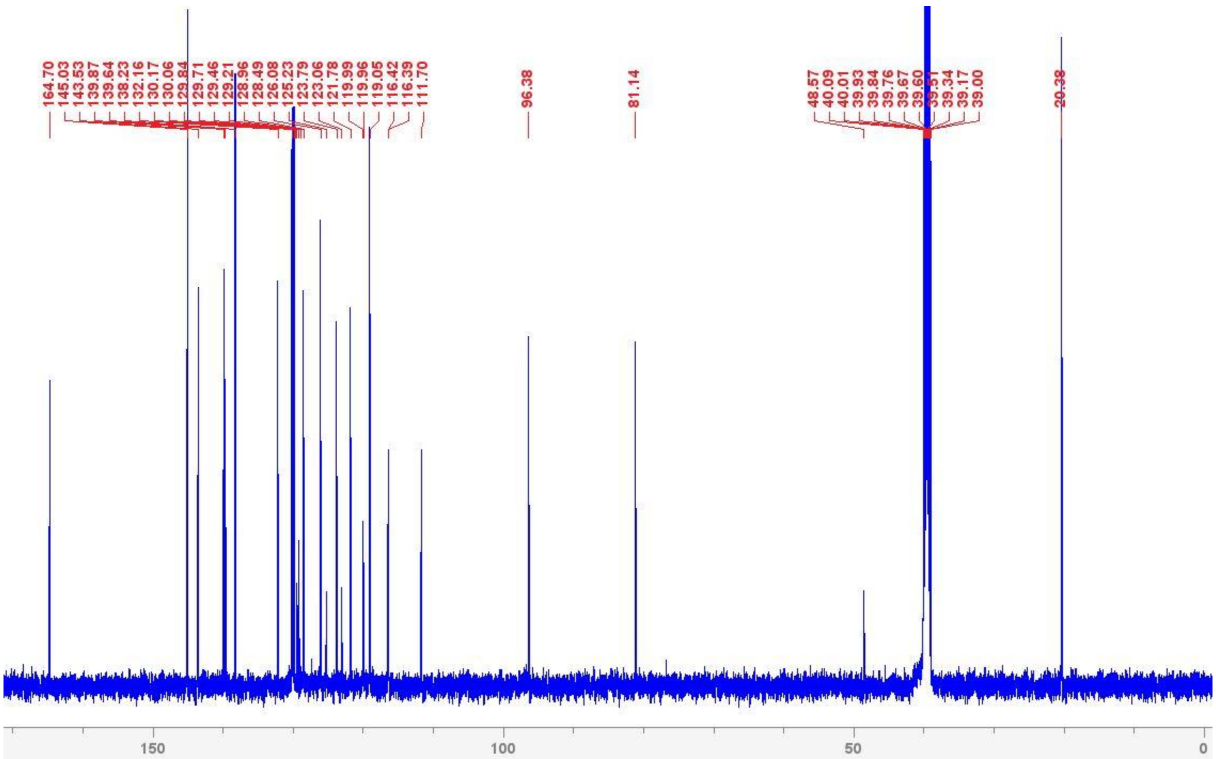

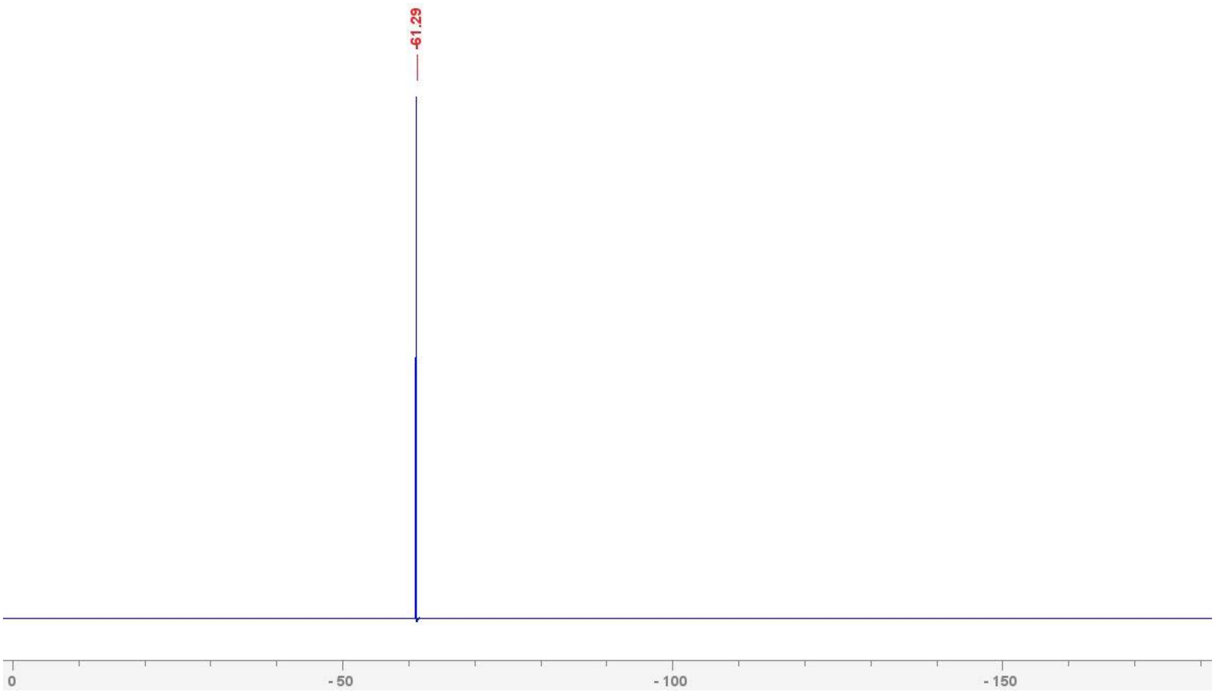
-61.29
0
-50
-100
-150

5-(Imidazo[1,2-*b*]pyridazin-3-ylethynyl)-2-methyl-*N*-(3-(trifluoromethyl)phenyl)benzamide (**3S**)

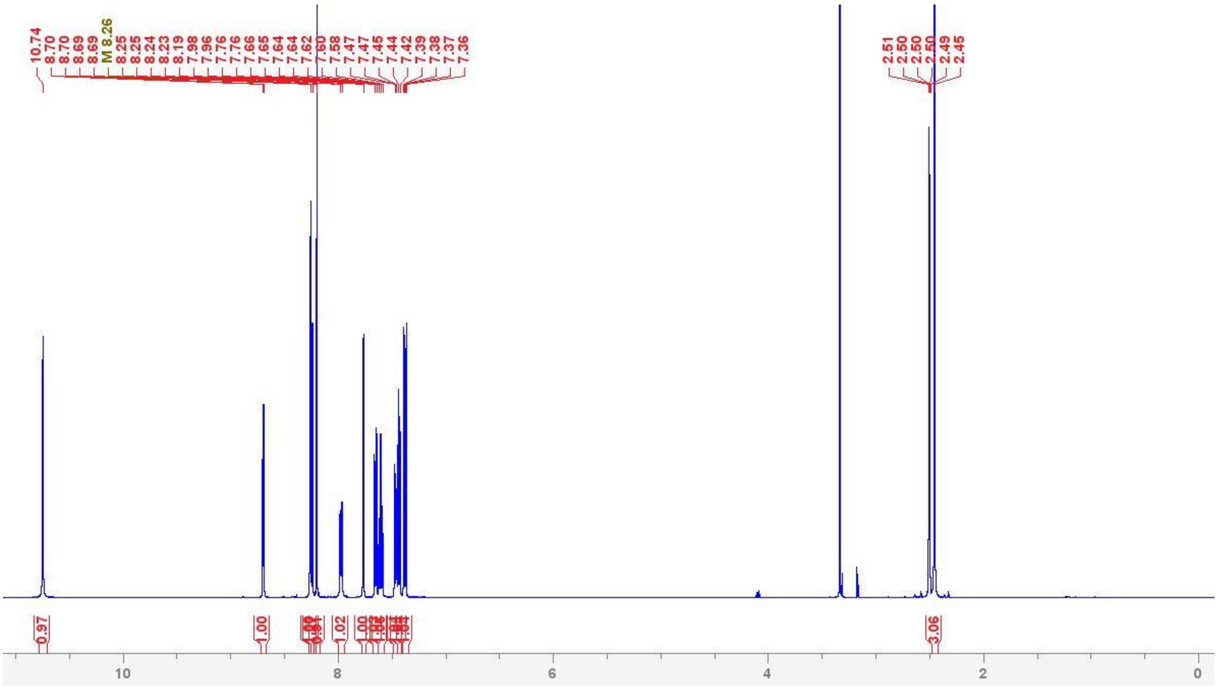

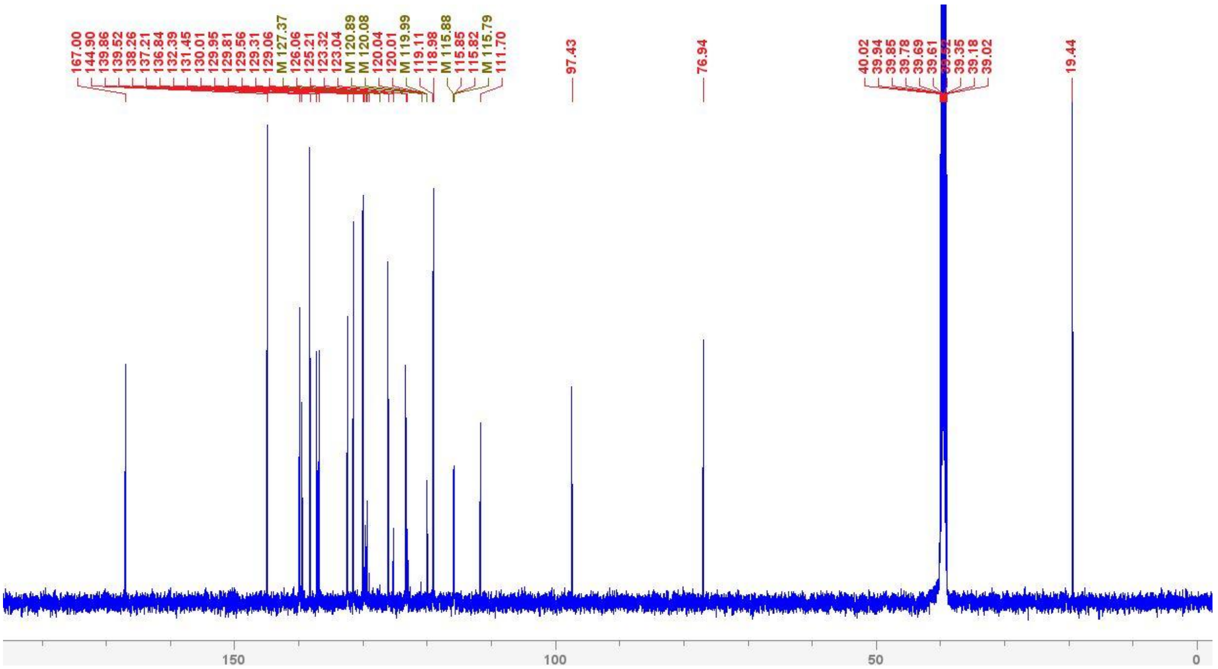

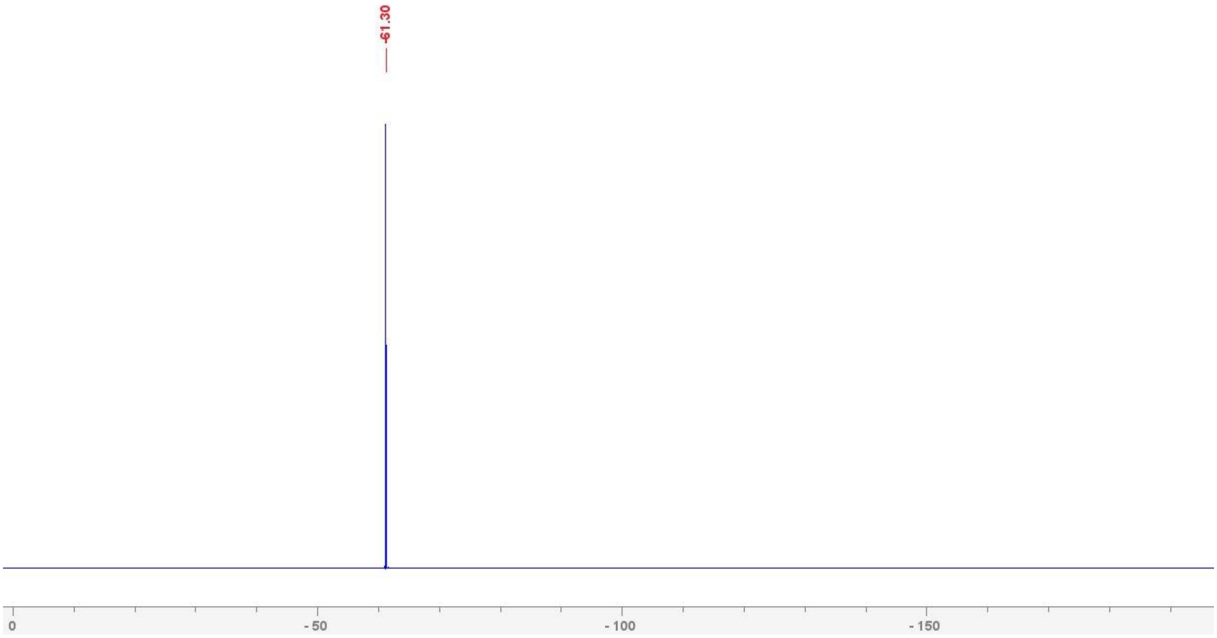
-61.30
0
- 50
- 100
- 150

3-(Imidazo[1,2-*b*]pyridazin-3-ylethynyl)-2,4-dimethyl-*N*-(3-(trifluoromethyl)phenyl)benzamide (**4S**)

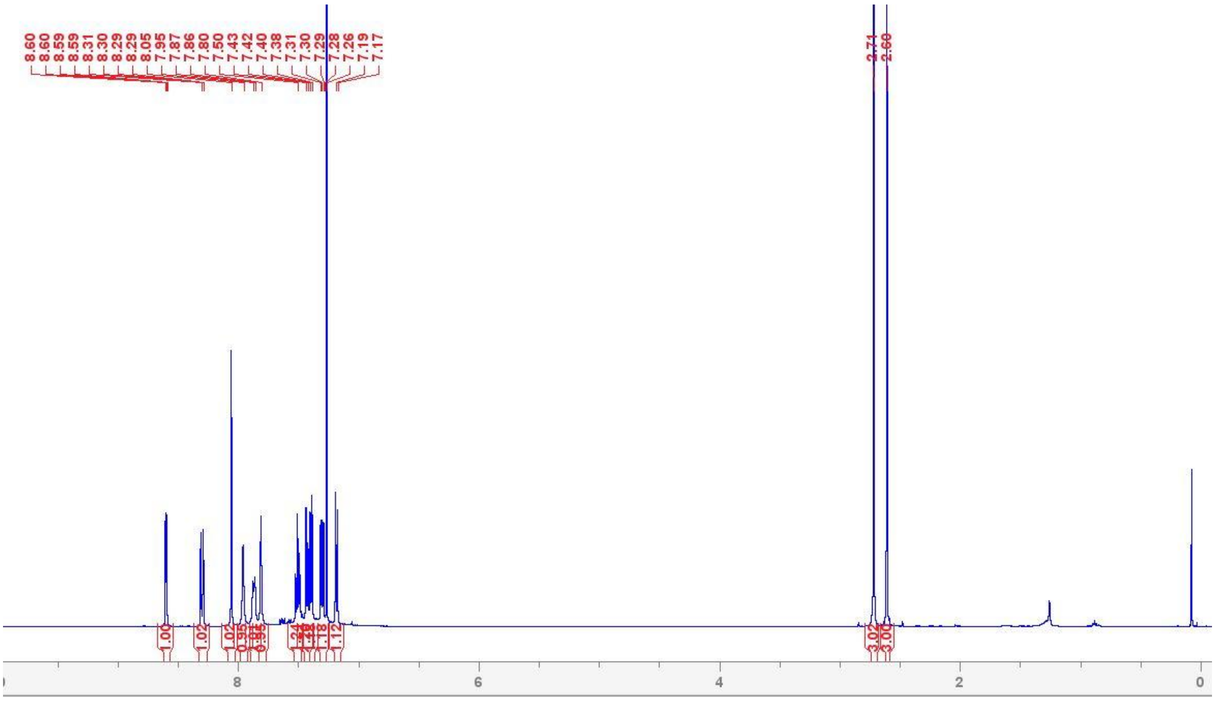

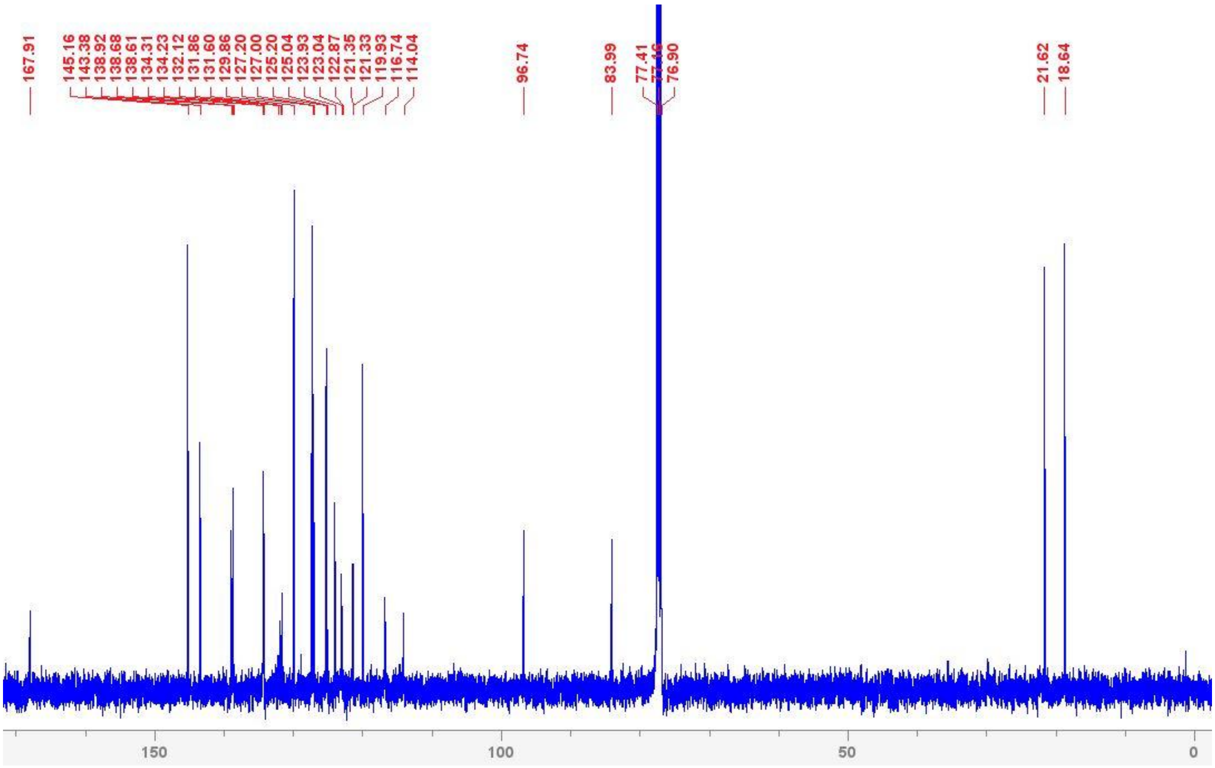

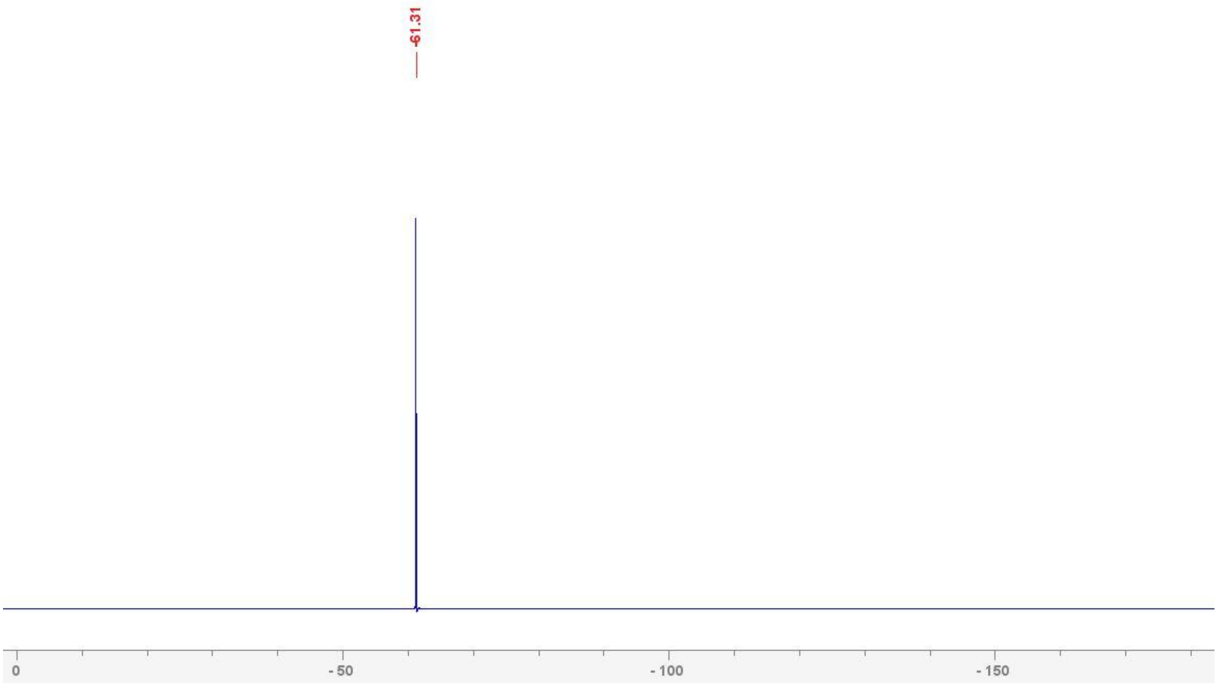
-61.31
0
- 50
- 100
- 150

3-Iodo-2-methyl-*N*-(3-(trifluoromethyl)phenyl)benzamide (**30S a**)

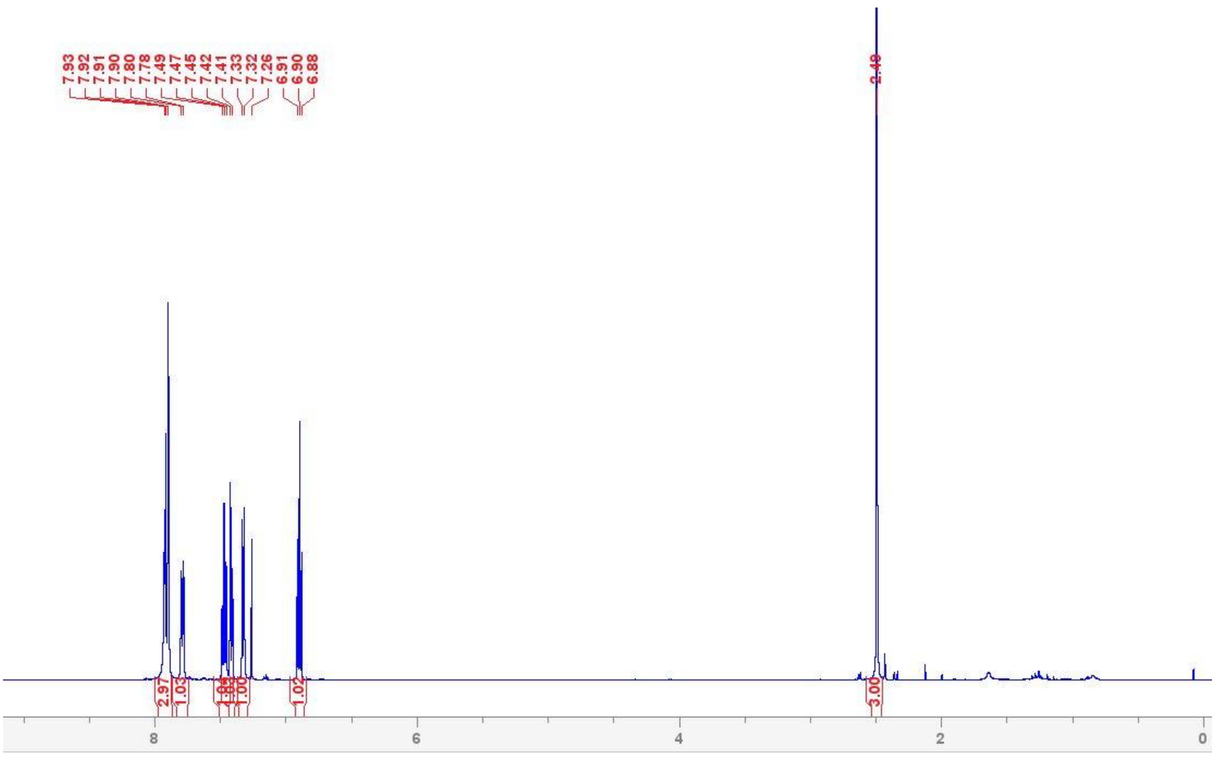

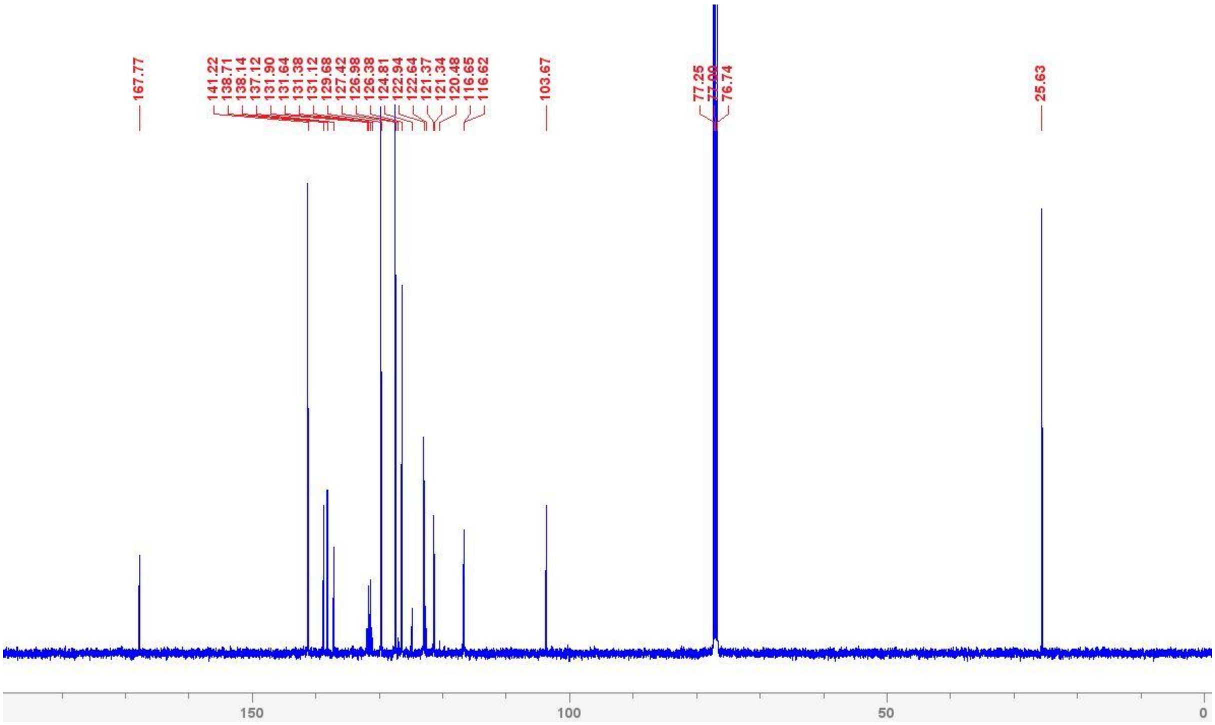

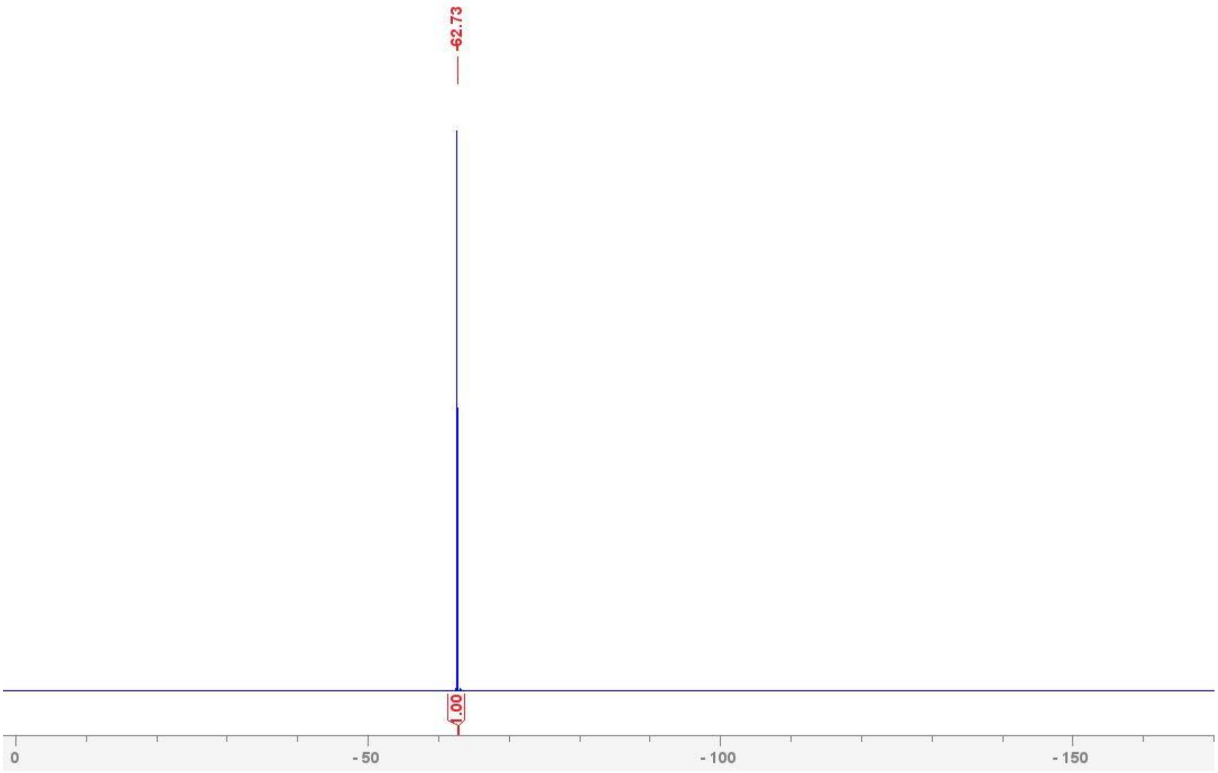
-62.73
1.00
0
- 50
- 100
- 150

3-Iodo-4-methyl-*N*-(3-(trifluoromethyl)phenyl)benzamide (**30S b**)

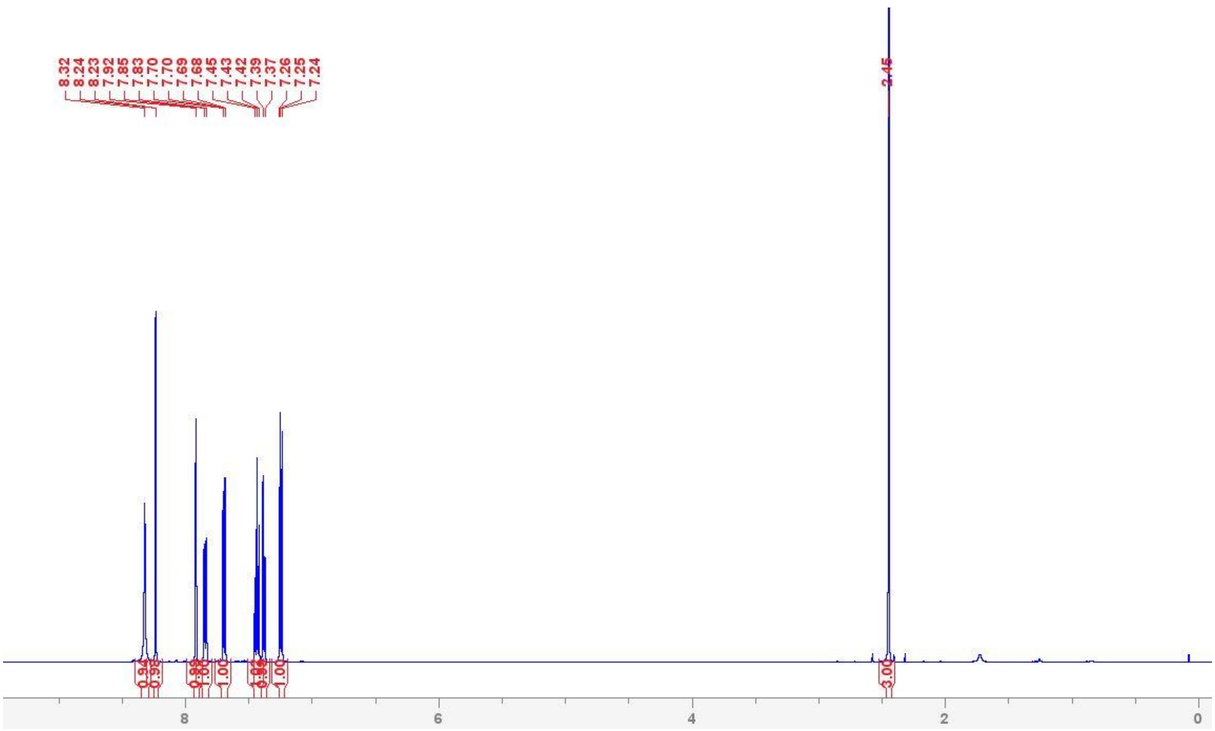

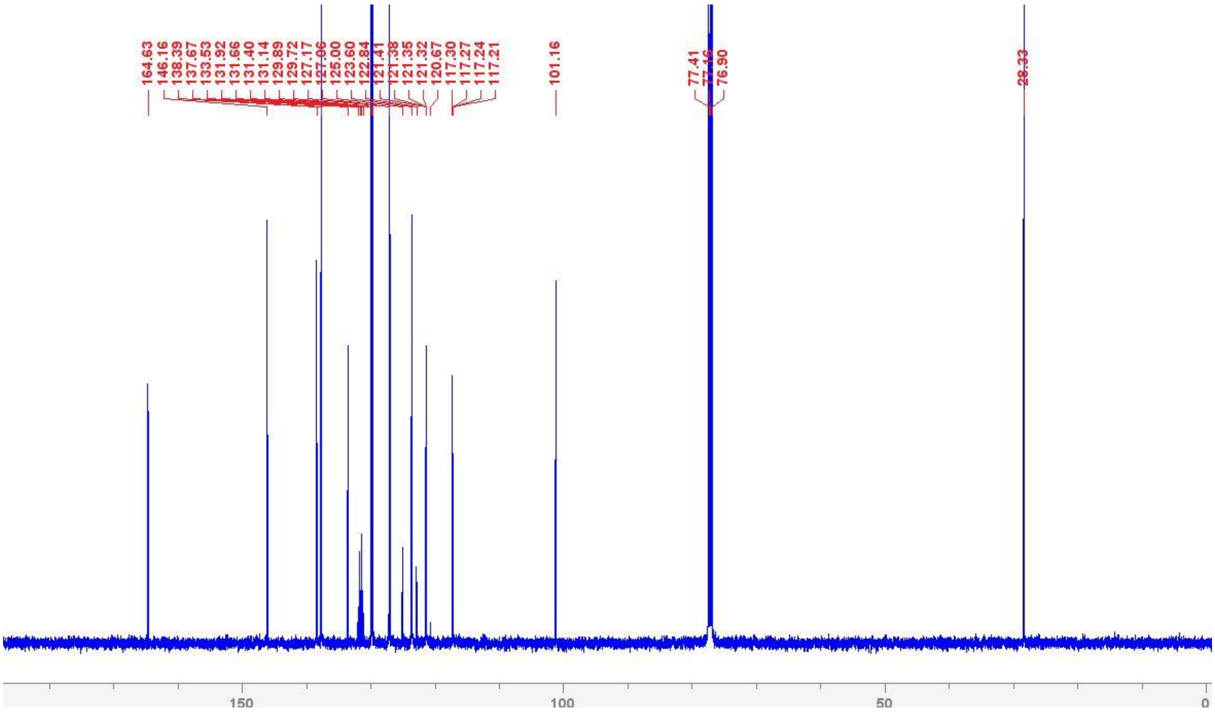

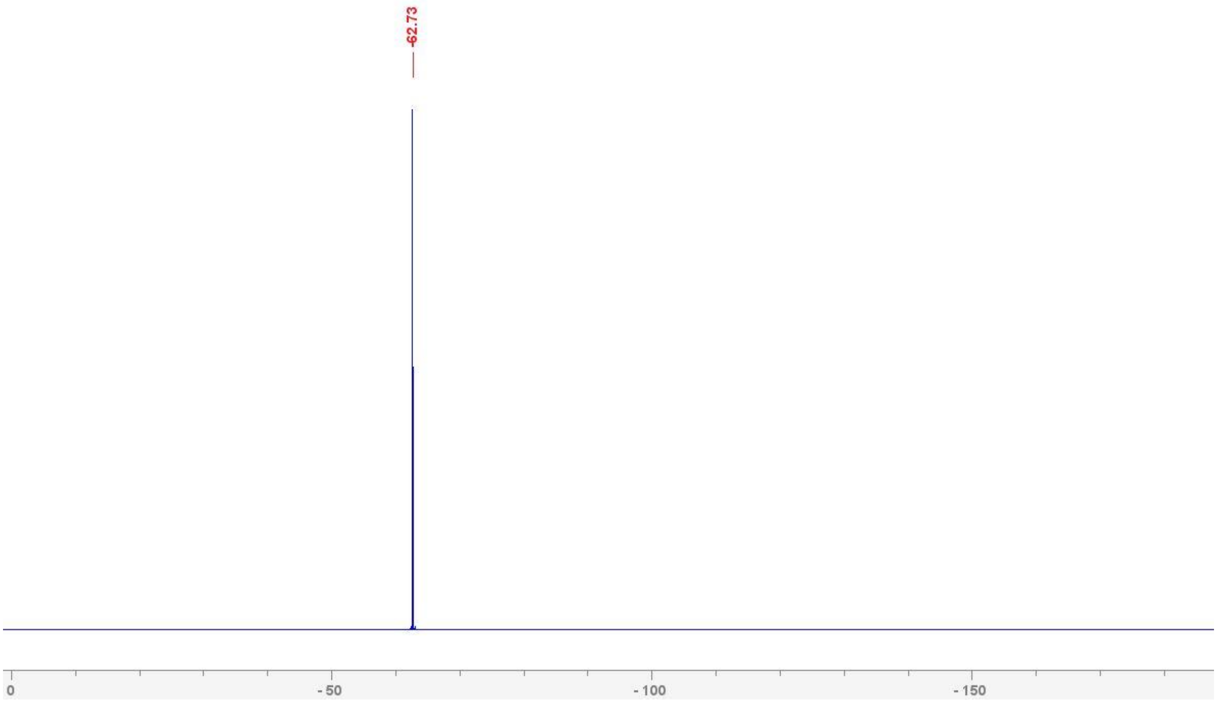
-62.73
0
- 50
- 100
- 150

5-Iodo-2-methyl-*N*-(3-(trifluoromethyl)phenyl)benzamide (**30S c**)

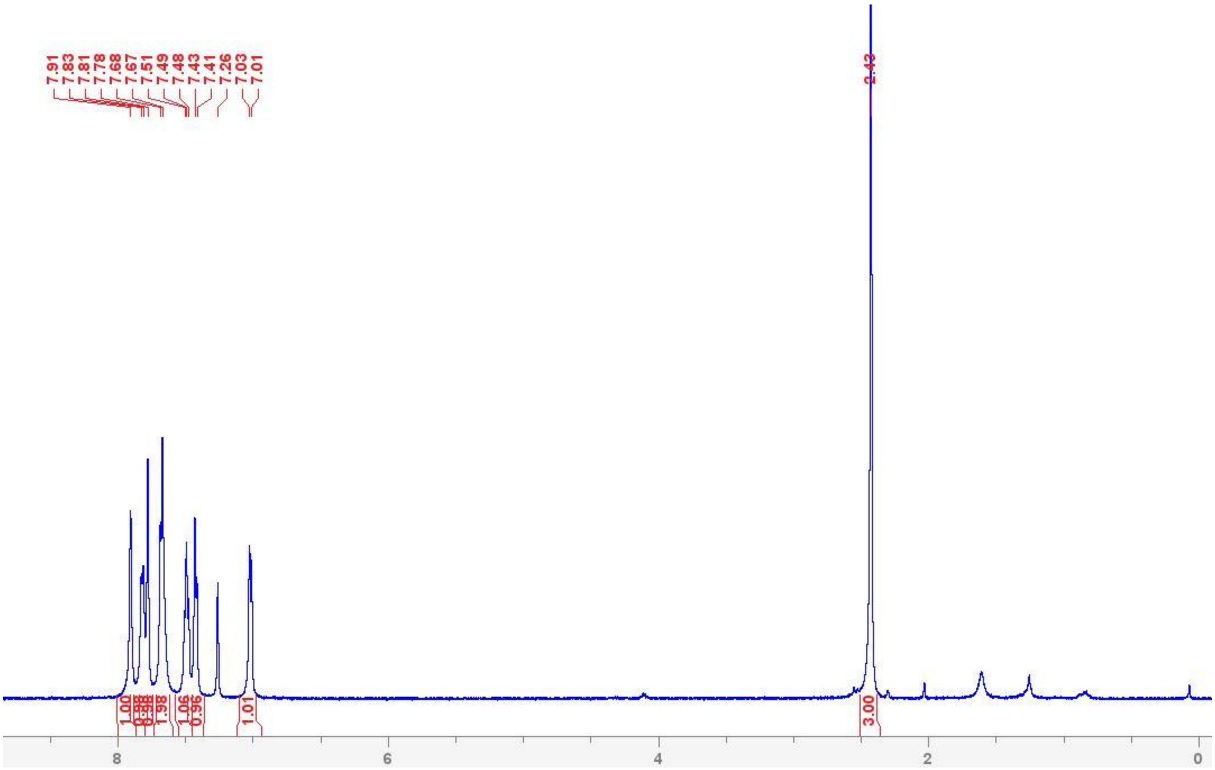

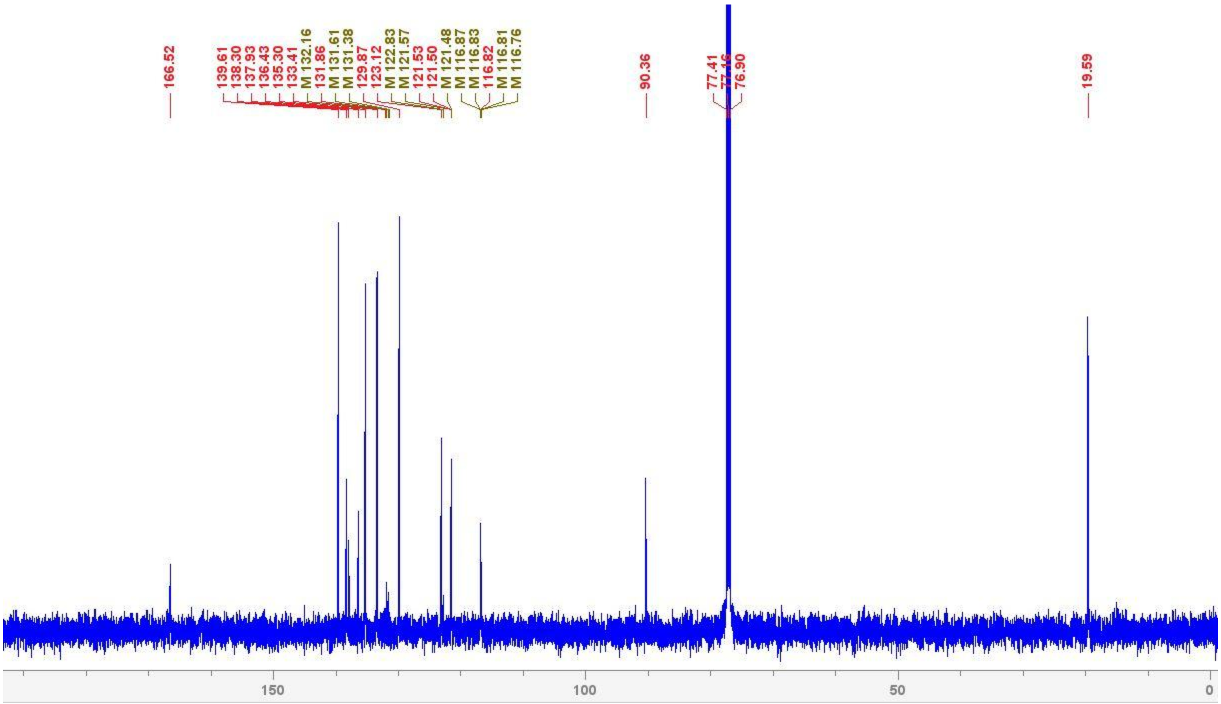

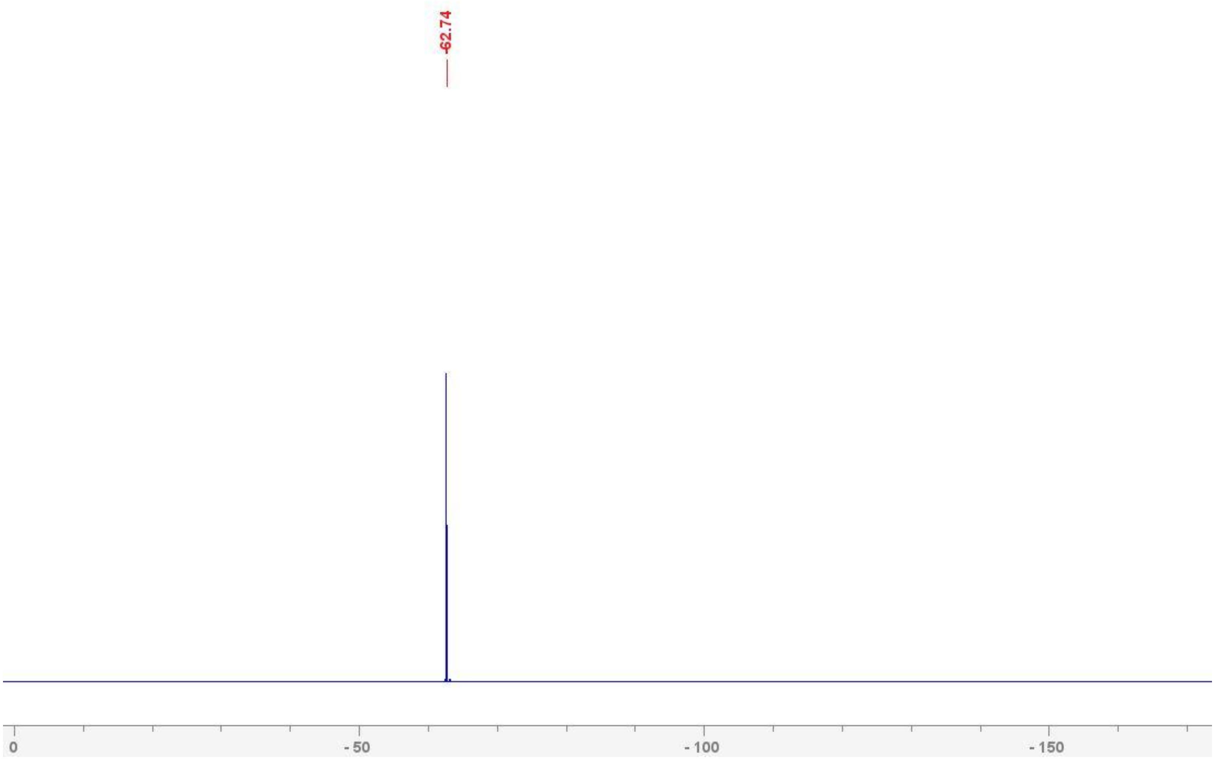
-62.74
0
- 50
- 100
- 150

3-Iodo-2,4-dimethyl-*N*-(3-(trifluoromethyl)phenyl)benzamide (**30S d**)

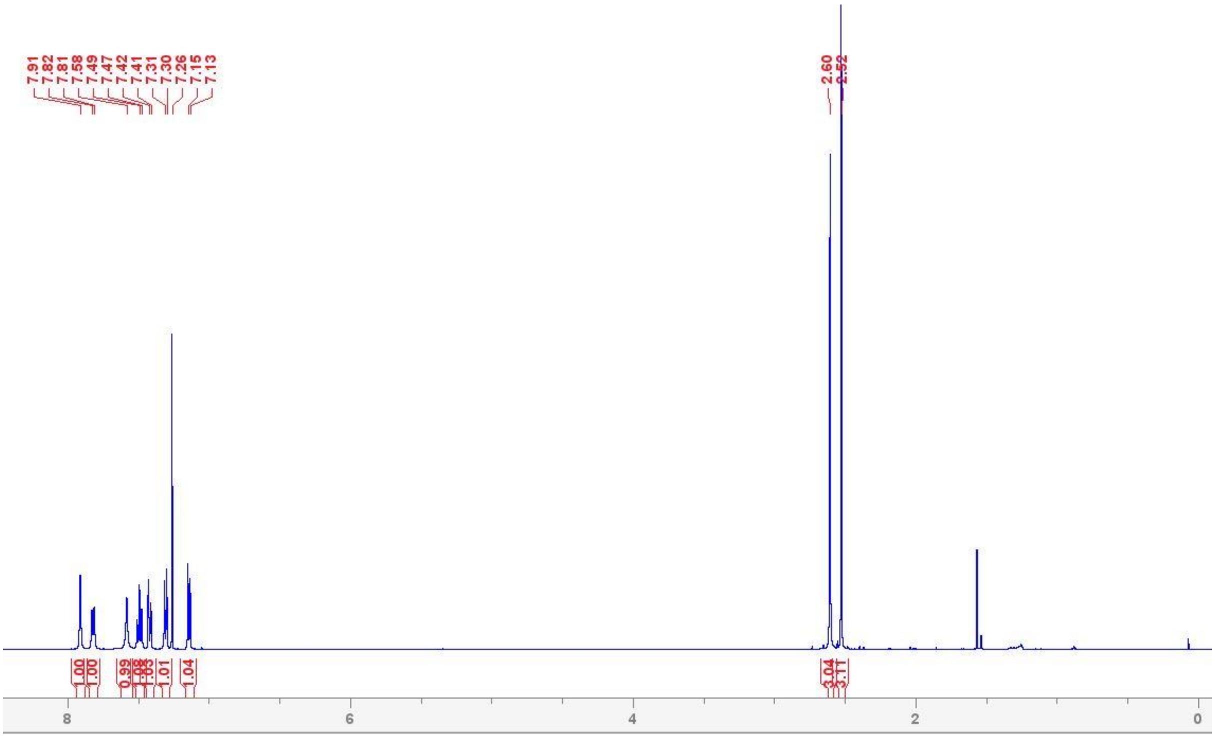

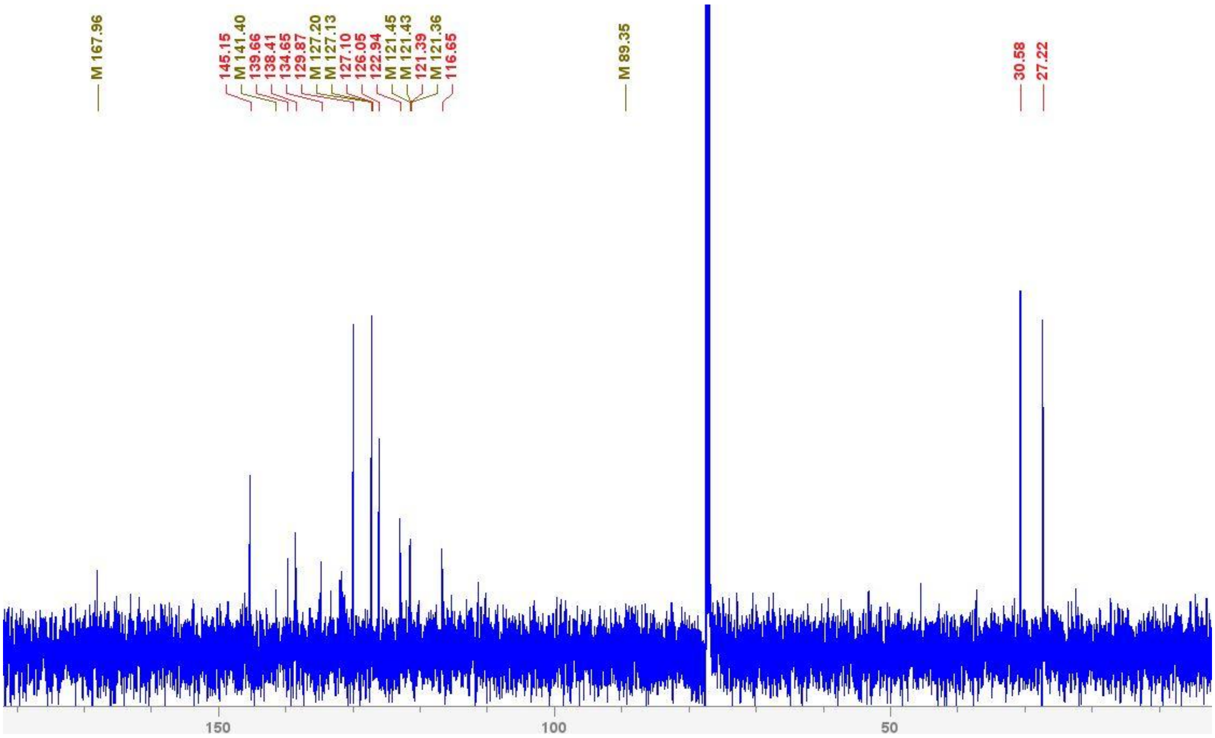

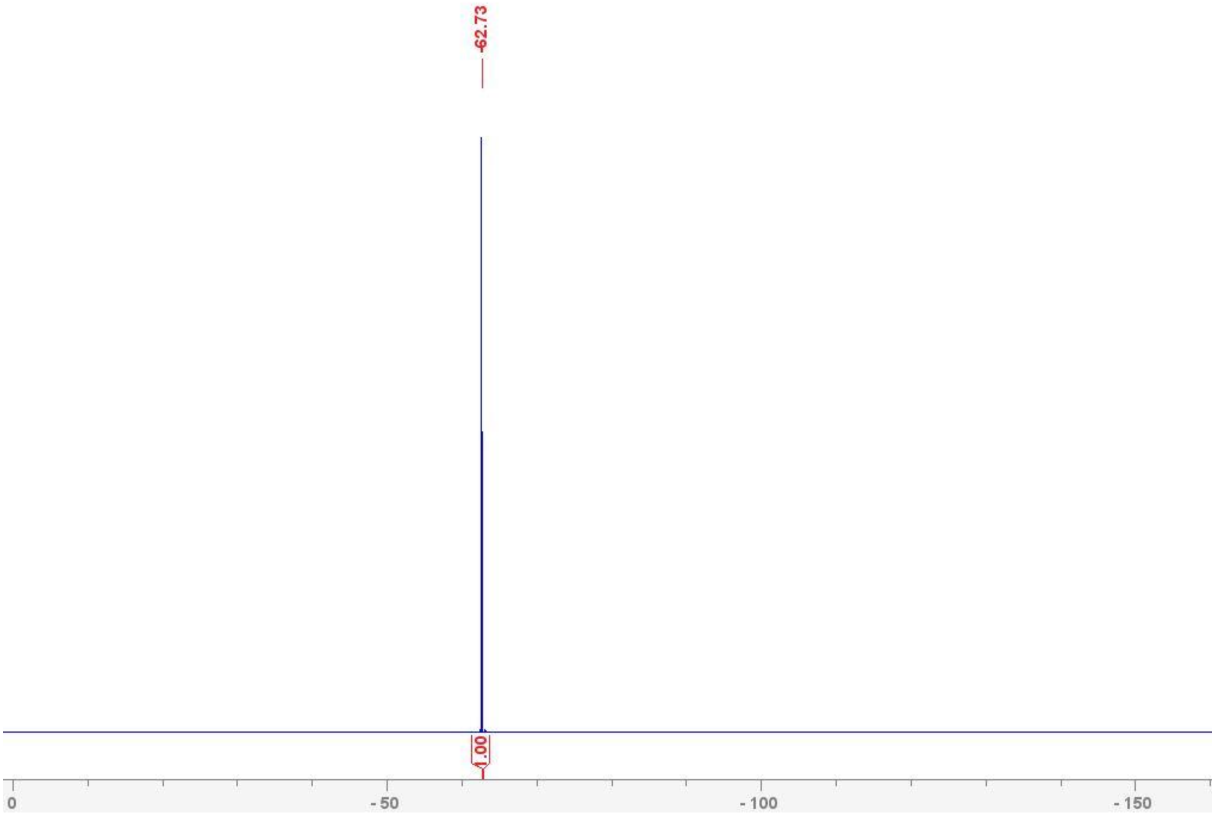
-62.73
1.00
0
- 50
- 100
- 150

References of the Supporting Information

[S1] A. P. Hnatiuk, A. A. N. Bruyneel, D. Tailor, M. Pandrala, A. Dheeraj, W. Li, R. Serrano, D. A. M. Feyen, M. M. Vu, P. Amatya, S. Gupta, Y. Nakauchi, I. Morgado, V. Wiebking, R. Liao, M. H. Porteus, R. Majeti, S. V. Malhotra, M. Mercola, *Cancer Res* **2022**, *82*(15), 2777-2791.
